# Insights into the Evolution of Shells and Love Darts of Land Snails Revealed from Their Matrix Proteins

**DOI:** 10.1093/gbe/evy242

**Published:** 2018-11-02

**Authors:** Keisuke Shimizu, Kazuki Kimura, Yukinobu Isowa, Kenshiro Oshima, Makiko Ishikawa, Hiroyuki Kagi, Keiji Kito, Masahira Hattori, Satoshi Chiba, Kazuyoshi Endo

**Affiliations:** 1 Department of Earth and Planetary Science, The University of Tokyo, Hongo, Japan; 2 College of Life and Environmental Sciences, University of Exeter, United Kingdom; 3 Department of Environmental Life Sciences, Graduate School of Life Sciences, Tohoku University, Sendai, Miyagi, Japan; 4 Research Institute for Ulleungdo and Dokdo Islands, Kyungpook National University, Bukgu, Daegu, Korea; 5 Organization for the Strategic Coordination of Research and Intellectual Properties, Meiji University, Kawasaki, Kanagawa, Japan; 6 Center for Omics and Bioinformatics, Department of Computational Biology and Medical Sciences, Graduate School of Frontier Sciences, The University of Tokyo, Kashiwa, Chiba, Japan; 7 Faculty of Animal Health Technology, Yamazaki University of Animal Health Technology, Hachioji, Tokyo, Japan; 8 Geochemical Research Center, Graduate School of Science, The University of Tokyo, Hongo, Japan; 9 Department of Life Sciences, School of Agriculture, Meiji University, Kawasaki, Kanagawa, Japan; 10 Cooperative Major of Advanced Health Science, Graduate School of Advanced Science and Engineering, Waseda University, Japan

**Keywords:** biomineralization, evolution, co-option, gastropods

## Abstract

Over the past decade, many skeletal matrix proteins that are possibly related to calcification have been reported in various calcifying animals. Molluscs are among the most diverse calcifying animals and some gastropods have adapted to terrestrial ecological niches. Although many shell matrix proteins (SMPs) have already been reported in molluscs, most reports have focused on marine molluscs, and the SMPs of terrestrial snails remain unclear. In addition, some terrestrial stylommatophoran snails have evolved an additional unique calcified character, called a “love dart,” used for mating behavior. We identified 54 SMPs in the terrestrial snail *Euhadra quaesita*, and found that they contain specific domains that are widely conserved in molluscan SMPs. However, our results also suggest that some of them possibly have evolved independently by domain shuffling, domain recruitment, or gene co-option. We then identified four dart matrix proteins, and found that two of them are the same proteins as those identified as SMPs. Our results suggest that some dart matrix proteins possibly have evolved by independent gene co-option from SMPs during dart evolution events. These results provide a new perspective on the evolution of SMPs and “love darts” in land snails.

## Introduction

A variety of calcifying organisms have evolved ever since the Cambrian. “Calcification” was a key morphological innovation that allowed for the diversification of metazoan life, because mineralized structures play various roles such as support for soft body parts, as devices for feeding or sensing, and as protection against predators or extreme environments. In the past decade, “omics” approaches have advanced considerably, making it possible to analyze the molecular basis of interesting phenomena in both model and nonmodel organisms. Recently, many skeletal matrix proteins that may be related to calcification have been identified by integrating transcriptome or expressed sequence tag analysis and mass spectrometric peptide analysis (e.g., corals, Ramos-Silva et al. 2013; molluscs, Marie et al. 2010; brachiopods, Jackson et al. 2015; sea urchins, Mann et al. 2008).

Molluscs are among the most diverse calcifying animals. Most calcifying organisms live in an aquatic environment, where calcium and carbonate ions are easily available, whereas one group of molluscs, the gastropods, and only a few other calcifying animal taxa, including vertebrates, have adapted to terrestrial ecological niches. Thus, molluscs are a good model to study various aspects of the evolution of biomineralization. In particular, gastropods provide a unique opportunity to study the evolution of shell matrices in relation to the transition from aquatic to terrestrial habitats. Since terrestrial environments differ from aquatic ones in various aspects including humidity, pH, temperature, and the availability of calcium ions, land snails have acquired novel features such as lungs instead of gills. Thus, it is possible that they have also changed their shell matrix proteins (SMPs) accordingly in the process of adapting to terrestrial environments.

**Fig. 1.**
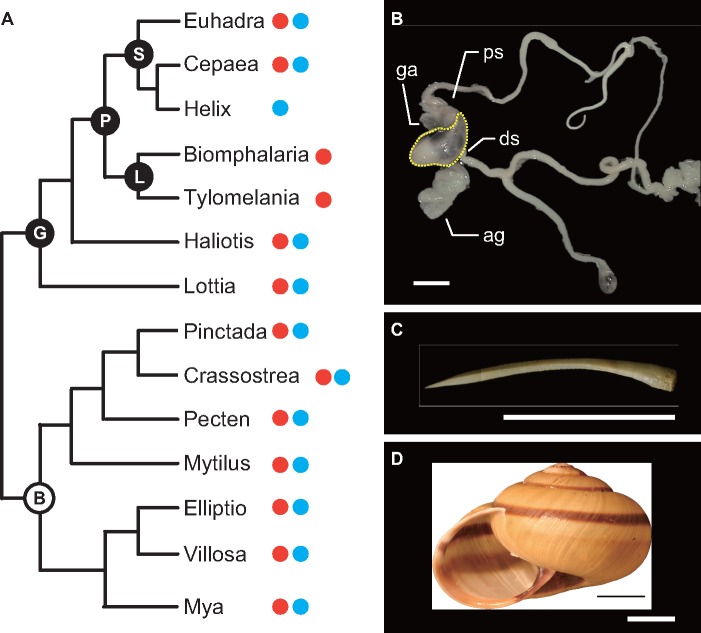
—Phylogeny and morphology of *Euhadra quaesita*. (*A*) Phylogeny of molluscs that have been published mantle transcriptome or SMP analysis. Red circles indicate transcriptome analysis using the mantle tissues. Blue circles indicate proteome analysis using the shells. B, Bivalvia; G, Gastropoda; L, Lymnaeoidea; P, Pulmonata; S, Stylommatophora. (*B*) Reproductive organs. Yellow broken lines indicate the dart sac. ag, accessory glands; ds, dart sac; ga, genital atrium; ps, penial sheath. (*C*) Morphology of the dart. (*D*) Morphology of the shell. All scales are 10 mm.

The pulmonate land snails represent one of the most diverse groups of gastropods (Tillier et al. 1996). Although SMPs or shell-related genes have already been reported from a number of molluscan species (abalone, *Haliotis asinina*, Marie et al. 2010; limpet, *Lottia gigantea*, Mann et al. 2012; oyster, *Crassostrea gigas*, Zhang et al. 2012; pearl oyster, *Pinctada margaritifera* and *P. maxima*, Marie et al. 2012; mussel, *Mytilus galloprovincialis*, Gao et al. 2015, and *M. edulis*, Liao et al. 2015, clam, *Mya truncata*, Arivalagan et al. 2016; king scallop, *Pecten maximus*, Arivalagan et al. 2017; fresh water mussels, *Elliptio complanata* and *Villosa lienosa*, Marie et al. 2017), reports for terrestrial snails remain relatively scarce, with only two studies having reported the SMPs of land snails for *Helix aspersa* (Pavat et al. 2012) and *Cepaea nemoralis* (Mann and Jackson 2014) (fig. 1*A*). Pavat et al. (2012) reported the biochemical properties of SMPs and 14 partial peptides (4–11 amino acid residues) from *H. aspersa*, showing that the repertoire of these proteins differs greatly from that of marine molluscan shell proteins. In addition, Mann and Jackson (2014) performed both proteome and transcriptome analyses for the grove snail *Cepaea nemoralis* and reported 59 major SMPs. Interestingly, more than half of these proteins (52.5%) were classified as uncharacterized and/or novel proteins (Mann and Jackson 2014). However, it is uncertain whether some of these novel SMPs are common to all land snails or are specific to this species.

Molluscs have evolved hard structures other than the shell. For instance, the radula, or the chitinous teeth for feeding, was acquired in the last common ancestor of molluscs and was lost in bivalves. Interestingly, some genes that are expressed in the radula-forming region (ventral outpocketing of the foregut) (Page 2002; Page and Hookham 2017) are shared with genes related to other hard tissues (chaetae, spicules, and shells) in Lophotrochozoa (Hilgers et al. 2018). Hilgers et al. (2018) provided new insights into the genetic basis of radula formation and suggested that the lophotrochozoan hard structures likely evolved by gene co-option. As another instance of molluscan hard structures, some land snails produce a “love dart” that is composed of calcium carbonate (Tompa 1980). The love dart is a device associated with reproductive behavior and is formed in a dart sac, which has muscle cells and different types of secretory cells (Koene et al. 2013) (fig. 1*B* and *C*). Snails pierce the body wall of a partner with the dart and transfer bioactive substances covering the dart during mating: this curious behavior is known as “dart shooting” (Adamo and Chase 1988; Chase 2007). Although the substances do not contain sperm, they induce physiological changes in the mating partner and increase fertilization success of the sperm donated by the dart shooter (Chase 2007). Interestingly, this reproductive trait has evolved repeatedly in land snails (Davison and Mordan 2007), and its evolution is an example of a coevolutionary arms race (Koene and Schulenburg 2005). Several studies on the functional aspects of dart shooting have been performed on species within the families Helicidae and Bradybaenidae (Chase 2007; Baur 2010; Kimura et al. 2014). *Euhadra quaesita*, which belongs to the Bradybaenidae, is one of the species used in those works (Kimura et al. 2013; Kimura and Chiba 2015) and our preliminary investigations have revealed that this snail discards its dart after a single mating event and produces a new one (*N* > 30: Kimura K, unpublished data). Preliminary observations have also revealed that it takes 6–7 days to complete dart formation in *E. quaesita* (Kimura K, unpublished data). Although their curious morphological evolution has attracted attention in the field of behavioral ecology, especially as a sexually selected behavior in hermaphrodites (Chase 2007; Baur 2010; Kimura et al. 2014), the genes or matrix proteins that correlate with dart formation have been ignored.

Here, we performed a combined transcriptome and proteome analysis in order to identify the SMPs in the terrestrial snail *Euhadra quaesita*. Comparisons of SMPs between two terrestrial snail species (*E. quaesita* and *Cepaea nemoralis*) and other marine molluscs have made it possible to investigate the kinds of SMPs that evolved in the common ancestor of these two terrestrial snails. Furthermore, we analyzed the dart matrix proteins (DMPs) of *E. quaesita*, and compared them with molluscan SMPs to infer whether DMP genes have evolved by gene co-option or represent novel genes.

## Materials and Methods

### Animal Collection and RNA Extraction

We collected the sinistral snail *Euhadra quaesita*, in Sendai, Miyagi Prefecture, Japan*.* This species shows determinate growth, and the adult shell size (shell diameter) is ∼35–45 mm (fig. 1*D*). Their love darts are in the range of ∼1.1–1.9 mm in length (fig. 1*C*). We cut out the mantle tissues of an immature snail and stored them in ISOGEN (Nippon Gene Co. Ltd, Tokyo, Japan) at −80 °C. We also dissected out the dart sac tissues from mature snails that had experienced a mating in the lab 48 h before the operation and were just making new darts; we stored them in ISOGEN at −80 °C*.* Total RNA was extracted from each of the two different tissues (mantle and dart sac) of *E. quaesita* according to the manufacturer’s protocols for RNA extraction using ISOGEN and RNeasy (Quiagen, 74104), and stored at −80 °C until used for complementary DNA (cDNA) synthesis and transcriptome analysis (mantle RNA: 100 µg/ml, dart sac RNA: 94.8 µg/ml).

### Transcriptome Analysis

We prepared 100-bp DNA libraries from the mRNA samples (1–5 μg each sample) that were extracted from the mantle and the dart sac tissues using an Ion Total RNA-seq Kit v2 (Thermo Fisher Scientific) according to the manufacturer’s protocols, and analyzed them using an Ion 318 v2 chip of the Ion Torrent PGM sequencer (Thermo Fisher Scientific), then performed 100-base single-end sequencing. We obtained a total of 6,056,290 and 5,351,015 raw reads from the mantle and the dart sac tissues, respectively (supplementary table S1, Supplementary Material online). We then combined these reads and assembled them using Newbler v2.8 (Roche, Basel, Switzerland) under default conditions for cDNA assembly (runAssembly -o output -cdna -large sff-file), and obtained a total of 74,293 contigs. Quality of the assembled sequences was calculated with the BUSCO v2 (Simao et al. 2015) (supplementary table S1, Supplementary Material online). We then filtered the contigs to collect contigs longer than 100 bp (59,618 contigs), and used them for our analyses. These shot-gun sequences (DRA006965 and DRA006966) and assembled sequences (PRJDB6927: IADG01000001–IADG01059618) are available in the DNA Data Bank of Japan (DDBJ).

### Comparison of Transcriptomes between Mantle and Dart Sac Tissues

We mapped the transcripts for each of the RNAseq reads obtained from the mantle and dart sac samples back to the master assembly using TopHat2 (Trapnell et al. 2009; Kim et al. 2013). We then calculated the number of fragments per kilobase of exon per million mapped reads (FPKM) for each contig in each sample, and filtered the contigs by the expression level (FPKM > 1). To find similar sequences to our transcriptomes, we performed BLASTX searches using the nonredundant protein sequence databases of GenBank (http://blast.ncbi.nlm.nih.gov; last accessed January 25, 2019; Altschul et al. 1990), with the e-value cut-off at 1.0e-5. We also searched for characteristic domains against the Pfam protein domain database (https://pfam.xfam.org; last accessed January 25, 2019; Finn et al. 2016) using HMMER (v3.1b2, http://hmmer.org; last accessed January 25, 2019; Krogh et al. 1994; Durbin et al. 1998; Eddy 1998, e-values<1.0e-5).

### cDNA Synthesis and Gene Cloning

Investigation of SMP sequences within transcriptome data sets revealed that 17 of the corresponding contigs possessed potential frame shifts. To clarify the correct sequence, a part of the total RNA extracted from each of the mantle and dart sac tissues was used for cDNA synthesis. cDNA was synthesized using ReverTra Ace (Toyobo, Osaka, Japan) according to the manufacturer’s protocols. Gene sequences for 17 SMP were amplified with PCR using primers designed with reference to the transcriptome data (supplementary table S2, Supplementary Material online). After purification of PCR products using the SV Gel Extraction and PCR Clean-Up system (Promega, A9281, WI), amplicons were ligated into the pGEM-T easy vector using a DNA ligation kit (Promega, A1360), and were used to transform competent *Escherichia coli* DH5alpha cells (Toyobo, DNA-901). Inserts of the vectors were sequenced by ABI3130 (Applied Biosystems, CA) with the standard protocols using T7 and SP6 primers.

### Preparation of Matrix Proteins

We cut out the dart sacs from mature snails and placed them for 48 h in an aqueous solution of 2N NaOH, which dissolved all the tissue but the intact dart. The shells and love darts (5 and 1 g, respectively) were briefly washed with deionized water and separately crushed to fine powder in an agate mortar. These powders were washed with 5% sodium hypochlorite with gentle shaking for 2 h at room temperature with 5-min sonication and a change of the bleach solution every 30 min. After this bleach treatment, the shell and dart powders were washed with ultrapure water and dried. After decalcifying the shell and dart powder with 0.5 M EDTA (pH 8.0) at a ratio of 23 ml to 1 g powders at 4 °C, we separated the EDTA-soluble matrix from the EDTA-insoluble matrix by centrifuging at 20,000 ×g for 1 h at 4 °C. The supernatant solution containing soluble matrix was desalted by an Amicon Ultra-15 centrifugal filter unit with an Ultracel-3 membrane (Millipore, Billerica, CA), and the resultant desalted and concentrated solution was used for protein analyses using SDS–PAGE and liquid chromatography-tandem mass spectrometry (LC-MC/MC). The insoluble matrix fractions were washed with distilled water and then dissolved in a buffer containing 9 M urea and 2% (v/v) Triton X-100 for 5 min at 100 °C. After centrifugation at 20,000 ×g for 1 h at 4 °C, the supernatant was subjected to protein analyses using SDS–PAGE and LC-MC/MC. SDS–PAGE was performed with 12% polyacrylamide gel (Mini-protean TGX Precast Protein Gels, BioRad, CA). We loaded 0.7–1 µg each of SM and IM samples on the gel, and detected the proteins by silver staining.

### Proteome Analysis Using LC-MS/MS

We performed a shotgun approach to identify matrix proteins. The detailed procedures of tryptic peptide preparation and LC-MS/MS analysis are described in a previous study (Isowa et al. 2015). The method is briefly as follows. The proteins extracted from shells or love darts were precipitated by methanol/chloroform and dissolved in 8 M urea, 0.1 M Tris–HCl (pH 8.5). Cysteine reduction and alkylation were then performed using dithiothreitol and iodoacetamide, respectively. After decreasing the urea concentration to 2 M, digestion into peptides was performed by the addition of sequencing grade modified trypsin (Promega). LC-MS/MS analysis was carried out using a *DiNa* nanoLC system (KYA Technologies, Tokyo, Japan) and a LTQ Orbitrap mass spectrometer (Thermo Fisher Scientific). The resultant MS/MS spectra were subjected to a database search against the protein sequence database translated from the combined transcriptome data of mantle and dart sac tissues using the SEQUEST program in Proteome Discoverer version 1.2 or 1.3 (Thermo Fisher Scientific). In this process, the combined transcriptome sequences of mantle and dart sac tissues containing 74,293 contigs were translated into the protein sequences to generate protein sequence databases, which were digested into peptides by trypsin in silico to calculate the theoretical mass of peptides and MS/MS spectra. The measured mass of each peptide was compared with the theoretical values to find candidate peptide sequences. Among them, the correlation between measured and theoretical MS/MS spectra was calculated and peptide sequences having the top scores were determined as peptide-spectral matching (PSM). We estimated the false discovery rate (FDR) for peptide-spectral matches (PSMs) above any scores with the target-decoy method (Elias and Gygi 2007) by Proteome Discover (version 1.3). We removed the low confidence PSMs and used the list of retained PSMs (FDR < 0.01) for the final protein identification. Parameters used for identification processes described earlier were the same as those in a previous study (Isowa et al. 2015). The MS/MS spectra acquired from analyses of shell extracts were also searched against the protein sequences from corrected transcript sequences without frame shift errors, which were found in the original transcriptome data and reanalyzed by Sanger sequencing using an ABI3130 (Applied Biosystems). If a peptide matched two or more potential protein sequences, we used all potential proteins for our analysis. Two unique peptides were required to identify a matrix protein in this study. The abundance of each protein in the shells was estimated as the abundance index calculated from the number of identified MS/MS spectra using normalization based on the theoretical number of the peptide fragments generated tryptic digestion.

### Characterizations of the Matrix Proteins

Sequences similarity searches of the matrix proteins were performed with the BLASTX program against the GenBank nonredundant protein database (e-value < 1.0e-5). We found the domain organization of the protein sequences by the online version of Simple Modular Architecture Research Tool (SMART; Letunic et al. 2015; Letunic and Bork 2018; http://smart.embl-heidelberg.de; last accessed January 25, 2019), including signal peptide prediction (SignalP; Petersen et al. 2011), Pfam domain search (Finn et al. 2016), transmembrane helices prediction (TMHMM; Krogh et al. 2001), and compositionally biased regions prediction (SEG; Wootton and Federhen 1996) (e-value < 1.0e-5).

### Phylogenetic Analysis

We performed molecular phylogenetic analysis on a total of 16 proteins obtained in this study, including well-known SMPs in other molluscs (C1q containing protein, dermatopontin, MSP130, and tyrosinase), specific SMPs in pulmonates (adipocyte plasma membrane-associated like protein and alkaline phosphatase), three SMPs of highest abundance in this species (Eq16217–21060, Eq23617–24364, and Eq21467), and common developmental proteins (bmp receptor, ferritin, frizzled, hes, notchless, and wnt). We used the sites of specific domains that were identified by the searches with HMMER (v3.1b2, http://hmmer.org; last accessed January 25, 2019, e-values < 1.0e-5) for the analyses except for three proteins that do not have a specific domain (Eq16217–21060, Eq21467, and MSP130), for which we used all sites. We conducted sequence alignments using the online version of MAFFT (v7.310; http://mafft.cbrc.jp/alignment/server/index.html; last accessed January 25, 2019; Katoh et al. 2002), and trimmed with TrimAl (1.2rev59) (Capella-Gutiérrez et al. 2009) (supplementary file S1, Supplementary Material online). The best-fit amino acid substitution model was inferred with MEGA (v5.1) (Tamura et al. 2011) and maximum likelihood trees were constructed with the online version of RAxML (Stamatakis 2014) using the best-fit amino acid substitution mode with 100 bootstrap replications.

### Raman Spectroscopy Analysis

Calcium carbonate polymorphs of the shell and the dart were identified from Raman spectroscopy. The samples were treated with 1% sodium hypochlorite overnight before Raman analysis. Raman spectra were obtained using a micro-Raman spectrometer equipped with a 50-cm single polychromator (500is; Chromex), an optical microscope (BX60X; Olympus Optical CO. Ltd.), a Si-based CCD detector with 1,024 × 128 pixels (DU-401-BR-DD SH, Andor Technology), and an Ar ion laser (514.5 nm, 543-AP-A01; Melles Griot) (Maruyama et al. 2017). Raman spectra were obtained from the surface of shell specimens in the range from 100 to 1,600 cm^−1^, which sufficiently covered the range to discriminate carbonate polymorphs (calcite, aragonite, or vaterite). The spectral resolution was ∼1.5 cm^−1^. Each Raman spectrum was obtained for 10 s at room temperature. The excitation laser beam was focused on a spheroidal spot of ∼2 × 2 × 10 µm in volume using a ×50 objective lens and the laser power was ∼5 mW at the sample surface of the intact shells and the darts. The bands from 100 to 300 cm^−1^ were fitted to Lorentzian functions by the Igor software package (WaveMetrix Co. Ltd.).

## Results and Discussion

### Raman Spectroscopic Analysis on the Shell and the Dart in *E. quaesita*

**Fig. 2.**
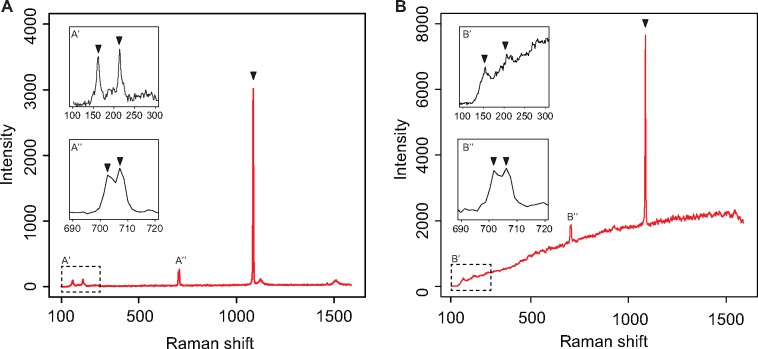
—Raman spectra of the shell and dart of *Euhadra quaesita*. Peaks at 154,205 cm^−1^ (lattice vibration), 702,706 cm^−1^ (in-plane bending), and 1,085 cm^−1^ (CO_3_ symmetric stretching) for shell (*A*) and the peaks at 153, 209, 702, 706, and 1,085 cm^−1^ for dart (*B*) are attributed to the Raman shifts specific to aragonite. Enlargements of these peaks are shown in the insets.

Calcite-specific Raman bands are observed at 152, 280, and 710 cm^−1^, whereas aragonite-specific ones are at 152 and 205, and doublet at 700 and 705 cm^−1^ (Kontoyannis and Vagenas 2000). Shell samples showed Raman bands at 154, 205, 702, 706, and 1,085 cm^−1^. Dart samples showed Raman bands at 153, 209, 702, 706, and 1,085 cm^−1^ (fig. 2). These data thus revealed that the major component of both the darts and the shells is aragonite.

### Transcriptome Results of the Mantle and Dart Sac Tissues

**Fig. 3.**
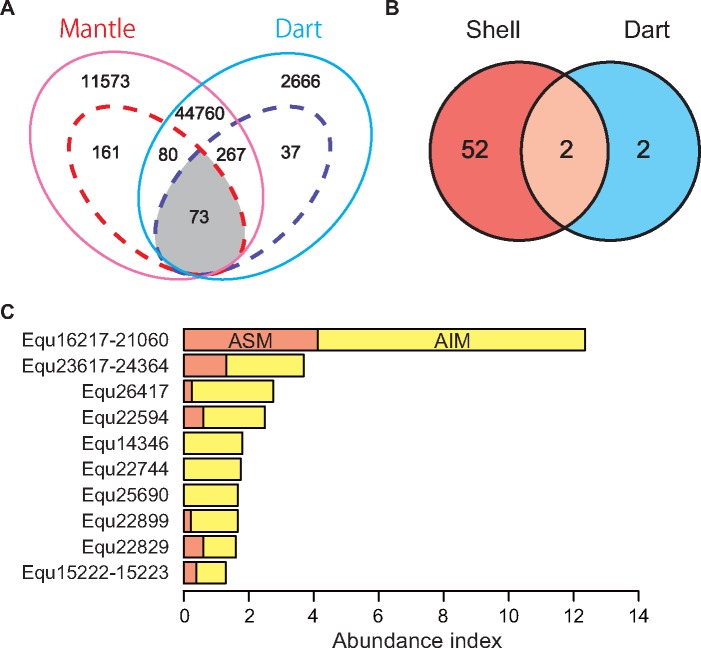
—Comparisons of the shell- and dart-related transcripts and proteins in *Euhadra quaesita*. Venn diagrams of transcripts and proteins that were identified by transcriptome (*A*) and LC-MS/MS analyses (*B*). Dashed lines indicate the highly expressed transcripts (FPKM >1,000). (*C*) The ten most abundant SMPs (high abundance index values) in *E. quaesita*. ASM, acid-soluble matrix; AIM, acid-insoluble matrix.

Our BLASTX searches indicated that 12,520 transcripts (21%) encode proteins significantly similar to known proteins in the database, while the remaining 47,097 transcripts (79%) encode novel proteins (supplementary table S3, Supplementary Material online). In this study, we defined the highly expressed transcripts by expression level (FPKM > 1,000). We identified 161 transcripts that are highly expressed in the mantle (FPKM > 1,000) and poorly expressed in the dart sac (FPKM < 1) (fig. 3*A* and supplementary table S3, Supplementary Material online). In contrast, we identified 37 transcripts that are highly expressed in the dart sac (FPKM > 1,000) and poorly expressed in the mantle (FPKM < 1) (fig. 3*A* and supplementary table S3, Supplementary Material online).

Mineralized tissues evolved independently in many bilaterian lineages during the early Cambrian. However, their developmental bases are still unclear. Interestingly, some transcription factors and signal molecules commonly involved in hard tissue development of bilaterians appear to have evolved by gene co-option. For instance, Dpp (BMP2/4) is a key signal in hard tissue formation in molluscs (Nederbragt et al. 2002; Shimizu et al. 2011 2013; Hashimoto et al. 2012) and in vertebrates (Chen et al. 2012). In gastropods, Dpp regulates the expression of chitin synthase and *ferritin* in the shell field (Hashimoto et al. 2012), and is responsible for asymmetric shell growth (Shimizu et al. 2011, 2013) and operculum formation (Hashimoto et al. 2012). We did not find *dpp* transcripts in this study, however we found that the *BMP type II receptor* (*bmpr2*) like transcript (contig62837) is expressed in the mantle and dart sac (supplementary tables S3 and S4 and fig. S1, Supplementary Material online). Those observations suggest that Dpp signaling is likely related with some of the hard tissue development (shell, operculum, and dart) in gastropods.

In a recent study, Hilgers et al. (2018) reported the genetic basis of radula formation and compared transcripts between the mantle and radula-forming tissues (the ventral out-pocketing of the foregut region). They showed that some transcription factors specifically expressed in the radula are known to be developmental genes of hard tissue formation in other lophotrochozoans (*hes1*, *arx*, *gbx*, and *heph*). We did not find transcripts of those genes except for *hes1* in the mantle or in the dart sac. *Hes1* is a downstream gene of Notch signaling pathway, and is expressed in the chitin-based chaetae forming regions in annelids (Gazave et al. 2014) and brachiopods (Schiemann et al. 2017). Notch signaling regulates many morphogenetic processes, similar to the Wnt signaling pathway. The Notch signal is transduced by the ligands Delta or Jagged and regulates the expression of many target genes. We found *hes1* and *notchless* genes, which are related to the Notch signaling pathway in the transcriptomes of the mantle and dart sac (supplementary tables S3 and S4 and fig. S2, Supplementary Material online). However, we did not find the ligands (Delta and Jagged) or the receptor (Notch) of this pathway in our transcriptome data. Notchless is known as a regulator of Notch signaling activity in the fruit fly (Royet et al. 1998). In the abalone, *delta* is expressed in the shell field, suggesting that the Notch signaling pathway is related to the specification of the shell-secretary cells in gastropods (Jackson and Degnan 2016). Thus, the Notch signaling pathway could play key roles in the specification of secretary cells of the shell and dart. Additionally, we found members of other signaling pathways (Wnt: *wnt-5* and *frizzled*, Hh: *hh*) in the mantle and/or dart sac (supplementary tables S3 and S4 and figs. S3–S5, Supplementary Material online). However, these transcripts indicated very low expression levels (supplementary tables S3 and S4, Supplementary Material online).

We found another interesting commonality in the mantle and dart sac transcriptomes from other molluscs biomineralizing tissues, in addition to the transcription factors and signaling molecules. *Ferritin* is a shell and operculum marker gene in the larvae of gastropods (Jackson et al. 2007; Hashimoto et al. 2012) and is highly expressed (FPKM > 1,000) both in the mantle tissue and in the dart sac (contig15651 and contig15652) (supplementary tables S3 and S4 and fig. S6, Supplementary Material online). Our observation suggests that *ferritin* is likely involved in the formation of hard tissues including the dart. In addition, we found many kinds of transcripts that have already been reported as shell-related genes both in the mantle and in the dart sac (1<FPKM, e.g., *carbonic anhydrase* and *dermatopontin*) (supplementary table S4, Supplementary Material online).

### Annotation of SMPs and DMPs in *E. quaesita*

We extracted SMPs and DMPs using EDTA and separated two matrices, acid-soluble organic matrix (ASM) and acid-insoluble organic matrix (AIM). ASM and AIM were analyzed by PAGE and silver staining to quality of extracted proteins (supplementary fig. S7, Supplementary Material online)*.* We then performed shotgun proteome analysis using LC-MS/MS for those ASM and AIM fractions. As a result of the integration of the proteomic (supplementary tables S5 and S6, Supplementary Material online) and mantle and dart sac transcriptomic results, we identified 54 SMPs and four DMPs from the terrestrial snail *E. quaesita* (fig. 3*B*, table 1, and supplementary file S2, Supplementary Material online). Out of the 54 SMPs, 37 SMPs were obtained only from the AIM fraction, two SMPs were obtained only from the ASM fraction, and 15 SMPs were obtained from both the ASM and AIM fractions (table 1). Three of the four DMPs were obtained only from the AIM fraction and the remaining one was obtained from both the ASM and AIM fractions (table 1). In order to annotate SMPs and DMPs, we performed a BLASTX search against the GenBank nonredundant protein database, and found that 28 SMPs have significant similarities to known proteins in the database and the remaining 26 SMPs are uncharacterized or novel proteins (table 1).

**Table 1:** The Shell Matrix Proteins and Dart Matrix Proteins of *Euhadra quaesita*

		Best BLASTX Hits of the Protein							
**Sample**	**Contig ID**	**Accession No.**	**Description**	**Species**	**Domain**	**Matrix**	**SP**	**TM**	**RLCDs**
Shell	588	No hit	—	—	—	AIM	—	—	**✓**
Shell	2505	ACD84942	Beta-actin	*Macrocentrus cingulum*	Actin	AIM	—	—	—
Shell	2555	BAB20937	Actin	*Octopus vulgaris*	Actin	AIM	—	—	—
Shell	10628	No hit	—	—	—	AIM	**✓**	—	**✓**
Shell	10634	XP_013087207	Uncharacterized protein	*Biomphalaria glabrata*	CBM_14	AIM/ASM	—	—	—
Shell	10941	XP_005107380	Serpin B6-like	*Aplysia californica*	SERPIN	AIM	—	—	—
Shell	11259	XP_013077192	Uncharacterized protein	*Biomphalaria glabrata*	—	AIM	—	—	**✓**
Shell	11340	CAD83837	Tyrosinase-like	*Biomphalaria glabrata*	Tyrosinase	AIM	**✓**	—	**✓**
Shell	12236	No hit	—	—	—	AIM	—	—	—
Shell	14131	No hit	—	—	—	AIM	—	**✓**	—
Shell	14133	ABR68007	Matrilin-like	*Ambigolimax valentianus*	VWA	AIM	—	—	—
Shell	14346	No hit	—	—	—	AIM	**✓**	—	**✓**
Shell	20937	XP_013085093	Uncharacterized protein	*Biomphalaria glabrata*	—	AIM/ASM	—	**✓**	—
Shell	20976	No hit	—	—	—	AIM	—	—	**✓**
Shell	20977	XP_013077192	Uncharacterized protein	*Biomphalaria glabrata*	—	AIM	—	—	**✓**
Shell	20990	XP_013067348	Elongation factor 1α	*Biomphalaria glabrata*	GTP_EFTU	AIM	—	—	—
Shell	20996	XP_013079725	Mucin-2-like	*Biomphalaria glabrata*	—	AIM	—	—	**✓**
Shell	21047	XP_013070259	Voltage-dependent anion-selective channel protein	*Biomphalaria glabrata*	Porin_3	AIM	—	—	—
Shell	21122	XP_013060527	Uncharacterized protein	*Biomphalaria glabrata*	—	AIM	—	—	—
Shell	21466	XP_013085038	Uncharacterized protein	*Biomphalaria glabrata*	Polysacc_deac_1	AIM	—	—	**✓**
Shell	22322	CAD83837	Sialic acid binding lectin	*Cepaea hortensis*	C1Q	AIM	**✓**	—	—
Shell	22329	XP_011442512	Complement C1q-like	*Crassostrea gigas*	C1Q	AIM	—	—	—
Shell	22594	No hit	—	—	—	AIM/ASM	**✓**	—	—
Shell	22616	XP_012944826	Ubiquitin-60S ribosomal protein	*Aplysia californica*	UBQ, Ribosomal_L40e	AIM/ASM	—	—	—
Shell	22744	XP_022331321	Adipocyte plasma membrane-associated protein-like	*Crassostrea virginica*	—	AIM	—	—	—
Shell	22829	XP_013085779	Uncharacterixed protein	*Biomphalaria glabrata*	—	AIM/ASM	—	**✓**	**✓**
Shell	22899	No hit	—	—	—	AIM/ASM	—	**✓**	—
Shell	23501	XP_012940649	Uncharacterized protein	*Aplysia californica*	—	AIM	**✓**	—	—
Shell	24576	XP_013076008	Uncharacterized protein	*Biomphalaria glabrata*	—	ASM	—	—	—
Shell	25494	No hit	—	—		AIM/ASM	—	**✓**	—
Shell	25690	No hit	—	—	—	AIM	—	**✓**	—
Shell	26376	XP_013063306	Adipocyte plasma membrane-associated protein-like	*Biomphalaria glabrata*	—	AIM	—	—	—
Shell	26417	XP_009060461	Hypothetical protein	*Lottia gigantea*	—	AIM/ASM	—	—	—
Shell	31647	XP_013070671	Ipopolysaccharide-binding protein-like	*Biomphalaria glabrata*	—	AIM	—	—	—
Shell	53877	CCE72407	Histone H4	*Trigonopterus granum*	H4	AIM	—	—	—
Shell	04504^a^	ASK84891	Actin	*Gibbulinella dewinteri*	Actin	AIM	—	—	—
Shell	09811^a^	DE45339	Thioester-containing protein	*Biomphalaria glabrata*	A2M_comp, A2M_recep	AIM	—	—	—
Shell	12085^a^	XP_012944123	Extensin-2-like	*Aplysia californica*	—	AIM	—	—	**✓**
Shell	12964^a^	ABF00124	Sialic acid binding lectin	*Helix pomatia*	C1Q	AIM	—	—	—
Shell	15435^a^	XP_013064296	Uncharacterized protein	*Biomphalaria glabrata*	—	AIM/ASM	**✓**	—	**✓**
Shell	15522–15523^a^	XP_011413013	Sushi, von Willebrand factor type A, EGF, and pentraxin domain-containing protein	*Crassostrea gigas*	Sushi	AIM/ASM	**✓**	—	**✓**
Shell	16104^a^	XP_013070066	Alkaline phosphatase-like	*Biomphalaria glabrata*	Alk_phosphatases	AIM	—	—	—
Shell	16217–21060^a^	XP_013061848	Extensin-like	*Biomphalaria glabrata*	—	AIM/ASM	—	—	**✓**
Shell	21150^a^	XP_012946463	Mesenchyme-specific cell surface glycoprotein	*Aplysia californica*	—	AIM/ASM	—	—	—
Shell	21247^a^	XP_022313781	Sushi, von Willebrand factor type A, EGF, and pentraxin domain-containing protein	*Crassostrea virginica*	Sushi	AIM/ASM	**✓**	—	**✓**
Shell	21457^a^	No hit	—	*—*	—	AIM	—	**✓**	**✓**
Shell	21679^a^	XP_012943090	Uncharacterized protein	*Aplysia californica*	H-lectin	AIM	**✓**	—	—
Shell	23617–24364^a^	XP_013063539	Uncharacterized protein	*Biomphalaria glabrata*	Collagen_mid	AIM/ASM	—	**✓**	—
Shell	25307^a^	XP_013063306	Adipocyte plasma membrane-associated protein-like	*Biomphalaria glabrata*	Str_synth	AIM	—	—	—
Shell	39344^a^	XP_013063306	Adipocyte plasma membrane-associated protein-like	*Biomphalaria glabrata*	Str_synth	AIM	—	—	—
Shell	43690^a^	No hit	—	*—*	—	AIM	—	—	**✓**
Shell	44650–23959^a^	XP_013088302	Uncharacterized protein	*Biomphalaria glabrata*	—	ASM	—	—	—
Shell/Dart	9762	ABE99841	Beta-actin	*Crassostrea ariakensis*	Actin	AIM	—	—	—
Shell/Dart	21104^a^	No hit	—	*—*	—	AIM/ASM	—	—	**✓**
Dart	47492	No hit	—	*—*	—	AIM	—	—	—
Dart	50224	XP_013064798	Uncharacterized protein	*Biomphalaria glabrata*	CBM_14	AIM	—	—	—

Note.—AIS, acid insoluble matrix; ASM, acid soluble matrix; RLCDs, repetitive, low-complexity domains; SP, signal peptide; TM, transmembrane.

a Resequenced transcripts.

### Highly Abundant SMPs in *E. quaesita*

**Fig. 4.**
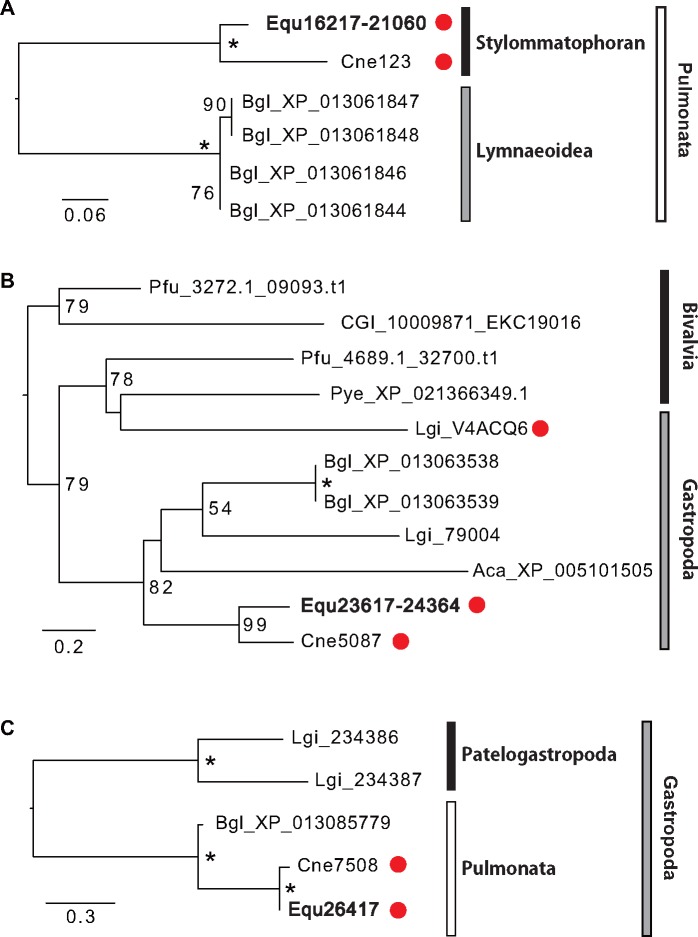
—Phylogeny of the most abundant SMPs identified from *Euhadra quaesita*. Three of the most abundant SMPs were identified by the abundance index values from LC-MS/MS analysis (fig. 3*B*). (*A*) The maximum likelihood tree was inferred from Equ16217–21060 and five genes that were found by BLASTX search against the GenBank database under the rtREV model (using 152 positions). (*B*) The maximum likelihood tree was inferred from Equ23617–24364 and ten genes that were found by BLASTX search against the GenBank database under the WAG  +  Γ model (using 332 positions). (*C*) The phylogenetic tree was inferred from Equ26417 and four genes that were found by BLASTX search against the GenBank database under the LG  +  Γ model (using 241 positions). All phylogenic analyses were performed with 100 bootstrap replicates, and bootstrap support values <50% are not shown. Asterisks indicate 100% bootstrap support. Branch lengths are proportional to the expected number of substitutions per site, as indicated by the scale bar. Red circles indicate proteins that have been identified as SMPs in this or previous studies. Aca, *Aplysia california*; Bgl, *Biomphalaria glabrata*; CGI, *Crassostrea gigas*; Cne, *Cepaea nemoralis*; Equ, *Euhadra quaesita*; Lgi, *Lottia gigantea*; Pye, *Patinopecten yessoensis*; Pfu, *Pinctada fucata*.

**Fig. 5.**
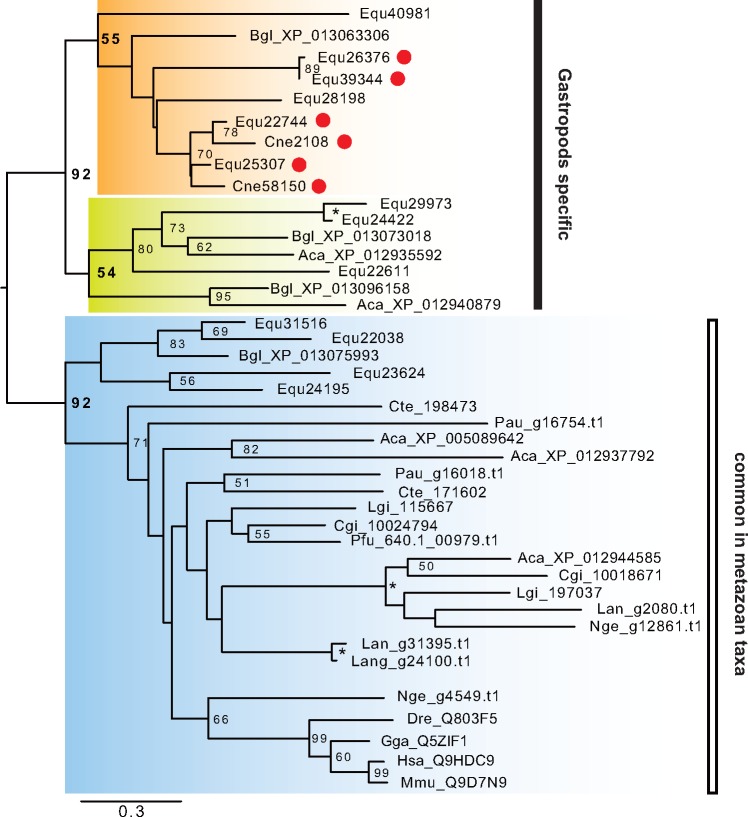
—Phylogeny of adipocyte plasma membrane-associated like proteins in various metazoan taxa. The maximum likelihood tree was inferred from 43 APMAP-like gene sequences under the LG  +  Γ + I model (352 positions of the strictosidine synthase domain, 100 bootstrap replicates). Bootstrap support values <50% are not shown. Asterisks indicate 100% bootstrap support. Branch lengths are proportional to the expected number of substitutions per site, as indicated by the scale bar. Red circles indicate proteins that have been identified as SMPs in this or previous studies. Aca, *Aplysia california*; Bgl, *Biomphalaria glabrata*; Cgi, *Crassostrea gigas*; Cne, *Cepaea nemoralis*; Cte, *Capitella teleta*; Dre, *Danio renio*; Equ, *Euhadra quaesita*; Gga, *Gallus gallus*; Hsa, *Homo sapiens*; Lan, *Lingula anatina*; Lgi, *Lottia gigantea*; Mmu, *Mus musculus*; Nge, *Notospermus geniculatus*; Pau, *Phoronis australis*; Pfu, *Pinctada fucata*.

We estimated the abundance of each protein in the shells using the number of identified MS/MS spectra (abundance index) and here focused on the ten highest abundant SMPs (fig. 3*C*). Most of the ten highest abundant SMPs have no homologous proteins (Equ22594, 14346, 25690, and 22899) or uncharacterized/hypothetical proteins (Equ23617–24364, 26417, and 22829) in the GenBank nonredundant protein database (table 1). The highest abundance index value of SMPs in *E. quaesita* is Equ16217–21060 (fig. 3*C*); it has no homologous proteins in the GenBank nonredundant protein database except for the proteins reported from the pulmonates *B. glabrata* (XP_013061844, XP_013061846, XP_013061847, and XP_013061848; table 1) and *C. nemoralis* (Cne123, Mann and Jackson 2014). These proteins contain Pro- and Gly-rich regions, but have no known specific domains. Interestingly, Cne123 is the most abundant SMP in *C. nemoralis* too (Mann and Jackson 2014). Thus, these novel proteins may have evolved in the last common ancestor of pulmonates with a key role in their shell mineralization under terrestrial or fresh water environments (fig. 4*A*). The second most abundant SMP of *E. quaesita* (Equ23617–24364) shows significant similarities with SMPs of *C. nemoralis* (Cne5087) and *L. gigantea* (LgiV4ACQ6) (fig. 3*C*). This protein has a collagen-related domain (Collagen middle region) and is likely to have evolved in the lineage leading to molluscs because there are no proteins with significant similarity in public databases, except for molluscan proteins (fig. 4*B*). The third most abundant SMP of *E. quaesita* (Equ26417, fig. 3*C*) shares significant similarity with the SMP of *C. nemoralis* (Cne7508) that is the third most abundant SMP in that species. This protein has no significant similarity with any proteins in the public databases except for proteins reported from a limpet *L. gigantea* (Lgi_234386 and 234387) and a pond snail *B. glabrata* (XP_013085779) (fig. 4*C*). Equ26417 and Cne7508 are likely to have evolved in the gastropods and play a role in shell formation in pulmonates, because these proteins were not found as SMPs in the limpet *L. gigantea* (Mann et al. 2012; Marie et al. 2013). The sixth most abundant SMP of *E. quaesita* (Equ22744, fig. 3*C*) shares significant similarity with the proteins in the public databases that are named adipocyte plasma membrane-associated like proteins (APMAP-like) by a BLASTX search (table 1). Furthermore, we found other three APMAP-like proteins (Equ26376, 39344, and 25307) as SMPs in *E. quaesita* (table 1). These proteins have strictosidine synthase domains (Str_synth) that are contained in one of the key enzymes in alkaloid biosynthesis in plants (Bracher and Kutchan 1992). They are also homologous proteins to the SMPs of *C. nemoralis* (Cne2108 and 58510) and have diversified in gastropods, and some of them likely correlate with shell formation only in pulmonates (fig. 5). Last, the tenth most abundant SMP of *E. quaesita* (Equ15222–15223, fig. 3*C*) and Equ21247 share significant similarity with the proteins in the public databases that are named sushi, von Willebrand factor type A, EGF and pentraxin domain-containing protein-like (SVEP1-like) by a BLASTX search (table 1). SVEP is a common SMP in Mollusca (Marie et al. 2010, 2011, 2012; Mann et al. 2012; Zhang et al. 2012; Gao et al. 2015; Arivalagan et al. 2016) and has VWA (von Willebrand factor type A), EGF domain, and CCP (complement control protein) modules. However, it is difficult to annotate these proteins (Equ15222–15223 and Equ21247) as SVEP1, because they have a Sushi domain but lack other three domains (VWA, EGF, and CCP). Thus, we just described them as Sushi-containing proteins in this study. On the other hand, we found VWA domain containing protein in SMP Equ14133 (table 1). VWA domain-containing proteins may interact with calcium ions during calcification, because it has calcium-binding structure. In other molluscan SMPs, most VWA domain-containing proteins have other specific domains (e.g., CCP or chitin binding domain), but we could not find other specific domains from Equ14133. Thus, we just described this matrix protein as VWA-containing protein in this study.

### Domain-Containing SMPs

**Fig. 6.**
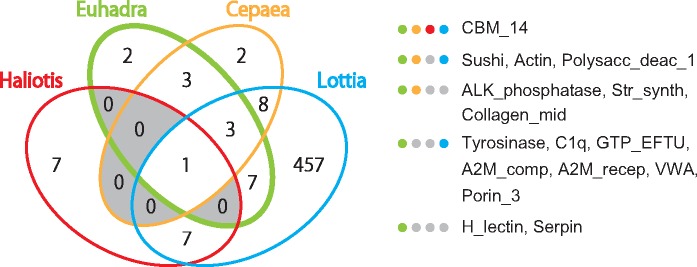
—Comparisons of domains of SMPs among four gastropods *Euhadra quaesita*, *Cepaea nemoralis*, *Haliotis asinina*, and *Lottia gigantea.* Carbonic anhydrase binding motif 14 (CBM_14) is a major domain in SMPs of the four gastropods. Three domains are conserved within three species (except for *H. asinina*), and four domains are conserved within pulmonate (*E. quaesita and C. nemoralis*). ALK_phosphatase, alkaline phosphatase; A2M_com, a-macroglobulin compliment component; A2M_recep, a-macroglobulin receptor; Collagen_mid, bacterial collagen middle region; C1q, compliment component 1q; GTP_EFTU, elongation factor Tu GTP binding domain; H_lectin, H-type lectin domain; Polysac_deac_1, polysaccharide deacetylase; Porin_3, eukaryotic porin; Str_syn, strictosidine synthase; Sushi, sushi repeat domain; VWA, von Willebrand factor type A domain.

We searched the domains from the matrix proteins by the online version of SMART (Letunic et al. 2015; Letunic and Bork 2018; http://smart.embl-heidelberg.de; last accessed January 25, 2019) and found 18 domains from 24 matrix proteins (table 1). We then compared them with the domains that were found in 12 previously studied molluscan species (grove snail, *Cepaea nemoralis*; abalone, *H. asinina;* limpet, *L. gigantea*; oyster, *Crassostrea gigas*; pearl oyster, *P. margaritifera* and *P. maxima*; mussel, *Mytilus galloprovincialis* and *M. edulis*; clam, *Mya truncata*; king scallop, *P. maximus*; fresh water mussels, *Elliptio complanata* and *V. lienosa*) (Marie et al. 2011, 2013, 2017; Mann et al. 2012; Zhang et al. 2012; Mann and Jackson 2014; Gao et al. 2015; Arivalagan et al. 2016, 2017) (supplementary table S7, Supplementary Material online). We showed the results of the domain comparisons among four gastropods *E. quaesita*, *C. nemoralis*, *H. asinina*, and *L. gigantea* in figure 6. We found that carbonic anhydrase binding motif 14 (CBM_14) is the only specific domain found in SMPs for all 12 molluscs (fig. 6 and supplementary table S7, Supplementary Material online) and is contained in SMP (Equ10634) and DMP (Equ50224) of *E. quaesita*. CBM_14 is found in the chitin-binding proteins in other animals. Furthermore, polysaccharide deacetylase 1 (polysacc_deac_1) domain-containing proteins were found from the SMPs in *E. quaesita* (Equ21466, table 1) and other gastropods (*C. nemoralis* and *L. gigantea*) (fig. 6 and supplementary table S7, Supplementary Material online). Polysaccharides especially chitin are well known as major components of the mineralized structures in the metazoan and is likely to involve in constructing the frameworks of the mollusc shell (Brunet and Carlisle 1958; Peters 1972; Falini and Fermani 2004; Weiss et al. 2006; Ehrlich et al. 2013). Thus, the polysaccharides or chitin-related proteins that contain CBM_14 domain or polysacc_deac_1 domain might play prominent roles for shell and/or dart formation.

Tyrosinase is one of the highly conserved domains in mollusc SMPs (supplementary table S7, Supplementary Material online). In this study, we found six *tyrosinase-like* transcripts (Equ11340, Equ11343, Equ14143, Equ24617, Equ32293, and Equ33769) from the transcriptome data, and only Equ11340 was found in the shell proteome of *E. quaesita* (table 1, supplementary table S3 and fig. S8, Supplementary Material online). Tyrosinase is known to be related to melanin biosynthesis in animals, and has already been reported as an SMP in molluscs (fig. 6 and supplementary fig. S8, Supplementary Material online, Nagai et al. 2007; Zhang et al. 2012; Liao et al. 2015). Tyrosinase is found in the black fibrous prism layer in the mussel *Mytilus coruscus* (Liao et al. 2015). *Euhadraquaesita* has a single black color band in its shell, not in the periostracum. Thus, the tyrosinase in the shell might be related to shell pigmentation in *E. quaesita*. On the other hand, tyrosinase has not been identified as an SMP in the abalone *H. asinina* (Marie et al. 2010) nor in the grove snail *C. nemoralis*, which has various stripe patterns in the shell (Mann and Jackson 2014). Thus, some shell pigments might be produced by the tyrosinase-related melanin biosynthetic pathway, while other shell pigments in some pulmonate snails could be produced by different mechanisms. The tyrosinase gene family has expanded in molluscs (Aguilera et al. 2014), and some of them are likely to have evolved independently as SMPs in several species (supplementary fig. S8, Supplementary Material online). Thus, those SMPs containing the same domain may have a similar function, but it is possible that they have evolved independently by domain shuffling, domain recruitment, or gene co-option in each group (Kocot 2016).

**Fig. 7.**
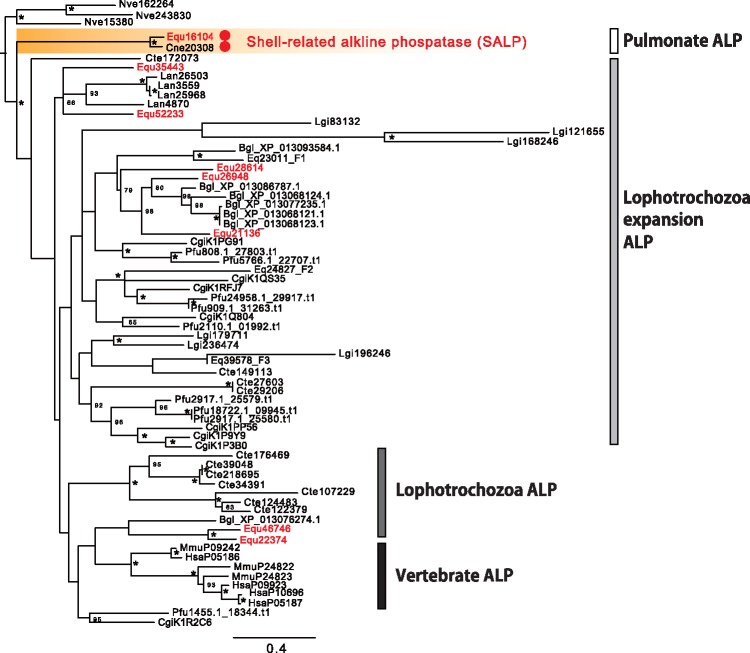
—Phylogeny of alkaline phosphatase (ALP) in various metazoan taxa. The maximum likelihood tree was inferred from 67 alkaline phosphatase gene sequences under the WAG + Γ model (250 positions of the ALK_phosphatase domain, 100 bootstrap replicates). Bootstrap support values <50% are not shown. Asterisks indicate 100% bootstrap support. Branch lengths are proportional to the expected number of substitutions per site, as indicated by the scale bar. Red circles indicate proteins that have been identified as SMPs in this or previous studies. Aca, *Aplysia california*; Bgl, *Biomphalaria. glabrata*; Cgi, *Crassostrea gigas*; Cne, *Cepaea nemoralis*; Cte, *Capitella teleta*; Equ, *Euhadra quaesita*; Hsa, *Homo sapiens*; Lan, *Lingula anatina*; Lgi, *Lottia gigantea*; Mmu, *Mus musculus*; Nve, *Nematostella vectensis*; Pfu, *Pinctada fucata*.

We found alkaline phosphatase domain-containing protein (Equ16104) from the SMPs in *E. quaesita*. In vertebrates, alkaline phosphatase (ALP) is well known as biomineralization-related enzyme (Henthorn and Whyte 1992). In this study, we found eight alkaline phosphatase (ALP) transcripts from the transcriptome data (Equ16104, Equ21136, Equ22374, Equ26948, Equ28614, Equ35443, Equ46746, and Equ52233), and only Equ16104 was found as an SMP from *E. quaesita* (table 1 and supplementary table S3, Supplementary Material online). ALP is expressed during the early development of bone and calcified cartilage tissues, and tissue-nonspecific ALP (TNAP) is an especially the important promoter of bone mineralization (Henthorn and Whyte 1992; Hessle et al. 2002; Harmey et al. 2004). In molluscs, ALP has already been reported as a SMP in *C. nemoralis* (fig. 6, Mann and Jackson 2014). In addition, Hohagen and Jackson (2013) have reported that alkaline phosphatase activity is observed in the shell forming cells that are located in the shell gland and the shell field during their differentiation stage in the pond snail *Lymnaea stagnalis*, suggesting that ALP is involved in the initial shell formation. Although molluscs have evolved many ALPs (fig. 7), ALPs have not been reported as SMPs in molluscs except in the grove snail *C. nemoralis*. Molecular phylogenetic analyses have indicated that the shell-related ALPs (SALPs) form a clade separated from other lophotrochozoan ALPs (fig. 7; abalone, *H. asinina*, Marie et al. 2010; limpet, *L. gigantea*, Mann et al. 2012; Marie et al. 2013; oyster, *Crassostrea gigas*, Zhang et al. 2012; pearl oyster, *P. margaritifera* and *P. maxima*, Marie et al. 2012; mussel, *Mytilus galloprovincialis*, Gao et al. 2015 and *M. edulis*, Marie et al. 2011; clam, *M. truncata*, Arivalagan et al. 2016; king scallop, *P. maximus*, Arivalagan et al. 2017). Thus, SALPs appear to have evolved only in the pulmonates (fig. 7). We did not detect *salp* gene expression in the dart sac tissue, and therefore SALP is unlikely to play role in dart sac formation.

C1q domain is a common domain in molluscan SMPs (fig. 6 and supplementary table S7, Supplementary Material online; Mann et al. 2012; Zhang et al. 2012; Marie et al. 2013; Gao et al. 2015; Liao et al. 2015) and is contained in the acid-insoluble organic matrix of *E. quaesita* (Equ12964 and 22322, table 1). C1q proteins correlate with the immunity pathway in the scallop *Chlamys farreri*, (Zhang et al. 2008; Gerdol et al. 2011) and play important roles in the innate immune response in invertebrates (Zhang et al. 2004; Carland and Gerwick 2010). However, C1q proteins are diverse in lophotrochozoans (supplementary fig. S9, Supplementary Material online), and their function in shell formation is unclear.

Several kinds of protease inhibitors (e.g., Kunitz-like, WAP, macroglobulin) have already been found as SMPs in molluscs (Marie et al. 2011, 2017; Zhang et al. 2012; Arivalagan et al. 2017). Protease inhibitors could be involved in the protection of SMPs against several kinds of protease or in the regulation of an immune response pathway (Arivalagan et al. 2016). In *E. quaesita*, we found serpin-like protein (Equ10941), which is a member of the serpin family and protease inhibitor, as a SMP by BLAST analysis. Serpin-like protein plays a major role in the inhibition of serine protease, which regulates proteolytic activities, by binding to the serine activation site. Serpin-like protein was found as a SMP in *Mytilus galloprovincialis* (Gao et al. 2015; supplementary table S7, Supplementary Material online). Furthermore, we found other domains related to another protease inhibitor, (Alpha-2-macroglobulin); Alpha-2-macroglobulin compliment component (A2M_comp) and Alpha-2-macroglobulin receptor (A2M_recep), in Equ09811 (table 1). A2M domain-containing proteins were found in other four molluscan SMPs (supplementary table S7, Supplementary Material online) and the byssus protein of *Mytilus coruscus* (Qin et al. 2016). Thus, these protease inhibitor-related proteins have most likely evolved as an SMP independently in molluscs.

### Other SMPs

We found the mesenchyme-specific cell surface glycoprotein-like protein (MSP130) as a SMP (Equ21250, supplementary fig. S10, Supplementary Material online). *Msp130* is expressed in primary mesenchyme cells and has been extracted from hard tissues of sea urchins (Anstrom et al. 1987; Leaf et al. 1987; Mann et al. 2008, 2010). Thus, Msp130 is possibly associated with biomineralization, because Msp130 has been found as a skeletal matrix protein not only in sea urchins (Mann et al. 2008, 2010) but also in molluscs (Mann et al. 2012; Zhang et al. 2012; Mann and Jackson 2014) and brachiopods (Isowa et al. 2015; Jackson et al. 2015). Although *msp130* has been reported in many phyla (Szabó and Ferrier 2015), its function remains unclear.

In previous studies, proteins containing repetitive, low-complexity domains (RLCDs) have been reported as SMPs in other molluscs (Jackson et al. 2010; Marie et al. 2011; Werner et al. 2013). For instance, some repeated acidic motifs could bind calcium ions and play important roles in the biomineralization processes (e.g., Aspein; Isowa et al. 2012). RLCD containing proteins have already been reported not only from molluscan shells but also from skeletons of other invertebrates (Wustman et al. 2002; Livingstone et al. 2006; Isowa et al. 2015; Jackson et al. 2015; Luo et al. 2015). In this study, we found 20 SMPs that contain RLCDs (supplementary table S8, Supplementary Material online) by the SMART program. Seven of the 13 SMPs that showed no similar sequences in protein database searches by BLASTP have RLCDs. This result suggests that these RLCD containing proteins were technically difficult to align with other proteins and/or they have rapidly evolved at the primary sequence level (Kocot 2016).

### Dart Matrix Proteins

We found four DMPs in *E. quaesita* against 54 SMPs (table 1). Interestingly, two of them are also SMPs (Equ09762 and Equ21104). Equ09762 is a beta actin-like protein and is an abundant cytoskeletal protein. Thus, it appears likely to be taken up inside the shell and dart accidentally, and unlikely to be directly involved in biomineralization. Equ21104 does not have homologous proteins with the GenBank nonredundant protein database except for the *Cepaea nemoralis* SMP, Cne2744 (Mann and Jackson 2014) (table 1 and supplementary table S8, Supplementary Material online). Like many SMPs, Equ21104 contains four RLCDs with relatively high aspartic acid composition (supplementary table S8, Supplementary Material online), these regions possibly bind calcium ions and play a role in shell calcification (Kalmar et al. 2012). Although it is technically difficult to find homologous proteins for RLCD containing proteins, Equ21104 and Cne2744 may have evolved in the lineage leading to the Stylommatophora as a SMP, and was possibly then recruited for dart formation, at least in *E. quaesita*. Equ50224 is the third most abundant DMP, and has a CBM14 domain. The last DMP, Equ47492, does not have characteristic domains, RLCDs, or homologous proteins in the GenBank nonredundant protein database.

### Evolution of Shell and DMPs

**Fig. 8.**
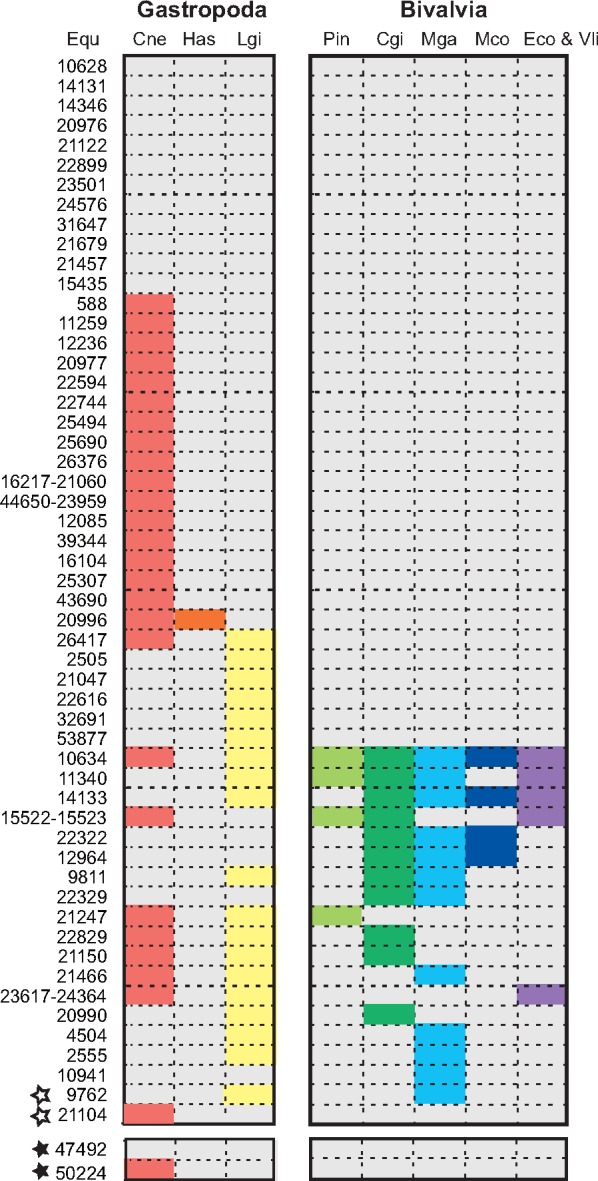
—Comparison of pulmonate SMPs and DMPs with other molluscan SMPs. Some SMPs and DMPs in *Euhadra quaesita* are homologous to other molluscan SMPs (BLASTP comparison, cut-off e-value>1.0e-5, details shown in supplementary table S8, Supplementary Material online), and these SMPs and DMPs are shown in colors. Open stars indicate DMPs that were identified also as SMPs. Black stars indicate DMPs that were identified only from the dart. Cgi, *Crassostrea gigas*; Cne, *Cepaea nemoralis*; Eco, *Elliptio complanata*; Equ, *E. quaesita*; Has, *Haliotis asinina*; Lgi, *Lottia gigantea*; Mco, *Mytilus coruscus*; Mga, *M. galloprovincialis*; Pin, *Pinctada*; Vli, *Villosa lienosa*.

We compared the SMPs and DMPs of *E. quaesita* with other SMPs in molluscan species. We found that 22.2% of SMPs in *E. quaesita* (12/54) do not share significant similarity with the other molluscan SMPs, and 29.6% of SMPs (16/54) share significant similarity only with the SMPs of *Cepaea nemoralis* (fig. 8 and supplementary table S9, Supplementary Material online). These results are consistent with the hypothesis of a rapidly evolving shell-forming secretome in molluscs (Jackson et al. 2006; Kocot 2016). On the other hand, we found that some SMPs of *E. quaesita* have domains often found in SMPs of other gastropods and bivalves (CBM_14, VWA, Tyrosinase, C1q, and Sushi) (table 1 and supplementary table S7, Supplementary Material online). Thus, some of these SMPs have possibly evolved independently by domain shuffling, domain recruitment, or gene co-option (Kocot 2016), and these common domains might play key roles in molluscan shell mineralization.

The oldest fossils of the Stylommatophora (a clade containing the majority of terrestrial snails and slugs, fig. 1*A*) are found from the Paleozoic and upper Carboniferous (∼300 Ma, Solem and Yochelson 1979), while snails belonging to the Lymnaeoidea (including the pond snail *B. glabrata*) first appeared during the Jurassic (Tracey et al. 1993). The Stylommatophora probably represent the first pulmonates, and the upper Carboniferous age (∼300 Ma) is supported not only by the fossil record but also by molecular phylogenetic analysis using mitochondrial genomes (Grande et al. 2008). The age of divergence of stylommatophorans including the Helicidae (e.g., *Cepaea nemoralis*) and Bradybaenidae (e.g., *E. quaesita*), has been estimated as the late Cretaceous (∼73.16 Ma) by molecular phylogenetic analysis (Razkin et al. 2015). We found some domains were only found in the SMPs of gastropods or pulmonates (gastropod specific: Polysacc_deac, pulmonate specific: ALK_phosphatase, Str_synth, and Collagen_mid) (fig. 6 and supplementary table S7, Supplementary Material online). These results indicate that the last common ancestor of pulmonates (or stylommatophorans) evolved specific SMPs including both novel proteins and already existing proteins that did not play roles in shell development before (e.g., alkaline phosphatase and adipocyte plasma membrane-associated proteins, figs. 4, 5, and 7). In fact, we found that the most abundant SMPs common to both *E. quaesita* and *Cepaea nemoralis* are novel proteins in pulmonates (fig. 4*A*). Our results suggest that some of these proteins are likely to play key roles in pulmonate shell formation in terrestrial or fresh water environments.

Dart shooting is one of the most peculiar reproductive behaviors that have evolved in stylommatophorans. Darts have evolved various shapes and sizes, and are used in different ways during mating (Koene and Schulenburg 2005). However, the molecular mechanisms and matrix proteins of dart formation remain unclear. In this study, we found three similar features both in the matrix proteins and transcripts. First, we found a much smaller number of DMPs in comparison with SMPs, and two of the four DMPs (Equ09762 and Equ21104) are the same as proteins identified from the SMPs of *E. quaesita* and other species (*Cepaea nemoralis*). In addition, some shell-related genes like dermatopontin are expressed in the dart sac tissue (supplementary tables S3 and S4, Supplementary Material online), although they were not detected in the dart proteome. Second, three of four DMPs have significant similarity with SMPs of other molluscs and have similar domain (CBM_14) or repeat structures (RLCDs) (table 1). These similarities were also observed in the byssus proteins of *M. coruscus* (e.g., VWA and Tyrosinase) (Qin et al. 2016). Last, we also found that some genes of the key signaling pathways (e.g., BMP, Wnt, and Notch) are shared between the mantle and the dart sac (supplementary tables S3 and S4, Supplementary Material online). These results suggest that some DMPs and dart-related proteins possibly have evolved by gene co-option from SMPs and/or the gene regulatory network (GRN) of shell development during evolution of the love dart in stylommatophorans.

## Conclusions

We found a total of 54 SMPs and four DMPs in the terrestrial snail *E. quaesita*, and two of them are the same proteins as those identified from the shell matrix. Most SMPs are novel proteins in this species, or do not show significant similarities with proteins of any other animals except for the grove snail *Cepaea nemoralis*. However, we found the widely conserved domains (CBM_14, VWA, Tyrosinase, Sushi, and C1Q) in the SMPs of *E. quaesita*. Some of these SMPs have possibly evolved independently by domain shuffling, domain recruitment, or gene co-option. In addition, we identified CBM_14 domain-containing protein from the dart proteome, and its homologous protein was found among the *Cepaea nemoralis* SMPs. This is the first report of the dart proteome, and our results suggest that some DMPs and developmental genes related to dart formation have possibly evolved by independent gene co-option from SMPs and GRNs over several dart evolutionary events in stylommatophorans. This provides a new perspective on “love dart” evolution.

## Supplementary Material

Supplementary data are available at *Genome Biology and Evolution* online.

## Supplementary Material

Supplementary Data

## Footnotes

**Data deposition:** This project has been deposited at the DNA Data Bank of Japan (DDBJ) under the accession numbers (The shot-gun sequences; DRA006965-DRA006966, assembled sequences; PRJDB6927: IADG01000001-IADG01059618, the protein sequences; LC375283-LC375300).
