# Supplementary material for: Insights into the Evolution of Shells and Love Darts of Land Snails Revealed from Their Matrix Proteins
Source: Genome Biol Evol. 2018 Nov 2;11(2):380–97. doi: 10.1093/gbe/evy242 (PMC6368272; doi:10.1093/gbe/evy242)
Supplement: Supplementary Data [file evy242_supp.zip › Supplymentary_181024.pdf]

Supplementary figures S1-S10 and files S1-S3

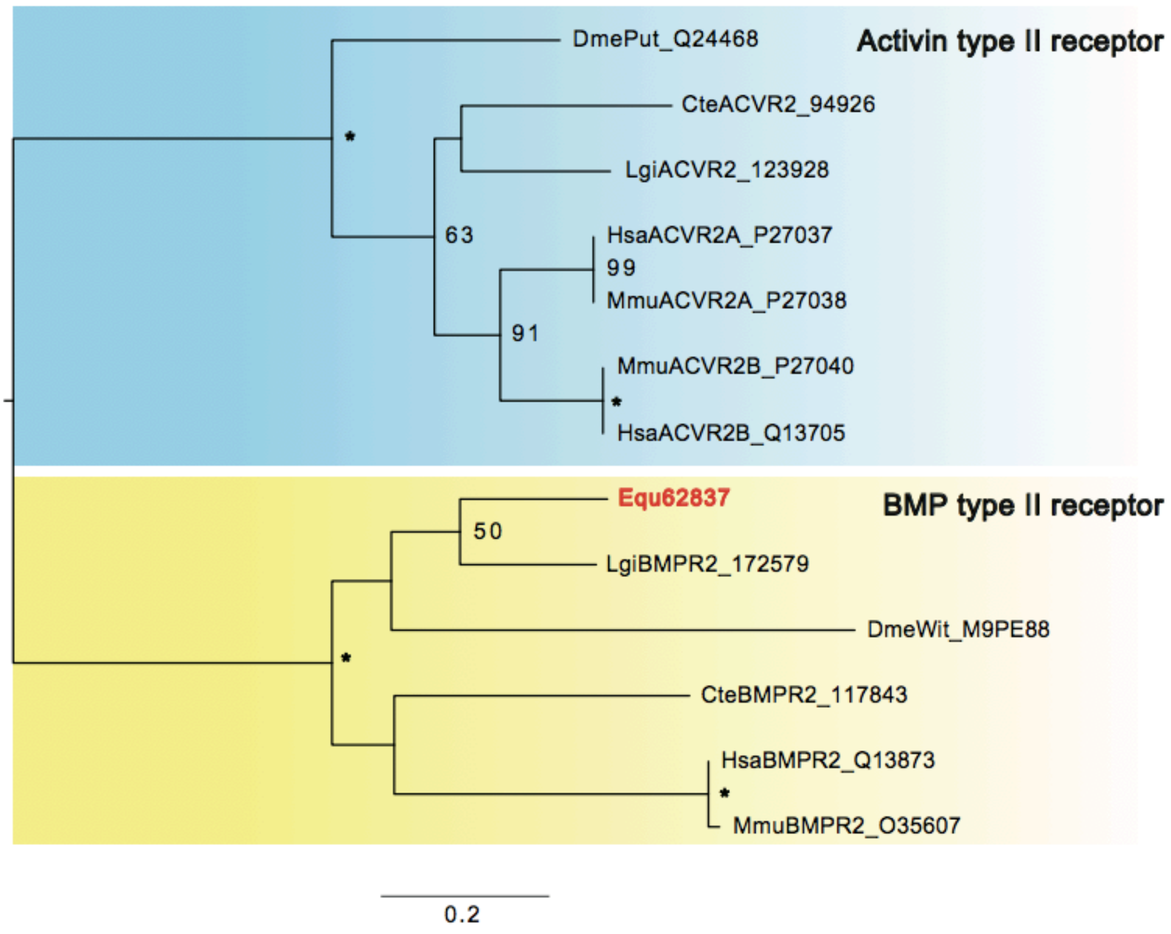

Figure S1. Phylogeny of BMPR in various metazoan taxa. The maximum likelihood tree was inferred from 13 BMPR sequences under the LG + Γ model (284 positions of the protein kinase domain, 100 bootstrap replicates). Asterisks indicate 100% bootstrap support. Bootstrap support values below 50% are not shown. Branch lengths are proportional to the expected number of substitutions per site, as indicated by the scale bar. OTU names in red indicate the sequence found from *Euhadra quaesita*. Cte: *Capitella teleta*, Dme: *Drosophila melanogaster*, Equ: *Euhadra quaesita*, Hsa: *Homo sapiens*, Lgi: *Lottia gigantea*, Mmu: *Mus musculus*.

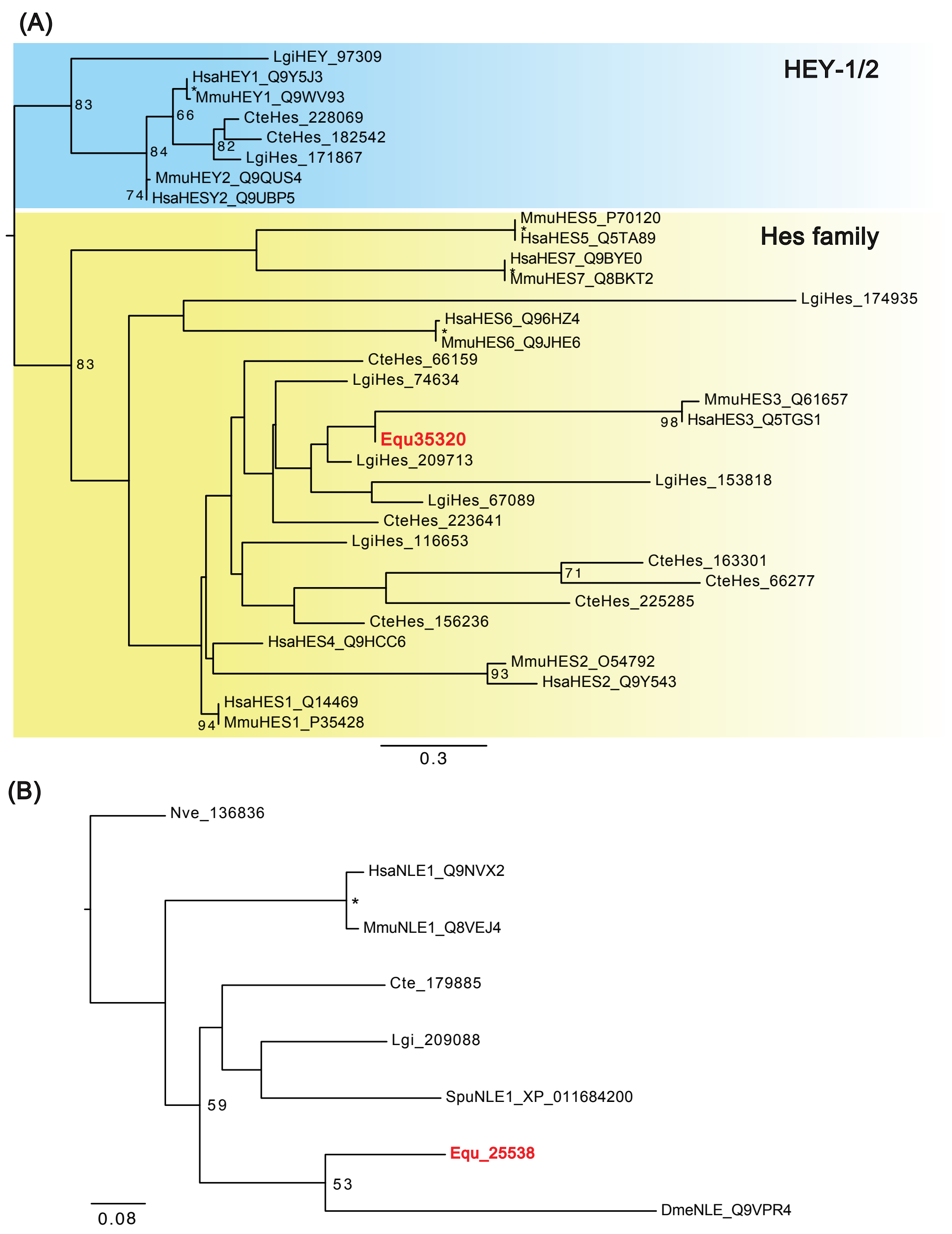

Figure S2. Phylogeny of Hes and Notchless in various metazoan taxa. (A) The maximum likelihood tree was inferred from 34 Hes sequences under the LG + Γ model (92 positions of the HLH and Hes_orange domains, 100 bootstrap replicates). **(B)** The maximum likelihood tree was inferred from 8 Notchless sequences under the LG + Γ model (475 positions of the NLE domain, 100 bootstrap replicates). Asterisks indicate 100% bootstrap support. Bootstrap support values below 50% are not shown. Branch lengths are proportional to the expected number of substitutions per site, as indicated by the scale bar. OTU names in red indicate the sequence found from *Euhadra quaesita*. Cgi: *Crassostrea gigas*, Cte: *Capitella teleta*, Equ: *Euhadra quaesita*, Gga: *Gallus gallus*, Hsa: *Homo sapiens*, Lgi: *Lottia gigantea*, Mmu: *Mus musculus*.

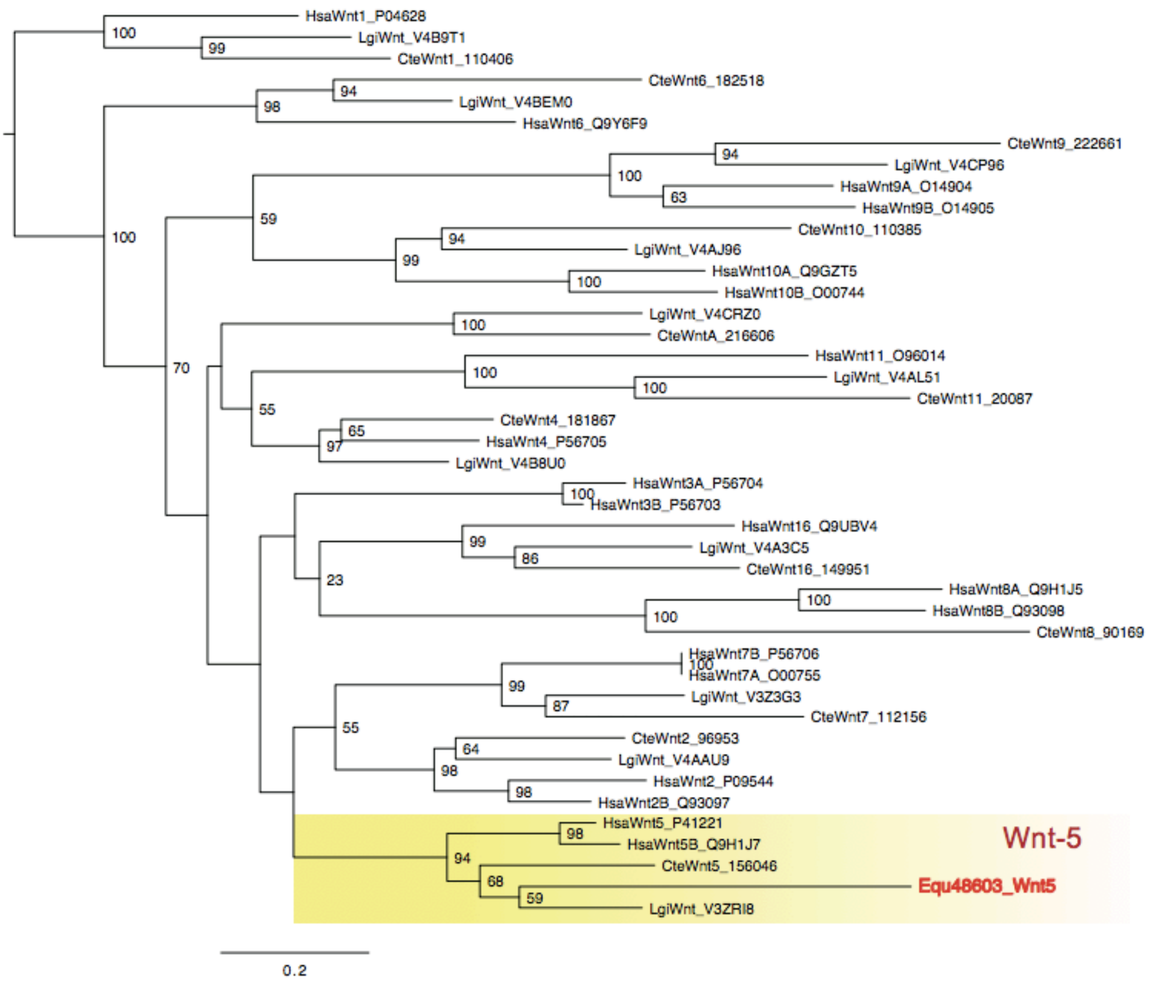

Figure S3. Phylogeny of Wnt in various metazoan taxa. The maximum likelihood tree was inferred from 43 Wnt sequences under the WAG + Γ model (295 positions of the wnt domain, 100 bootstrap replicates). Bootstrap support values below 50% are not shown. Branch lengths are proportional to the expected number of substitutions per site, as indicated by the scale bar. OTU names in red indicate the sequence found from *Euhadra quaesita*. Cte: *Capitella teleta*, Equ: *Euhadra quaesita*, Hsa: *Homo sapiens*, Lgi: *Lottia gigantea*.

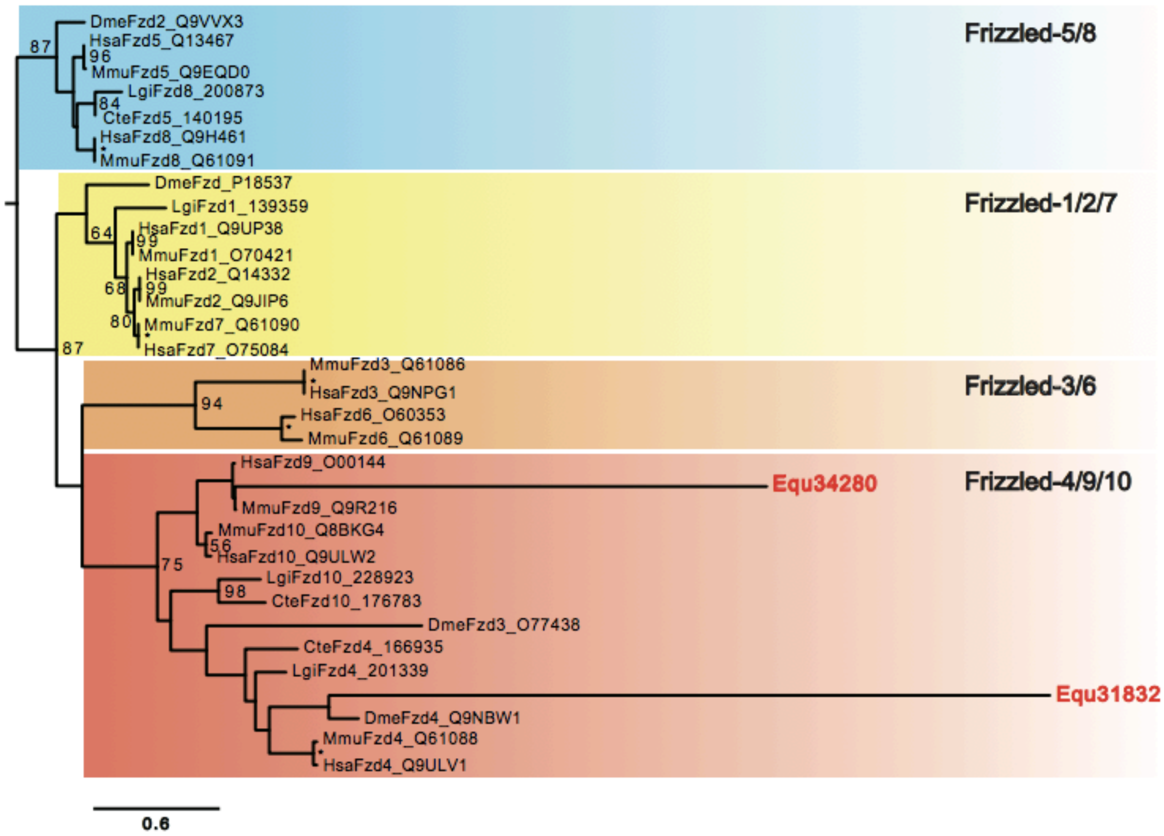

Figure S4. Phylogeny of Frizzled in various metazoan taxa. The maximum likelihood tree was inferred from 33 Frizzled sequences under the LG + Γ model (108 positions of the Fz domain, 100 bootstrap replicates). Bootstrap support values below 50% are not shown. Branch lengths are proportional to the expected number of substitutions per site, as indicated by the scale bar. OTU names in red indicate the sequence found from *Euhadra quaesita*. Cgi: *Crassostrea gigas*, Cte: *Capitella teleta*, Dme: *Drosophila melanogaster*, Equ: *Euhadra quaesita*, Gga: *Gallus gallus*, Hsa: *Homo sapiens*, Lgi: *Lottia gigantea*, Mmu: *Mus musculus*.

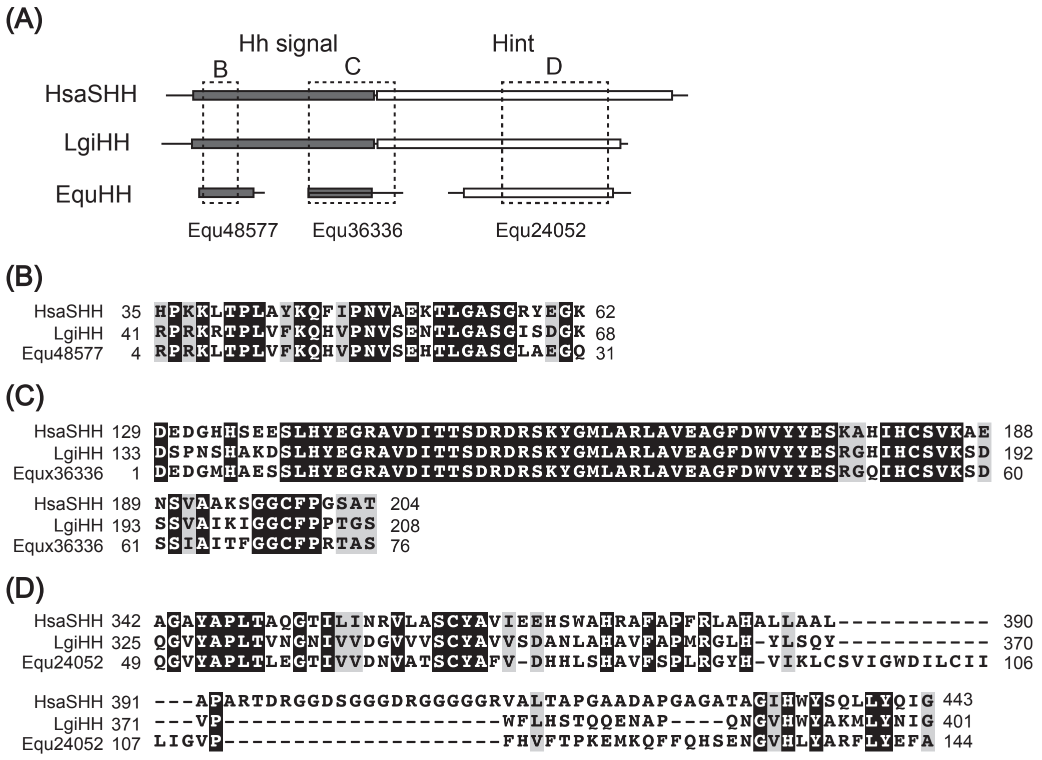

Figure S5. Hh-like protein structures in *Euhadra quaesita*. (A) Domain structures of three Hh-like proteins identified in this study (Equ48577, Equ36336, and Equ24052). Typical Hh protein has Hedgehog signal domain (grey boxes) and Hint domain (white boxes) like HsaSHH and LgiHH. However Equ48577 and Equ36336 have only Hedgehog signal domain. On the other hand, Equ24052 have only Hint domain. Dash lines indicated the alignment sites of Equ48577 **(B)**, Equ36336 **(C)**, and Equ24052 **(D)**. **(B-D)** Alignment sequences of Hh-like proteins in *Euhadra quaesita*. Conserved residues are shaded black (100%) and gray (>70%). Numbers indicate the amino acid positions of each protein. These three Hh-like transcripts (Equ48577, Equ36336, and Equ24052) matched different sites of other Hh proteins (HsaSHH and LgiHH), suggesting that a true Hh-like transcript is split in three contigs (Equ48577, Equ36336, and Equ24052) by misassembling. Equ: *Euhadra quaesita*, Hsa: *Homo sapiens*, Lgi: *Lottia gigantea*.

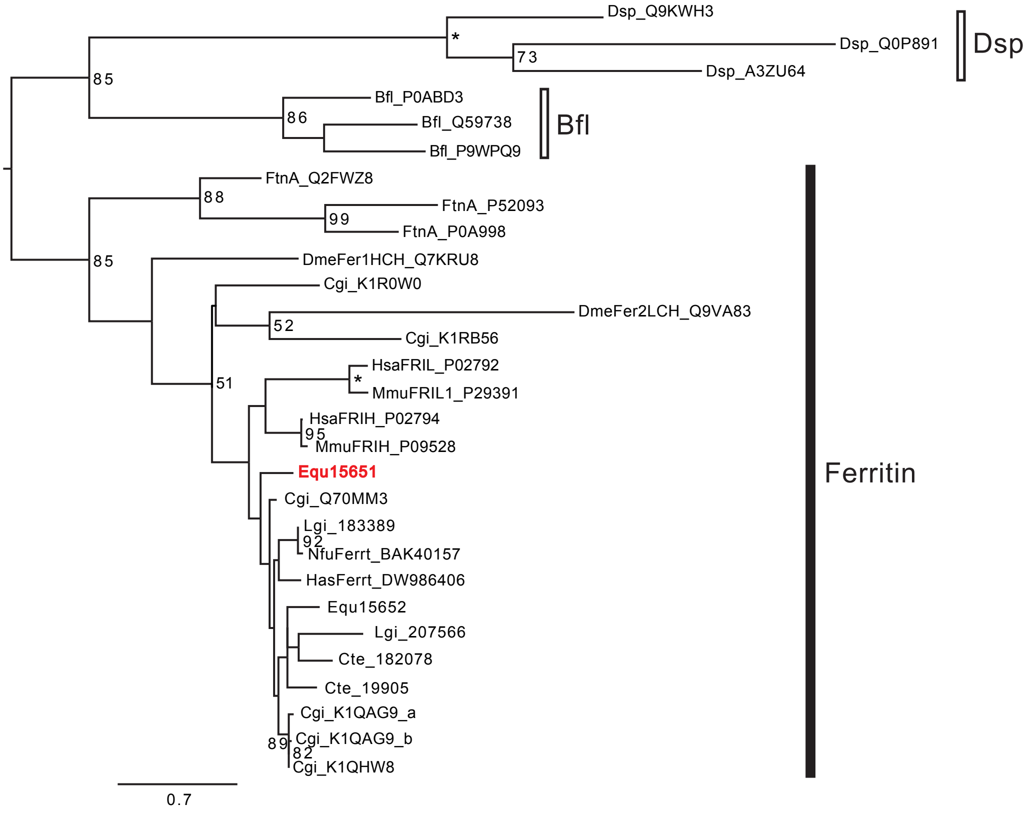

Figure S6. Phylogeny of Ferritin in various taxa. The maximum likelihood tree was inferred from 13 Ferritin-like sequences under the LG + Γ model (138 positions of the Ferritin domain, 100 bootstrap replicates). Bootstrap support values below 50% are not shown. Branch lengths are proportional to the expected number of substitutions per site, as indicated by the scale bar. OTU names in red indicate the sequence found from *Euhadra quaesita*. Bfr: Bacterioferritin, Cgi: *Crassostrea gigas*, Cte: *Capitella teleta*, Dme: *Drosophila melanogaster*, Dps: DNA protection during starvation protein, Equ: *Euhadra quaesita*, FtnA: Bacterial non-heme ferritin, Has: *Haliotis asinina*, Hsa: *Homo sapiens*, Lgi: *Lottia gigantea*, Mmu: *Mus musculus*, Nfu: *Nipponacmea fuscoviridis*.

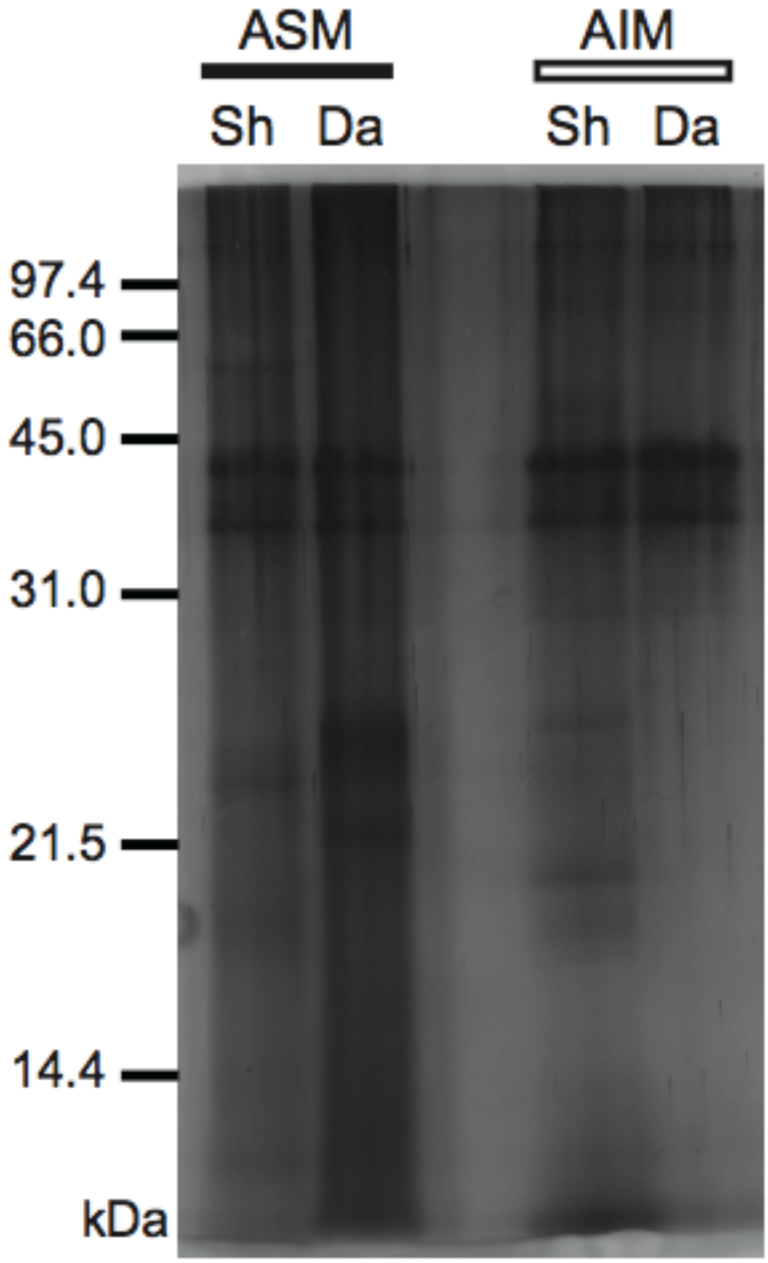

Figure S7. Silver staining of the matrix proteins in the shell and dart of *E. quaesita*. Silver staining of the acid-soluble matrix (ASM) and acid-insoluble matrix (AIM) extracted from the shell and the dart. Sh: shell, Da: dart.

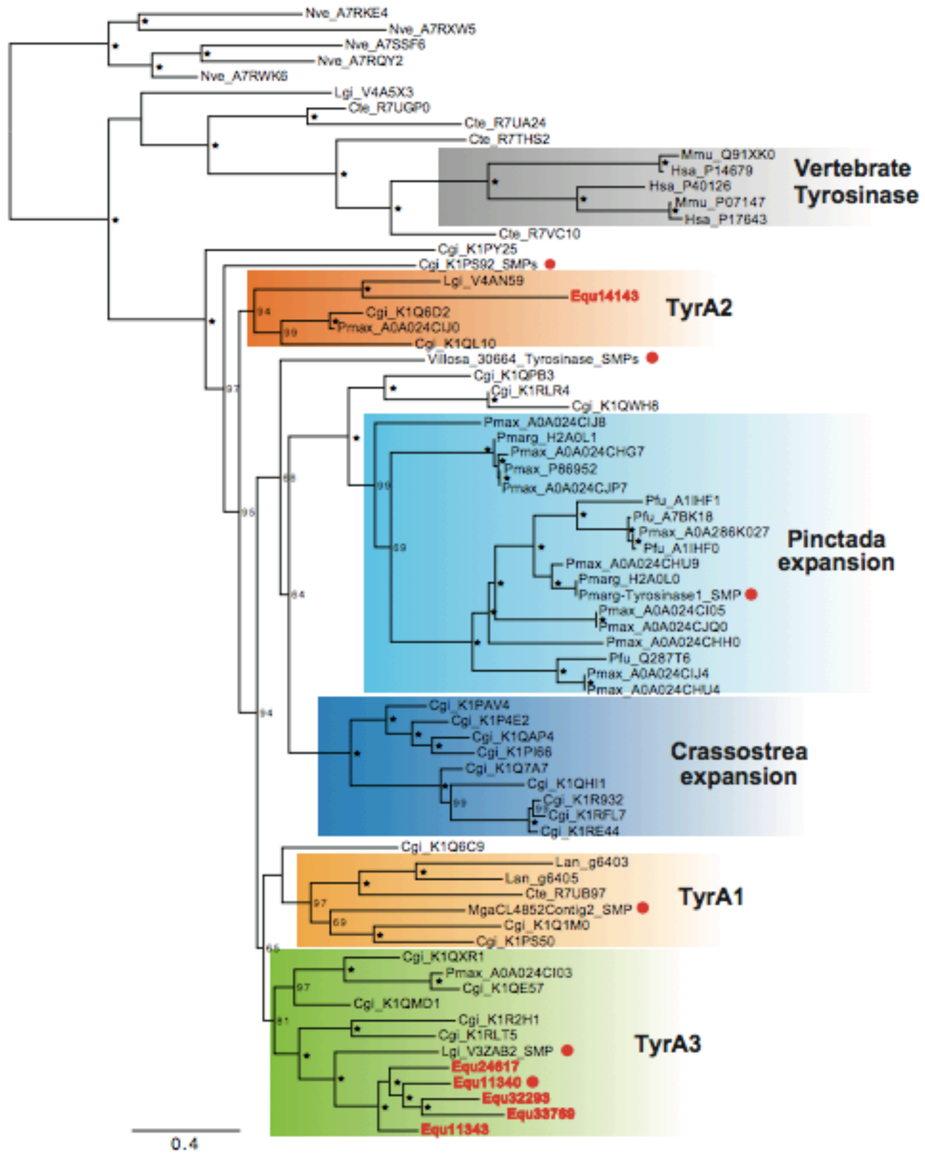

Figure S8. Phylogeny of tyrosinase in various metazoan taxa. The maximum likelihood tree was inferred from 70 tyrosinase sequences under the LG + Γ model (149 positions of the tyrosinase domain, 100 bootstrap replicates). Bootstrap support values below 50% are not shown. Asterisks indicate 100% bootstrap support. Branch lengths are proportional to the expected number of substitutions per site, as indicated by the scale bar. OTU names in red indicate the sequence found from *Euhadra quaesita*, Red circles indicate proteins that have been identified as SMPs in this or previous studies. Cgi: *Crassostrea gigas*, Cne: *Cepaea nemoralis*, Cte: *Capitella teleta*, Equ: *Euhadra quaesita*, Gga: *Gallus gallus*, Hsa: *Homo sapiens*, Lan: *Lingula anatina*, Lgi: *Lottia gigantea*, Mga: *Mytilus galloprovincialis*, Mmu: *Mus musculus*, Nve: *Nematostera vectensis*, Pmarg: *Pinctada margaritifera*, Pmax: *P. maxima*, Pfu: *P. fucata*.

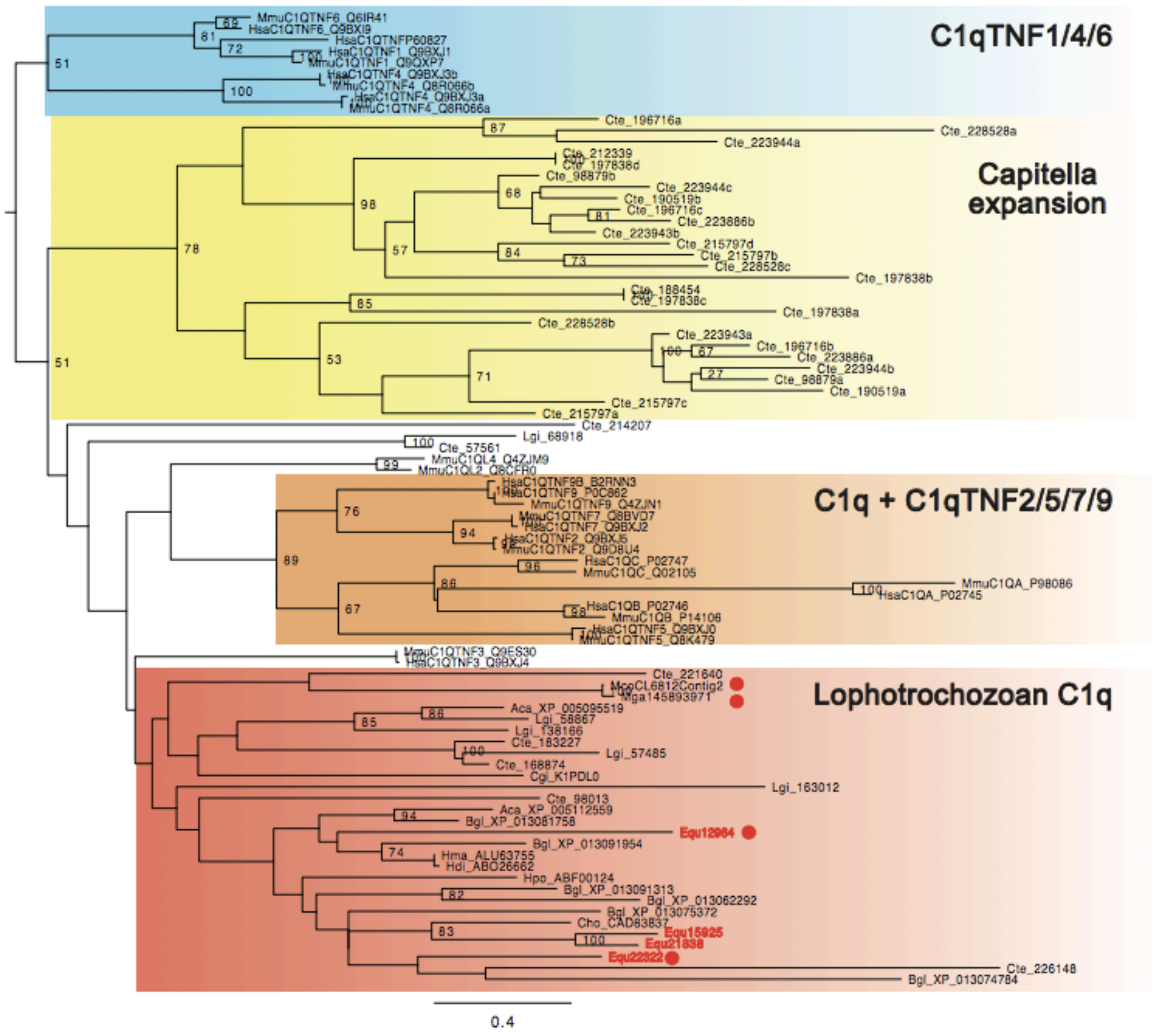

Figure S9. Phylogeny of C1q in various metazoan taxa. The maximum likelihood tree was inferred from 86 C1q sequences under the LG + Γ model (121 positions of the C1q domain, 100 bootstrap replicates). Bootstrap support values below 50% are not shown. Branch lengths are proportional to the expected number of substitutions per site, as indicated by the scale bar. OTU names in red indicate the sequence found from *Euhadra quaesita*, Red circles indicate proteins that have been identified as SMPs in this or previous studies. Aca: *Aplysia californica,* Cgi: *Crassostrea gigas*, Bgl: *Biomphalaria glabrata*, Cho: *Cepaea hortensis*, Cte: *Capitella teleta*, Equ: *Euhadra quaesita*, Gga: *Gallus gallus*, Hdi: *Haliotis discus*, Hma: *Haliotis madaka*, Hpo: *Helix pomatia*, Hsa: *Homo sapiens*, Lgi: *Lottia gigantea*, Mco: *Mytilus coruscus*, Mga: *Mitilus galloprovincialis*, Mmu: *Mus musculus*.

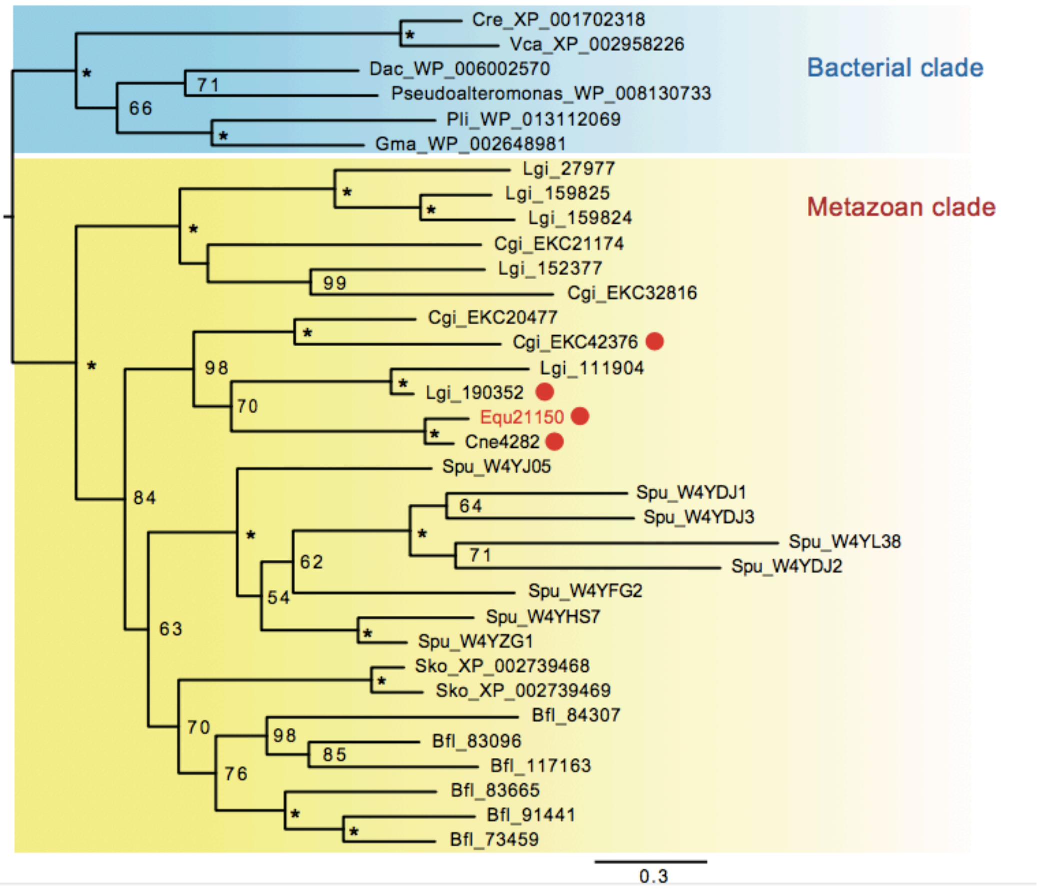

Figure S10. Phylogeny of MSP130 in various metazoan taxa. The maximum likelihood tree was inferred from 34 MSP130 sequences under the LG + Γ model (958 positions, 100 bootstrap replicates). Asterisks indicate 100% bootstrap support. Bootstrap support values below 50% are not shown. Asterisks indicate 100% bootstrap support. Branch lengths are proportional to the expected number of substitutions per site, as indicated by the scale bar. OTU names in red indicate the sequence found from *Euhadra quaesita*, Red circles indicate proteins that have been identified as SMPs in this or previous studies. Bfl: *Branchiostomia floridae*, Cgi: *Crassostrea gigas*, Cne: *Cepaea nemoralis*, Cre: *Chlamydomonas reinhardtii*, Dac: *Desulfuromonas acetoxidans*, Equ: *Euhadra quaesita*, Gma: *Gimesia maris* Lgi: *Lottia gigantea*, Pli: *Planctomyces limnophilus,* Sko: *Saccoglossus kowalevskii*, Spu: *Strongylocentrotus purpuratus*, Vca: *Volvox carteri.*

Supplementary file S1. Alignment sequences for phylogenetic analysis.

Eq16217-21060 (Figure 4A)

>EqA_16217-21060

--------------PPNFKPP---K-QSPFLPPPMPM----PMAPPGPVPTPNDGIFMDMPNAGGGAYGAGP
YMGPPVMYHYCPPGPTTADHCKDQKLQEALYFPDGTPRYNWVPPRNPWDTSLLDTVKETA
KNILMMKVNSRPSRT----

>Cne123_fr2

---------------QNYK-P---KPSSPFLPP--PM----QMAPPGPPRXVNDGLFMDLPHAGGAHYGGGPY
MGPPVMYHYCPPGPTTADHCKDQKLQEALYFPDGTPRYNWVPPKNPWDTSLPDTVKETAK
NILMMKVNSRPSRICLIM

>BglExtensin1_XP_013061844.1

MKWLFSFVLFGVAASAPQFNPPLPPAPFPPFAPPPQPFQPGPPMAPPAPMPGPAGGVLMDLPN
TGGGYAGGYPGYAPPVMMRYCPPGPTSNDACKDQKLQEALYHPDGRPRYNWVPPRNPWDT
SLPDTVKDTALNVLMMKVNSRPGMSCLFF

>BglExtensin2_XP_013061846.1

MKWLFSFVLFGVAASAPQFNPPLPPAPFPPFAPPPQPFQPGPPMAPPAPMPGPAGGVLMDLPN
TGGGYAGGYPGYAPPVMMRYCPPGPTSNDACKDQKLQEALYHPDGRPRYNWVPPRNPWDT
SLPDTVKDTALNVLMMKVNSRPGMSCLFF

>BglFormin_XP_013061847.1

MKWLFSFVLFGVAASAPQFNPPLPPTAFPPFAPPPQPFQPGPPMAPPAPMPGPAGGVLMDLPN
TGGGYAGGYPGYAPPVMMRYCPPGPTSNDACKDQKLQEALYHPDGRPRYNWVPPRNPWDT
SLPDTVKDTALNVLMMKVNSRPGMSCLFF

>BglExtensin4_XP_013061848.1

MKWLFSFVLFGVAASAPQFNPPLPP-AFPPFAPPPQPFQPGPPMAPPAPMPGPAGGVLMDLPN
TGGGYAGGYPGYAPPVMMRYCPPGPTSNDACKDQKLQEALYHPDGRPRYNWVPPRNPWDT
SLPDTVKDTALNVLMMKVNSRPGMSCLFF

Eq23617-24364 (Figure 4B)

>EqC_23617-24364

MGLPDLEAGPEKSP-----TKCLKKSFKWAGGLTGALGITGLLVGLHLSGNLVPETVSAASQTA
DTLAPTFVNSAANSLGAAMIAS---SDGVKDTVSNVVDGFKDAAGNVGDAVANVGDAVANV
GDAVANVGDGVKDIVSDVAGGVKDAVGNVGDAVADVASGVKDVVDGVGDKTKDIVGDV
GDVVKDVVGDVGDVVKDVVGDAGDAVKDVVGDAGDAIKDVVKDVVGDAEDVITD-VSDA
AVKDVAEDVGDVVKDVVDTAGDVLKDVVDDIGDAAKDAAEGVGEHISEGFSEGADIFXEPR
RNQA-----GN-------------------

>Cne_5087_fr4
MHVPDLENGPEKSP-----TKCLKKSFKWAGGLTGALGIVGLLVGLHLSGNLTPETVTSDLQIGE
A-RPKFVNSAANSIGAAIIAT---SDEVKDTVSNVVDGVKDAAGNIGDVVSNVGDAVIG---------
GLGDAVSDVGDGMKDAVGNFGDAVSDVASGVKDAVGGAGDSVKDVVEGVGDVAKDVLK
DAGGAVKDVVDDAGGAVKDVVADAGDALKDVVQ----NAGDVVKDVVDDAAVKDVVGDS
GDALKDVVGDVGDAVKDVVDDAGDVVKEAAEGIAEKAGEGLGEVSDIIQEIRDGLASFFDE
GNQPSSISQPPSVNLDFNLR-
>Bgl_XP_013063539
MGLLEVEA----SPALAAASTCSNTALKWASGLVGLLGTAGVLVGLHATGNLTPETTKGSQSA
LEGQP---LESASNMINTALIAA-LVKDAASDAVGNIKDALPDL-GSVQEALF---DAGSDTHDTL
SDLGGDVHDTLSDLGGDVHDALGDVHDALSDVGGNINDALSNAGDSVKDTLSSLGDNIKESI
PDVGESVADSVDDIDD--EDELGDIIAAMASVSDDIVDSAKDVLSDTAKNAAAKEVSEKLATG
AKDAAENVGDGTKEIVSKAGELAGNAMKNVAEAAKEATGQVADGVKDASGKLSGFLDPPN
NRPTASATPHRDLDFEFR-
>Bgl_XP_013063538
MGLLEVEA----SPALAAASTCSNTALKWASGLVGLLGTAGVLVGLHATGNLTPETTKGSQSA
LEGQP---LESASNMINTALIAAQLVKDAASDAVGNIKDALPDL-GSVQEALF---DAGSDTHDTL
SDLGGDVHDTLSDLGGDVHDALGDVHDALSDVGGNINDALSNAGDSVKDTLSSLGDNIKESI
PDVGESVADSVDDIDD--EDELGDIIAAMASVSDDIVDSAKDVLSDTAKNAAAKEVSEKLATG
AKDAAENVGDGTKEIVSKAGELAGNAMKNVAEAAKEATGQVADGVKDASGKLSGFLDPPN
NRPTASATPHRDLDFEFR-
>Lgi_V4ACQ6
ETLVDIQAGEKVNPADLIGTVRDGEADIAEAKALSATTEAPTDVTIMADMIDTREDAADNMA
DNIDTAQDAQEHAVDNIADMIDTAQDAADNMADNIDTAQDAQENAADNMADMIDTVQHA
RGDMIDTVQDAQEDAVDNMADAADNMADNIEHAVDNIADMIDTAQDAQENAADNMADNI
D----TAQDAQENAADNMADIIDTIQDAMADNVQDAQENAADTMADIIDTIQDAVQDRIDNIEP
KDLVEEIREELEDVVDKIADQ-EQLVGFLDMQLGAELKKNAPRVRRAAGRMA-----------RFAS
K-----GITRSRDRNYFSGLR-
>Lgi_79004
---AAESTALSDEVKVALSSAAGKV
KDALSSAAGRVKDALS---SAAGRVKDALSSVAGRVKDALSSAA-------GRVKDALSSTAGKEN
DALSSVAGRVKDALSSAAGRVKEALSSAAGKVKE---

>Aca_XP_005101505
------------SPR-------------------------PILVG----GRLD--------------IQVFGTRWYTLANDVSDACGSY

DDDVSDAYGSYEDDVSDAGGSYDDDVS---DAGGSYDDDVTDDGGSYDDDVSDAG-------GS
YDDDVSDAGGSYDDDVSDAGGSYDDDVSDAGGSYDDDVSDAGGSYDDDVS-------------------
---------------------------DAGGSYDDDVSDAGGSYDDDVTDGG--

>CGI_10009871_EKC19016
MSTSTVTKTTNSGPASRSVSTKSSSFHR----------------DVSSSGSFTRSTAFSSQARSQLPPPTFIA
SSMTKSNEATIASPESSNMMENATGNEVKVVERAAEAAGDIVEGAQEVAGNVVEGAQEVAG
NVIEGAQEVVEGAQEAAGNLMEAASNVGENMAEAGANVVENMAEGVEKGVEMVKDVAE
NVAERVEDVID---DGLSTDRGDTDDRVNDDKDSRSREIGAVVGARMNDFSTSELTTSSSSRSK
DLQSSASGS-NRATGSPGYQRGD-MRRIISQIKNNGGAVISLPEGKTNTMVHFFVKSNSRTAILR
HPNR--------
>pfu_aug2.0_3272.1_09093.t1
RSTYNIQNSPELMPVTADAARDTNAVNSDSQNII----------DL---------------------TNTAGNNAAN
VAADAMVLSSVADGGISDAVGNVADTISDVGDNMQDAVSNVGDAMGNVVDGVSDVAGDV
VDNVNDAVDNAGDAIGNVGDALSDVGDNVNDAMSNVADNINDMGE----NLGDGIGDVGEN
LRDAVDNVDDAMADNVGDMAENVKDGLDNLADNAGEIIDDRIDDAFVKD----------DSKEDS
SDD-DKDGNKVGFMKGN-LRRLAKRINRSGGAVISVPESKTNTMVHFFVK--RKAGILRHPKK
KTDEGIR-
>pfu_aug2.0_4689.1_32700.t1
IGNDDSEESADDSEEKSHEAVDSSESNE-----------RDVKVSISDDGNDDDESKSREDDVSESESE
TTDDNQDNDSSNSDNDSSESDDNIDDTIDNIKDVVDDGVDNAKDVIDNLNDGLGNTVDNIED
VVNDGVDNLKDVMDDGMDNLDGLGDALHDTVDNINDFVGEGLDNAKDILSDTMDNINDA
VDDGLDGVKDIVDNLKDGINDVVGDGLDNVKDGIDNLGDNLGDNVKDVIDDSENKDNEED
SSTSKEDSKDDSGDAKEDLIPDVGDMIGDAVENVADRVDEGDNKLSDVFDGSSNEMDHHTK
SSNKQHSIMKSTSKSLDNQVR-
>Mye_XP_021366349.1
ISLPEFARSLGHDPKTFHIRAAFSEVDRNSDGKIDVLEHSDVLVGMSKDGDDSDSSDSDSSDDS
DDSDDSSDSSDSSDSSDSSDS-SDSSDDGDGNDNDDDDDDSRNTDDGVNHVKD------DKL
DDTADDMLDNVLDISDNMDDVVDDVDDGVSDVVENVNDAM---GDGISDVVENVNDAMGD
AVDTMADRASDVVDNIKDGRMDAVNDMTGNVAGAVSDVAGNIGD---------AVSDVAGNIG
DTVGDVVDTINDMTEKVVDTVGDVMPGAANLIASRINSGANRRLDIPPNPVPAVARLPDSSN
GRTSVVQT---ARDDSIQ-

Equ26417 (Figure 4C)

>Eq_26417_fr1

---------------------------------------PPARNQVSVLWWPTKEALLYPEIKFVANIKSMGNWFKLQSTP
WKVNRPVDQWANTESMLAQYQAMQNNMRAMERRLEQPV
>Cne_7508_fr3
MATAGLIVAVSSLAWFIAGVASQ-ATDASSHCSYLINSVSRYQPNAAAFKIYTRSRSPRVTSGE
PIEVTIGPFSSLNFFNFTDFILYATPSNIANLEVEFIGPTSPHVGVFQLFDKWRAGAGGLNCNPR
SRAEDSVGAFEDRLLATFKQYYPNNPMLLRYHAPARNQVSVLWWPTKEALMYPEIKFVANI
KSMGNWFKLQSTPWKVNRPVDQWANTESMLAQYQAMQNNMRAMERRLEQPI
>Bgl_XP_013085779
MMFLSAALGVCVVAIMAVTVSSQGAADPTSHCNYLVNSKSQYQENAAPFKLYTRSRQPRITS
GEPIEVTIGPFSQLNFFNFTDFILYATPSNTANLEIEFIGTPNPHVGVFQMFDQWRAGAGGLSCN
PRSR--
>Lgi_234386
MMATLAAMGGKPTAAPAAPAAAKGRQKLSDACSYLKSRTSIWGKNNAPFKLETDGFYGDV
RRGYPIEVAIKPWSQFQPDNFTDFVIYATASGVGAFEAQVMGMPPTLLGVFKVVNDYAAGA
RGFMCDPRAMGPDAFGSAEERRILINRALKPENQYWKTRQPAIRQYAAALWYPTEMAMGA
ANIRFTAKVKSDGHWYELKSKVLTPYNPPDPWQSMAASISQYQRMQQHMRQLERQAKGPP
>Lgi_234387
--------------------QQQGQRKLTDSCTYLQTRSSIWGENSSPFYFNITGFYGQIRSGQPLEVTIKRWS
SITPDNFTDFFMYATPRGIAQMNGQGVGMPVLYLGVFKLVPPYNTGGRGFTCDPRAIGYDAF
GSVEERRILIKRAMEPNKPYWHNRKPYVRKYTSVLWFPTEMAMNVPSIQFIGKVKSDGHWY
EIKSRSFVPYIPPDPMSTMYSSLSQVSSMSQNMRQFERQADAAT

Adipocyte plasma membrane-associated proteins (Figure 5)

>Eq 22744 fr2

-------------------------HSG---
-------------------------IPNSLDMFSNNPAARTRTVTTVSQNTIKSYFPKYGLLVELDANGRIVGSLHD
PTGQVYSAISEAAENQGLLYVASPSNFFTGRIVL-
>Cne 2108 f6

---TGQVYSSISEAVENQGLLYVGSPNTF

FAGRIVL-

>Eq 24422 f2

---D

NIRSSGRGTYWVAMSYARHSGVTNPLDQNSRIPDYRAMGFRWMTLKELRNLFTKWGVVVE

LDDTGAVVGSLHDPTGNTLSMITEVNEFGGVLNIGSHIANYMTRFVL-

>Eq 29973 f3

---ANGAALLTQPARL----------------------

----------ARQHPSSGRGTYWVAMSYARHSGVTNPLDQNSRIPDYRAMGFRWMTQKELKNMFS

KWGVVVELDDTGTVIGSLHDPTGNTLSMITE--------GIGG-----------

>Eq 24195 f2

---E----LFVL------------

---------------------------SG--

-----------KGCVW-----------------SPPLLLS------ELIEQIMDMNEKYGMVVEVNENGEVVRSLQDP

TGNQISSVSEAAEHDGFLYLGSFGQPFVSRIPV-

>Eq 28198 f2

---ISEVAEDKGMVYIGSVVNRFTG

RLVQ-

>Eq 23624 f2

--RC-------------------------------------

---SRISQ------------------

------HPSNQGYFWVGTSP-----KHNFVIIY--QPERSEMLARTYSIKQIVDMLPRYGMALEFNVYG

EILRSLQDPTGDRVPFVTEAQEHDGCLYLGNIDQPFVSRLSL-

>Eq 22611 f2

---------ACA---PKP------------------------------HQGTKLFCSRP---LVCQYCQQDLLGQMAPSHCR

WQTPVWSTYKLRLYANGQLLVLDTFRGLFIVNPTTGNYRLVLSTDTPINGRRPVHLNDMVVT

STGTIIMSDSSDVYDIDKDIYVCMDGRPTGRLLALDPRTAQVTELQSHFNFPNGLELTPDG-DL

LVGETCRATIHRVSLKRNSWLRVEPFSVNLPGLP---

>Eq 22038 f3

-------------IRRPSKSDWTIKTKFELTKAERLKVNELVGPESFAFYKGHIYTGLANGSVVDISGPG

VRTVANMGPPGC---QECGRPLGLTMDNNGRLIVADAPHGLFRVNVDTGEKELLFSVKTVVN

GKQSKFINHPTVAKDGTIYFTDSSTRWSFEEYIYILLEGEASGRLMAYNENLNTTTVLVENFAF
ANGV--

>Eq 31516 f1
--ECGRPLGLRLDSRGRLLV
ADSSNGIFRVNVDTGEKQLVVSSKTLVNGKQSKFINDVIVAKDDTILYTDSSTKWSRKEFLYV
VLEGESSGRLLAYSEKSN--

>Eq 40981 f1
---------------PHP--IMCSSTASCGLLLSIRASNKG
TLLVVDGYRGVFEVDPTNSQVKQLYPRKPA---
---WK----------------------------------T-----------------------
----

>Eq 26376 fr2

--GRSLLVAETGRARILSISLETG
QRPAPTIFTDNLPGVPTNIRGTSHGTFWVGLSLIRHSRIANSMDQYSNNPAARYRTYHRDSLN
QITKYYPSYSLLVHXRG---

>Eq Shell03 39344

------------RPRYLNDVLQTPDGKIYVSDSSDKFDAYRDLYIILEGRTSGRILELNPSTGGISVFAD
GIAYPNGLELTSDGRSLLVA--

>Eq Shell09 25307
---SIPTCGLLTSIRMGVDGQLL
VLDAYRGLFQVNPITGAIRQLYSAINQVGGRLPRYLNDMVQTPDGIVFISDSSDRFDAANDIYI
IMEGRPSGRILALNPVTGAITEVLRVLAYPNGLELTADGTALLIAETGRARIFEX--------------------
----

>Cne 58150 f6
-------------------------------LYNAEKLHFRRVVGPTSFAFLNGSVYTVTSDRKVVDIAMCQPKVIVN
LSTPGCNSIPTCGLLTSIKVGLDGQLLVLDAYKGLYVVNPATGAVRQLYSSINPVGGRLPRYL
NDMVQTPDGIIFISDSSDRFDAANDIYIIMEGGPRGRILALNPTTGAVTEVLRVLVYPNGLELT
ADGTAILIAETGRARILRMSLLAATYRQITTFASNLPGLPGNIRRSARGTFWVGLSLVRHSGIR
NSMDMFSNNPAARSRTVATVDQNT---

>Bgl XP 013063306

--MLTSIRVDNNGKLVVL
DAYKGLYRVDPLTGAIEQLYSSTVQVGGRTPKYLNDIVIKSDGTIFISDSSDKYDAANDMYIIL
EGRPSGRILLYSPDTKQTTEFIKGIVYPNGLELAANETELLVSEMGRARLVRISLLPSTFRQLSL
FSENLPGLPENIRRSSRNTFWVGISYVRHSGIPNSMDTYAGNPAARARTAYVIKKTT-------------
----

>Bgl XP 013096158
PLVSNVEINAASFQLPAPPFLTGNLASNNVLAQAERLHEGVVGTPGSFVMHGDAIYTITRDGM
VVNIAPCRPLVVATLAPRGCQAPKECGFLISIKMNADNMLIVLDAYRGLYEVNPKTGSSRLIY
DAKSLVNGRPSIYLNDIVFMPGGTIIMSDSSPTFDYANEFWIRFEGRPDGRLLGFHPGNGQVKE
ILAGLAYPTGLEITTDGQALLVAEAGRARILR---

>Bgl XP 013075993
STSRALQITAVKYELGKPPTLTGPLEKNFMLTDAEQYKNGELKGPESFVYYKGHIYTGLSDGR
IVDISDCGVQTIARLAPKNCVGEAQCGRPLGMRVDNFGRLVVADSARGIFRINLQTGYVETLY
LSTTLVNGRRCKFINDVVIAKDGTILFTDSSSRWSRKEFLYVVLEGETTGRLLAYSDNSNKTIV
VLDKLAFPNGLEFGPGDDYLLIAETARARIRRLSLKGRTWLQLSNFADNLPGLPDNIRSSGRN
TYWVAMSQARHANMTSMVDEYANQPQMREMIANMATMDSILAMSEKYGMVVEIDGSGRII
RSLQDPTGKTISAVSEVMEKDGFLYLGSFDRPFVSRVPL-

>Bgl XP 013073018
---------------------------------------MGLINGPASFAFHQGELFAGTRDNKVYNLARCKPKLVADLSFP
GCEQTSSCGQLTSLRRDSTGDLIALDTDKGLFRIKNDTGTFEQIYSVDVPVNGRPPVHLNDLV
VTKDGIIIMSDSSDAHEFGADVYIGMEGRTSGRLISFVPSTGVIRELLPVMAFPNGLELTPEG-N
LLVAETGRVRILR---

>Lgi 115667
------------FSLEKPPEFKGPLEPNTLLQKASRLYLDEVKGPESIVVDGGHVYTGTMDGKVIDIFQ
GHVKVLAQLGKPPCEDEPTCGRPLGLKLDKEGYLIVVDAYLGLYRINVATGNINFLFDSKEKI
LGYRPVFLNDVVILKDGTMYITDSSRKWDRRHHPYSILEAEDSGRLLKFSK--GKMTEEVTGIS
FANGVVLSKNEDFVLICETTKARILKYHLSGNKKGELEIWSDNLPGLPDNIRPTSRGTYWVGL
TGVRRADKFNFLDFVCDKPWIKTLVTKVLSMDLIKKVVPKYGLLIEVNSEGKIVRSLHDPTGE
KIPAISEAEEHNGVIYLGSYNLPYISKLYV-

>Lgi 197037
VYFIPSPIDPKPFLYKHPPQLKGPLAVNQALERVDKLFENQVIGPESFTADQGFVYTGSADGKI
WRFKDGQLEMLARTGISHCELEPECGRPKGMKIDKNGDLVLVDAYKGLLKVNLQNKNIEVL
VRPDTDQLREPLRFLNGLAITSDGMIFFTDSSTKWDRRNYRYEVIETNSYGRLLSFNPTTDQV

KVVLEGLYLANGLVLSQDESYLLIVEMTVSRIIKYHLTGSRAGEREVFIENLPGYPDNIQLNSA
GNYFVGMGSVKFEGLGPFLDNIGPYPWFKRFLAKVTPRPLFDVFLPRHAMLVEISPKGKIAAS
YHDPTGSVVRGVGEGYQHGVYIYIGHFSMPFVGRIEI-
>CGI 10024794
ILLTPSPIDPVYFTLPEPPEFEGVLEPNNLLQKSERIFENEISGPESIVVDGDHIYTGTADGKILHI
YKGEISVLAKLGKGPCDNEPTCGRPLGMRLTKEGYLIVIDTYLGLFKVNVATGDHYQLYSAEI
PVNGKRPRFLNDLTIAEDGTIYMTDSSTKWDRRHNRHQIMEGEVSGRVLIYDPKSQEVTELIN
SMSFANGIQLTRSEEALLICETTRARLLKYHLKGPKKGSLEVINNNLPGIPDNIRRSSTGGYWI
GMALIRKKNKISFIDYCAEKPWLRALIMKVVSMDLVLQYLPKYGLVVEVNEEGKVIQSLHDP
TGQVIPAVSEVEDKDGVLYFGSYNLPYLGRLYL-
>CGI 10018671
LYKKLMGMSTMCYSYPKPPRLEGALAINNLLQGATRAFQGQITGPESFAVDEGVLYTGLADG
RIVAFKGGELWQLTRTGEPHCDLEPVCGRPKGMKVDPTNPLIVLDSYRGLLQVDTKTGDIQV
LLPSSTGVNGEPLKFLNALDITHDGIVYFTDSSKKWDRRNYRYEVIEVNRQGRLIMYNMVTR
ETKLLLDDLHLANGVALSSDESMLFIAEMSACQIRRYFLKGPRAGQSDVITQNLPGYPDNIKL
NSQQNFYVGLGSVRYQGLGPFLDLIGPYPAIKRFLTKLTPLKVFDIFMPKHSIILEINRHGEIISS
LHDPGAKVISASGEGFEFNNTLYIGSFWTPYIGMLNL-
>pfu aug2.0 640.1 00979.t1
IALIPSPIDPVKFSFPERPEFTGALEPNTHLQKAERIYENQLNGPESIVVDGEHIYTGTADGKLLD
IYKGEIRVLARFGKEPCEDEPTCGRPLGMRLNKDGYLIVIDAYLGLYKVNVATGDYHQIWSSS
TLVNGKKVMLLNDLTIASDGTIYLSDSSVKWDRRHNRYTIMEAEASGSYDEYYT----------LLR
FQTVPQY-------------------------------IFMLNLPPLSEQV--IPMDAIL
AALPKYGLLIEVDDNGNIMRSLHDPTGKVIPSLSEAEDKNGVLHFGSYNLPFMGRLYL-
>Cte 171602
VIVVPSKVNAVEYSLPNPPVWEGPLKPNDLLLQSERIFEGKIKGPESMVNQNGHIYTGTADGK
VLHIYKGEIQVLATLGQPPCADEPNCGRPLGMRIDKEGYLVVIDTYLGLFRINVATGDVFQIFS
TSMKIGNRDPVFMNDLDVASDGMMYITDSSI-FQRREFPLDVLEGRNHGRLIQYDPETNSSRVI
LENLAFANGVQLSKKEDFVLVAETTRFRIIKYHLKGPKTGRAEVFIENLPVSPDNIRRSSTGGF
WVAGAVCRGHHTFNLFDWIGPKPWLRSLVSRQLPLWLVHKALMPCGMILELNQQGDIVRAF
MDPSGEKVAFLSEVEDDSGILYLGSFSTPFMSRLNI-
>Cte 198473
LYLWPSKFTPVAYRFPDSPPLTGALQPNSELAKAKQGFKGELNGPEAIVSHEGVLYTGSADG
KVLSIHNGEILVLAQFGPNPCYYEEICGRPLGMAVSFNGHLWVIDAIFGLYSVNMTTGEFKRK
VSADQLIAGRYSKFFNDVSISPNGRVYISDTSTKWRRTDFFVLGLETNPDGRILEYNPATGDLI
EVCTGVS-PNGIQITSDGSAILISDTSFATISKCQLIGKTRGKVEVVMKNMPGFPDNIRASPRGT

HWVALTAVRQPT---LIELVSPCPFLKEMIYKLWKSEPVRDMIPKYGLIIEINEKGHVIRSLHDNS
GK-ISSISHVQEEDGVLYLGSATNDYIGILQL-

>Lan g2080.t1

LYLYPSPIQPEPFTYPPAPRLEGALAVNYKLQKAERWFEGRLAGPESFTVDKGYIYTGLNDGR
LVRFRGNHLEELGRTGQTECAYEPMCGRPKGMKIGPDGHLYFVDAYQGLMRLNLPSQEMET
LVPSENGSEGLPFRFLNGLDVSKDGVVYFTDSTPKWQRNQYKYAVLETNAMGRLLAYNVKT
RKVRTLLTGLYLANGVAVSTDQSYVLVVEMTAARITKLHLRGSKAGVREILAENLPGYPDNI
NPTPQGTFYVGLTALRFEGFGPFVDLTAPFPGLKRFIAKVTPLRLYDYFISKRAMFIEIDQNGEI
VQSFHDPDGSVIGYVAEAFPHDGKIFLGHFSQPYIGVLSM-

>Lan g31395.t1

--KLFTGTADGKILEINGDDIRVVATLGKPPCDDEPTC
GRPLGMRFDKDGSLVVVDAYLGLFRVNVETGKFVQLYSPETLVDGEQAAFLNDLDIDSDGT
VYFTDSSTNWGRRYNRYAAMEAKKNGRLLAYNPNTKQTEVILKNMSFPNGVQLSPEKDFILI
AELCTCKILQYHTKGPRKGEVEIFSDNLPGAPDNIRPSSSGGYWVGLATTRRAGKFSLLDYTS
TKPWMKKLIFKVFGMKMRNVF---

>Lang24100.t1

LCLLPSPIEPIEYSLPLPPQFIGPLAENKLLAKADKLFLDRLHGPESITVHNGKLFTGTADGKILE
INGDDIRVVATLGKPPCDDEPTCGRPLGMRFDKDGSLVVVDAYLGLFRVNVETGKFVQLYSP
ETLVDGEQAAFLNDLDIDSDGTVYFTDSSTNWGRRYNRYAAMEAKKNGRLLAYNPNTKQTE
V---FSQEFLLKMIPKY
GLLIELDASGNIIRSLHDPSGEDVPNVSEVEDDNGVLYFGSYYLPYIGRLKL-

>Nge g4549.t1

MAVINIPIDPVSFRLPLPPQFKDGLTRNNILQYTQHILENEVIGPESLAVNKEAIYTGTYDGKIV
EINQGSMRTITELGKAPCSYEEHCGRPLGIRIDKDGDLVVADAYLGLFKVNPKTGEHKMLLSS
STKIDGVKPTFLNDLDIAKDGTIYVSDSSSKWQRKDVHLMILEGGTDGRVLWYEPSTNTSGV
LVDGLCFPNGIQLSPSGNFLYIAETTRARIRQYHLSGPLKGQLHVFSANLPGLPDNIRPSESGGY
WVALALTRMSEKLSVFDLTAPYPWMRNIVSKLMSYEVFLKTVSKYGLVVELDSSSKVITTPL
D---------------------------------

>Nge g12861.t1

IYLLPSPLDPEPYIFPDYPELTGPLTPNKKLQKAKHIFENIVIGPETFTADEGYIYTGSADGKLW
RFKGEDLQLLGRTGIERCEMESVCGRPKGMKIDHHGDLVFVDSYKGLMKMDLTTKKIEMLA
SSEKGIDGVPFMFLNSVDIAKDGTIYFTDSTTRWTRKDYRYEVIETNHLGRLLTFNPRTKKLT
KILDNLYLANGIALSEDGSYILINEMSVIRISKYYLKGPKAGTLEVIMENMPGYPDNIKLTKSG
TFYVGMGVSSFPDLTKILDNLGSYILINRISKQVTPFWAYSAFLPRHNYFIEINGDGKILRSFHD
PEGKVIRGVGEVFEHDRRLYLGHHEFPYVSVMDI-

>Pau g16754.t1

VVFCDARIDLGAYLQPAGKAVVGPKQLSKLDFRAREVMLGLLSEPKVAGLGEGEVW----------

----QARALEDLIIHFCKKETEYRRKVSSMM-MIGVLLHSVRFCGVMLRRVFVCTYTPLLEIQMS

CATSVKNFLRYFGF--SGLI------------------------CRLLEINPETEEVKVLLSNLNFANGVVLSYKE

DYILVAETLLARIQRLWLKGDKKGQVDIFANNLPGLPDNIRRSGRGTYWVAMATTRKADKFS

MFDFTASRPWLRKLVAKVVSQEALKKLFSKEGLIVELDKEGRIVQSLQDPTGKVVKAASEVE

EKDGTIYIGSYVNSHLSTLNL-

>Pau g16018.t1

---MEIYDGKLREVTRLGKPPCADEPTCGRPLG

MKFDPQGFLIIADAYLGLYQVNVVAGDTTQLMSSQRL----QSRFLNDMDISRSGMIYVTDSSL

KWSRRHFMYCALEGRPTGRYIDI---

--ALH-----------------------------------

>Aca XP 005089642

MLLLPCPIEPLEISLTDRPELTGVLKPNNLLHLCEKLYQNHLEGPESIEIDGEHIYTGTADGWV

KHIHKGEVQNLVRFGTPPCMNENTCGRPLGMRMSKDGFLIVIDAYYGLYKVNVATGDYTLL

VSAKTPVNGRPLKFVNDLDFGHDGKIYFTDTSSRWHRNQFMNVVFEGSATGRLLVYDPVTG

DVKQLMDGFLFANGVQLTKDKKSVLVSDTMAGRIYRYILQ---SGDTVVWADNLPGLPDNIR

YCTSGTYWVGFAGVRQAGKFSIVDSLASHPWIRGILAKVVTVSTVAKHLSSKAITLEMSEDG

KILRSLQDSTGAALHGASQVSCADGALYFGSFHSTYLSRLYK-

>Aca XP 012944585

---MKVDSSGRLVVADA

YNGILRIDVDSGETEVLVDR---FNGSRFLFLNALDIASDGTIYFSDSSTKWERRDYRYEVIETN

KLGRVLALSPQSKEVRIVQDGLYLPNGLSLSEDENFLLVAEMSVSRISRIYLKGPRANSVEVLT

ENLPGYPDNIKRNSRGNYYVGMGSVRFQGIGSFLDLVGPYPPIKRFITKIVPASLFDVFLPKHA

MLLEMDEEGNIVTSHHDPGALVVRAVSEAFEHSGKIYIGHFKIPYIGVIKK-

>Aca XP 012935592

LMISPSKIQPVAFRLPKPPTLQGPTAPNTALATAEIVHMGKLQGPASFVNTGEDLFVATKDNKI

YNVARCQPKLIVDMRPQGCRTRQTCGQLISLRKDTTGDLLALDSFRGIFRVNSTTGETSQLFSS

QTPVNGRAPVHLNDFVITRQGFIIMSDSSDSHDFDNDIYIGMEGRTDGRLIAFIPSSGAVNQLRP

KFSFPNGLELTSDEKNLLVVESTQVRVVSVSLETNTFLSVTPLSNNLPGIPDNIRASDHGTFWV

GMMFARHAASKNPMDLYSSNTMFRSMGAQRMSKKALKNMFSRWAIAVELDASGKILRSIQ

DPKAARMSSVTEVSDYGGVLNIGSVDSKFMTRILD-

>Aca XP 012937792

---MGIRMDKDGSLLVVD

AYFGLFRVNKTDGAYETLYTTSFHVAQKQPRFLTDLDIGPDGKIYFVDASTKYYKNQYHYAL

LEMSSSGRLLMYDPKTKVTLQLADQLAYPFGVQLSKDKTGVLITSPLLHAIYRYDLK---TRNV
SVWTEGLPGMPLGIRYSSSDSYWLAMSSPRSVERPNMFENLAKSPSNRRYAAKFVSMDMIET
LRQEEAMVAEVTASGTIGRVLQDSNTNPLGAVTEAQHVNGVLYTSSFGADAVHTVNL-
>Aca XP 012940879
RTTGAVNIDAATFKLPPPPALSGTLATNNVLYEAERVHDGQIAAPHSYVPLGDSLFAVTKTGL
VINVAPCRPVLLADLKPRGCRSPKECGHPVSMRLNADKHLIVVDAYRGLFDVDPVTG----------
--FDPGNRQVKEILVGLAYPSGLELTTDGQALLVSERRT
MQ---QLKMMYHKRGMAVELDDRGRV
RSSVHDPTGLKVSEVMEVKETDGMVYIGSRDQDAIIRFPL-
>Hsa Q9HDC9
MMLLESPIDPQPLSFKEPPLLLGVLHPNTKLRQAERLFENQLVGPESIAHIGDVMFTGTADGR
VVKLENGEIETIARFGSGPCDDEPVCGRPLGIRAGPNGTLFVADAYKGLFEVNPWKREVKLLL
SSETPIEGKNMSFVNDLTVTQDGKIYFTDSSSKWQRRDYLLLVMEGTDDGRLLEYDTVTREV
KVLLDQLRFPNGVQLSPAEDFVLVAETTMARIRRVYVSGLMKGGADLFVENMPGFPDNIRPS
SSGGYWVGMSTIRPNPGFSMLDFLSERPWIKRMIFKLFSQETVMKFVPRYSLVLELSDSGAFR
RSLHDPDGLVATYISEVHEHDGHLYLGSFRSPFLCRLSL-
>Dre Q803F5
VFVLESPIQPEVFSLNEPPLMTGCYEPNLKLRQAERLFEERLVGPESLANIGDVFYTGTADGKI
VKIEGRNIHVLATIGKPPCEHEHTCGRPLGIRVGPNGTLFVADAYLGLFEVNPVTGEVKSLVST
EKRIAGRRLGFVNDLDVTQDGKVYFTDSSSRWQRRDFMHLIMEATADGRVLEYDTETKEVN
VMMENLRFPNGIQLFPDEESVLVAETTMARIKRVHVSGLNKGGMDTFIENLPGFPDNIRRSSS
GGYWVAMSAVRPNPGFSMLDFLSQRPWLKKLIFKLFSQDTLLKFVPRYSLVVELQSDGTCVR
SFHDPQGLVSAYSSEAHEYSGHLYLGSFRSPYLCKLDL-
>Gga Q5ZIF1
TVLLDCPIDPQPISLKEPPLLTGVLEPNNKLQKAERLWENQLVGPESIVNIGDVLFTGTADGKI
LKIEDGEVQTVARIGHGPCEDEPTCGRPLGIRVGPNNTLFVADAYYGLYEVNPGTGETKMLV
STKTLIEGQKLSFLNDLTVTQDGKIYFTDSSSKWQRRDFLFLVMEGTDDGRLLEYDTVTKEV
KVLMVGLRFPNGVQLSPAEDFVLVLETAMARIRRYYVSGLMKGGADMFVENMPGLPDNIRL
SSSGGYWVAMPVVRPNPGFSMLDFLSEKPWIKRMIFKLLSQETVTKLLPKRSLVVELSETGSY
RRSFHDPTGLTVPYVSEAHEHNGYLYLGSFRSPFICRLNL-
>Mmu Q9D7N9
MMLLESPIDPQSFSFKEPPFMFGVLHPNTKLRQAERLFENQLSGPESIVNIGDVLFTGTADGRV
VKLENGEIETIARFGSGPCDDEPTCGRPLGIRAGPNGTLFVVDAYKGLFEVNPQKRSVKLLLSS
ETPIEGKKMSFVNDLTVTRDGKIYFTDSSSKWQRRDYLLLVMEATDDGRLLEYDTVTKEVKV
LLDQLQFPNGVQLSPEEDFVLVAETTMARIRRVYVSGLMKGGADMFVENMPGFPDNIRPSSS

GGYWVAAATIRANPGFSMLDFLSDKPFIKRMIFKMFSQETVMKFVPRYSLVLEVSDSGAFRR
SLHDPDGQVVTYVSEAHEHDGYLYLGSFRSPFICRLSL-

Alkaline phosphatase (Figure 7)

>Eq16104 F3
--LLKNLFKVMAEAGKMTGL
LSTARLTSPGVAGTFAATPDMQMESDVHLTEAGCGKFKDIAAQLIKDYDINIIIGQGREYFMK
PSSRNVSSNSSVYPWRRD-LDLTTYWAMRKKTYKEAVGTQA---VKDALLQYFLGFLD------EV
QSSPA--DDLQDLVSLVVSALKPNNNGYF---
--PS--------------------------------

>Eq21136 F2

----------------------------QVILGGGRRSFLNNTTPDPSTGQIPRNHRKDGLDLREEWKARGVSH
AYVETKGQLEAVDPEKTDYLFGLFHSNHMTYELERDNSGEPSLPEMTEKAIQILRRNPKGYIL
LVEGARIDQAHHNNSAKKALEEVSVFDDAVILVKKMVSPADTLHIVTADHSHVFFMGGYPSR
GNNILGTVDVINSDKLPFLTLSYGNGPEF--GRNDLTNVDTTANDFRQPGCLPMLGETHGGEE
VAIYARGPMAFLFHSTHEQSFIGHVMMFSTCI-

>Eq22374 F1
--GLSAQAELGNCNSSKGTEVLSVLRWSA
NAGKSTGVVTTTRITHATPASSYAHSADRDWESDRLIPEEE-KACEDIALQLITRNDINVILGG
GRAYFYPENYSDPDPNNTNDVSRLDGRNLVEEWKNRNLTHQLCTTNRGCKTQPCNRVGPLP
GPYGLSGQKQ--DR-----TILAEMTQKAIDILKRTTKDF---
--SC--

>Eq23011 F1

----EGDVNLPDDEDDHVDDIAKQLILNNDIKVILGGGRRYFLDNCTQDPVLGTVDPHQRRDGL
NLVDEWVRRNASYRYVWEKSDFDTVDGNTTDFLLGLFNPSHMDFDLHDSSQTEPTLVEMTQ
KAIEVLSKDPKGYFLMVEGARIDFGHHANSAITAITETLDLDEAVHTAVRMTGSHETLIIVT----
----

>Eq24827 F2
DGMGISTVTASRIRGGQLRGEDGEENLLFFERFPHVGLVKTYSADSQVTGSAAAGSAILTGVK
INSGVLGCDSRVKKGNCTD--------------------------HRT---
---PNV-------------------------------------

>Eq26948 F3
------------------------------KLSFEEFPHVGLSKTYNLDRQVPDSAGTATAIMTGVKVNLGS----------------
---WESM---

>Eq28614 F3

---DI--PDGLPYTTLGYTNGPV
F--GREDLTHVDTGSPGFRQSGCIPVSIETHAGEDVSVYALGPMAHLFHSTHEQNYLYHVMEY
AACV-
>Eq35443 F2

--ETLIVVTADHSHVLNIAGYANRGTPILGLSTDGP
GDGKPYTTLLYGNGPGYRSQRQDLTDVDTTDKKIQ----VPVG--C-
-
>Eq39578 F3
DGMGLPSVTAGRIYKGQKRGQT--

>Eq46746 F3
--TTRITHATP
AGAYAHSGDRDWETDRAIPESE-RACEDIALQLITRNDINVIMGGGRGNFYPDGFTDP-------------
----

>Eq52233 F3
--C--------------------------------------T---------
---KW--P
--MAEAVEMATRLTSKSDTLMVVTADHSHVFSIAGYASR
GNP--
>Cne20308 F1
EGLDSHTISVARILETQTTT---VNNYMTAVDFFLSGHLKPASRDFIVADPAAAVSAVFNGLPEY
NGQMGWVEQED-APCGFRSKKLLKNLFKVMSEAGKMTALLTTTRLTSPGVAGAFAASPDMQ

IESDIHLVESGCGKFKDIAAQLIKDYDLNIIIGQGREYFMKSSNRDVSTNSSVYPWRRD-LDLKT
YWAMRKKTFKDAVGTQA---VKDALLQYFLGFLD------EVQSSPA--DDLQELVSLVVGALKPN
NNGYFLTIYDDHLARA-HENDAYNVGSDLNNILKAINYLDTILTTDDTLIVLASVQGSTLTLSS
GRLHEETIVGSSFTDTS------KIRYPDGSR---------SGDNGTNS----------QDPAGGPDLPVFARGPG
AQAFTGVNDFAYMYHALLYATCL-
>HsaP10696
DGMGVSTVTAARILKGQKKDKLGPETFLAMDRFPYVALSKTYSVDKHVPDSGATATAYLCG
VKGNFQTIGLSAAARFNQCNTTRGNEVISVMNRAKKAGKSVGVVTTTRVQHASPAGAYAHT
VNRNWYSDADVPASAREGCQDIATQLISNMDIDVILGGGRKYMFPMGTPDPEYPYSQGGTRL
DGKNLVQEWLAKHQGARYVWNRTELLQASLDPVTHLMGLFEPGDMKYEIHRDSTLDPSLM
EMTEAALLLLSRNPRGFFLFVEGGRIDHGHHESRAYRALTETIMFDDAIERAGQLTSEEDTLSL
VTADHSHVFSFGGYPLRGSSIFGLAPGKARDRKAYTVLLYGNGPGYDGARPDVTESESGSPE
YRQQSAVPLDGETHAGEDVAVFARGPQAHLVHGVQEQTFIAHVMAFAACL-
>HsaP05186
DGMGVSTVTAARILKGQLHHNPGEETRLEMDKFPFVALSKTYNTNAQVPDSAGTATAYLCG
VKANEGTVGVSAATERSRCNTTQGNEVTSILRWAKDAGKSVGIVTTTRVNHATPSAAYAHS
ADRDWYSDNEMPPEALQGCKDIAYQLMHNIDIDVIMGGGRKYMYPKNKTDVEYEEKARGT
RLDGLDLVDTWKSRYKHSHFIWNRTELLTLDPHNVDYLLGLFEPGDMQYELNRNNVTDPSL
SEMVVVAIQILRKNPKGFFLLVEGGRIDHGHHEGKAKQALHEAVEMDRAIGQAGSLTSSEDT
LTVVTADHSHVFTFGGYTPRGNSIFGLAPMLSDDKKPFTAILYGNGPGYGGERENVSMVDYA
HNNYQAQSAVPLRHETHGGEDVAVFSKGPMAHLLHGVHEQNYVPHVMAYAACI-
>HsaP09923
DGLGVPTVTATRILKGQKNGKLGPETPLAMDRFPYLALSKTYNVDRQVPDSAATATAYLCG
VKANFQTIGLSAAARFNQCNTTRGNEVISVMNRAKQAGKSVGVVTTTRVQHASPAGTYAHT
VNRNWYSDADMPASAREGCQDIATQLISNMDIDVILGGGRKYMFPMGTPDPEYPASQNGIRL
DGKNLVQEWLAKHQGAWYVWNRTELMQASLDQVTHLMGLFEPGDTKYEIHRDPTLDPSL
MEMTEAALRLLSRNPRGFYLFVEGGRIDHGHHEGVAYQALTEAVMFDDAIERAGQLTSEED
TLTLVTADHSHVFSFGGYTLRGSSIFGLAPSKAQDSKAYTSILYGNGPGYSGVRPDVNESESGS
PDYQQQAAVPLSSETHGGEDVAVFARGPQAHLVHGVQEQSFVAHVMAFAACL-
>HsaP05187
DGMGVSTVTAARILKGQKKDKLGPEIPLAMDRFPYVALSKTYNVDKHVPDSGATATAYLCG
VKGNFQTIGLSAAARFNQCNTTRGNEVISVMNRAKKAGKSVGVVTTTRVQHASPAGTYAHT
VNRNWYSDADVPASAREGCQDIATQLISNMDIDVILGGGRKYMFRMGTPDPEYPYSQGGTR
LDGKNLVQEWLAKRQGARYVWNRTELMQASLDPVTHLMGLFEPGDMKYEIHRDSTLDPSL
MEMTEAALRLLSRNPRGFFLFVEGGRIDHGHHESRAYRALTETIMFDDAIERAGQLTSEEDTL

SLVTADHSHVFSFGGYPLRGSSIFGLAPGKARDRKAYTVLLYGNGPGYDGARPDVTESESGSP
EYRQQSAVPLDEETHAGEDVAVFARGPQAHLVHGVQEQTFIAHVMAFAACL-
>MmuP09242
DGMGVSTVTAARILKGQLHHNTGEETRLEMDKFPFVALSKTYNTNAQVPDSAGTATAYLCG
VKANEGTVGVSAATERTRCNTTQGNEVTSILRWAKDAGKSVGIVTTTRVNHATPSAAYAHS
ADRDWYSDNEMPPEALQGCKDIAYQLMHNIDIDVIMGGGRKYMYPKNRTDVEYEEKARGT
RLDGLDLISIWKSRHKHSHYVWNRTELLALDPSRVDYLLGLFEPGDMQYELNRNNLTDPSLS
EMVEVALRILTKNLKGFFLLVEGGRIDHGHHEGKAKQALHEAVEMDQAIGKAGAMTSQKDT
LTVVTADHSHVFTFGGYTPRGNSIFGLAPMVSDDKKPFTAILYGNGPGYDGERENVSMVDYA
HNNYQAQSAVPLRHETHGGEDVAVFAKGPMAHLLHGVHEQNYIPHVMAYASCI-
>MmuP24822
DGMGVPTVTATRILKGQLEGHLGPETPLAMDRFPYMALSKTYSVDRQVPDSASTATAYLCG
VKTNYKTIGLSAAARFDQCNTTFGNEVFSVMYRAKKAGKSVGVVTTTRVQHASPSGTYVHT
VNRNWYGDADMPASALEGCKDIATQLISNMDINVILGGGRKYMFPAGTPDPEYPANETGTRL
DGRNLVQEWLSKHQGSQYVWNREQLIQKAQDPVTYLMGLFEPVDTKFDIQRDPLMDPSLKD
MTETAVKVLSRNPKGFYLFVEGGRIDRGHHLGTAYLALTEAVMFDLAIERASQLTSERDTLTI
VTADHSHVFSFGGYTLRGTSIFGLAPLNALDGKPYTSILYGNGPGYTGERPNVTAAESSGSSY
RRQAAVPVKSETHGGEDVAIFARGPQAHLVHGVQEQNYIAHVMASAGCL-
>MmuP24823
DGMGVSTVTATRILKGQQQGHLGPETQLAMDRFPHMALSKTYNTDKQIPDSAGTGTAFLCG
VKTNMKVIGLSAAARFNQCNTTWGNEVVSVMHRAKKAGKSVGVVTTTSVQHASPAGTYAH
TVNRGWYSDAQMPASALDGCKDISTQLISNMDIDVILGGGRKFMFPKGTPDQEYPTKQAGTR
LDGRNLVQEWLAKHQGARYVWNRSELIQASLNRVTHLMGLFEPNDMKYEIHRDPAQDPSLA
EMTEVAVRMLSRNPKGFYLFVEGGRIDHGHHETVAYRALTEAVMFDSAVDKADKLTSEQDT
MILVTADHSHVFSFGGYTQRGASIFGLAPFKAEDGKSFTSILYGNGPGYNGARADVTEEESSN
PTYQQQAAVPLSSETHSGEDVAIFARGPQAHLVHGVQEQNYIAHVMAFAACL-
>Lgi179711
DGMGISTITAARLYRSQLQKEKNGDAALEFEKFPFVGLSKTYNANRQTSGSASTATALLSGV
KINQGTIGVDNTVVRHDCSSTKESQLTSILDWSMTEGKSTGIISTARLSHASPAATYGHTASRA
WEGDADMTNVT-GNCKDLSLQLFEDNNITVIMGGGRRTLLPKDFIDPQTNSAHSKQRIDGRN
LIEEWKKRSKSHSYITNKKQLMEVDASKTDYLLGVFSASHMDYEIDRDDSGQPSLAEMTQKA
IQILKKNDNGFFLFVEAARIDHGHHATKAKKAIHDVLSFDDAVKAGMDETDADDTLIVVTGD
HSHVFNIAGYPAIGNDIF------------------------------------DKEYQQPAAVPVVDETHGGEDVAIFA
TGPMSHLFRGVHEQNYVAHVMAYASCL-
>Lgi236474

DGMGINSVSAARLYKAQLTQQEEGTSTLAFEKFPYIALSKTYNTDRQTPDSAGTATAFLCGV
KANLGTVGVNSNVPRFNCSLEEGNTVQSIIDWSEAEGKSTGIVTTARVTHATPAAAYAHSAD
RDWENDVN------GSCKDIARQLIEDNHIEVILGGGQREFIPEG---------DGGRRTDNRTLTEEWIE
KKKTGHYVTTDTEFKTVDLSKTDHLLGLFAMSHMDYAIDRNDSDQPSLAEMTATALKILQK
NDKGFFLLVEGARIDHGHHENWAKRALYDTIAFDEAVEEALKNTDKEDTLIVVTADHSHAFT
MAGYPEKGNDIFGIVKPVKPDGLPYLSLVYGNGKGANGTRRNFTDVDTHNNDFRFHAAVPL
PYETHAAEDVGIFATGPMSHLFHGVHEQNYIAHVMAYASCV-
>Lgi196246
DRKYISSVVSADIFHGETELSSSINRKIEFRQ-PTESLEKTLANTHRVPDSSSSGTSQFDTGSQTS
DITSVTSPISE---AASADTDISSPNQNSTDEEK-----LCQRLTYVPPA----NSPPRFSLENLE------LE
CNDYAALFA----LCMLYAIGQNEGLSQNVLDQVL-MPTDKSRKHHYNVIL----RLLRIITIAAQS
NLATLEMT-IKLLKQLTFKEDTSYLQDR----HASVENAKERSTQLLMKNPNVEYLMMDASGID
PCGEVERARRAIRLSLTLLNEIDIHLPLTKNNSDLIACTIDPVQLILVEPDTKRGWGVVRFVGFL
Q------------------------DIEVT------------GDKDDSRSLHIVVHKPSSSLLAGIFDDH--------IRCM-
>Lgi83132
D---------VR--TGVKNKMKQHNC-------------------------
--SVTGATPA--------------KEKPSE-----PTIPAECSYQ---------GKQVF-------------------------------RH
CGLFGDPHLSTFNGEFVQGAWPLVNNEHLTVQVTN-----PVIGDSGATATSKLKKNRSDNFVM
YQAQTDDYDGHTFYGKHKSVELLEIEEGKHVEINIRYIATTIVVRQVNNSHSATSNGLCYR----
----------GCPLSRINYQE----LASKQDIVNLSENSANLRKDAELICR----EANVVDFYFDSCVFLMT
TG--DQNFTLAAL-------
>Lgi121655
DQTSLDKLLNYNPYKYEVSERLGKEQYAILYRCRKLRIKDTYQYDDGVDDGSDTFAREPFAV
RFESPTTEIKDFAII-------------------------------IHTAP--------------RNAVNEIK---HQVYEQTKHH-
LEDIIIAG---DFNAD-GDYV--------------RKGK-ITL-----------
RSD-RRFSWLIEDD-ADVG-STDCAYDRFVAGRKLTSAVVPGSATVYRFDLAFDLTSDH---------
-----------------------------Y---------------------------------------PIELKIAGLGRSTRICFR--------
>Lgi168246
DLTGIGKIVSVK-YAFVHSSKIGKEYYGFIYRTDKVSVKKTYQY----NDVPDWFEREPFSVLFH
VPTAAIKDFVLT-------------------------------THIKP--------------SDAPAEIG---PTVYDEVSSA-TK
NVLIAG---DFNAD-CSYL--------------SATA-SPL----------H
AD-SRFTWLISTA-VDASHHTNCAYDRFVVGSEFKAAIDPKSAKVYNYETGLHLTSDH----------
----------------------------Y---------------------------------------PIELIIK---DSSHVIG---------
>Cte29206
--VVTNTGITHA

TPASAYANSVSRNYESDKDIIFPW-QNCTDIASQLIEKGDIEVAMGGARSKFFPEEEKDPVQ-PE
SFGVRKDGRNLVEEWKEKGKSHEVVFTAEELEEVNTEETDYLFGLFSRGHMSYHRERPD-TE
PALSKMTQKAIEVLQKNEKGFFLMVEGGRIDMAHHGSRPRRALTDTLEFSQSVEMAVKMTS
EEDTLIIVTADHSHVMAFAGYHTKGSDVLGTG-----DIMPYSKLSYTNGPGYSCRRGNLREVDT
SHNDFVAQSAAMLGSETHGGEDVAIYASGPMAHLLQG-----------------
>Cte176469
DGMGMTSITGARWHMKQDEDLIAYEAELSWDKFPTVGLSKTYNTDIMTPDSAGTATAFLCG
EKAREGVIGVNQDVHYGECETTEDTELDSILKIAQTEDKWTGLVTTTRITHATPAASYAHSAH
RYWENDNEIPDDQKEACEDIAKQLVYSGNLKVIFGGGRSQFRPNTTMDPEY-SDYPGFRGDG
RDLIAEWISAGVNFHYVQNQTQFDNIDVENVDHVLGLFNPGHMNYEADRADDGEPSLEEMV
QKAITILERSPNGYILFVESGRIDHAHHDTNANRALIDTYQMQKAVGRAIAMTSPDETLTVVS
ADHSHAFAISGYPDITTDIYGYVYEVSADDKPYTTLAYANGPGWGSLRIDMNDLPEDNVNFL
QDAGVPFNSETHGGEDVPVYANGPMAYLLAGTYEQSYIPHVIMYATCI-
>Cte218695
DGMGMSTLTAARWHKAEAEGTKAVETMLQWDKWPASGMSKTYNVDRMTPDSAGTATAF
SCG-------------------------------------KSTGIVTTTRITHATPAASYAKSADRDWENDSDFPEELKAE
CKDIAYQLVHTNNIQVLMGGGRREFMSNNETDPEY-PELLGYRNDGRNLIMEWESENVQWA
FVDRQEDFDAVDPAQTDYLLGLFDHGHMQYEIDRSNDGEPSLAEMVEKAIRILSKNPNGFVLI
VEGGRIDHAHHATNAFRALSDTIALEEAVAKADQMTSIEDTLMVVTADHSHVFSIGGGPDINL
DIYKLAADVIGDGKPYTTLAYANGPGWYSLRVDMSEWSDDNIDFLQDAGVPMKSETHAGED
VAVFARGPMAYLLTGTYEQSFIPHVMMYAACI-
>Cte122379
---------------------------------LASAQVYTVDYITPDSAAAGTALLCGQKTHFGVIGLSQNAQYGN
CSSVDGNELKSILDEANTVGKWTGVVTTTRVTHATPATAYAHSVTCDWESDADIPKDQRDK
CKDIADQLITENHLRVVLGGGRSKFTPIDVEDGEI-RNATGNRLDQRNLIERMKSENMNAIYV
TKQSEFDAVDPENTEFLLGLFEPSHMKFEVDRANDGEPSISQMVDKAIQILKRGPKGYVLVVE
GGRIDEGHHLNNAYRANEDTIALSDAVSTAMDLNCENDTLVVVTADHGHVFSFGGYHMINE
DIY-------ADEKPYTLMNYANGRGWGQLRKDLRNLSEG-------------------------KGPSA----G------
----
>Cte124483
DGMGVTPLTAARWHQGQKSGSKAFETHLAMDLMPVIGHTKVYTVDYITPDSAATGTALLCG
QKTHFGVIGLSQDAQYSNCSSVDGNELNSILDEADAAGKWTGVVATTRVTHATPATAYAHS
VTRDWESDADIPEDQRLQCKDIADQLVTDNHLRVVLGGGRSKFTPIDVEDGEI-PNTFGHRLD
DRNLIEEWELKHQTARYVTRQSEFDDVDPENTEFLLGLFEASHMNFEVDRVNDGEPSISQMV
DKAIRILRRGPEGYVLVVEGGRIDHGHHFNNAYRANEDTVALSDAVSTALELTSEDDTLVVV

TADHGHVFSFGGYHMINEDIYDVVKTKQADTKPYTLMNYANGPGWNFLKTDARALVSRNP
MFMQDAAIPMQIETHGAGDVAVYARGPMAHLFAGTYEQSFIAHAAMYAACL-
>Cte149113
DGMGIPTITAGRIYKGQKEGNPGEETVLNFEAFPHTALAKTYNVNHQVPDSAGTGTAYLCGV
KANYATLGVTHKVEKDDCDAEDKNRVSSIAEWFNKDGRSTGIITTARLTHATPAAVYAKSSS
RYWESDKDLEASNEGKCKDIAAQLIEDNYIQVLMGGGRRNYWPKNKVDPQY-PTKNGTRVD
NRDLMKVWRDEGVTHADFYDKAGFDQVDPEKIDYMLGLFDYSHMQYELDRKQTNQPSLKE
MTEKAIKILNRNDKGYFLLVEAGRIDHAHHDALPKKALEDTVALDEAVKQALSMTDTEDTLI
VVTADHSHVFTQGGYPARGRNILGFVGPESTDGFPTPILNYAQGP--VVGRYDLSDVDMEADD
FTSPALIKMDYETHGGEDVAVYAQGPHSHLFKGTIEQNVIAHVMGYSACV-
>Cte172073
--MGPGTVTAARIHKGQLQGRPGEEGSLAFDRFPNVALSKTYNVDHQVTDSAATATAFLCGV
KANYGTLGVGPKVKRGDCNAKTESNITCFAELAQRAGKATGFVTTSRVTHATPAPLYARTA
DRMWEGDSELPEEAKGGCVDIAAQLLGPGNLNVVMGGGRKFLLPNTEPDPEQ-SQSRGLRED
GRNLVVEWMRRRFQAKYAYDQSSFDSIEPSETDYMLGLFEYSHMKYESERSMEGEPSLAEM
VQKAIQVMQANEKGFFLLVEGARIDHAHHSSTARRALEEVLSMEKAVLAAMQQVNLDETLI
VVTADHSHPLTISSYATRGNPILG------YDFMPYTTLSYANGPGYSSPRPNLTNVITDTYVYQQ
ASAVPSRIVSHDGTDVGIYATGPMAHLFHSTHEQHYIYHVMSYASCL-
>Cte27603

----------NEKGFFLMVEGGRIDMAHHGSRPRRALTDTLEFSQSVEMAVKMTSEEDTLIIVTADH
SHVMAFAGYHTKGSDVLGTGT---ADIMPYSKLSYTNGPGYSCRRGNLREVDTSHNDFVAQS
AAMLGSETHGGEDVAIYASGPMAHLLQG--EQHYIAHAAAYAACI-
>Cte39048
DGMGMSTLTAARWHKAEAEGTKAVETMLQWDKWPASGMSKTYNVDRMTPDSAGTATAF
SCGEKARYGTLGVNQYVKRGDCAAVETNQVQSMIHIA---

>Cte34391

---------------------------------AFRALSDTIALEEAVAKADQMTSIEDTLMVVTADHSHVFSIGGGPDI
NLDIY-------GDGKPYTTLAYANGPG---CKIDHSDYTTANIDFLQDAGVPMKSETHAGEDVAVF

ARGPMAYLLTGTYEQSFIPHVMMYAACI-

>Cte107229

DGMGVTPLTAARWHQGQKSGSKAYNTRLAMDLMPVVGLSKVSTQGHYVPIPI-----------------

---------C--S

W--

>Nemve162264

DGCDINTNTAGRILKGQLKGQVGEKGWLSYEEFPYTGLSKTYTTNRQGSDSAGTANAMFTG

VKTRSAMIGVNEEVVTNKCETTEDRKVDSILKLAEEAGMATGFITSMRLTHATPANLYAHSA

SRYWESDKEMVSRGYTSCKDMAQQLVDFGGIEVVMGGGRRSFLPNTTRDPEH-VNKTGERY

DGRNLIQEWLN---GSVYAWNKTQFDAVDPKTTKHFLGMFESSHMQFAIDRDEDGEPSIAEMV

EKAVSILKNNDKGFFLAVEAGLIDIGHHNGIARQALNEVVNLEEGVAKAVEMTDKEDTLIIST

ADHGHVFTLTGYPDINSPIFGLMKQDGLDNKPYTILGYMNGPGAKGVRANLTGVDTGAKDF

KQQALYIGYDEAHGSQDVGIYSRGPWAHLLTGVVEQNVIFHVMDHALCL-

>Nemve15380

DGMGISTVTAARILEGQMRGETGEENWLSFEKFPWTALSKTYNTDQQVADSAGTATAYLNG

VKARAGTIGVDETVVRGLCKTTEKAKVKSIITLAEEAGMSTGFVTTTRVTHASPAVLYAHSP

DRDWSSDRRYATKGDTACKDIALQLSEYGGIDVIFGGGRRDIMPXHETDPEY-PTEKGYRGD

GRNLIDEW---

>Nemve243830

--------------------------------LPFV--LLLLIE-------------------

---------------------------AQQLVDFGGIEVVYGGGRLAFLPNTTKDPEH-VNSTGERYDGRNLVQE

WTE---DSKYIWNKKQM--AELGNAKHVLGLFESSNMQYDAHREADGEPSITEMVESAIKILKN

NNKGFFLMVE-----------------------------------EETLIIVTADHGHTMTMAGYPERGNPILGLAKDG

GADKLPFTTLGYMNGPSAFGKRKNLTSTDTLSAKFQQQSLYNGNYESHGGQDV----------------

----

>CgiK1PG91

DGMGISTVTAARIYKGQQQNKTGEEAKLAFEEFPEIGLVKTYSVDEQVPDSAGTATAFLCGV

KAQSGVLGLDDTATYKNCSSSIGAEVDSILRMSKQKGKSVGVVTTARLTHATPGAAYSHSAS

RGWEGLSNMEGVE-PQCTDIAYQLVMNNDIDVLMGGGRRFFLSMNKTDPELNRTSSYQRWD

-LDLVDEWLRENVSHSYVWNNEGFNAINPQTTDYVLGLFESSHMQYEYQRDTGGEPSLAEM

TQKAIEILGRNDKGFFLLVEGGRIDHGHHASQAAVALSETVMFDKAVQVAKEITNDDDTLLV

VTADHSHAFMMAGYPSRGNDILGVVDT--GDDMPYTTLTYGNGPQ---QRRNLTGIDTTAFTFR

QSGVVPLGSETHAGEDVPVYASGPMSHLFVGTHEQNYITHVLAYASCV-

>CgiK1Q804

DGMGVSTVTAARIYGGQLKGRHGEESVLEFEKFPNVGLSKTYLCDRQVADSAATATAMLTG

VKTNWYNVGLTSKARMGQCQFPKESEIDTILQQSEKEGKSTGIVTTSRITHATPATAYAKVSH

RYWETDTDQKKYGGAGCQDIAQQLIYKNDIDVIFGGGRRGFYPNTTRDPVTN--SSYLRQDGI

NLIQEWKVSGQNYKYVENTEQLRSVDPATTDKVLGLFGSSNMAYELQRDKGGEPSLSEMTK

KAIQILQKNSKGYFLLVEGARIDHGHHDSKAKVALSETLMLDDAVRVATEMTD-DDTLMIVT

ADHSHVFNIAGYPKRGNDILGVVNPIPPDDKAYTTLLYGNGPGGDKPRQDPREVDTTADDYQ

QTSAIPLKSETHGGEDVAIYARGPWAHLIHGVHEQHYIYHVMSLASCV-

>CgiK1RFJ7

DGMGMSTVTAARILRGQLNGNSGEENMLEFEKFPHIGLSKVYSSDRQTPDSAATATALLCGV

KTNSQLIGVDDSAVVGNCSSQFGAEINCMNDWFEEQGRSMGIVTNTKITHATPAAGYAHAAS

RLWEGDVHMHGVQ-GGCKDIAHQLIYNNNIQVLFGGGRQYFIRNRQQDPQYGAYISFQRQD

DLDLIEIWGRRGYTPKYAWNKQQFDAINPDETDFALGLFNPQHMQYDLERDTGGEPSLAEM

TDKAIRILSHNTRGYFLMVEGGRIDHGHHNNTAKRALYETLAFEDAVKTALQMTNSSDTLIV

VTADHSHVFNIGGHSYRGNNILGVVNPIAPDGNAWTTLSYGNGPGYWGFRKDPNTVDTTHN

DYRQVSAVPLLYETHSAEDVPVYATGPMAHLFDGVHEQHYIAHALAYAACV-

>CgiK1R2C6

DGMGVSTVTGGRILKGQLENKTGEETVLSWEKFPNVALSKTYNQDHQTPDSAGTATAYLCG

VKTNMGTIGVDAQIVRGHCDTTQAAKITSILDWSLAEGKSVGVVTTTRVTHATPAGTYANTA

ERNWEGDSDMTHVT-GGCKDIALQLIEENDIQVIMGGGRRFFLPADATDPERGSNTAHGRLD

GRNLIDEWMNRHKSYRYVWNETDFNTVDPAQTDFLLGLFESSHMQYDIDRNDPGEPHIKDM

TEKAIQILSKNPKGYFLLVEGGKIDLAHHGSRPVRSLHDVVAMDLAVDKAASITNNQDTLIVV

TADHSHTFVISGYPQRGNGIFDLMSSRPADHNPYTTLLYGNGPGAHSTRADLRNVDTTSKHY

TYQSAVPLSAETHGGEDVAIFARGPMAHLLHGVREQNYIPHVMAYASCV-

>CgiK1P9Y9

--MGLSTINAARIYKGQKLGNTGEETILEYETFPNVALSKVYGSDSQVPESAQTATALLCGEKT

NFNVVGLKDSVGASNCSAGQEAEVKSIIRHAIEQGKSTGVVTTTRVTHATPAATYAHSPHRD

WESDADINATLHGDCMDIAQQLINSNDIQVVLGGGRRAFLPVTEPDPKSNLRGVNLRLDGRN

LVQRWELKNKKYKYVWRKQDFDAVDPNDTDYLLGLFSPSHMQYELERDTSGEPSLTEMTD

KAIKILSRNKKGFVLMVEGGRIDHAHHDTTAKKALVDAVQFEEAVKMAVTLTNKDDTLVVV

TADHSHPFSLTGYTNRGNPILGLVDPESTDDLPYTSLLYANGPGYTTPRQNLTEIRTDANNYM

QQSAVPLDSETHSGEDVGIYAMGPMSHLFHGVHEQHYVAHVIQYAACI-

>CgiK1PP56

DGLGVSTLNAARIYRGQKNNKTGEETVLEWEKFPYAAFSKIYGADKQIPDSAQTATAILGGV

KVNYKTVGVTDAVPGEGCDKGEAGKVVSVLRKALSEGKSTGVVTSTRLTHATPAATYAHSA

HRNWESDVDVIDEARGKCRDIAYQLIHNNDIQVLLGGGRRAFYPANYSDPETGRTDVNKRN
DTNNLVEDWLNKNRKHKFVWKKDDFDNIDVNNVDYVLGLFSPSHMAYEIERDKTGEPSLSE
MTSKAINILKRNDKGFFLLVEGGLIDIAHHDSLAKKALEETLQFEEAVKVAASMTNQEDTLLI
VTADHSHPFSLAGYTKRGNPVLGLVDEESPDKKPYTALVYGNGPGYKADRENLTGVDTAAD
NYIQQSAVPLEYETHSAEDVGIFSQGPMSHLFHGVHEQHYIAHVIQYAACI-

>CgiK1P3B0
DGMGLSTINAARIYKGQKLGNTGEETILEYETFPNVALSKVYGTDKQVPESAQTATALLCGE
KTNFNLLGLKDSVGTSDCSAGEEAEIDSIIRQAIEQGKSAGVVTTARLTHATPAATYAHSPDR
NWESDADIGPAN-GACKDIAQQLIDNNDIQVLLGGGRRAFLPVSEPDPETNAPGVNKRMDGR
NLADDWESKNNNYRYVWKKQDFDDVDPDEVDFLLGLFSPSHMQYELERDTSGEPSLAEMT
DKAIKILRRNKNGFALMVEGGRIDHAHHENKAKKSLEEAVQFEEAIKMAMSLIDQKDTLVVV
TADHSHPFSLTGYTNRGNPILGLVDPLGTDGLPYTSLLYGNGPGYTSPRQDLTGVDTETTDFI
QQSAVPLEFDTHSGEDVGIYALGPMSHLFHGVHEQHYVAHVIQYAACI-

>CgiK1QS35
DGMSISTVTASRILQGRLKAELGEGNYLAFEKFPNSGLIKTYCNDQTTPDSASTATAILCGVKT
NARTLGVKDSAVLGNCTSQAGNEVDSILDWFHADGRSTGIVTTARITHATPGAGYAHSADRV
WEGDSEMEGVT-GGCKDIAYQLVKENHIQVLFGGGRYYFLPNTTNDPKHGKNVKKQRRDGL
DLTK------------------------------VCLFNDGHLSFELDKDSDEEPSLAEMTEKAIRILRKNPKGFFLLV
EGARIDSAHTDNNAKRALYETLAFETAIRGALELVDTSNTLVSVTADHSHSLLISGYASRHNS
LFGYVDKVRPDGVLWTTLLYGTGPGYWGLRTNKTKQELESKDYIQAAASPRSSGTHDGQDV
AVYARGPMSHLLTGVHEQHYINYVMTYAACV-

>Pfu2110.1 01992.t1
DGMGVSTVTAARILKGQLRNRSGEESFLEFEKFPYVALSKTYVTDRQVSDSAATATAILSGIK
TKWQHIGVDGTNASEDCNV--DARVETILDWSQTEGKSTGIVTNSRITHATPAALYAHVPNRN
WESDTSLPNS--TKCRDIAYQLIHENDIEVLFGGGRSKFLPDTEADIEKGYTQKYHRKDGLNLI
HNWETRGGRYKFLWNNTDFDTLDPNNVDYVLGLFESNHMQYELERDRGGEPSLEQMTKKA
IQILQRNNNGFFLLVEGGRIDHAHHDSKAKKALYETLALDEAIKTAVELTKEEETLIVVTADH
SHVFNIAGYPNRGNDILGIVDPPKPDNKPYTTLLYANGPGWSEVRQDPRTVDTTDDNYIQISA
VPLSQETHGGEDVAIYARGPMSHLFHGVKEQHYTAHVMAYASCV-

>Pfu18722.1 09945.t1
---TYGTDRQVSDSAQTATAFLCGVKTRWGVVGLKDGVTIRDCP
SSSGQEINSILDLSMQEGKATGVVTTTRITHATPASAYAHAANRDWEADSNMNDVV-GGCSD
IAKQLVYDNDIQ--

>Pfu1455.1 18344.t1

DGMGVSTLTPARILKGQMNNKSGEEEHLTWDKFPHVAFSKTYNQDHQTSDSAGTATAILTG
VKTNMGMLGVDARTKEGDCASAQGAEIYSIGKWSLDQGKKVGVISTARVTHSTPGALYSHT
PHRDWEGDVDLPKTGAEQCTDIATQLVDNNDIS---------------------------------DWQNRKLKYK
FVWNATEFHKVDPQNTDYLLGLFESSHMQFEADRTDPGEPSLAEMTEKAIQILRKDSDGFFLF
VEGARIDHGHHETRAYKALRDTIAMEEAVAKAVEMTSEQDTLIVVTADHSHAFSMGGYPSR
GNPIMGLTDWDGDDNHNFTTLVYGNGPGYNNTRLNLTNGNQPNKEYRQMAAFPLGSETHG
GEDVGIFARGPWSHLFHGVHEQNYITHVMAFASCV-

>Pfu5766.1 22707.t1

E--IQSS--------------------------C-------------------------------PG
FAFVHNS-------TSVVVQANSGC-----------SLFLITSGDRFYY--
------VVLVPXLFESSHMQYEYARDKSGEPSLREMTEKAIQILSKNPKGYFLLVEG-KVDDSEGD
---DGLPYTTLVYGNG---YEQRKNITDIDTTDFG
YRQESLVPLYSETHGGEDVAVYARGPMAHLFVGTHEQSYIPHVMAYASCV-

>Pfu2917.1 25579.t1

DGMGISTITSARIYKGQKMGNPGEETVMEFEKFPNTALIKTYGTDRQVPDSAQTATAFLCGV
KTRWGVVGLKDGVTIRDCPSSSGQKINSILDLGMQEGKATGIVTTTRISHATPASSFSHVASRN
LEADSNMNGVT-GGCTDIAKQLVYDNDIQVIMGGGRRNFFPSNVPDIETGEDKVNKRNDSLD
LVQ--

>Pfu2917.1 25580.t1

DVAAFARGPWAHLFRGVKE----QHYIAHVMQYA----SCTYGTDRQVSDSAQTATAFLCGVKT
RWGVVGLKDGVTIRDCPSSSGQEINSILDLSMQEGKATGVVTTTRITHATPASAYAHAANRD
WEADSNMNDVV-GGCSDIAKQLVYDNDIQVIMGGGRRNFFPSNVPDIETGEDKVNKRNDSLD
LVQVWKDLNRRHSYVWKKSDFDAVNPENTDYLLGLFSASHMSYELERDSSGEPSLAEMTDK
AIKILRKDQDGFFLLVEGGRIDHAHHDSLGKKALEDTVAMEAAVKKAVELTNEDDTLIIVTA
DHSHPFSLTGYTGRGNNILGVVDTLATDKLNYTALLYANGPGGNDSRVGLTNEISEGNDYRQ
VAAIHLDYETHSGEDVAAFARGPWAHLFRGVQEQHYIAHVMQYASCV-

>Pfu808.1 27803.t1

DGMGVSTVTAARIYGGQKVNQSGEEHILSFEAFPEIGLIKTYNTDLQVPDSAGTDTAFLCGVK
SKAGTLGLNDHVIYSNCTSQRGAEVTSILDWSTAEGKSTGIVTTARLTHATPAAAYAHAARR
GWEGDTEMPTDA-QTCKDIAYQLVMENNIEV--
-----------------------------HVQLL----KG--------KVDDSEGD--
-------------DGLPYTTLVYGNG---YEQRKNITDIDTTDFGYRQESLVPLYSETHGGEDVAVYAR
GPMAHLFVGTHEQSYIPHVMAYASCV-

>Pfu24958.1 29917.t1

--------------------------------------VYSNDQQVPDSAATATAMMCGVKTNSMTLGVDDTVKFRNC

TSQQGKELDCMPNWAQNEGSV---

---LEFD----------

>Pfu909.1 31263.t1

DGMGLSTVTASRILKGQLNGKPGEEGLLEFEKFPNIGLVKVYSNDQQVPDSAATATAMMCG

VKTNSMTLGVDDT---VLFGGGR

EFFYPESLVDPVYGSYKSLQRKDGLNLVNVWGHRGYKARYAWNSRQFHSINPRTTHFALGL

FHPSHMSYELDRKKGGEPSLSEMTSKAIKILQRNPRGFFLLVESGRIDHAHHNNTAKRALYET

LMFEEAIKAAMALTNESDTLMVVTADHSHVFNIAGYPKRGSSILGVINPENPDKKPWTTLVY

GNGPGYWGFRINPLFIDTTNKNYRQVSAVPLSSETHSGEDVIVYARGPMSHLFHGVHEQHYIA

HVMAYASCV-

>Lan3559

DGLGVSTVTAARIHKGQLQGKTGEEGSLFFENFPYVGLSKTYNTDRMVSDSAGTATAILCGV

KTKFGLIGVDDRTSFGNCTSEEGASVDSILKWANAEGKSTGIITTTRITHATPASGFAHSASRY

WEADADLPADARGTCTDIAKQMIEGDFIKVILGGGRRQFYHRNESDPEY-PNVKGKRTD-RNL

INQWLQMNMRAEYVWNSTGFNSVEPADVDFLMGLFEPSHMQYEGERNHDMEPSLAEMTEK

AIKILKKNAKGFVLLVEGGRIDHGHHASLARHAVTDTIAMEAAVKKAAELTSANDTLTVVTA

DHSHVFNIGGYAGRGIPILGVNDAESDNGVPYTVLSYGNGPGYTAPREDPTTVNTSSWNYVY

QSAVLTASETHGGEEVPVYAQGPMSHLFDFVYEQSYIAHVMAYAACV-

>Lan4870

DGMGVSTLTAARIYKGQQAGRPGEEGTLAFESFPHAGLAKTYNTDRQTADSAGTATAFLCG

AKTKSGLIGLDDRAVRGNCSSEKGGAISSILKWSNSEGKSTGVVSTARVTHATPASGYAHSA

DRNWEADADMPESARTGCSDIAAQLVGEDFLNVILGGGRSKFLPNTTADPEW-PGKNGSRVD

GRNLIEDWSKPS-RARYVWNLDGFKAVDPIGTDYLLGLFEPSHMQYELDRNKAKEPSLAEMT

EKAIKMLRKNPKGFFLLVEGGRIDHGHHNSTAHKALADTIAMDKAVTKALELTDTEDTLIVV

TADHSHVFTIGGYASRGNNILGITDKDKDNGTAYTSLLYANGPGYEAPRPDPAPINTNDSGYQ

FQSAVQLDSETHGGEDVAIYATGPMSHLFHSLHEQHYIAHMMAYASCV-

>Lan25968

--MEPS

LAEMTEKAIKILKKNAKGFVLLVEGGRIDHGHHASLARHAVTDTIAMEAAVKKAAELTSAN

DTLTVVTADHSHVFNIGGYAGRGIPILGVNDAENDNGVPYTVLSYGNGPGYTAPREDPTTVN

TSSWNYVYQSAVLTASETHGGEEVPVYAQGPMSHLFDFVYEQSYIAHVMAYAACV-

>Lan26503

DGLGVSTVTAARIHKGQLQGKTGEEGSLFFENFPYVGLSKTYNTDRMVSDSAGTATAILCGV

KTKFGLIGVDDRASFGNCTSEEGASVDSILKWANAEGKSTGIITTTRITHATPASGFAHSASRY

WEADADLPADARGTCTDIAKQMIEGDFIKV----------------------------------SWR---------------------

-----------------------RSL-------------NKEGFMMF--

>Bgl XP 013068123.1

DGMGLSTITAGRIYQGQKKGKSGEENKLTFEQFPYLALSKTYNVDRQVPDSAGTAVAMATGI

KVNYGTLGLSIDVSQGNCSKTEENKLKTVLDYALEEGKSVGIVTTTRVTHATPAAMYAHSPD

RDWEGDSEMNGMANRHVKDIAYQLILENNINVILGGGRRYFMNSSTFDPVI-KTAQGRRNDG

LDLIQAWKNRNLKHQYVDTRQQLKDIDVASTDYLLGLFAPSHMTYEADRPSSVEPTLTEMTE

KAIQILKKNPKGYFLLIEGGRIDHSHHDNGAMRALEELLELDNTVSKVNQLTSEDDTLTVVTA

DHSHVFTIAGYPSRGNNILGLVDIMAPDKMPYLTLGYTNGP--FARRDNLTGVNTNTSTFKQG

AYIKMPNETHGGEDVIIYAKGPMAHLFQGTKEQSYIGHVMMYSACL-

>Bgl XP 013068121.1

DGMGLSTITAGRIYQGQKKGKSGEENKLTFEQFPYLALSKTYNVDRQVPDSAGTAVAMATGI

KVNYGTLGLSIDVSQGNCSKTEENKLKTVLDYALEEGKSVGIVTTTRVTHATPAAMYAHSPD

RDWEGDSEMNGMANRHVKDIAYQLILENNINVILGGGRRYFMNSSTFDPVI-KTAQGRRNDG

LDLIQAWKNRNLKHQYVDTRQQLKDIDVASTDYLLGLFAPSHMTYEADRPSSVEPTLTEMTE

KAIQILKKNPKGYFLLIEGGRIDHSHHDNGAMRALEELLELDNTVSKVNQLTSEDDTLTVVTA

DHSHVFTIAGYPSRGNNILGLVDIMAPDKMPYLTLGYTNGP--FARRDNLTGVNTNTSTFKQG

AYIKMPNETHGGEDVIIYAKGPMAHLFQGTKEQSYIGHVMMYSACL-

>Bgl XP 013093584.1

DGMGISTVTASS----------------------PCMTSYESDTHVSD--LI

TGLSTGIVTTSRVTHATPAAAYASTPQREWEGDVNMLSDEDSHVDDIAKQLIRNNDIKVILGG

GRRYFLDNSTEDPETHIVSPYHRQDGNNLIEEWKERNATYSYVWTRDQLMNLSSD-TEFLLGL

FNPSHMDF----DRVNEPSLAEMTEKAIEVLRKDQEGYFLLVEGARIDFGHHSNSAYTAITETLD

FNEAVEVAARLTDPEDTLIIVTADHSHAFNIQGYSSKGNDIL--

>Bgl XP 013086787.1

--MSYEADRPS

SVEPTLTEMTEKAIQILKKNPKGYFLLIEGGRIDHSHHENGAKRALEEVVEFDNAVAKVNELT

SPENTLTVVTADHSHVFAIAGYPTRGNNILGLVDSVSPDKMPYLTLGYLNGP--YSERVNLTG

VDTTTNNFRQPGCIQMSYETHGGEDVIIYGKG-------------------------

>Bgl XP 013077235.1

---KSVGIVTTTRVT

HATPAAMYAHSPDRDWEGDSEMNGMANRHVKDIAYQLILENNINVILGGGRRYFMNSSTFD

PVI-KTAQGRRNDGLDLIQAWKNRNLKHQYLDTRQQLKDIDVASTDYLLGLFAPSHMTYEAD

RPSSVEPTLSEMTEKAIQILKKNPKGYFLLIEGGRIDHSHHDNGAMRALEELLELDNTVSKVN

QLTSEDDTLTVVTADHSHVFTIAGYPSRGNNIL---

>Bgl XP 013076274.1

DGMGIPTVTAARILAGQMAGNSGEENKLSFDKFPYTGLSRTYNVDRQTTDSAASGTAYLTGV

KTNQGLLGLSGKAQRLNCSSAQDAHVDSILRWSISAGK---

--PSI-----------------QNFITL

WQ--

>Bgl XP 013068124.1

-------------------------------MDYNINVILGGGRQYFTNSSTADPVM-SSMPGLRNDGFDLIKAWKTRKV

QHDYVYTRQQLNALDASSIDYLLGLFAPSHMTYEADRPSSVEPTLTEMTAKAIQILKKNPKG

YFLLIEGGKIDHGHHENGAKKALEELLEFDNAVAKVTEMTSEDDTLTIVTADHSHVFAISGYP

TRGNNILGLVDNMDPDNMPFLTLGYLNGP--FTNRTNLTGVDTTASSFHQPGCIKMNLETHAG

EDVVIYARGPMAHLFQGTREQSYIGHVMMYSACL-

BMPR (supplementary figure S1)

>HsaBMPR2_Q13873

ELIGRGRYGAVYKGSLDERPVAVKVFSFANRQNFINEKNIYRVPLMEHDNIARFIVGDERVTD

GRMEYLLVMEYYPNGSLCKYLSLHTSDWVSSCRLAHSVTRGLAYLHTELPRGDHYKPAISH

RDLNSRNVLVKNDGTCVISDFGLSMRLTGNRLV--AISEVGTIRYMAPEVLEGAVNLRESALK

QVDMYALGLIYWEIFMRCTDLFESVPEYQMAFQTEVGNHPTFEDMQVLVSREKQRPKFPEA

WKNSLAVRSLKETIEDCWDQDAEARLTAQCAEERM

>HsaACVR2B_Q13705

EIKARGRFGCVWKAQLMNDFVAVKIFPLQDKQSWQSEREIFSTPGMKHENLLQFIAAEKRGS

NLEVELWLITAFHDKGSLTDYLKGNIITWNELCHVAETMSRGLSYLHEDVCRGEGHKPSIAH

RDFKSKNVLLKSDLTAVLADFGLAVRFEPGKPPGDTHGQVGTRRYMAPEVLEGAINFQRDAF

LRIDMYAMGLVLWELVSRCKAADGPVDEYMLPFEEEIGQHPSLEELQEVVVHKKMRPTIKD

HWLKHPGLAQLCVTIEECWDHDAEARLSAGCVEERV

>HsaACVR2A_P27037

EVKARGRFGCVWKAQLLNEYVAVKIFPIQDKQSWQNEYEVYSLPGMKHENILQFIGAEKRG
TSVDVDLWLITAFHEKGSLSDFLKANVVSWNELCHIAETMARGLAYLHEDILK-DGHKPAISH
RDIKSKNVLLKNNLTACIADFGLALKFEAGKSAGDTHGQVGTRRYMAPEVLEGAINFQRDAF
LRIDMYAMGLVLWELASRCTAADGPVDEYMLPFEEEIGQHPSLEDMQEVVVHKKKRPVLRD
YWQKHAGMAMLCETIEECWDHDAEARLSAGCVGERI
>MmuBMPR2_O35607
ELIGRGRYGAVYKGSLDERPVAVKVFSFANRQNFINEKNIYRVPLMEHDNIARFIVGDERLTD
GRMEYLLVMEYYPNGSLCKYLSLHTSDWVSSCRLAHSVTRGLAYLHTELPRGDHYKPAISH
RDLNSRNVLVKNDGACVISDFGLSMRLTGNRLV--AISEVGTIRYMAPEVLEGAVNLRESALK
QVDMYALGLIYWEVFMRCTDLFESVPDYQMAFQTEVGNHPTFEDMQVLVSREKQRPKFPEA
WKNSLAVRSLKETIEDCWDQDAEARLTAQCAEERM
>MmuACVR2A_P27038
-VKARGRFGCVWKAQLLNEYVAVKIFPIQDKQSWQNEYEVYSLPGMKHENILQFIGAEKRGT
SVDVDLWLITAFHEKGSLSDFLKANVVSWNELCHIAETMARGLAYLHEDILK-DGHKPAISHR
DIKSKNVLLKNNLTACIADFGLALKFEAGKSAGDTHGQVGTRRYMAPEVLEGAINFQRDAFL
RIDMYAMGLVLWELASRCTAADGPVDEYMLPFEEEIGQHPSLEDMQEVVVHKKKRPVLRDY
WQKHAGMAMLCETIEECWDHDAEARLSAGCVGERI
>MmuACVR2B_P27040
EIKARGRFGCVWKAQLMNDFVAVKIFPLQDKQSWQSEREIFSTPGMKHENLLQFIAAEKRGS
NLEVELWLITAFHDKGSLTDYLKGNIITWNELCHVAETMSRGLSYLHEDVCRGEGHKPSIAH
RDFKSKNVLLKSDLTAVLADFGLAVRFEPGKPPGDTHGQVGTRRYMAPEVLEGAINFQRDAF
LRIDMYAMGLVLWELVSRCKAADGPVDEYMLPFEEEIGQHPSLEELQEVVVHKKMRPTIKD
HWLKHPGLAQLCVTIEECWDHDAEARLSAGCVEER-
>DmePut_Q24468
EQKASGRFGDVWQAKLNNQDVAVKIFRMQEKESWTTEHDIYKLPRMRHPNILEFLGVEKHM
D--KPEYWLISTYQHNGSLCDYLKSHTISWPELCRIAESMANGLAHLHEEISKTDGLKPSIAHR
DFKSKNVLLKSDLTACIADFGLAMIFQPGKPCGDTHGQVGTRRYMAPEVLEGAINFNRDAFL
RIDVYACGLVLWEMVSRCDFA-GPVGEFQLPFEAELGLRPSLDEVQESVVMKKLRPRLLNSW
RAHPGLNVFCDTMEECWDHDAEARLSSSCVMERF
>DmeWit_M9PE88
GMLGSGKYGTVMKGLLHDQEVAVKIYPEEHHQYYVNERNIYALPLMECPALLSYFGYDERC
TDGRMEYQLVLSLAPLGCLQDWLIANTLTFSECCGMLRSITRGISHLHTELRLGDQHKPCVAH
RDINTRNVLVQADLSCCIADFGFALKVFGSKYETKSINEVGTLRYMAPELLEGAVNLRETSLK
QMDVYALGLVLWEVATRCSDFYQATPPYKAPYEQEVGSHPSFDQMQALVVRHKARPLFPTG
WGGGAAAKVVRDTCEDCWDHDADARLTSLCAEERM

>LgiBMPR2_172579
SVLNRGRYSEVWKGLLNDQEVAVKIYSANYKQHFTNEKNIYSLPFMEHDSLLQFYGSDERLS
DGNMQYMLILTYIPTGSLFSYLKHNTVDWSTLCKMCISFTRGLAHLHNDITKGDQFKPAVAH
RDFNSRNILVRSDLSCVVADLGFSMSAMGSKLIQTSLTDVGTLRYMAPELLDGAVNLREASL
KQIDIYALGLVLWEVATRCIDLYAPVPDYMLPYQAEAGIHPTFEQLQLLVCRNKTRPKFSEIW
KTNQAVRSLRETMDDCWDADADARLTVLCVEERL
>LgiACVR2_123928
ELRARGRFGAVWKAQLLSEFVAVKIFPLQDKLSWVSEHDIYVLPQMKHENILKYIAVEKRGE
SLNTELWLITEFHERGSLSDYLKSHTITWNELCKIAESMARGLAYLHDEISKDRLAKPAIAHRD
FKSKNVLLKSDLTACIGDFGLALKFEPGVSPGETHGLVGTRRYMAPEILEGAICFNRDAFLRID
MYACGLVIWELISRCTAHDGPIDDYVLPFEEEIGAHPSLEEMQEMVVTQKVRPTVKDHWLSH
PGLSALVSTVEECWDQDAEARLSAGCVQERI
>CteBMPR2_117843
HLIASGRFGTVWKGQLSSVPVAIKVFSPAQRQYFTNEQDILNLPHMTHVALPKFYGAGERESD
GIPEYLIVMEYIESGSLTEYLRTHTLDWIKLCRMMYSAAAGLAHLHADFTKADKSKPAIAHR
DFNSRNIMVKADLSCCLVDFGFATRIDGCHFYQTSLSDVGTVRYMSPEVLEGAVNLREASLK
QIDVYALGLVIWEMAMRCTDLFLTTPEYQLPFQAELGLHPSFEQMQVWVSRNKQRPPFPDIW
KTNQAIRALKETIEDCWDQDAEARLTAMCVEERA
>CteACVR2_94926
EVKARGRFGCVYKAQMGERVIAVKVFPLQDKQSWATEKDIYSLPQLNHANILHYIGAEKRG
ENLNMDLWLITQFHEQGSLYDYLKGNLVSWQQLLAICESMAKGLAYLHEDLTRTQQAKPAI
AHRDFKSKNVLIKSDLTACIADFGLALKFEGGVSVGETHGQVGTRRYMAPEVLEGAINFSRD
SFLRIDMYACGLVLWELISRCSAADGPVEEYRLPLEEDVGTHPSLEEMQDAVVSKKSRPGFK
DVWRKHPGLCTMCDTIEECWDHDPEARLSAGCVQERL
>Equ62837

---IASRCIELYAPVPDYQLPFQAEAG
PHPTFEEMQVLVVKYKTRPRX-------------------------------------

Hes (supplementary figure S2A)
>HsaHES1_Q14469
SKPIMEKRRRARINESLSQLKTLILDALKSSRHSKLEKADILEMTVKHLRNLQKYRAGFSECM
NEVTRFLSTCEGVNTEVRTRLLGHLANCM
>HsaHES2_Q9Y543
LKPLLEKRRRARINQSLSQLKGLILPLLRNSNCSKLEKADVLEMTVRFLQELPSYREGYSACV

ARLARVLPACRVLEPAVSARLLEHLWRRA
>HsaHES3_Q5TGS1
----MEKKRRARINVSLEQLKSLLEKYSHQIRKRKLEKADILELSVKYMRSLQ-----------------------
----
>HsaHES4_Q9HCC6
SKPVMEKRRRARINESLAQLKTLILDALKSSRHSKLEKADILEMTVRHLRSLRKYRAGFHECL
AEVNRFLAGCEGVPADVRSRLLGHLAACL
>HsaHES5_Q5TA89
-KPVVEKMRRDRINSSIEQLKLLLEQEFRHQPNSKLEKADILEMAVSYLKHSKDYSEGYSWCL
QEAVQFLASDT-------MKLLYHFQRP-
>HsaHES6_Q96HZ4
RKPLVEKKRRARINESLQELRLLLAGE----VQAKLENAEVLELTVRRVQGV-RFAAGYIQCMH
EVHTFVSTCQAIDATVAAELLNHLLESM
>HsaHES7_Q9BYE0
LKPLVEKRRRDRINRSLEELRLLLLERTDNLRNPKLEKAEILEFAVGYLRER-----------------------
----
>HsaHEY1_Q9Y5J3
RRGIIEKRRRDRINNSLSELRRLVPSAFEKQGSAKLEKAEILQMTVDHLKMLHYRSLGFRECL
AEVARYLSIIEGLDAPLRVRLVSHLNNYA
>HsaHESY2_Q9UBP5
RRGIIEKRRRDRINNSLSELRRLVPTAFEKQGSAKLEKAEILQMTVDHLKMLQ--SIGFRECLTE
VARYLSSVEGLDSPLRVRLVSHLSTCA
>MmuHES1_P35428
SKPIMEKRRRARINESLSQLKTLILDALKSSRHSKLEKADILEMTVKHLRNLQKYRAGFSECM
NEVTRFLSTCEGVNTEVRTRLLGHLANCM
>MmuHES2_O54792
LKPLLEKRRRARINESLSQLKGLVLPLLATSRSSKLEKADILEMTVRFLQE-QSYLEGYRACLA
RLARVLPACSVLEPAVSARLLEHLRQRT
>MmuHES3_Q61657
----MEKKRRARINVSLEQLRSLLERYSHQIRKRKLEKADILELSVKYMRSLQ------------------------
----
>MmuHES5_P70120
-KPVVEKMRRDRINSSIEQLKLLLEQEFRHQPNSKLEKADILEMAVSYLKHSKDYSEGYSWCL
QEAVQFLASDT-------MKLLYHFQRP-
>MmuHES6_Q9JHE6

RKPLVEKKRRARINESLQELRLLLAGE----VQAKLENAEVLELTVRRVQG--RFAAGYIQCMHE
VHTFVSTCQAIDATVSAELLNHLLESM
>MmuHES7_Q8BKT2
LKPLVEKRRRDRINRSLEELRLLLLERTDNLRNPKLEKAEILEFAVGYLRER----------------------
----
>MmuHEY1_Q9WV93
RRGIIEKRRRDRINNSLSELRRLVPSAFEKQGSAKLEKAEILQMTVDHLKMLHYRSLGFRECL
AEVARYLSIIEGLDAPLLVRLVSHLNNYA
>MmuHEY2_Q9QUS4
RRGIIEKRRRDRINNSLSELRRLVPTAFEKQGSAKLEKAEILQMTVDHLKMLQ--SIGFRECLTE
VARYLSSVEGLDPPLRVRLVSHLSTCA
>CteHes_223641
NKPLMEKRRRERINKCLDQLKAILMEVTKSSYYSKLEKADILEMTVKYLKNMKKYVAGFNE
CSSEVTKYLSNVDGLSTDVKGRMLSHLANCL
>CteHes_66159
SKPLMEKRRRARINASLHQLKVLVLDALKSARFSKLEKSDILELTVKHLKSIQRFHSGFSECAR
EVSRYLSSVDNFDESIRGRLLNHLNRCL
>CteHes_156236
KKPIIERKRRERINDSLNQLKALVLDALKESRYSKMEKADILEMTVRHLKVVQKYRAGYHEC
ATEVSRYMASMRGVDTDTQSRLLRHLSQKL
>CteHes_225285
RKPIIERRRRERINRCLDQIKSLVLKALQETKYEKMDKADILEMAVRHLLDNENYRTGFNRCT
SQVREFLNNNNNNNNEVQKRLLGHLQSPL
>CteHes_182542
RRGVIEKRRRDRINQSLGELRRLVPSAFEKQGSAKLEKAEILQMTVDHLKILSYRAIGFRECMS
EVSRYLVSMEGLDIPLRVRLGSHLQCYS
>CteHes_228069
RRGVVEKRRRDRINQSLGELRRLVPSAFEKQGSAKLEKAEILQMTVDHLKMLHYRSVGFREC
AAEVARYLVSVEGLDLPLRLRLMNHLQCYS
>CteHes_66277
IKHLVEKRRRGRINQCLEELRCLVLEAMKVQQYEKMEKADILEMAVQHMRHVRHFDSGFRA
CVHEIAAFLDSYPNLDEGMKQRLLTQL----
>CteHes_163301
IKHIVEKKRRGRINQCLDDLKCLVLDELRPDQYEKMEKADILEMTVRYLRQRK-----------------
----

>LgiHes_209713

NKPLMEKRRRARINSCLTQLKSLVLQAMKSSQFSKLEKADILELTLKHLRALQKYRAGFNEC
ANEVMRYMG-AGGVSDDIRSRLVGHLANCM

>LgiHes_74634

NKPLMEKRRRARINECLVELKSLVLQALKSTQYSKLEKADILEMTVKHLKLLQKYRAGFNEC
AAEVARYLDSVQGGSQEVKGRVLNHLSNCI

>LgiHes_116653

SKPIMEKKRRARINASLSELKSLLLEVIKGSRHSKMEKADILEMTVKHLRQLQNYRLGFEECA
QEVTRYLSQMEVCDVDLRSRILNHL----

>LgiHes_67089

NKPLVEKRRRARINDCLGELKTLVLQAMQSAQISRLEKADILEMTVKYLHHVQKYRAGFTEC
ASEVMRYMTNIQGINPNISNKLTGHLAGCL

>LgiHes_153818

NKPLIEKRRRARINECLMQLKNLVLKATSMARTGKLEKADILEMTVEYLKKINNYSAGYEKC
MEELVQFLDKSNGFNPEIKQKITKHCRNKL

>LgiHes_171867

RRGIIEKRRRDRINSSLTELRRLVPSAFEKQGSAKLEKAEILQMAVDHLKMLHYRTVGYRECA
AEVARYLVAVEGLDIPLRMRLLSHLQCYS

>LgiHes_174935

RKHVAERNRRAKINCYLEEIEKLITPSLQFTANTKLEKAEVLERTVEYIKQNK--------------------
----

>LgiHEY_97309

---VIEKRRRDRINNCLMELSQAVPSVFAKQSSGKLEKAEILEMTVEYLRTIQNYELGYNECMR
EVVHYMTDIEGLGLSRCVRILSYLQ---

>Equ35320

NKPLMEKRRRARINDSLTQLKSLVVQGSKSSQFNKLEKADILELTVKHLRSLQ--------------------
----

Notchless (supplementary figure S2B)

>HsaNLE1_Q9NVX2

MADVQRLLVQFQDEGGQLLGSPFDVPVDITPDRLQLVCNALLAQEDPLPLAFFVHDAEIVSSL
GKTLESQAVETEKVLDIIYQPQAIFRVRAVTRCTSSLEGHSEAVISVAFSPTGKYLASGSGDTT
VRFWDLSTETPHFTCKGHRHWVLSISWSPDGRKLASGCKNGQILLWDPSTGKQVGRTLAGH
SKWITGLSWEPLHANPECRYVASSSKDGSVRIWDTTAGRCERILTGHTQSVTCLRWGGDGLL
YSASQDRTIKVWRAHDGVLCRTLQGHGHWVNTMALSTDYALRTGAFEPAEASVNPQLQGS

LQELKERALSRYNLVRGQGPERLVSGSDDFTLFLWSPAEDKKPLTRMTGHQALINQVLFSPDS
RIVASASFDKSIKLWDGRTGKYLASLRGHVAAVYQIAWSADSRLLVSGSSDSTLKVWDVKA
QKLAMDLPGHADEVYAVDWSPDGQRVASGGKDKCLRIWRR
>MmuNLE1_Q8VEJ4
MADVQRLLVQFQDEGGQLLGSPFDVPVDITPDKLQLVCNALLAQEEPLPLAFYVHDAEIVSSL
GKTLESQSVETEKIVDIIYQPQAVFRVRAVTRCTSSLEGHSEAVISVAFSPTGKYLASGSGDTT
VRFWDLSTETPHFTCKGHRHWVLSISWSPDGKKLASGCKNGQVLLWDPSTGLQVGRTLTGH
SKWITGLSWEPLHMNPECRYVASSSKDGSVRVWDTTAGRCERILTGHTQSVTCLRWGGDGL
LYSASQDRTIKVWRAHDGVLCRTLQGHGHWVNTMALSTDYALRTGAFEPAEATVNAQLQG
SLKELKERASSRYNLVRGQGPERLVSGSDDFTLFLWSPAEDKKPLARMTGHQALINQVLFSP
DSRIVASASFDKSIKLWDGRTGKYLASLRGHVAAVYQIAWSADSRLLVSGSSDSTLKVWDVK
AQKLATDLPGHADEVYAVDWSPDGQRVASGGKDKCLRIWRR
>Nve_136836
-----RILAQFKSEDGELVGAPFDLPVDITPDKLELVCNAVLQKEETVPYSFFVNESEVIGSLQDTL
HKQDVETEKVVEIVYQPQAVFKVRAVTRCTSSIPGHTEAVISVAFSPDGRYLASGSGDTTVRF
WDVTTETPHFTCKGHMHWILHIAWSPDGKKLASGCKNGEIRIWDPATGKQMGKTLKGHLK
WITWLSWEPLHRNPDCRYLASSSKDSSVKIWDTATGSVDKTFSSHTQSVTCVKWGGEGLIYS
ASQDRTIKVWRAEDGVLCRTLQGHAHWVNHMALNTDYVLRTGAFEPSKGTTL--QTMSAET
LQSEASKRYKEAKGSKAERLVSGSDDFTLFLWEPEAKTKPIARMTGHQALINQVCFSPDGRLI
ASAAFDKSVKLWNGETGKFITSLRGHVNCVYQIAWSADCRLICSGSADSTLKVWDMKTKKL
LYDLPGHADEVYSVDWSPDGARVGSGGKDKVLKM---
>Lgi_209088
MSLGQRLLVQFKSEAGEVTGSPFDLPVNINVHKLELICNAILQKEEPTPYSFFVNDIEITESLEK
TLDKDQLETEKVLDIIYQPQSVFKVRAVTRCTGTIEGHADAVISVAFSPDGRYLASGSGDTTV
RFWDVNTETPQFTCKGHKHWILCIAWSPDGLKLASGCKNSQVCIWDPKSGQQIGKSLIGHKQ
WITWLAWKPLHLDPECRYLASASKDSTVRIWDTLKSECHLTLSGHLQCISCVKWGGSNLIYT
SSQDRTIKVYRAEDGVLCRTLQGHGHWVNTMALSTDYAMRTGWFDPKNATIVKEITDSAEE
LGKKAQARYDAAKGSEPERLVSGSDDFTLFLWTPETEKKSVARMTGHQQLINEVQFSPDTRL
IASASFDKSVKLWEGRSGKFLASLRGHVNRVYQVAWSADSRLLCSGSSDSTLKVWDILEKKL
LFDLPGHADEVYAVDWSPDGQRVASGGKDKVLKIWRS
>Cte_179885
MEQTRRILAQFQSETGEVLGAPFDLPMDITTEKMQLICNALLQKEEAIPYSFYVEEKEITESLE
KAVPSEMLESEAVLTIVYQPQAIFRVRAVTRCTSTIEGHAEAIISVQFSPDARFLASGSGDTTVR
FWDLSTETPHFTCKGHKHWVLCIEWSPDGNKLASGCKNGQICLWDPSTGKQIGRTLTGHKQ
WITWLTWKPIHLDHECRWLVSSSKDGSVKLWDTHLGQCKLTLGGHMQCVTCVRWGGSDLL

YTASQDRTIKVWRP-DGTLCRTLQGHGHWVNHLALSTGYALRTGAYDPSKATLIAESTMSGP
EKQETALKKYQASKGDEPERMVSCSDDFTMFLWQPEVEKKPICRMTGHQQAINAVHFSPDA
RVIASASFDKSVKLWDGRTGKFITTLRGHVQRVYQIAWSADSRLLCSGSADSTLKVWDMKA
KKFSVDLPGHIDEVYAVDWSPDGQRVASGGKDKVLKLWRK
>SpuNLE1_XP_011684200
MEEGQRILAQFVSEKGEAAGAPFDLPLDVSTEKLQLICNAILQQEDTVPYAFYVDEKEITSTLS
ESLSKEDQETEQVLSIVYQPQALFKVRAVTRCTSTIEGHAEAVISVAFSPDGRYLASGSGDTTV
RFWDVTTETPHHTCKGHKHWVLCIAWSPDGRRLASGCKNSQIIVWNPETGKQEGKVLTGHK
QWITWLAWQPFHLIHECRHLASASKDGNIKIWDVVRGACLRTLSGHLQSVTCIRWGGTDLIY
SASQDRTVKVWRASDGILCRTLQGHGHWVNTMALSTDYVMRTSWFDPVQATINYQISQSAE
ELSKIAQDRYDTVKGSEPERLVTGSDDFTLFLWQPEKEKTSVARMTGHMQLINDVAFSPDTR
LVASASFDKSIKLWNGHTGKFITNLPGHLKVVQYVSWSSDSRLLCSGSSDSTLKVWDVKTNK
MSGDLPGHADEVYAVDWSPDGQRVASGGKDKVLKIWRR
>DmeNLE_Q9VPR4
MLTPHTIQARLVSDTGEEAGPPIDLPAGITTQQLGLICNALLKNEEATPYLFFVGEDEIKKSLED
TLDLASVDTENVIDIVYQPQAVFKVRPVTRCTSSMPGHAEAVVSLNFSPDGAHLASGSGDTT
VRLWDLNTETPHFTCTGHKQWVLCVSWAPDGKRLASGCKAGSIIIWDPETGQQKGRPLSGH
KKHINCLAWEPYHRDPECRKLASASGDGDCRIWDVKLGQCLMNIAGHTNAVTAVRWGGAG
LIYTSSKDRTVKMWRAADGILCRTFSGHAHWVNNIALSTDYVLRTGPFHPVKDRSKSHLSLS
TEELQESALKRYQAVCPDEVESLVSCSDDNTLYLWR-NNQNKCVERMTGHQNVVNDVKYSP
DVKLIASASFDKSVRLWRASDGQYMATFRGHVQAVYTVAWSADSRLIVSGSKDSTLKVWSV
QTKKLAQELPGHADEVFGVDWAPDGSRVASGGKDKVIKLWAY
>Equ_25538

------------------DGFTGAFVATLRGHVNMVYQVAWSADSRLLVSGSKDSTLKVWKVQDKALM
LDLPGHSDEVFAVDWSPDGQRVASGGKDKVLKMWRA

Wnt (supplementary figure S3)

>Equ48603_Wnt5

----------------------------------VSGSCALKTCWQQLPTFREIGDRLRDRYDGATEKLTEAASSWSE
KTKDTT----NLL-------------------------KKIS-----------------------------YTWTN-----------------
>HsaWnt1_P04628

LLSRKQRRLIRQNPGILHSVSGGLQSAVRECKWQFRNRRWNCPTAPHLFGKIVNRGCRETAFI
FAITSAGVTHSVARSCSEGSIESCTCDYRRRGPGGPDWHWGGCSDNIDFGRLFGREFVDSGEK
GRDLRFLMNLHNNEAGRTTVFSEMRQECKCHGMSGSCTVRTCWMRLPTLRAVGDVLRDRF
DGASRLYGNRGLRLEPEDPAKPPSPHDLVYFEKSPNFCTYSGRLGTAGTAGRACNSSSPALDG
CELLCCGRGHRTRTQRVTERCNCTFHWCCHVSCRNCTHTRVLHECL
>HsaWnt2_P09544
GLVSSQRQLCHRHPDVMRAISQGVAEWTAECQHQFRQHRWNCNTLDSLFGRVLLRSSRESA
FVYAISSAGVVFAITRACSQGEVKSCSCDPK-KMGSAKSFDWGGCSDNIDYGIKFARAFVDAK
ERGKDARALMNLHNNRAGRKAVKRFLKQECKCHGVSGSCTLRTCWLAMADFRKTGDYLW
RKYNGAIQVMNQDGTGFTVANERKKPTKNDLVYFENSPDYCIRDREAGSLGTAGRVCNLTS
RGMDSCEVMCCGRGYDTSHVTRMTKCGCKFHWCCAVRCQDCLEALDVHTCK
>HsaWnt2B_Q93097
GLVSRQRQLCQRYPDIMRSVGEGAREWIRECQHQFRHHRWNCTTLDTVFGRVMLRSSREAA
FVYAISSAGVVHAITRACSQGELSVCSCDPYTRGRHHDDFDWGGCSDNIHYGVRFAKAFVDA
KEKLKDARALMNLHNNRCGRTAVRRFLKLECKCHGVSGSCTLRTCWRALSDFRRTGDYLRR
RYDGAVQVATQDGANFTAARQGRRATRTDLVYFDNSPDYCVLDKAAGSLGTAGRVCSKTS
KGTDGCEIMCCGRGYDTTRVTRVTQCECKFHWCCAVRCKECRNTVDVHTCK
>HsaWnt3A_P56704
GLVPKQLRFCRNYVEIMPSVAEGIKIGIQECQHQFRGRRWNCTTVHAIFGPVLDKATRESAFV
HAIASAGVAFAVTRSCAEGTAAICGCSSRHQGSPGKGWKWGGCSEDIEFGGMVSREFADARE
NRPDARSAMNRHNNEAGRQAIASHMHLKCKCHGLSGSCEVKTCWWSQPDFRAIGDFLKDK
YDSASEMRESRGVELRPRYTYKVPTERDLVYYEASPNFCEPNPETGSFGTRDRTCNVSSHGID
GCDLLCCGRGHNARAERRREKCRCVFHWCCYVSCQECTRVYDVHTCK
>HsaWnt3B_P56703
GLVPKQLRFCRNYIEIMPSVAEGVKLGIQECQHQFRGRRWNCTTIDAIFGPVLDKATRESAFV
HAIASAGVAFAVTRSCAEGTSTICGCDSHHKGPPGEGWKWGGCSEDADFGVLVSREFADAR
ENRPDARSAMNKHNNEAGRTTILDHMHLKCKCHGLSGSCEVKTCWWAQPDFRAIGDFLKD
KYDSASEMRESRGVELRAKYSLKPPTERDLVYYENSPNFCEPNPETGSFGTRDRTCNVTSHGI
DGCDLLCCGRGHNTRTEKRKEKCHCIFHWCCYVSCQECIRIYDVHTCK
>HsaWnt4_P56705
GLIQRQVQMCKRNLEVMDSVRRGAQLAIEECQYQFRNRRWNCSTLDPVFGKVVTQGTREAA
FVYAISSAGVAFAVTRACSSGELEKCGCDRTVHGVSPQGFQWSGCSDNIAYGVAFSQSFVDV
RERASSSRALMNLHNNEAGRKAILTHMRVECKCHGVSGSCEVKTCWRAVPPFRQVGHALKE
KFDGATEVPRRVGRALVPRNAQKPHTDEDLVYLEPSPDFCEQDMRSGVLGTRGRTCNKTSK
AIDGCELLCCGRGFHTAQVELAERCSCKFHWCCFVKCRQCQRLVELHTCR

>HsaWnt5_P41221
GLSQGQKKLCHLYQDHMQYIGEGAKTGIKECQYQFRHRRWNCSTVDSVFGRVMQIGSRETA
FTYAVSAAGVVNAMSRACREGELSTCGCSRAPKD-LPRDWLWGGCGDNIDYGYRFAKEFVD
ARERYESARILMNLHNNEAGRRTVYNLADVACKCHGVSGSCSLKTCWLQLADFRKVGDAL
KEKYDSAAAMLNSRG-KLVQVNSRNSPTTQDLVYIDPSPDYCVRNESTGSLGTQGRLCNKTS
EGMDGCELMCCGRGYDQFKTVQTERCHCKFHWCCYVKCKKCTEIVDQFVCK
>HsaWnt5B_Q9H1J7
GLSPGQRKLCQLYQEHMAYIGEGAKTGIKECQHQFRQRRWNCSTADSVFGRVMQIGSRETA
FTHAVSAAGVVNAISRACREGELSTCGCSRTPKD-LPRDWLWGGCGDNVEYGYRFAKEFVD
AREREEQGRVLMNLQNNEAGRRAVYKMADVACKCHGVSGSCSLKTCWLQLAEFRKVGDR
LKEKYDSAAAMVTRKG-RLELVNSRTQPTPEDLVYVDPSPDYCLRNESTGSLGTQGRLCNKT
SEGMDGCELMCCGRGYNQFKSVQVERCHCKFHWCCFVRCKKCTEIVDQYICK
>HsaWnt6_Q9Y6F9
RLAGRQAELCQAEPEVVAELARGARLGVRECQFQFRFRRWNCSSHS-AFGRILQQDIRETAFV
FAITAAGASHAVTQACSMGELLQCGCQAP-RGRAPPGWEWGGCGDDVDFGDEKSRLFMDA
RHKRGDIRALVQLHNNEAGRLAVRSHTRTECKCHGLSGSCALRTCWQKLPPFREVGARLLER
FHGASRMGTNDGKALLPAVRTKPPGRADLLYAADSPDFCAPNRRTGSPGTRGRACNSSAPDL
SGCDLLCCGRGHRQESVQLEENCLCRFHWCCVVQCHRCRVRKELSLCL
>HsaWnt7A_O00755
GLAPRQRAICQSRPDAIIVIGEGSQMGLDECQFQFRNGRWNCSALETVFGKELKVGSREAAFT
YAIIAAGVAHAITAACTQGNLSDCGCDKEKQGHRDEGWKWGGCSADIRYGIGFAKVFVDAR
EIKQNARTLMNLHNNEAGRKILEENMKLECKCHGVSGSCTTKTCWTTLPQFRELGYVLKDK
YNEVVRASRNK---LKIKKP-RKPMDTDLVYIEKSPNYCEEDPVTGSVGTQGRACNKTAPQASG
CDLMCCGRGYNTHQYARVWQCNCKFHWCCYVKCNTCSERTEMYTCK
>HsaWnt7B_P56706
GLAPRQRAICQSRPDAIIVIGEGSQMGLDECQFQFRNGRWNCSALETVFGKELKVGSREAAFT
YAIIAAGVAHAITAACTQGNLSDCGCDKEKQGHRDEGWKWGGCSADIRYGIGFAKVFVDAR
EIKQNARTLMNLHNNEAGRKILEENMKLECKCHGVSGSCTTKTCWTTLPQFRELGYVLKDK
YNEVVRASRNK---LKIKKP-RKPMDTDLVYIEKSPNYCEEDPVTGSVGTQGRACNKTAPQASG
CDLMCCGRGYNTHQYARVWQCNCKFHWCCYVKCNTCSERTEMYTCK
>HsaWnt8A_Q9H1J5
------------GPKYTTSVALGAQSGIEECKFQFAWERWNCPEN-QLSTHNLRSATRETSFIHAISSAG
VMYIITKNCSMGDFENCGCDGSNNGKTGGGWIWGGCSDNVEFGERISKLFVDSLEKGKDAR
ALMNLHNNRAGRLAVRATMKRTCKCHGISGSCSIQTCWLQLAEFREMGDYLKAKYDQLIED
LRAGNAEGHWPAEALPSAEAELIFLEESPDYCTCNSSLGIYGTEGRECLQNSRERRSCGRLCC

GLQVEERKTEVISSCNCKFQWCCTVKCDQCRHVVSKYYCA

>HsaWnt8B_Q93098

------------PKIYSSVAAGAQSGIEECKYQFAWDRWNCP--EQLSSHGLRSANRETAFVHAISSAG

VMYTLTRNCSLGDFDNCGCDDSRNGQLGGGWLWGGCSDNVGFGEAISKQFVDALETGQDA

RAAMNLHNNEAGRKAVKGTMKRTCKCHGVSGSCTTQTCWLQLPEFREVGAHLKEKYHAL

VDLQGAGNAAGRGIADTRSISTRELVHLEDSPDYCLENKTLGLLGTEGRECRGRARERRSCR

RLCCGLAVEERRAETVSSCNCKFHWCCAVRCEQCRRRVTKYFCS

>HsaWnt9A_O14904

KLERKQRRMCRRDPGVAETLVEAVSMSALECQFQFRFERWNC-TLE-RYRASLKRGFKETAF

LYAISSAGLTHALAKACSAGRMERCTCDEA-PDLENRAWQWGGCGDNLKYSSKFVKEFLGR

RS-SKDLRARVDFHNNLVGVKVIKAGVETTCKCHGVSGSCTVRTCWRQLAPFHEVGKHLKH

KYETALK-VGSTTGAISPPRGL--PRTPELVHLDDSPSFCLAGRF--SPGTAGRRCHREK----NCESI

CCGRGHNTQSRVVTRPCQCQVRWCCYVECRQCTQREEVYTCK

>HsaWnt9B_O14905

KLSRRQKQLCRREPGLAETLRDAAHLGLLECQFQFRHERWNCS----LEGRMLLKGFKETAFLY

AVSSAALTHTLARACSAGRMERCTCDDS-PGLESRAWQWGVCGDNLKYSTKFLSNFLGSKR

GNKDLRARADAHNTHVGIKAVKSGLRTTCKCHGVSGSCAVRTCWKQLSPFRETGQVLKLRY

DSVVSATNEALGRLELAPARTKPRSGDLVYMEDSPSFCRPSKY--SPGTAGRVCSREA----SCSS

LCCGRGYDTQSRLVAFSCHCQVQWCCYVECQQCVQEELVYTCK

>HsaWnt10A_Q9GZT5

GLSRRQMEVCVRHPDVAASAIQGIQIAIHECQHQFRDQRWNCSSLTIPYESPFSRGFRESAFAY

AIAAAGVVHAVSNACALGKLKACGCDASRRGDEEALWEWGGCSPDMGFGERFSKDFLDSR

EPHRDIHARMRLHNNRVGRQAVMENMRRKCKCHGTSGSCQLKTCWQVTPEFRTVGALLRS

RFHRTIRHNRNGGLEPGPAPSRRRASPADLVYFEKSPDFCEREPRLDSAGTVGRLCNKSSAGS

DGCGSMCCGRGHNILRQTRSERCHCRFHWCCFVVCEECRITEWVSVCK

>HsaWnt10B_O00744

GLSKRQLGLCLRNPDVTASALQGLHIAVHECQHQLRDQRWNCSALGLPHHSALKRGFRESAF

SFSMLAAGVMHAVATACSLGKLVSCGCEQDRRARGKSPWEWGGCNHDMDFGEKFSRDFLD

SREAPRDIQARMRIHNNRVGRQVVTENLKRKCKCHGTSGSCQFKTCWRAAPEFRAVGAALR

ERLGRIIDHNRNSGPRLRP---R--RLSGELVYFEKSPDFCERDPTMGSPGTRGRACNKTSRLLDG

CGSLCCGRGHNVLRQTRVERCHCRFHWCCYVLCDECKVTEWVNVCK

>HsaWnt11_O96014

GLVSAQVQLCRSNLELMHTVVHAAREVMKACRRAFADMRWNCSSI-PNYLLDLERGTRESA

FVYALSAAAISHAIARACTSGDLPGCSCGPV-PGEPPGGNRWGGCADNLSYGLLMGAKFSDA

PMKGSQANKLMRLHNSEVGRQALRASLEMKCKCHGVSGSCSIRTCWKGLQELQDVAADLK

TRYLSATKVHRPMGTRLVPDLDIRPVKDSELVYLQSSPDFCMKNEKVGSHGTQDRQCNKTSN
GSDSCDLMCCGRGYNPYTDRVVERCHCKYHWCCYVTCRRCERTVERYVCK

>HsaWnt16_Q9UBV4

PLNSRQKELCKRKPYLLPSIREGARLGIQECGSQFRHERWNCMITATLFGYELSSGTKETAFIY
AVMAAGLVHSVTRSCSAGNMTECSCDTTLNGGSASGWHWGGCSDDVQYGMWFSRKFLDF
PIGENKVLLAMNLHNNEAGRQAVAKLMSVDCRCHGVSGSCAVKTCWKTMSSFEKIGHLLK
DKYENSIQ-ISDK---TKRKDQRKIPIHKDLLYVNKSPNYCVEDKKLGIPGTQGRECNRTSEGAD
GCNLLCCGRGYNTHVVRHVERCECKFIWCCYVRCRRCESMTDVHTCK

>LgiWnt_V4BEM0

RLAGKQRAICRKEPEIVEEVAKGAKVALMECQYQFRTRKWNCTTLR-SLSKILRRDTREAAFV
YAITAAGVVYAVTEACSMGRLLQCTCDNNLRDIATDEWEWGGCGDNVDFGYQKSREFMDA
RRRRGDVTTLVQLHNNEAGRQAVRKYMRKECKCHGLSGSCTLKTCWRKMPLFRDVGNRLK
QKFDGGSKISSNDGKYLIPEGDTKPPSKEDLVYSEESPDFCRRNKKEGALGTRGRECDPTSMG
VGGCDLLCCGRNYSKKQVTIKENCNCRFMWCCEVICETCQKIKTETRCL

>LgiWnt_V3Z3G3

GMAPKQRAICRSRPDAIVSIGEGAKLGLTECQYQFRFMRWNCSTLSSMFGYESLGGTKEAAFI
YAMTSAGVSYAITQSCGLGSLPNCGCDKDKDGLAPQGWKWGGCSADIKHGLRLARKFMDA
REIAQNARSLMNLHNNRAGRKAVKDNMGTDCKCHGVSGSCTMKTCWTTLPPFRKIGDSLK
KRYKKKVV-LGRRATALKRAKRPRKPRRSHLVYLDKSPNYCDFDGKTGSLGTVGRKCNRTT
KDTDGCDLMCCGRGYNTHQYTRTWQCNCKFHWCCYVNCNKCSERTEEYTCK

>LgiWnt_V3ZRI8

GLSPGQIRFCRVYHDHMPSIGRGAQLGIHECQYQFRNRRWNCSTVDSVFGPVLNIGNREAGFT
HSISAAGVVYAISRACREGELSKCGCSRAPKD-LHRDWIWGGCGDNIEYGYKFAKAFVDMRE
KRDLSRMLMNVHNNEAGRRAVYNFAKVACKCHGVSGSCSLKTCWQHLPNFREIGNRLKDR
YDASTEVFNKRGTKLTRKVKKNKYTKNDIIFLDDSPDYCDKNAETGSVGTAGRECDRNSQGL
GGCGLLCCGRGYNTFKRKLIERCNCKFHWCCFVRCGSCERYVDVHICR

>LgiWnt_V4CRZ0

------------------------MGIDECQHQFRDRRWNCTTFNNVFGQILKIKSRETGYIYAILSAGIMYSVT
RACAKGDLDHCGCDTKVRRISTEEFEWGGCSDNIRYGSKFSKDFVDSKEMRISEDGLMNVW
NNGAGRKMVKDELELMCKCHGVSGSCSVKICWRKMKSFRAIGSALKGRFDGSVK-MDRRR
KRLKRTNKLKRPTKKDLVYLNESPDFCEHNLENGSVGTRGRECNKTSYGLDGCRLMCCGRG
YYTLIKEEKDDCDCKFYWCCRVECKKCTNVKEMHYCN

>LgiWnt_V4B8U0

GFVKRQKKLCRKNLEIMDNVKVGARVAIEECQSQFGNRRWNCSTANKLFGNVLKQGTREA
AFVHAISSAGVAHSVTRACSSGTLQKCGCDRTVRGRSPEGFEWSGCSDNIDYGMAFSKAFVD

AREKKNTGRALMNLHNNEAGRKAVDANMKVECKCHGVSGSCEMRTCWKAMPSFKKVGEI
LKEKFDGATEVHQVKGRNLVPLNPQKRHTESDLVYMVASPDFCEADKKTGSLGTHGRICNK
TSKAIDGCELLCCGRGYRTRIVTIKERCFCKFLWCCYVKCKECTRQIEEHTCL
>LgiWnt_V4AAU9
GLVGKQRKMCRHYPDVMVWLREGARLGVEECQYQFQDHRWNCSTLHSVFGKSMLKGTRE
AAFVYSISSAGVVYSITSACSKGELLHCACDPTKRGTSKDEFDWGGCSDSVRFGSRFSRMFVD
AKEKVSDARALMNLHNNRAGRRAVRRFRKLMCKCHGISGSCTLRTCWLAMEDFRRVGDFL
KRKYNGATQIMNQAGTGLIVANKNKHPTRSDLVYFESSPDYCIKDEESGSLGTEGRECIKGSL
GTGGCDIMCCGRGYDTKTIVKQEQCECKFHWCCLVKCKECSKTMDVQTCK
>LgiWnt_V4CP96
KLHRRQKRMCRRGKGIAESLLEATRLAVIECQYQFKHERWNCSLGQ-YRQNILRRGFSETSFL
YAISSAGLVHAFARACSKGVLDRCTCDES-KHLENHTWLWGGCGDNLRYGLKFTRKFLKRA
RKGKDVRAKVNQHNSRVGIKVVKENVNTTCKCHGVSGSCTVKTCWLQLSPFSKIGRTLKNK
YERKVLQTNQATLLVRRTNAERSPKRGNLLYMDESPSFCRRSRY--TPGTTGRTCDKDR----DC
ETMCCGRGYNVKHTTVVKACKCQVFWCCHVKCKQCLKNQEIYMCK
>LgiWnt_V4A3C5
GLVIQQKEVCQKNPESVYCIGEGARRGILECQKQFKFERWNCTLIGTVFGHVLSKGTREAAFI
YAILSAGVVHSVTQSCSAGNLTDCSCDMSKYGRENKGFQWGGCSDNVDYGLKFSRGFVDAP
EISRDIRNLMNLHNNEVGRQIVEKNMQLRCRCHGVSGSCAVRTCFRSLPNFRKVGLELKDKY
EKSVRLRRSRK-RLRRKDKRQGISKQELVFVHRSPNYCKEDIKRGIFGTRGRVCNRSSPTEESC
DLLCCGRGYNTQVVKYVERCHCKFFWCCYVKCKTCETMMDIHTCK
>LgiWnt_V4AL51
GLLPRQRRICKQHLEIMSTIVGASLLSVETCQNQFSDRRWNCSSISPSVPRDLSRGTREQAYVY
GIASAALTHSVARACSIGVTRKCSCGALPNTAPTGAFKWGGCGDDLHFGMALGRAFTDASL
KKSSKKAMMNRHNFAAGRKIVESSLTTACKCHGVSGSCSIKTCWKSLPDFDSIGATLKNRYA
LAVEKRKRKKKQLVPMKNKKTIRSDELIYYTKSPDYCSPDAKSGSIGTHNRLCDKTSRGSGG
CDVMCCGRGYDSFKMEVMERCECKYYWCCYVKCKTCVKTLNLSKCR
>LgiWnt_V4B9T1
PLTKKQKKLVTRNPGSIMAIAKGAKMAIDECKYQFKNRRWNCPTYGSIFGKILNTGCRETAFI
YAATSAAVSHSIARACSEGSIHTCSCDFEHKVPVGKDWEWSGCSDNARYGHKFSRRFVDVLE
KGRDFRYMMNLHNNEAGRVHVSSGMKQECKCHGMSGSCTIKTCWMRLPPFRNIGHILKDRF
DGASKLPGNTGFNFSPVNPNKRPGRRDLVYFEHSPTFCEKENMIGFEGTAGRECNSTSLGVNG
CDLMCCGRGYKSETFPVKERCHCTFHWCCQVKCQVCTRLKVRNTCL
>LgiWnt_V4AJ96
--SFAYAISSAGVTHQVSKACSMGKLKSCGCDM

SVYG-NQQNFEWGGCSHNINFGAKYASKFLDSKERSRDIHAQINLHNNRAGRLAIIRHVRKQC
KCHGMSGSCELKTCWKAAPDFRAVGTILKKKYDRTVDTNSANT--LIKRNG-RIPKHLELLFYE
KSPNFCDPNPLVDSPGTTGRLCNKTSGGINNCETLCCGRGYNTLRVKRTERCHCKFFWCCYV
TCKTCEYDEWVTVCK
>CteWntA_216606
------------------------MGIDECQHQFRDRRWNCTTYNDVFGKVLDLKTREKAYIYAVSSAGVMF
AVTTACAKGELHICDCDEKVRSQDTKGFIWGGCSHNVAFGDRFTREFVDSNENRFNDEGLM
NLWNNNAGRKAIRTSMKLLCKCHGVSGSCSAKICWKTMTGFRNIGSQLKDKFDGSVI-HHDK
KHRLKPMDRYKKPNKKDLVYLQESPDFCSSNTTIGSLGTQGRACNKTSYGLDGCSLMCCGR
GYQTTLITVVEDCNCKFVWCCNVVCDECIKREERHICN
>CteWnt1_110406
PLTRKQRRLVTRNPGTSIAIAKAARMAVDECQRQFSTRRWNCPVYSSIFGKVVRKGIRECAFI
YAIMSAALAHSIARSCAEGSIYTCTCGRHSRRLANSDWEWGGCSDNAEFGRKFSHDFIDVAE
KGRDLKCLMNLHNSEAGRTQVSSEMSKECKCHGMSGSCTVKTCWMKLPMFGRVGKVVKD
RFDGASQEQGNGAFNLIPINTNKPPEPKDLVYFERSPTFCTKDPSIGHTGTHGRPCNASSIGVE
GCDLLCCGRGYRSELYTARERCNCIFHWCCKVTCDTCTKTKVRHICL
>CteWnt2_96953
GLMSRQRLLCRQHPDVMMSVVFGAKKGVKQCQRQFRHHRWNCSVSHSVFGKLMLKGSKE
AAFVYAISSAGVVHAITRACSQGRLTNCACDPTKTGSSSDKFDWGGCSDNVRYGSHFARMF
VDAREKVKDARALMNLQNNRAGRRAVRRHMTLECKCHGVSGACTIRTCWLALQEFSRVGS
YLKTRYDSAKQVMNPMGTGLIATTSGKKTTRSDIVFFDESPDYCVQDPLAGSLGTADRECNH
TSKGPHGCDVMCCGRGYDTHVVRRMRKCDCKFHWCCYVKCRECEELVKVNTCR
>CteWnt4_181867
GLVKRQKKICKRNIEVMHAVKKGAVSAIDECQYQFQNRRWNCSTVDAVFGNVLNAGTREA
AFVHAISSAGVTYAVTKACSSGQVDKCGCDRSIRGKSPQGFEWAGCSDNVAYGSAFCGMFV
DARERRQSSRALMNLHNNEAGRLAVEENMKVQCKCHGVSGSCEMKTCWRGLPSFREVGA
MLKDRFDGASEVEQKVGRKLVPKNTQKPHETQDLVYLEASPDYCISDPETGSLGTSGRTCNR
SSKAIDGCELMCCGRGFNVKRRVVDERCHCKFHWCCYVKCQQCKKVVDEYVCR
>CteWnt5_156046
----------------MPAVSAGAKIALGECQQQFHSRRWNCSLMEAVFGPVLDTGSRESAFTYAIFAAG
VVHAVSRSCRDGQLTNCGCSRRPKT-LHRDWLWGGCGDNTDYGYRFAQGFVDIRERPELAR
TLMNLHNNEAGRRAVYSHTVVACKCHGVSGSCSLKTCWNQLAPFRGTGNRIKDAYDRGTE
VFNRQGTRLVQTNKRNKPTKEDLLYLAESPDYCEADPGIGSLGTQGRQCNKHSQGMDGCNL
MCCGRGYNTYKAKVSERCQCKFHWCCYVQCKTCERVVDINTCK
>CteWnt6_182518

KLGGKQHEMCRNEPEIVEEVANGAKMAISECQHQFRHRRWNCTTANRSIQRVLKHDTREAA
FVHSLTSAGVLYAVTQACSLGLLLQCMCDNSVMDRSTDKWEWAGCNEDVRFGSRKAAEFL
DIPPTRNDVQGRILLHNNKAGRASVAKYQQKICKCHGLSGSCELKTCVLKMPSFRDVGDRLK
ERFDGAYKSIANDGRNIIPDHDARRPSGENLVYLDESPSFCKPSRKQGSLGTLDRLCNPDTSTT
DSCDIMCCGRGYRSYKVVVQENCRCQFKWCCKVICQTCSRTLSIHRCN
>CteWnt7_112156
GLAPRQRTICLSHPDSMAAAGQGAKLAFEECSYQFRLHRWNCTITRSPLAAQHTIASKEAAYT
SAIRSAGVSYIITQACSQGSIMSCGCDKT-KGDTSNEWKWGGCSADIKYGLTFSRLFLDSKEVK
EDERALMNLHNNRAGRKAVKSQMDTQCKCLGVSGACTIKTCWTTLPGFRSIGDHLKQKFTK
AKQVQSSRTLHLKRLKSLRKPRKADLVYLKRSPSYCEKDEGIGSLGTTGRLCNRTANSYNNC
DLMCCGRGYNTHQYTRTWQCDCKFHWCCHVTCDECTELTEEYTCK
>CteWnt8_90169
-LNNFLKAGSKSYMALVDSVSSGTQLGLRECQEQFKWERWNCPLKSPFNARVADTVTKEISF
GQAITCAGVAHTITKNCSSGEFENCRCDESKKGKKGGTWKWGGCSDNLRFGERVSKLFFDD
RVAATDAMATVNLHNNEVGRTALKKTMELICKCHGVSGSCTTKTCWQHLSDFRSVGTFLKR
CYARIVD-FQNGALQATRRRDRELIRKTDLVYLQTSPDYCRVN---GSYATLGRQCVRSSHERK
SCHRLCCGLKVTDKVVEVTSSCKCKFKWCCEVACQQCRRKVELSTCT
>CteWnt9_222661
KLHPAQKKMCLSSLGISSTLLDSVRLSVVECQANFKFDRWNCSLGK-PRINMLNKGYKETAY
LHALTSSSLVHTFSRACAQGRLDRCTCDESFSASKNKAWLWGGCGDNIQYGMKFARRFLRW
MRKSLDLQATADSHNSDVGIRVVRNGINKTCKCHGVSGSCTVQTCWRQLANFHEVGSDLKS
KYDSVVVSANQSTQLTKRKNSQSNPKKGDLVFLEKSPNYCEPTAF--GHGTTGRVCDLNK----
NCDILCCGRGYNIHTRIVDKPCHCQVIWCCHVKCQRCSVREDIYTCK
>CteWnt10_110385
DLTGPQYALCLKHPDVMEVAVQGLKMAMEECQFQFREHRWNCTSMKSPYGNRLHKGYRE
TAFASAISAAGMSAQIALACAMGNLPACGCNPRM--TSTQQWVWKGCQHNVRFGDYFTRKF
WGSKKEATNVYSEMDVHNSRAGRMIWRENVRLHCKCHGMSGSCEVRTCWKAASSFRKVG
SIIKQKFEQTVDDNSSKRRRLKTRRRKRHIDNTDLVYFERSPNFCEPDKTLDSPGTVGRVCNSS
SLHIDSCDTLCCGRGYNTVRLTKIERCQCKFRWCCDVLCKKCLMTSWVTVCK
>CteWnt11_20087
GLVKQQIQICKRNMELMPVVAHATRQTTDVCQYLFAQYRWNCTSVRPKYLPDLTGGSREQA
YVHALSAAALAQTISKACTQGATTKCSCGRIPNEAPPSEFKWGGCGDDLRFGMIFSASFADSP
FLKRSKQAMMNLHNNNAGRKIISDSLVTDCKCHGVSGSCNIKTCWKALPDMRTVGTKIQER
YRLAVEVNKKVKKEFVPMDKKTSFSENELIYYTKSPDYCLPDGGLGSMGTRGRECEKTNDG
SIGCQSMCCGRGFTSQVVEVKHRCECKYFYCCYVECKTCTKKVEINRCR

>CteWnt16_149951

GLVYQQLRVCEENPHTMPCVSYGARVGIEECHHQFKKERWNCTTPDNLFGQILKRGTKETAF

MYAVTSAGVVHAVTKACSSGNLTDCTCDLSQQGTSGEGWKWGGCSDNVDYGMWFAETFV

DAPEKSKDIRSLMNLQNNAVGRQVINDQMNLKCRCHGVSGSCAVKTCWRTLTSFREAGNEL

KQKYENSISIEKSKR-KLRQRRQRTSNNGLDMVYIEDSPNYCRKNMKRGILGTKGRECERDPD

ARDSCNTLCCGRGYNTEVVRFVERCQCKFVWCCEVKCKICETITDKQTCK

Frizzled (supplementary figure S4)

>HsaFzd1_Q9UP38

CQPISIPLCTDIAYNQTIMPNLLGHTNQEDAGLEVHQFYPLVKVQCSAELKFFLCSMYAPVCT

VLEQALPPCRSLCERARQGCEALMNKFGFQWPDTLKCEKFPVHGA

>HsaFzd10_Q9ULW2

CQPIEIPMCKDIGYNMTRMPNLMGHENQREAAIQLHEFAPLVEYGCHGHLRFFLCSLYAPMC

TQVSTPIPACRVMCEQARLKCSPIMEQFNFKWPDSLDCRKLPNKND

>HsaFzd2_Q14332

CQPISIPLCTDIAYNQTIMPNLLGHTNQEDAGLEVHQFYPLVKVQCSPELRFFLCSMYAPVCTV

LEQAIPPCRSICERARQGCEALMNKFGFQWPERLRCEHFPRHGA

>HsaFzd3_Q9NPG1

CEPITLRMCQDLPYNTTFMPNLLNHYDQQTAALAMEPFHPMVNLDCSRDFRPFLCALYAPIC

MYGR-VTLPCRRLCQRAYSECSKLMEMFGVPWPEDMECSRFPDCDE

>HsaFzd4_Q9ULV1

CDPIRISMCQNLGYNVTKMPNLVGHELQTDAELQLTTFTPLIQYGCSSQLQFFLCSVYVPMCT

KINIPIGPCGGMCLSVKRRCEPVLKEFGFAWPESLNCSKFPPQND

>HsaFzd5_Q13467

CQEITVPMCRGIGYNLTHMPNQFNHDTQDEAGLEVHQFWPLVEIQCSPDLRFFLCSMYTPICL

DYHKPLPPCRSVCERAKAGCSPLMRQYGFAWPERMSCDRLPVLGR

>HsaFzd6_O60353

CEPITVPRCMKMAYNMTFFPNLMGHYDQSIAAVEMEHFLPLANLECSPNIETFLCKAFVPTCI

QIH-VVPPCRKLCEKVYSDCKKLIDTFGIRWPEELECDRLQYCDE

>HsaFzd7_O75084

CQPISIPLCTDIAYNQTILPNLLGHTNQEDAGLEVHQFYPLVKVQCSPELRFFLCSMYAPVCTV

LDQAIPPCRSLCERARQGCEALMNKFGFQWPERLRCENFPVHGA

>HsaFzd8_Q9H461

CQEITVPLCKGIGYNYTYMPNQFNHDTQDEAGLEVHQFWPLVEIQCSPDLKFFLCSMYTPICL

DYKKPLPPCRSVCERAKAGCAPLMRQYGFAWPDRMRCDRLPEQGN

>HsaFzd9_O00144

CQAVEIPMCRGIGYNLTRMPNLLGHTSQGEAAAELAEFAPLVQYGCHSHLRFFLCSLYAPMC
TQVSTPIPACRPMCEQARLRCAPIMEQFNFGWPDSLDCARLPTRND

>MmuFzd1_O70421

CQPISIPLCTDIAYNQTIMPNLLGHTNQEDAGLEVHQFYPLVKVQCSAELKFFLCSMYAPVCT
VLEQALPPCRSLCERARQGCEALMNKFGFQWPDTLKCEKFPVHGA

>MmuFzd10_Q8BKG4

CQPVEIPMCKDIGYNTTRMPNLMGHENQREAAIQLHEFAPLVEYGCHSHLRFFLCSLYAPMC
TQVSTPIPACRVMCEQARLKCSPIMEQFKFRWPDSLDCSKLPNKND

>MmuFzd2_Q9JIP6

CQPISIPLCTDIAYNQTIMPNLLGHTNQEDAGLEVHQFYPLVKVQCSPELRFFLCSMYAPVCTV
LEQAIPPCRSICERARQGCEALMNKFGFQWPERLRCEHFPRHGA

>MmuFzd3_Q61086

CEPITLRMCQDLPYNTTFMPNLLNHYDQQTAALAMEPFHPMVNLDCSRDFRPFLCALYAPIC
MYGR-VTLPCRRLCQRAYSECSKLMEMFGVPWPEDMECSRFPDCDE

>MmuFzd4_Q61088

CDPIRIAMCQNLGYNVTKMPNLVGHELQTDAELQLTTFTPLIQYGCSSQLQFFLCSVYVPMCT
KINIPIGPCGGMCLSVKRRCEPVLREFGFAWPDTLNCSKFPPQND

>MmuFzd5_Q9EQD0

CQEITVPMCRGIGYNLTHMPNQFNHDTQDEAGLEVHQFWPLVEIHCSPDLRFFLCSMYTPICL
DYHKPLPPCRSVCERAKAGCSPLMRQYGFAWPERMSCDRLPVLG-

>MmuFzd6_Q61089

CEPITVPRCMKMTYNMTFFPNLMGHYDQGIAAVEMGHFLHLANLECSPNIEMFLCQAFIPTC
TQIH-VVLPCRKLCEKIVSDCKKLMDTFGIRWPEELECNRLPHCDD

>MmuFzd7_Q61090

CQPISIPLCTDIAYNQTILPNLLGHTNQEDAGLEVHQFYPLVKVQCSPELRFFLCSMYAPVCTV
LDQAIPPCRSLCERARQGCEALMNKFGFQWPERLRCENFPVHGA

>MmuFzd8_Q61091

CQEITVPLCKGIGYNYTYMPNQFNHDTQDEAGLEVHQFWPLVEIQCSPDLKFFLCSMYTPICL
DYKKPLPPCRSVCERAKAGCAPLMRQYGFAWPDRMRCDRLPEQGN

>MmuFzd9_Q9R216

CQAMEIPMCRGIGYNLTRMPNLLGHTSQGEAAAQLAEFSPLVQYGCHSHLRFFLCSLYAPMC
TQVSTPIPACRPMCEQARLRCAPIMEQFNFGWPDSLDCARLPTRND

>DmeFzd_P18537

CEPITISICKNIPYNMTIMPNLIGHTKQEEAGLEVHQFAPLVKIGCSDDLQLFLCSLYVPVCTILE

RPIPPCRSLCESARV-CEKLMKTYNFNWPENLECSKFPVHGG

>DmeFzd2_Q9VVX3

CEEITIPMCRGIGYNMTSFPNEMNHETQDEAGLEVHQFWPLVEIKCSPDLKFFLCSMYTPICLD

YHKPLPVCRSVCERARSGCAPIMQQYSFEWPERMACEHLPLHGD

>DmeFzd3_O77438

CQPIAVSACQGLGYNMTALPNLAGHTNQLEAELQIAKLVPLIESGCSRRARFLLCSSLFPLCTD

VPRPVAACKLLCETVRGECMELMEL----WPSFLNCDGLPQPEK

>DmeFzd4_Q9NBW1

CETIRIEMCRKIGYNETSMPNLVGNEMQTDVEYTLQTFAPLIEYDCSSQLKLFLCAAYVPMCT

KAPVAIGPCRSLCESVRIRCHPVLQGFGFPWPPALDCDKFPRENN

>LgiFzd1_139359

CEPITIPLCKDIQYNETIMPNILNHQKQDDAGLEVHQFFPLVKVQCSTKLKFFLCTMYVPVCTV

LEDAIPPCRPLCNQARDGCESLMNKFGFEWPESLKCEKLPVSG-

>LgiFzd8_200873

CLEISIPMCKGIGYNYTYMPNQFNHETQEEAGLEVHQFWPLVEIQCSPDLLFFLCSIYAPICMN

YKKHLPACRSVCERAKSGCAPLMRQYGFAWPERMNCESLPEYGD

>LgiFzd4_201339

CEPIRFEMCKGLGYNVTGMPNLVAHTDQQDAGLQLQTFTPLIQYGCSKQLKFFLCSVYVPMC

TKVMDPIGPCRPMCESVRSRCQPVLNEFGYPWPAALNCSKFPARND

>LgiFzd10_228923

CEPITIPMCKDMKYNTTRMPNLVGHENQKDAAIQVHEFLPLVQFGCSRLLKFFLCSLYAPMC

TLVDGIIPACRSMCLKVKAKCEPVLTKFAFPWPKMLSCDNLPEKSN

>CteFzd5_140195

CQEITIPMCRGIGYNLTHMPNQFNHDTQEEAGLEVHQFWPLVEIQCSPDLKFFLCSMYAPICM

NYHKPLPACRSVCERAKSGCAPLMRQYGFAWPERMRCDSLPEYGD

>CteFzd4_166935

CEPITVDKCHNLPYNVTGMPNLVGHANQADAELQFNTFTPLIQYECSKALRFFLCSVYFPMCT

KVSKPIGPCRPLCEHVQLKCRPVLRQFGFPWPNALNCSKFPIRND

>CteFzd10_176783

CERITIPMCRDMRYNMTRMPNLIGHTSQKDAEQQIREFIPLVQIRCSKLVKFFLCSLYAPMCTQ

VDEVVPACRSMCLEVKSKCEPIMTRFSFNWPSVLDCSKLPLKSD

>Equ31832

VKRFCVEACTI--HDKT--AKPINHTTQLEVYRHLERLWPYMDQSCSKNVRVMACATYL-QCH-

-GKAQGPC-------------------------------------

>Equ34280

-----------------------------------DALISTNCHLHLKFLICGVYSPFCV--NEFTFPCREICEEIRQACEP
HYRRLYHEMP----CHRSSSVTG

Hedgehog (supplementary figure S5)

>HsaSHH_Q15465
CGPGRGFGKRRHPKKLTPLAYKQFIPNVAEKTLGASGRYEGKISRNSERFKELTPNYNPDIIFK
DEENTGADRLMTQRCKDKLNALAISVMNQWPGVKLRVTEGWDEDGHHSEESLHYEGRAVD
ITTSDRDRSKYGMLARLAVEAGFDWVYYESKAHIHCSAENSVAAKSGGCFPGSATVHLEQG
GTKLVKDLSPGDRVLAADDQGRLLYSDFLTFLDRDDGAKKVFYVIETREPRERLLLTAAHLL
FVAPHNDSGGALGRALFASRVRPGQRVYVRDGDRRLLPAAVHSVTLEEAAGAYAPLTAQGT
ILINRVLASCYAVIEEHSWAHRAFAPFRLAHALLAALAPGAGATAGIHWYSQLLYQIGTWLL
D-
>HsaDHH_O43323
CGPGRGVGRRRYARKLVPLLYKQFVPGVPERTLGASGPAEGRVARGSERFRDLVPNYNPDIIF
KDEENSGADRLMTERCKERVNALAIAVMNMWPGVRLRVTEGWDEDGHHAQDSLHYEGRA
LDITTSDRDRNKYGLLARLAVEAGFDWVYYESRNHVHVSADNSLAVRAGGCFPGNATVRL
WSGERKGLRELHRGDWVLAADASGRVVPTPVLLFLDRDLQRRASFVAVETEWPPRKLLLTP
WHLVFAARGPAPAPGDFAPVFARRLRAGDSVLA-PGGDALRPARVARVAREEAVGVFAPLT
AHGTLLVNDVLASCYAVLESHQWAHRAFAPLRLLHALGALLPGGAVQPTGMHWYSRLLYR
LAEELLG-
>HsaIHH_Q14623
CGPGRVVGRRRPPRKLVPLAYKQFSPNVPEKTLGASGRYEGKIARSSERFKELTPNYNPDIIFK
DEENTGADRLMTQRCKDRLNSLAISVMNQWPGVKLRVTEGWDEDGHHSEESLHYEGRAVD
ITTSDRDRNKYGLLARLAVEAGFDWVYYESKAHVHCSSEHSAAAKTGGCFPAGAQVRLESG
ARVALSAVRPGDRVLAMGEDGSPTFSDVLIFLDREPHRLRAFQVIETQDPPRRLALTPAHLLF
TADNHTEPAARFRATFASHVQPGQYVLV-AGVPGLQPARVAAVSTHVALGAYAPLTKHGTL
VVEDVVASCFAAVADHHLAQLAFWPLRLFHSLAWGSWT---PGEGVHWYPQLLYRLGRLLLE
-
>MmuSHH_Q62226
CGPGRGFGKRRHPKKLTPLAYKQFIPNVAEKTLGASGRYEGKITRNSERFKELTPNYNPDIIFK
DEENTGADRLMTQRCKDKLNALAISVMNQWPGVKLRVTEGWDEDGHHSEESLHYEGRAVD
ITTSDRDRSKYGMLARLAVEAGFDWVYYESKAHIHCSAENSVAAKSGGCFPGSATVHLEQG
GTKLVKDLRPGDRVLAADDQGRLLYSDFLTFLDRDEGAKKVFYVIETLEPRERLLLTAAHLL
FVAPHNDSGPTPGSALFASRVRPGQRVYVRGGDRRLLPAAVHSVTLEEEAGAYAPLTAHGTI
LINRVLASCYAVIEEHSWAHRAFAPFRLAHALLAALAPGAEPTAGIHWYSQLLYHIGTWLLD-

>MmuDHH_Q61488
CGPGRGVGRRRYVRKLVPLLYKQFVPSMPERTLGASGPAEGRVTRGSERFRDLVPNYNPDIIF
KDEENSGADRLMTERCKERVNALAIAVMNMWPGVRLRVTEGWDEDGHHAQDSLHYEGRA
LDITTSDRDRNKYGLLARLAVEAGFDWVYYESRNHIHVSADNSLAVRAGGCFPGNATVRLR
SGERKGLRELHRGDWVLAADAAGRVVPTPVLLFLDRDLQRRASFVAVETERPPRKLLLTPW
HLVFAARGPAPAPGDFAPVFARRLRAGDSVLA-PGGDALQPARVARVAREEAVGVFAPLTAH
GTLLVNDVLASCYAVLESHQWAHRAFAPLRLLHALGALLPGGAVQPTGMHWYSRLLYRLA
EELMG-
>MmuIHH_P97812
CGPGRVVGRRRPPRKLVPLAYKQFSPNVPEKTLGASGRYEGKIARSSERFKELTPNYNPDIIFK
DEENTGADRLMTQRCKDRLNSLAISVMNQWPGVKLRVTEGWDEDGHHSEESLHYEGRAVD
ITTSDRDRNKYGLLARLAVEAGFDWVYYESKAHVHCSSEHSAAAKTGGCFPAGAQVRLENG
ERVALSAVKPGDRVLAMGEDGTPTFSDVLIFLDREPNRLRAFQVIETQDPPRRLALTPAHLLFI
ADNHTEPAAHFRATFASHVQPGQYVLV-SGVPGLQPARVAAVSTHVALGSYAPLTRHGTLVV
EDVVASCFAAVADHHLAQLAFWPLRLFPSLAWGSWT---PSEGVHWYPQMLYRLGRLLLE-
>DmeHh_Q02936
CGPGRG-LGRHRARNLYPLVLKQTIPNLSEYTNSASGPLEGVIRRDSPKFKDLVPNYNRDILFR
DEEGTGADRLMSKRCKEKLNVLAYSVMNEWPGIRLLVTESWDEDYHHGQESLHYEGRAVTI
ATSDRDQSKYGMLARLAVEAGFDWVSYVSRRHIYCSSDSSISSHVHGCFTPESTALLESGVRK
PLGELSIGDRVLSMTANGQAVYSEVILFMDRNLEQMQNFVQLHTDGG-AVLTVTPAHLVSV
WQPESQKL---TFVFADRIEEKNQVLVDVETGELRPQRVVKVGSVRSKGVVAPLTREGTIVVNS
VAASCYAVINSQSLAHWGLAPMRLLSTLEAWLPASAQQQNGIHWYANALYKVKDYVLP-
>CteHh_129621
CGPGRASGRRRGARKMTPLVFKQHVPNISENTLGASGLNEGRITRDDPRFKDLVENYNPDVV
FKDEEGTGADRIMSQRCKDKINTLAISVMNQWPGVKLKVTEAWDEDGFHAKDSLHYEGRA
VDITTDDRDRSKYGMLARLAVEAGFDWVYYENRGHIHCSSDSSITAKTGGCFSADDTVKRV
DGSSLPIQHLRIGDAIQASTDNGDVVYSPVILFLHREENAVASFVTLKTEGG-RSLTLSPSHLIH
TAEHGE--------IYASDVKIGQHLLALNNNRSLDKDPVVAMTTQYRRGVFAPLTAIGTIVVNDIS
SSCYAHVQSHAFAHAFLAPVRWHYQVLPV---SDSPQEGVHWYVQLLYDISTYVLP-
>LgiHh_121665
CGPGRGSGSRRRPRKRTPLVFKQHVPNVSENTLGASGISDGKIRRNSEKFKNLVKNENPDIVF
KNEEGDGSDYLMSRRCQDKLNSLAVSVMNNWKDVRLRVTEAWDDSNSHAKDSLHYEGRA
VDITTSDRDRSKYGMLARLAVEAGFDWVYYESRGHIHCSSDSSVAIKIGGCFPPTGSVQTLHG
-WKTMGQLTVGDKVLSINSAGQLEYSPVIAFIDRNDLEFEKYLTLHTEDD-TDITLTSKHLIYAS
GTNSSNFESYDVVYADDIMEGDHVLISSEKGAISPTRVVTISEKTLQGVYAPLTVNGNIVVDG

VVVSCYAVVSDANLAHAVFAPMRGLHYLSQYVPWENAPQNGVHWYAKMLYNIGSTFLN-
>CgiHh_K1R6F4
CASPRGSGRRHRTGNRTPLVYKQHVPNVSENTLGASGLAEGRISKEDPRFKKLVTNDNPDIIF
RDEEGDGTDRIMTQRCKDKLKILAISVMNTWQGVKLRVTEAWDDDGHHAKDSLHYEGRAV
DITTSDKDRAKYGMLAKLAVEAGFDWVYFESRGHIHCSSDSSAAIKIGGCFPPLATVYAENK
GTLNMADLRIGDKVLSLKADGKLGFSEGDLFVG--

>Equ36336
--GMHAESS
LHYEGRAVDITTSDRDRSKYGMLARLAVEAGFDWVYYESRGQIHCS-------------------------------
----

>Equ48577
-----------RPRKLTPLVFKQHVPNVSEHTLGASGLAEGQSNVATPSSR----------------------------------
--TSFAIITRILF--

>Equ24052

--KFVQ------------LSFIDALK---------------
---RNDDAELNTRKSCLYSNSFHQGVYAPLTLEGTIVVDNVATSC
YAFVD-HHLSHAVFSPLRGYHVIKLCSVIFQHSENGVHLYARFLYEFAVNVC--

Ferritin (supplementary figure S6)
>Dsp_A3ZU64
AQQLQSSLVDQIDLALQAKQAHWNVRGLGFRAVHLHLDEIIESTREASDEIAERIAAIGVAAT
VAQPFPAGI-I-SVQEAIRRISDQMATCSTSLRLRIKTIGD-DDLISQDLLIGICRTLEKQLWMLES
Q---AADA
>Dsp_Q9KWH3
KAVLNQAVADLSKAASIVHQVHWYMRGSGFLYLHPKMDELMDALNGHLDEISERLITIGGL
KEFDEET----TWDSITDHLKRLVQVYDYLSSLYQVGLDVTDEEDDAVSNDIFTAAQTEAQKTI
WMLQAELGQAPGL
>Dsp_Q0P891
TKQLLQMQADAHHLWVKFHNYHWNVKGLQFFSIHEYTEKAYEEMAELFDSCAERVLQLGE
QKVLMENAKSPKDCFTPLEVIELIKQDYEYLLAEFKKLNEAAEKESDTTTAAFAQENIAKYEK

SLWMIGATLQGACKM

>Bfl_P0ABD3

INYLNKLLGNELVAINQYFLHARMFKNWGLKRLNDVEYHESIDEMKHADRYIERILFLEGDL

GKLNI-------GDVEEMLRSDLALELDGAKNLREAIGYADSVHDYVSRDMMIEILRDEEGHIDW

LETELDLIQKM

>Bfl_P9WPQ9

LRLLNEQLTSELTAINQYFLHSKMQDNWGFTELAAHTRAESFDEMRHAEEITDRILLLDGRIG

SLRI-------GTLREQFEADLAIEYDVLNRLKPGIVMCREKQDTTSAVLLEKIVADEEEHIDYLET

QLELMDKL

>Bfl_Q59738

IEFLNAALRSELTAISQYWVHFRLQEDWGLAKMAKKSREESIEEMGHADKIIARILFLEGKLD

PLRI-------GGPRETLECDLAGEHDALKLYREARDYCAEVGDIVSKNIFESLITDEEGHVDFLET

QISLYDRL

>FtnA_P0A998

IEKLNEQMNLELYSSLLYQQMSAWCSYHTFEGAAAFLRRHAQEEMTHMQRLFDYLTDTGNL

PRINTVESPFAEYSSLDELFQETYKHEQLITQKINELAHAAMTNQDYPTFNFLQWYVSEQHEE

EKLFKSIIDKLSLA

>FtnA_P52093

IKLLNEQVNKEMNSSNLYMSMSSWCYTHSLDGAGLFLFDHAAEEYEHAKKLIVFLNENNVP

VQLTSISAPEHKFEGLTQIFQKAYEHEQHISESINNIVDHAIKGKDHATFNFLQWYVSEQHEEE

VLFKDILDKIELI

>FtnA_Q2FWZ8

LEALNDQMNHEYFAAHAYMAMAAYCDKESYEGFANFFIQQAKEERFHGQKIYNYINDRGA

HAEFRAVSAPKIDFSSILETFKDSLSQEQEVTRRFYNLSEIARQDKDYATISFLNWFLDEQVEEE

SMFETHINYLTRI

>Equ15651

EAGVNRQINMELYASYSYLSMAYYFDDVALPGFSKFFKKSSDEEREHAQKLMKYQNKRGGR

IVLQDIKKPSVSGGVVWR---

>Equ15652

--LDLHKLCTGHEDAQ

MADFLSEFLEEQVRSIKEIGDHITNLKRV

>NfuFerrt_BAK40157

EAGINRQVNMELYACYTYQSMAFYFEDVALPGFSKFFKSSSDEEREHAKKLMKYQNKRGGR

VVLQDIKKPEREWGSGLEAMQVALQLEKSVNQSLLDLHAVAEKHNDSQMQDFLGEFLKEQ

VDSIKEISDYVTQLKRV

>HasFerrt_DW986406

EAGINRQINMELYASYTYQSIGFYFDDVALPGFSKYFKKAAEEEREHAEKLMKYQNTRGGCI
VLQDIKKPDREWGSPLESMQVALALEKNVNQALLDLHAVANKHNDAQMCDFLSEYLEEQV
KAIKEISDHITNLKRV

>Lgi_183389

EAGINRQVNMELYASYTYQSMAFYFDDVALPGFSKFFKSSSDEEREHAEKLMKYQNKRGGR
VVLQDIKKPEREWGSGLEAMQVALQLEKSVNQSLLDLHAVAEKHNDSQMQDFLGEYLKEQ
VDSIKEISDYVTQLKRV

>Lgi_207566

EAGINRQINLELYASYVYQSMAFHFDDVNLPGFFKFFKKQGDEEREHAELLMKYQNTRGGRI
VLNNVQKPEQEWGTGLEAMQVSLQLEKKVNQSLLDLHKIADTHDDAQMTDFIGKFLDEQVE
SIKEISNYIASLKRV

>Cte_182078

EAGVNKQINLELYASYVYQSMAFYFDDVALKGFHEFFKKSSDEERGHAEKLMAYQNKRGG
RIVLQPIQKPEREWVSGLEAMKAALALEKNVNQALLDLHKLADGHGDTQMADFLSEYLEEQ
VDAIKEISGHVTNLKRV

>Cte_19905

EALINKQINMEMHANYVYTSMAYYFDDVALSGFARFFRKAAEEEREHAERLMKYQNTRGG
RVVLQDIQKPEQEWGTGLDAMLFSLDMEKRVNQSLLDLESTALAHADPELADFISEYLHEQV
AAIKGICCHITNLRRC

>Cgi_K1RB56

RKGLSKMISDCFNLYYQYLSMANYFGDVALPGFQKFFTTSAERELTRAKDMMEYMNKRGG
TLDLLPIKKPVTDFSDGRSALDLARRLSRKLNARALVNHNIAVSKRDPNLKLFYETIIDGQVD
VIKELGDLITRLDLM

>Cgi_K1R0W0

----------------QRKTSSQYFADVSLPGFSKFFSEASKEERSHAEKLMEYINKRGGDVQMKEIKKS
MRSWFNGLLAMEDTLVLERYVNEELLKIH--SGHDDDAHLSHVLHDFLDEQVNAIKEIGDKIS
QLKRA

>Cgi_Q70MM3

EAGINRQINMELYACYTYQSMAYYFDDVALPGFSKFFKNSSDEEREHAEKLMKYQNKRGGR
VVLQDIKKPDREWGTGLDAMQVALQLEKTVNQSLLDLHKVADSHQDAQMCDFLTHYLEEQ
VNAIKEISDHITQLKRV

>Cgi_K1QHW8

EAGINRQINMELYASYTYQSMALYFDDVALPGFHKFFKHSSDEEREHAEKLMKYQNKRGGRI
VLQDIKKPDREWGTGLDAMQIALQLEKSVNQSLLDLHKLADGHRDAQMCDFISEFLEEQVN

AIKEISDHVTQLKRV
>Cgi_K1QAG9_a
EAGINRQINMELYASYTYQSMALYFDDVALPGFHKFFKHSSDEEREHAEKLMKYQNKRGGRI
VLQDIKKPDREWGTGLDAMQIALQLEKSVNQSLLDLHKLADGHRDAQALYF--------------------
-----
>Cgi_K1QAG9_b
------------------AQALYFDDVALPGFHKFFKHSSDEEREHAEKLMKYQNKRGGRIVLQDIKKPD
REWGTGLDAMQIALQLEKSVNQSLLDLHKLADGHRDAQMCDFISEFLEEQVNAIKEISDHVT
QLKRV
>HsaFRIL_P02792
EAAVNSLVNLYLQASYTYLSLGFYFDDVALEGVSHFFRELAEEKREGYERLLKMQNQRGGR
ALFQDIKKPAEEWGKTPDAMKAAMALEKKLNQALLDLHALGSARTDPHLCDFLTHFLDEEV
KLIKKMGDHLTNLHRL
>HsaFRIH_P02794
EAAINRQINLELYASYVYLSMSYYFDDVALKNFAKYFLHQSHEEREHAEKLMKLQNQRGGRI
FLQDIKKPDCDWESGLNAMECALHLEKNVNQSLLELHKLATDKNDPHLCDFITHYLNEQVK
AIKELGDHVTNLRKM
>MmuFRIL1_P29391
EAAVNRLVNLHLRASYTYLSLGFFFDDVALEGVGHFFRELAEEKREGAERLLEFQNDRGGRA
LFQDVQKPSQEWGKTQEAMEAALAMEKNLNQALLDLHALGSARTDPHLCDFLSHYLDKEV
KLIKKMGNHLTNLRRV
>MmuFRIH_P09528
EAAINRQINLELYASYVYLSMSCYFDDVALKNFAKYFLHQSHEEREHAEKLMKLQNQRGGRI
FLQDIKKPDRDWESGLNAMECALHLEKSVNQSLLELHKLATDKNDPHLCDFITYYLSEQVKSI
KELGDHVTNLRKM
>DmeFer1HCH_Q7KRU8
IKGMRNQIQEEINASYQYLAMGAYFSTVNRPGFAEHFFKAAKEEREHGSKLVEYLSMRGQLT
DLINVPTVAKEWTDGAAALSDALDLEIKVTKSIRKLIQTCENKNHYHLVDYLGVYLEEQLHG
QRELAGKLTTLKKM
>DmeFer2LCH_Q9VA83
EPEIQSYINANLAKSYDYLLLATHFNQKNRPGFQKLYQGLSDRSFEDSIALIKQVTRRGGIVDF
NTRHESSGTLEDELHSLALALDTEKQLATGATHVHSRATHARDPELAHYFENFLGKQAESVR
KLSGYANDLAKL

Tyrosinase (supplementary figure S7)

>Equ11340

PNKYDAIALLHVLYAFAHLGDGFLGWHRLYMNMFEAAMQEVSVCLPYWDTSLELGDPALS

NLWTPEFMGTPNGPVVDGPFANWNTPGGPLIRNVDSRVDLVTATNIRNILTRTGYEQITF------

----

>Equ11343

PNRYDAIALFHVTGVRAHGGAGFFGWHRLYLNMYETALQEISVCLPYWDNSLDLDDITRSNI

WTPAFMGTPNGPVVDGPFANWVTPRAQLIRNFGSDGEPMTVTTVQDILTRSRYEDITSQTRY

DLEFHHGAVHMLVGGAMTQLNASFDPIFFLLHAYIDYIGSA----

>Equ14143

PNVHDAYSYLHHPEVNAHGGPAFLPYHRVFIFLYEKLLRIYGISLCYWDTTLEPENILWSSSW

TPELFGNVQGHSTASP------ETGP-------SGRAPRKD-----------DQMT---RTKWSC-------WTPSTLT

GAS-----------------WGRWRRQ

>Equ24617

--LIRNVGVDGELMTVTTF

QNILTRSSYQDIIFRPRYNLEIHHGGIHVFVGGAMSQLDAAFDPVFFQVHAFIDYKFEQFRX-

>Equ32293

SNQF------------RSRWSRVLGWHRLYITLFETALQEVSVCLPYWDVSLDLEDPTLSNLWTPAFM

GTPNGPV---

>Equ33769

----------HILSASATIRPGVIPWR--LP---------------------

------------------------HVW--GVMSQLDASFDPIFFLLHAYIDYLWELFRTK

>Lgi_V3ZAB2_SMP

PNMYDAVAMFHVGDASAHGGPGFLGWHRMYLVMYERALQSKGVCIPYIDNTIELGD-DGSY

LWSDEFLGTPNGVVTSGPFANWNTPIGELTRNVGNQAFPMDKDILNDIMSRGRIEDIVSELEH

DIEYHHGSYHIHVGGLMESIDASFDPVFFMHHAYIDYVWEQFRQK

>Cgi_K1PS92_SMPs

PNVYDALAGVHQATKVSRFGCGFLGFNRIFLLRLEMAMRQVTITIPYWASTLELNDPTVSVV

WSPEFFGNGYNFVETGPFGNWTDGTG-ISRNLNHIGGVLTESDIERVLERNTHHDIF-PRTKDF

ANINYNVHGYVGGTMNRLDAAYDPVFIFHYAYIDHLFEKAREN

>Pmarg-Tyrosinase1_SMP

MSRYDTIAGLHRQAIAAHMGANFLGWHRLYLDMFEMALQETDVVLCYWDSTLDGTSQVNT

VSFSAELFGNGRGVVINGPFRFWRLPGGTLQRFIARPGSSLTRPVVDLIATDPRINTNSQ-RGQG

FPDEHNNTHVWVGGVMQVVSSPQDPVFWFHHTYVDYVWELFRQK

>MgaCL4852Contig2_SMP

PNKYDSFAIMHQGAVGAHDGPNFVSWHRYFLVLFENALREKRVTLPYWDSRADMDNKEDSI
LFTEIFLGNSKGVVYSGPFAFWSTPTKPLRRELGVVGSPVHPERLKAVFTKRYHREILRSSYAN
LESHHDNVHRWVGGQMSSILSPMDPVFWLLHCFVDYLWEKFRER
>Villosa_30664_Tyrosinase_SMPs
NGEYDTFARIHSGPNLFHDGPNFLGWHRIYLAYFEEAVRRHSLSLPYWDYTLDLSDPTQSVL
WSATFLGNGDGVVWSGPFSGWVVNGSPLIRNTGHQGALMSKQDVDTVLTRCDTSEITFTGIY
NLEIYHNRVHNWVGGNMEMLDAAFDPAFFLHHAFVDYVWELFRLR
>Nve_A7SSF6
IKTYDKLTHLHPKFFDVHNKKNFFPWHRWFILNFENFIRKIRVTVPYWDWSQA-------VSGNRI
FRRSHIRDNGVGKFKYV--IDGPPKRAIKEAGIWLSRDNPCNSMYLPKASHVYGKNFFKFEDIH
DEFHNAVGGTMMLACSSNAPEFWFHHGFLDKLWSDWQNR
>Nve_A7RQY2
KSEYDRITKYHSDLFNVHILEYFFPWHRWYLLLFENLLRKIRVTVPYWDWSRARLWRGSYLH
DVWSPGPHGIGGVTNGPLRKDSWELPA-----WHEQPWLSRENDCYKERLPNSHEVRDEKFVK
FEDMHNDFHNAIGGVMSLDASANAPEFWFHHGYIDKVWSDWQKR
>Nve_A7RXW5
KDDFEKLLIEHSRLPSHHMPQIFFPWHRWYLSKIESFLKMIRVTIPYWQWTAQWASGPQGLG
GNG---------VQDGIFRVGNWH--P-----VKGGGCLKRQNKTCHLPDEADKALE-KDFLTFERFHN
RFHDCVGRLMHFHVASDTPEFPLHHAFIDKIWDMWEKK
>Nve_A7RKE4
KKEYDTIVRVHREHRTIHSARYFLPWHRQFLLNLENQMRKIRLTIPYWDWTLTWSPQDYGIG
GNG---------VLDGPFREGSWTFGK-------GGKVLTRNSGVLPNKVTVQRALQ-KRFTDFETFHHF
VRCAIGGTACSLDSARSPEFLLITAYIDKLWGDWQAR
>Nve_A7RWK6
KRSYDQLIATHNNFKNIHNLMYFLIWQRYFLIWQQYFLQQEERYFLIWQQERYWQQVRYFLI
WQQ---------QQERYFLIWQ-QIAP-------SDKYLTVH-----------NRVP-NDFTKFEMLHNNMHCRI
GGTMCSTSSANAPEFLLHHGFTDKLWADWQKI
>Hsa_P40126
CSVYDFFVWLH-----SHQGPAFVTWHRYHLLCLERDLQRLSFALPYWNFATGCDVCTDQLFG
AARPDDPTLIS-RNSRFSSWETHLVTNQMGRNS-MKLPTLKDIRDCLSLQKF-----NSTFSFRNLH
NLVHSFLNGTNAPHSAANDPIFVVLHSFTDAIFDEWMKR
>Hsa_P17643
ISIYNYFVWTHVGQESSHEGPAFLTWHRYHLLRLEKDMQEMSFSLPYWNFATGCDICTDDLM
GSRSNFDSTLIS-PNSVFSQWRVTLGTNPAGNVARPMVQRLPEPQDVAQCLEV----GNSTNSFR
NLHNLAHLFLNGTGGTHLSPNDPIFVLLHTFTDAVFDEWLRR

>Hsa_P14679

INIYDLFVWMHG-SEIAHEAPAFLPWHRLFLLRWEQEIQKLNFTIPYWDWRDACDICTDEYMG

GQHPTNPNLLS-PASFFSSWQISLCNRRNPGNHDKSRTPRLPSSADVEFCLS----LAANFSFRNM

HNALHIYMNGTMSVQGSANDPIFLLHHAFVDSIFEQWLRR

>Mmu_P07147

ISVYNYFVWTHTGQESSHEGPAFLTWHRYHLLQLERDMQEMSFSLPYWNFATGCDVCTDDL

MGSRSNFDSTLIS-PNSVFSQWRVTLGTNPAGNVGRPAVQRLPEPQDVTQCLEV----RNSTDSF

RNLHNLAHLFLNGTGGTHLSPNDPIFVLLHTFTDAVFDEWLRR

>Mmu_Q91XK0

INIYDLFVWMHG-SEIAHEAPGFLPWHRLFLLLWEQEIRELNFTVPYWDWRDACDICTDEYLG

GRHPENPNLLS-PASFFSSWQIVLCDLRNPGNHDKAKTPRLPSSADVEFCLS----LTANFSFRNM

HNALHIFMNGTMSVQGSANDPIFLLHHAFVDSIFEQWLRR

>Cte_R7VC10

LSVYDYLVHVHRTQSLAHNGPGFLTWHRLYLMMMEREMGRVFFALPYWNWSGACSVCTD

DLAGSINWSDPLRTLSTGSPFSRWESPRGPVIRDPEASPFLPPMEDVIFTLSQATFDLYP-TSRYS

FRNMHIQVHSFFRGTISVSSSPDDPLFFFHHTYIDKLFEVWLRM

>Cte_R7UGP0

ITSLDELNTTRKCETT-----------RRYIMQYKMRLRVA----ITWGVAIGTCQATDQQSSDQHAMPI

----VRDSPCPPCALNLSPTVEAEQQDEEEVKEEEEEVVAEEEYKPNIVQPDSIYFHTVHRNPPRL

YG-------KIKDPVFYTSHSNVDRLKILWLNS

>Cte_R7THS2

SSIYDTLTFLHVQFILAHNGPGFPTWHRRFLLEFERELQRAQMGIPYWDWTDHCSVCTNDLV

GE-MVLDDPEGIDRGSDFWNWTMSLPPWRLPTGSAPNLPILEDISFALNAEVYDAEP-SPANSF

RNLHGQGHTFFSGSVNVPISPSDPLFYLHHAYVDKILEVWLRN

>Cte_R7UB97

QSKYDLFVRHHARQAPAYFGPGFYGWHREMLFRFENAIREMLVSLPYWDSNLDDDLATQSI

LWTPDFFGNGQGEVNTCPFAHWTTQTGPIHRNLNASASLMSNRAISAILKAPSFREIT---DAEF

QSHSLAVHKWIGGSMRLHLSPADPIFFLHYAFVDCLWDQFRTQ

>Cte_R7UA24

--------------------------------MLMRMRIANIGLTLGGLRIARSELTDFLAADT-ANND----YRNIPV---

---------------TEPRRDTDDLV------PNLVHPDVIYFHTVHREPPTLFG-------RIKDPLFYTSHSNVD

RIKILWINS

>Lgi_V4A5X3

PNSFWSIASYHMPFKDQHSNVLFPTWHRFYCLRMEQALQTVDVALHYWDTTSEPILVQPTVT

IDGPSIPNPLLDFYVKPEGYTTMIVNPTKIREVQPTELLQENIYFINNGRYPG----QDDYNNFSNP

HNDIHLSIGGEMGNEVAAFDPIFFSHHSNIDRVFWIWQRK

>Lgi_V4AN59

PNVLDAFSFLHRNEVNAHGGVGFLPFHRVFLYLYEKLLRQYGVSLCFWDPTLE--DYEQSEIW

GDRLFGTARGIVSEGFAANWMTPVGPLIREVGRTGRTLNEKDIENVLSKKRLGEISFRLSENV

EELHNHVHLYIGGLMAQIEAAYDPIFWFYHVYVDCLFEEFREK

>Cgi_K1PY25

TSNYETIANYHQANAGAHGGPNFPGWHRVFLLIYEEALIEALSGIPYIDWRLDLSSPGSSILWT

NEYLGNGDGRVRNGPFENWVINRTTLVRNVGTTQRDPTNESVAELFA---------TSARSFADVH

NIGHIYVGGLMNSLRATYDPVFFMHHCWIDYLWWRYQCP

>Cgi_K1QHI1

MSTYDLIGSMHTGRAAMHNGPGFFPRHMLMMLIMETAC---HSPMHYWDMTMDMIVPTDSI

MFSEEFYGNGDGIVRTGPFAHWRTPIGPIIRNIGSGGESLAGKGIRAMLSRRRLAEISEHAAYS

VEVHHNGVHNWIDGHMARLNAWFDPIFYGIHSFFTLIWIAFKGL

>Cgi_K1Q7A7

MSTYDLIASLHSGQAAMHNGPAFLPRHMVYLLVMETAC---RVPMPYWDMTTDMMDPTTSI

VWSDLFFGPGNGPVLTGPFGRFRTPTGPIIRNIGSGGASLARKGIRALLSRRRTFEISEQSIFSIEV

HHNGVHNYIDGYMSGLNASWDPVFWFIHSFFQLLWVAFRNG

>Cgi_K1QPB3

DGRYDALANMHTGIALGHEGPGFLAWHREYLTAFETALRRVSVSLPYWDSSKDMDNPALTS

FFSSALVGNGDGIVVNGPFAGWPAPDGRLSRDIGVIGSLFTPEGLDLFLNDPTVNLTRQMLAN

TLEGQHNNVHNWVGGDMSRLNAAHDPVFFMYHAHVDYVWERFREK

>Cgi_K1PS50

FNLYDILCNFHHASRAAHFGPAFLSWHRVSLYIFEQALRSKGVSLPFWDSRLELENPSATSLFT

DELMGPGNGVVKTGHFANWSHPAGDLVRNVGNLGEPIQRRDIERLLKARYTKEFMFYPHMN

LELIHGKVHMWVSGTMNNLNSPADPIFWMHHCFIDYVWEKIRQR

>Cgi_K1R2H1

ESKYDVLASFHSGATQAHGGCNFLGFHRLYTLMYEEALTQVEVTLPYLDSTLDLENSANSMI

WHDDFLGTKEGFVTSGPFANWNTTVGQLFRNVGISGRPMDDEEIKNVTSRTRMSEICGSTWH

DLEFVHGPFHLFVDGVMGIIEASEEPVFWMHHAFIDYVWELQREN

>Cgi_K1RLT5

PSRYDTISSFHTGDIEAHFGCHFAGFHREYLKVYEMALQEITVTLPYWDSLLDMDDSASSLMF

SPQFMGNGNGEVTSGPAANWQTETGPLRRNVGVEGELYLYEEIQNITTRTRFVEICGMENHG

LEFHHGDIHLWVGGNMAELNAADDPLFWMHHASVDRIWELQRQN

>Cgi_K1QL10

LNKYDLLANIHSRSSSAHGGPGFLGWHRVFLLLFENALRQMTATLPYWDCTLDLSHPSESVI

WSDLFLGNGDGEVNTGPFRGWNTQFGLLHRQVSSLRHLMSVHDLRNILEEDFLGNISYKSSK
NLEQLHNNVHVWVGGLMRKIEGAFDPVFYVLHTFIDKVWEDFRVH

>Cgi_K1P4E2

RNTYQTLAVLHSGEVLAHGGP-----------ALETAC---GSPIPYWDSTVDMMDPTMSILWSEQFFG
NGDGDVMTGPYQSIRTILGPLVRNIGTGSPLFTKEGLQAVLSRRRYSEIVEEYIFSLEGHHNGP
HTWVGGHLALTNAAYDPIFFNHHAFIDHVYELFRQQ

>Cgi_K1PAV4

MNEYQAIASLHTGQALAHDGAAFLPWHRIYLLLLETACR--GVPVPYWDSTVDMPDPTRSIV
WSEQFFGNGDGQVLTGPFRNFQTTPGSITREIGTSNALFTKEGLAAVLSRTRYSEIVEEYVYSL
EGHHNGPHNWVGGHLPLPWAAFDPVFFMHHAYVDAVWEVFRAQ

>Cgi_K1PI66

RNTYQTLAAFHSGQALAHGGPGFAPWHRIYLLLLETAC---GAPIPYWESGLDMEDPTASILWS
DDFFGNGNGVVTTGPFRSMRTILGPIIRNYGTGGALFTKTGYNAVLSRTRYDDISEAYFFTLEG
HHNGPHTWTGGHLARPNAPYDPVFYMHHSYVDAVYEAFRQR

>Cgi_K1QAP4

RNTYQTMAVFHTGETLAHGGPAFAPWHRIYLLLLETAC---RAPIPYWDSGLDMTDPTMSILW
SNQFFGNGDGEVMNGPFRDMRTILGPVIRNYGTGSALFTKEGLRAVLSQRNYADIAEAFMNS
LEGHHNGPHVWVGGHISALNAPWDPVFYMHHAYVDAVWERFREQ

>Cgi_K1QWH8

---MDMERPQESAMFTSQLVGNGIGPVINGPFAGWLEDG
GPIVRQIALFNSSLMVKRMEIFFEGNEALRHRDDGRYTIEGQHGNVHNWVGGTMSDPFTAFD
PVFILHHTFIDYIWEKFRQK

>Cgi_K1Q1M0

PNVYDWFCNLHPNKVAAHYGPAFFGFHRIYLYL-QQQLRTYDTFLPFWDSTYELGTPTSSVLF
TEDFLGGGTGTVTDGPFKNWKHDVGVLIRNTANSGQLFQRETIDKIMMKMFMSDISNEFDVN
LEAKHGQVHAWIGGAMDNLDSPADPIFYMHHCFVDAIWERFRDN

>Cgi_K1RE44

RSIYDLIGSLHSGQAAMHNGPGFLGRHTLYVLALETAC---RTPIPYWDFMMDMNDPTSSAVW
SNTFFGNGNGPVRTGFCGNWVTPQNPIIRNVGAGGVRLPRRALRALMSRTRTSEITEMSVFSI
EVHHNAVHNHIDGHFSALDSPFDPVFWFLHSMFHYMWYMFKNN

>Cgi_K1RLR4

-----------MMVIGHGGPNFLGFHRVYLLFFEFALQRISVSLCYWDSTMDMAEPQETAMFTSQL
VGNGNGPVVNGPFANWEEDDGPLTRNIATARSSLMVKALNRFFEGNSATTHRDNLANTIEG
QHNNVHNWVGGTMADAVTAYDPVFILHHTFIDYVWERFRQK

>Cgi_K1R932

TNTYDLIGSLHSGQAAMHNGPGFLGRHSLYVLAMETAC---RTPIPYWDFMMDMTDPTSSAIW
SNTFFGNGNGPVRTGFCGNWVTPQNPIIRNVGAGGLQLPRRVLRAILSRTRTSEITEMSVFSIE-
----
>Cgi_K1RFL7
TNTYDLIGSLHSGQSAMHNGPGFLGRHALYVLAMETAC---RTPIPYWDFMMDLNNPTSSAIW
SNTFFGNGNGAVRTGFCGNWVTPQNPIIRNVGAGGVRLPRRALRAILSRTTTREITEMSMFSIE
VHHNAVHNYVDGHFSALDSTFDPVFWFLHSMFHYMWYMFKNN
>Cgi_K1Q6C9
SNRYDVIAALHEGAIVAHEGPNFMGWHRIYLIVYENALRQIGVTIPYFAGDLDLRDSTQSVLF
CERFFGNGNGVVTSGPYANWSTPSGPLVRNYGDDGELWTREGLQRILNKTRNAEIIAEEEDN
LEDQHGAIHNWIGGQIGELQSSQDPAFFSLHAYVDYIWEEFRKR
>Cgi_K1QE57
PNRFDALGLLHQQRGDVHHGAAFLSFHRVLLLIFENALRQKGVALLYFDSRLDLRDPTRSIIW
SPQFLGTVKGRVTDGPFRFWQTPAGPLVRAGGHEGEYFTYRHIRAVMTRSRLEEISEPPPFDF
EIRHGDVHQHIGGIMAPAEAGFDPVFYMHHCFVDYLWEVFRRS
>Cgi_K1QMD1
PNKYDSLAALHQGITEAHGGPNFLGWHRVFLIMFENALREKDVTLPYWDSTIDMANSVASAI
WSDRFLGNGNGLVTNGPFADWMTPAGPLIRNIANTGQLFRRNVIQNILTRKRLSEITEFTQFN
LEFHHGEVHMWVDGQMGELTAAMDPVFFLHHAYIDYVWEKFRAQ
>Cgi_K1Q6D2
PNKFDAIASLHHLNTAAHGGPGFLGWHRIYLTLFENALREKNVTIPYWDNTLDLPDPRRSIM
WSPLFFGNGNGAVVTGPFRRFTTPYGPLRRDIGADRRLMSKTDLENVFSRRWMWEISNEDRY
NLELLHNHVHVWIGEQMSRIESSYDPAFFSHHAFVDCLWEEFRQR
>Cgi_K1QXR1
PNRYDALGLVHFRMVDIHHGGAFLAWHRLFITIFENALRQKDVTLPYWDSTMDMIDPTQSVT
WSPQFLGNGDGLVTTGPFAFWQTPNGPLIRNVGQDGQLLSRQAIMRVLSRTRMAEITEPDQY
NIENYHGDAHTWIGGQMEPMESAFDPVFYLHHAFVDYVWEIFRQQ
>Pmax_P86952
PNEYDTLANLHRGAVQAHDGSNFLGWHRVYLMYYERALRRIDVTLCFWDTTMEMDNWEY
TAVFSSDFFGNRRGQVITGPFRDWPLPPGTLYRNMTRGGMPFDSRAASSIFYNPNTSTITDTRN
ITIEGEHNNVHNWVGGAMGFLDAPQDPIFFFHHCYIDYVWERFREK
>Pmarg_H2A0L0
MSRYDTIAGLHRQAIAAHMGANFLGWHRLYLDMFEMALQETDVVLCYWDSTLDGTSQVNT
VSFSAELFGNGRGVVINGPFRFWRLPGGTLQRFIARPGSSLTRPVVDLIATDPRINTNSQ-RGQG
FPDEHNNTHVWVGGVMQVVSSPQDPVFWFHHTYVDYVWELFRQK

>Pmarg_H2A0L1

PNEYDTLANLHRGAVQAHDGSNFLGWHRVYLMYYERALRRIDVTLCFWDTTMDMDNWEY
TAVFSSDFFGNRRGQVITGPFRDWPLPPGTLYRNMTRGGMPFDSRAASSIFYNPNTSTVTDTR
NITIEGEHNNVHNWVGGAMEFLDAPQDPVFFFHHCYIDYVWERFREK

>Pmax_A0A024CJP7

PNEYDTLANLHRGAVQAHDGSNFLGWHRVYLMYYERALRRIDVTLCFWDTTMEMDNWEY
TAVFSSDFFGNRRGQVITGPFRDWPLPPGTLYRNMTRGGMPFDSRAASSIFYNPNTSTITDTRN
ITIEGEHNNVHNWVGGAMGFLDAPQDPIFFFHHCYIDYVWERFREK

>Pmax_A0A024CI03

PNRYDALGLLHQRRGDVHHGAGFLGFHRVLLVVYENALRQKTVTLPYWDSRLDLRDPTRSII
WSPQFLGTMRGRVINGPFAFWQTPAGPLVRNGGQEGELFTYNHIRAVMTRSHLEEISEPPPFD
FEIRHGDVHQMVGGIMAPAEAGYDPVFFLHHCFVDYLWEVFRRS

>Pmax_A0A024CHU4

GNEYDTMAHTHLPAVISHDGSNILGWHRLFLFLFEIALRRKGVVLCYWDSSLDGRGQVQSAA
FSHELFGNARGQVTTGPFANFPTPWGPLRRNFGGEGGSLVRPIVDMIERDPNIRSHGQDGATG
FTDEHNNAHVAVGALMAIPNAAYDPLFFFHHCYIDYVWELFRRK

>Pmax_A0A024CHG7

PNEYDTLANLHRGDVQAHQGSNFLGWHRVYLMYYERALRRIDVTLCFWDTTMEMDNWEY
TAVFSSDLFGNRRGQVITGPFRDWPLPPGTLYRNMTRGGIPFDSRAASSIFYNPNTSTITDTRNI
TIEGEHNNVHNWVGGAMEIIKAPQDPIFFFHHCYIDYVWERFREK

>Pmax_A0A024CIJ0

PNKYDALASLHHLNSAAHGGPGFLGWHRVYLVLFENALREKNVTIPYWDSTLDLPDPRRSII
WSPLFLGNGNGPVVNGPFRRWSTPYGPLRRDIGADRRLMNRQDIQNVFSRRWLWEITNRDE
YNIELLHNHVHVWVGEQMSRIESSYDPAFFAHHAFIDCLWEEFRQR

>Pfu_Q287T6

GNRFDTMARIHMPAVIAHDGSSILGWHRVFLYLFENALRRKGVVLCYWDSTID-----YLIPGPG
QAQSSSFSHVRTGPFANFPTPWGPLRRNFGGEGGSLMRPVVDMIASDPRIRSHGQQGATGFID
-HNNAHVAVGALMAIPNAAWDPLFYFHHCYIDYVWQLFRRK

>Pfu_A1IHF0

MSRYDTIAGMHFAAYRAHLGPSFLGWHREYLIMYEEALQEIDVVLCYWDSTLD-----FLMPGS
TQRFTVAFSAVINGAFANWQLPGGGAGDRFPGPPPSLTRPIVDLIATDPSITSHTQRGGTGFTD-
HDNTHVWVGEIMQVVAAPGDPVFFFHHTFIDYGWELFRQK

>Pfu_A7BK18

MSRYDTIAGMHFAAYRAHLGPSFLGWHRVYLIMYEEALQEIDVVLCYWDSTLD-----FLMPGS
TQRFTVAFSAVINGAFANWQLPGGGARAQFPNPPPSLTRPIVDLIATDPSITSHTQRGGTGFTD-

HDNTHVWVGEIMRVVAAPGDPVFFFHHTFIDYGWELFRQK

>Pfu_A1IHF1

MSRYNTLAAMHLQAFGAHNGPNFLGWHRVYLNMYEEALQEIGVALCYWDSTLD-----YLMP

GDSQRRTVAFSDVINSQFANWRLDNTPLRRMIGENNSSLTRPIVDLILTDPRINRHRWSPRFGF

ID-HDNTHVWVGGIMVVERSPEDPVFWFHHLYIDYVWELFRRK

>Pmax_A0A024CIJ4

GNEYDTMAHTHLPAVISHDGSNILGWHRLFLFLFEIALRRKGVVLCYWDSSLDGRGQVQSAA

FSHELFGNARGQVTTGPFANFPTPWGPLRRNFGGEGGSLVRPIVDMIERDPNIRSHGQDGATG

FTDEHNNAHVAVGALMAIPNAAYDPLFFFHHCYIDYVWELFRRK

>Pmax_A0A024CI05

TSAYDFFAGLHRSLRSAHIGSNFLGWHRVYLWYFERILIRVGVPLCYWDSTLDGSGQRNTTM

FTSEVVGNGIGMVINGPFRNWPIPDRSLRREIAS-FASLMRPVVDLIMTSNLIRNHSQAGSVGMI

DEHDNTHVWVGGVMSDATAPQDPVFWLHHTYIDYVWEKFREK

>Pmax_A0A024CHH0

ESMYDTFARVHQNQESAHGGSNFLGWHRLYVLFFENALRRIGLVLCYWDPTLDSTLQIHSVT

FSDRLFGNGYGTVINGPFKNWQLPYNRLRRNIGQ-EGSLTRPVIDIITLNPKIIRSTQLGAIGFKD

CHDNTHVYVGEVFSSLPTAQDPIFWFFHAYVDYVWELFR--

>Pmax_A0A024CJQ0

TSAYDFFAGLHRSLRSAHIGSNFLGWHRVYLWYFERILIRVGVPLCYWDSTLDGSGQRNTTM

FTSEVVGNGIGMVINGPFRNWPIPDRSLRREIAS-FASLMRPVVDLIMTSNLIRNHSQAGSVGMI

DEHDNTHVWVGGVMSDATAPQDPVFWLHHTYIDYVWEKFREK

>Pmax_A0A024CHU9

MSQYDTIAGLHLQAFPAHQGANFLGWHRIYLNMFETALQESDVVLCYWDSTLDENTQLNTV

TFSAELFGNGRGSVINGPFRNWRLPGGTLQRFIAGPGSSLTRPVVDLIATDPRINSHSQ-GGQGF

PDEHDNTHVWVGGVMQVVLSPQDPVFWFHHTYIDYVWELFRQK

>Pmax_A0A024CIJ8

PNLFDSLAAVHTGTAIAHFGPNFLGWHRIYLYYYEIALRRIRVALCYWDSTLDMDSPERSVM

FSSEYFGNGNGVVLEGPFRDWILPNRPLRRQINEVGSLMVYDGVREILTDPQLIRTANPIQRTI

EGLHNNVHVWVGQIMSGVDAPQDPVFYFHHCYIDYFWERYREK

>Pmax_A0A286K027

MSRYDTIAGMHFAAYRAHLGPSFLGWHRVYLIMYEEALQEIDVVLCYWDSTLD-----FLMPGS

TQRFTVAFSAVINGAFANWQLPGGGAADRFPGPPPSLTRPIVDLIATDPSITSHTQRGGTGFTD-

HDNTHVWVGEIMQVVAAPGDPVFFFHHTFIDYGWELFRQK

>Lan_g6403

FSEYDIFTAYHQATEAAHFGPAFLPWHREFIKRFETAMQRHGVTLPYWDPTMDMPRPQDTIM

WTPEYMGNGFGYVTSGPAANWNTP--PLYRNLTAGRVPLLPNDLEMGFLYAGGRGLRD--DP
SLEFVHGRVHDWIGGHMGDLEAPADPVFYMYHAYLDCWWDDYRQK
>Lan_g6405
VREYDIFVGYHRANLSAHFGPAFLPFHRELLFRLERALQRFDVALPYWNSTMDLENAEDSIM
WTEEFAGNGYGNVTTGPFANWPTTEG-LFRNLTYGPGLMADD----------------DSATRFRDNHG
KTHNWVGGQMIDLESPGDPVFFLHHAFIDCAWEEFRER

C1q (supplementary figure S9)
>SMP_Equ22322
AFSALINGNVYYTIVRYPVVTTNNGNNYNPATGVFTAPATGFYFFHVNAQAQLEKVLALGLY
HNHKYVLSVYASSSKQPANGSNAIVLKLNYGDTVCVKARGPSFLYGGDCIQSSFSGFQL
>SMP_Equ12964
AFFANIKQARDYAIVKFDNVITNDGNAYNKDTGIFTARKPGLFVFYFSTLSAQGGLFLFDLFL
NNEILASAYENVGSSYGQGGNSAIVRIKTGDQVCVKAKQASKIYNNPDPYATFSGYSL
>Equ15925
-------------TLVLNKVLFNQGGGYNSNTGIFTAPIGGIYQFQMYGLTAKGSSFWIKLYHNNVYVI
SAFARSGDY-SSAGNSVFVLAKKNDQFSLRAVGAAAFYNRPDIYTTWSGQ--
>Equ21838
AFTAGCDGNRDLKTLVLNKVILNQGGGYDKNTGIFTAPQGGIYQFQMHGLAPSDNSFWIKLY
HNNMYVISAFAGVNSHDSSGGNSAFLLVRRNDKIYLKVVEQAHFYGRPDIYVTFSGHRI
>Equ38058
---LELWLHLYHNDDYVSTAFARESTQFDNTGNTAILP
LKKNDKVYIKVEGTSLFVG-ENVYTT------
>Equ21683
-------------RLCF--------------TGIFTAPKAGYYVFHIHGLAFPNAGLWFRLFHNGVARASAHGSSP
TG-ATGGNTVGLYVKKNDRIYVKNVLHHPRLT---RRATL-----
>Lgi_57485
AFFVGLQENQGPVDLLFDKVITNVGNVFNPDTGRFTATHNGTYQFNVITTHHAFFQAAVNLV
RNGEMIATVWAESIPYWASASNTAILKLKGGDQVWLVLLS-RAPYIHGYMYSTFSGHLL
>Lgi_138166
SFSAKLSYNRELQTVIFDTVVTNNGDGYNSENGKFTVPVTGTYFLFSTILSGYNTKVETAVIV
NDKEVGRMYSGAHDAHGSGSNGVVVNLQSGDNVWIRLLYQGGTHVHG-FYTTFSGFLV
>Lgi_58867
AFAARTRTNKAFLTIVFDEVTVNNGNAYDPLTGIFTAPFNGVYHFSSTILSGFNATIETMFVLN
GEEVGRMFSGAFLSRGSGSNSILLNLKERDEVTVNVFYGNGHYVHG-GWSTFMGYLI

>Lgi_163012

HFFARMSGTKTL-IVVFDEEIDDYDNNYNRGDGIFVAPVTGFYQFSWTLFTASKKNIYTELRV

DNFVIDVAYDYSSAGSTSVSKTVICKVKKGNHVWIQTGGQENYFYKYVSKNSFMGLLL

>Lgi_68918

AFSASRSTKLGPVAVTFDRVHINLGDSFDPYTSHFICKHNGTYIFTTHILGQNKIDAYAWIMVN

KNHKLPLHGDGRAGYGTGSNTIILELTVDEHVWIQLSINSALMN---DYSSFAGHIL

>Cte_57561

------------TITFDVQYTNVGDGFDVYSSHFVCKHNGTYLFATHILGQNEKDVYAWIMFNDKHK

VPLHGDGRAGYGTGSQTVILQLRVDDHVWVQLSKDSGLLN---DYTTFSGYLL

>Cte_98013

AFSATMDQDMPVSVVKYNIVLTNLGDAYSPHTGVFVAPCNGTFYVGFSGVSLSGKDVLLHL

VRNGQRLLSAFDNSGCQVGMASNALVLVLTTGDKLWVELPDGYGLHNALYSYSTFHGALV

>Cte_98879a

AFYAAREESFYEQRIPMSRVYMDIGENYDETEMEFTAPETGVYYFFFGAGQETGTPNRVALR

TWEATVAELWVHSSSHNDMAARGTVVELDAGQQIWVTAMEDP-LYSDTDNQIFFGGFA-

>Cte_98879b

AFAAHRTSPWQGGPVPFQEIAVNVGDAFDAASNTVVIPTSGYYYIELSIGSMPFKAIDVEMLA

NGERIVHVNVASTSHSTTTGRSVVVHLNQSDVLRLSAGGVTGIFSDRDKQTTWMGFLL

>Cte_168874

AFFVGLSDNMGPVDIIFDRVVTNVGGAYDPVTGRFTSPANATYQFNVIVAAQGRQKAAVMII

KNGGMIATVWAESIPYWATASNVVVLSLEKGDQVWLMLLD-RASYLHGYMYSTFSGFII

>Cte_183227

AFFAGLSDNIGPVDILFDQVVTNEGSGYDATTGRFTAPVNGTYQFNVVISAQGRQKAAVMIL

KNGIMVATVWAESIPFWATSSNIVILSLEKGDSVWLALLN-RASHLHGYMYSTFSGFLI

>Cte_188454

TFAATITRNYNN-VMTFNDVYVNENAVYDEENQQYICGETGVYLFDLTVGLRAGQAVRVAIE

RTDPKQFELTRTSTSHDDTLGRTIVVNCQRNERVFVNLIYG--LYGSEEAVTSFSGFQ-

>Cte_197838a

CFFAGRKGGTSLLRLQFSDQFITPSGSAYTNGDKYICPQTGIYYFGFSFGVDNQLPARLALMK

TVVQVAELTMTNNNIENTYSKEVLIQCSSGDEIWLKLVQGS-ISGKGGYPISWMGFQ-

>Cte_197838b

AWSMSATKGGSGSSISFSNELVMQPKSMISGNSEVTIPQTGYYYLSISGGASGSSNLDIGLYVN

EEKLFGVARTTHGNGETLGHGMLYWLMSGDTLSVKAELNSDYFSQLSGQTSFMGFLL

>Cte_197838c

TFAATITRNYNN-VMTFNDVYVNENAVYDEENQQYICGETGVYLFDLTVGLRAGQAVRVAIE

RTDPKQFELTRTSTSHDDTLGRTIVVNCQRNERVFVNLIYG--LYGSEEAVTSFSGFQ-
>Cte_197838d
---AYRNTEWQPVPVTFNLVEVNLGGAF--VDNTVVIPRSGYYLISVSVGTSPIKGVNVDIHV-DS
VYADVYRAASYHNDTLSKTFVLRLEAGDILRLVSESNTNIFSDPGLQTAFTGFLI
>Cte_212339
---AYRNTEWQPVPVTFNLVEVNLGGAF--VDNTVVIPRSGYYLISVSVGTSPIKGVNVDIHV-DS
VYADVYRAASYHNDTLSKTFVLRLEAGDILRLVSESNTNIFSDPGLQTAFTGFLI
>Cte_214207
IFSAVRNEDVKS-TINFDVILSNEGGAFQ--NPNFVAPYSGTYSFALNFETVSTQAVYYDLMHA
NNLLVR---------TVLEIDVAVHMLKGQKLWLKVTGGGTMKG---GYQLFSGQML
>Cte_215797a
TFSAVRNSVFNSTNLPMDDVIINEGSAFTTDGSYFTAPEDGIYYFSFSVGQVPFVESRVSLLIDP
QAEVDLWKGSNNHNDMQSRAVLARLTKGQRAWLAVQADGVVYSEEEFQTAFAGYH-
>Cte_215797b
AWCVHRLTSHPAGPIPFEEITVNEGDLFN--DGFIDVSTSGYYFIEFNIGVLGGQAVNVELVRTD
EVLAGVARTSFAHEDNVGRSLIARLESGDRLRLRVAQGTGFFSTRERHTSFLGLLL
>Cte_215797c
AFYVTRNSSFHIHAIMFEEKLIDTKDAFNMEFGYFTAPKTGLYFLTYGVGQAAGIESRVDLRT
RPQTEVSLTKQSSEHNDMQTRSTLVHLDEGEDIWVALTTGE-IHSDTRNQISFAGFHL
>Cte_215797d
--CAHRFSSWHGIPVPFPLIEVNEGDIYNPDTHVITVSISGYYYIEVNIGALPDRAVGVDILRAED
LLGRVSRKSTVHRDSIGRSVFVYLAVGDTLRLRAHVQTGIFSHDDKATTFMGFLV
>Cte_221640
AFSATVTQPYNITVIVFDDIATNIGGFYNAQLGNFSPACGGVFSFTVDLSSQRGTLASVHLMH
NERVLLPLRANGRFTGGTASGTAMVQLLEDDHVFVVLVYDTYVLP---GYTRFSGFMV
>Cte_228528a
IFFATRTSDWSG-VLLFDDTRVNIGQRFQDGF-SFTANNSGLHFFSLSAGALAHTETILSLTDIKP
ARGNIRRESTANSDVIYRNTIVNLDRDQAITVEAM--TVLASNGDALISWGGF--
>Cte_228528b
VFSAGRDSEYRPDIIELPDVFVNQGDSFIEDSSQFKAPEDGIYYFTFSIGQINGDPARVALGVQA
DNEAEIICGQGNTNNIQSRSTVVQLEEGDLVWISTLNPS-VYSDPDYQTSLSGFK-
>Cte_228528c
---------WSGNPIEFTSVAVNVGEVYNEASDVFEISVSGYYFLEFNVGVLGGRPTYVKLIKGGEV
LASVVHEAVAENDNVGRSLITLLEAGDVIRLAADENTGIFSDSERQTTFLGLLL
>Cte_190519a

AFYVAREEPFEFNRIPMSRIFMDIEDNYNKTGREFIAPETGIYYFFFGAGQQSGKTNRVALQTW
PKTEAELWMNSDSHNEMASRSTILRLTAGERAWVTAVEGP-VESMVDCQVYFGGFW-

>Cte_190519b

AFAVHRTSPWQSAPVEFHEVGVNQGNAFNHAANVVIIPKSGYYYIELSIGSMPHTTLDVEILL
NGERIAHVSVESTNHTATSGRGLITHLWQNDILRLRANGLSGIFSDLNKQTTWMGLLL

>Cte_196716a

AFSVSRSSDWYG-DVYFDEVDVNLGDCFKTST-TFQPGRYGVNFFSFSGGVPSQTPADIKLRLN
PNSPSSIYRESTTPEVMMYRNALLEMVPSDSLTFQSL--NGLKS--RSQASFSGFSL

>Cte_196716b

AFYTTRDDSFDEQRIQMSHIDLNIGNNYDLVNREFIAPQAGVYYFFFGCGQEAGIDNRIALRT
WPQTEAELWVHSTSHNDMSTRGTMIELNAGQRIYVSLMEKA-IYSDAERQIYFGGFLY

>Cte_196716c

AFAVHRGKPWQSAPVPFEYIGVNQGNIFNALSNTVHIAIRGYYYLEVSLGTLPFLPLDVEIMV
NGETIAHIIVASSNHSASTGRSLITHLEQNDVLRLRAKADTGIFSDMDKQTTWMGFLL

>Cte_223886a

AFYVARDYPFYGQAIPMTEVYLNVGNNYDIYNHQFVAPQAGVYYFFFGSGQAAFENNRIAL
MSWPETEAELWVHSTSHNDMANRGMMMELYAGQRVWLNLQEDP-LYSDAENQAYFGGFL
Y

>Cte_223886b

AWAVHRDKPWQGAPVPFEVIGVNEGNAFQALANRVIIPTRGYYYMEVSFGTLPYLPLDVDIL
VNGVAIGHAFVASTNHSASTGRSLIIHLEMHDVVRLKAQPETGIFSDLDKQTSWMGFLL

>Cte_223943a

AFYVARENSFHEQRIEMLRVFMDTGNNYNMSGREFVAPEAGVYYFFFGSGQEAGIDNRVAL
RSWPETEAELWVHSTSHNDMATRGTMLELNAGQSVWVTLMENS-VYSDADNQIYFGGFLY

>Cte_223943b

AFAVHRTKSWQSVPVPFEEIEVNQGNAFDAGTNTVVIPTSGYYYIELSVGTLPFKPLDVEMLV
NGDPLASVIVRSSNHSASSGRSLVTHLQQNDVLRLRAMGSTGIFSDLDKQTTWMGFLL

>Cte_223944a

AFYATSTTSWHG-DMRFDRTYVNTGGCYKDET-TFEPGREGVYFITFSAGVPARTRADLKVRP
MPNYPSTIYRENTVQEDAVYRNFLCTLQDDSYLSYKGF--EATVSTEAQQTSWAGFSL

>Cte_223944b

AFYAARETDFIGQRIPMDRVYMDHGNNYNETGHEFIVPVTGVYYFFFGSGQAPGVTNRVALR
TWPQTEAELTVQTTSHNDMAARGVLLELTAGERIWMSALEDP-INCDDLHQSYFGGFLY

>Cte_223944c

AWAVSRTEPWQSGPMPFQDVGVNVGSAYDPYSNTVTITTTGYYYLEVCLGTMPLKPLDVEM

ILNGERIAHFLVAGANHTATTGRGLIYHLEVNDVLRLRARGTTGVFSDVDKQTSWMGFLL

>Cte_226148

GFSANAFGVEHE-LIRFTLTTSNIGGGFE-TSGKFIAPLGGAYFFHVTARANGNGATPLLLFQGN

NIVMLTGRNDADAVPVGTNSILVELEAGESVQLKIKPKSGFFSTSSTDITFVGHRI

>HsaC1QTNF1_Q9BXJ1

AFSVGRKKPMHSNTVIFDTEFVNLYDHFNMFTGKFYCYVPGLYFFSLNVHTWNQKETYLHI

MKNEEEVVILFAQVGDRSIMQSQSLMLELREQDQVWVRLYKGNAIFSEELTYITFSGYLV

>HsaC1QTNF2_Q9BXJ5

AFSVAVT--KSYPPIKFDKILMNEGGHYNASSGKFVCGVPGIYYFTYDIT-LANKHLAIGLVHN

GQYRIRTFDANTGNHDVASGSTILALKQGDEVWLQIFYSNGLFYDPYTDSLFTGFLI

>HsaC1QTNF3_Q9BXJ4

AFMASLATHFSNQGIIFSSVETNIGNFFDVMTGRFGAPVSGVYFFTFSMMHEDVEEVYVYLM

HNGNTVFSMYSYEMKGKDTSSNHAVLKLAKGDEVWLRMG--NGALHGDHRFSTFAGFLL

>HsaC1QTNF4_Q9BXJ3a

AFSAARTTPLEGTAVTFDKVYVNIGGDFDVATGQFRCRVPGAYFFSFTAGKAPHKSLSVMLV

RNRDEVQALAFDEQRRPRAASQSAMLQLDYGDTVWLRLH--GAPQYALGPGATFSGYLV

>HsaC1QTNF4_Q9BXJ3b

AFSAARTRSLVGSPLAFDTEFVNIGGDFDAAAGVFRCRLPGAYFFSFTLGKLPRKTLSVKLMK

NRDEVQAMIYDDGASREMQSQSVMLALRRGDAVWLLSHDHGA--YSNHKYITFSGFLV

>HsaC1QTNF5_Q9BXJ0

AFSAKRSESRVPPPLPFDRVLVNEQGHYDAVTGKFTCQVPGVYYFAVHAT-VYRASLQFDLV

KNGESIASFFQFFGGWPASLSGGAMVRLEPEDQVWVQVGVGIGIYASIKTDSTFSGFLV

>HsaC1QTNF6_Q9BXI9

AFSVGRKTALHSGTLLFERVFVNLDGCFDMATGQFAAPLRGIYFFSLNVHSWNYKETYVHIM

HNQKEAVILYAQPSERSIMQSQSVMLDLAYGDRVWVRLFKRNAIYSNDFTYITFSGHLI

>HsaC1QTNF7_Q9BXJ2

AFSVGIT--TSYPPIIFNKVLFNEGEHYNPATGKFICAFPGIYYFSYDIT-LANKHLAIGLVHNGQY

RIKTFDANTGNHDVASGSTVIYLQPEDEVWLEIFFTNGLFSDPGADSLFSGFLL

>HsaC1QTNFP60827

AFSVGRREGLHSSAVPFDTELVNLDGAFDLAAGRFLCTVPGVYFLSLNVHTWNYKETYLHIM

LNRRPAAVLYAQPSERSVMQAQSLMLLLAAGDAVWVRMFQRNAIYGEHGLYITFSGHLV

>HsaC1QTNF9_P0C862

AFTVGLTVLSKFPPIKFDKILYNEFNHYDTAAGKFTCHIAGVYYFTYHIT-VFSRNVQVSLVKN

GVKILHTKDAYMSSEDQASGGIVLQLKLGDEVWLQVTGGNGLFADEDDDTTFTGFLL

>HsaC1QTNF9B_B2RNN3

AFTVGLTVLSKFPPIKFDKILYNEFNHYDTAVGKFTCHIAGVYYFTYHIT-VFSRNVQVSLVKN
GVKILHTRDAYVSSEDQASGSIVLQLKLGDEMWLQVTGGNGLFADEDDDTTFTGFLL
>HsaC1QA_P02745
AFSAIRRNPPMGGVVIFDTVITNQEEPYQNHSGRFVCTVPGYYYFTFQVLWEICLSIVSSSRGQ
VRRSLGFCDTTNKGLQVVSGGMVLQLQQGDQVWVEKDPKKGHIYQGSADSVFSGFLI
>HsaC1QB_P02746
AFSATRTINVPLRTIRFDHVITNMNNNYEPRSGKFTCKVPGLYYFTYHAS--SRGNLCVNLMRG
AQKVVTFCDYAYNTFQVTTGGMVLKLEQGENVFLQATDKNSLLGMEGANSIFSGFLL
>HsaC1QC_P02747
VFTVTRQTHQPPALIRFNAVLTNPQGDYDTSTGKFTCKVPGLYYFVYHAS--HTANLCVLLYR
SGVKVVTFCGHTSKTNQVNSGGVLLRLQVGEEVWLAVNDYYDMVGIQGSDSVFSGFLL
>MmuC1QL2_Q8CFR0
AFYVGLKSPHEGYVLKFDDVVTNLGNHYDPTTGKFSCQVRGIYFFTYHILGGDGTSMWADL
CKNGQVRASAIAQDADQNDYASNSVVLHLDSGDEVYVKLDG-GKAHGGNNKYSTFSGFLL
>MmuC1QL4_Q4ZJM9
AFYAGLRRPHEGYVLRFDDVVTNVGNAYEAASGKFTCPMPGVYFFAYHVLGGDGTSMWAD
LMKNGQVRASAIAQDADQNDYASNSVILHLDVGDEVFIKLDG-GKVHGGNTKYSTFSGFII
>MmuC1QTNF1_Q9QXP7
AFSVGRKKALHSNPVVFDTEFVNLYKHFNMFTGKFYCYVPGIYFFSLNVHTWNQKETYLHIM
KNEEEVVILYAQVSDRSIMQSQSLMMELREEDEVWVRLFKGNAIFSDEFTYITFSGYLV
>MmuC1QTNF2_Q9D8U4
AFSVAVT--KSYPPIKFDKILMNEGGHYNASSGKFVCSVPGIYYFTYDIT-LANKHLAIGLVHNG
QYRIRTFDANTGNHDVASGSTILALKEGDEVWLQIFYSNGLFYDPYTDSLFTGFLI
>MmuC1QTNF3_Q9ES30
AFMASLATHFSNQGIIFSSVETNIGNFFDVMTGRFGAPVSGVYFFTFSMMHEDVEEVYVYLM
HNGNTVFSMYSYETKGKDTSSNHAVLKLAKGDEVWLRMG--NGALHGDHRFSTFAGFLL
>MmuC1QTNF4_Q8R066a
AFSAARTTPLEGTAVTFDKVYVNIGGDFDAATGRFRCRVPGAYFFSFTAGKAPHKSLSVMLV
RNRDEVQALAFDEQRRPRAASQSAMLQLDYGDTVWLRLH--GAPQYALGPGATFSGYLV
>MmuC1QTNF4_Q8R066b
AFSAARTRSLVGSPLAFDTELVNIGGDFDAAAGVFRCRLPGAYFFSFTLGKLPRKTLSVKLMK
NRDEVQAMIYDDGASREMQSQSVMLPLRRGDAVWLLSHDHGA--YSNHKYITFSGFLV
>MmuC1QTNF5_Q8K479
AFSAKRSESRVPPPLPFDRVLLNEQGHYDPTTGKFTCQVPGVYYFAVHAT-VYRASLQFDLVK
NGQSIASFFQYFGGWPASLSGGAMVRLEPEDQVWVQVGVGIGIYASIKTDSTFSGFLV

>MmuC1QTNF6_Q6IR41
AFSVGRKTGLHSSSLLFDRVFVNTDGHFDMATGSFVAPLRGLYFFSLNVHSWNYKETYVHIV
HNEQAVVILYAQPSERSIMQSQSVMLPLVPGDRVWVRLFKRNGIYSDDVTYITFSGHLI
>MmuC1QTNF7_Q8BVD7
AFSVGIT--TSYPPIIFNKVLFNEGEHYNPATGKFICAFPGIYYFSYDIT-LANKHLAIGLVHNGQY
RIRTFDANTGNHDVASGSTVIYLQPEDEVWLEIFFNNGLFSDPGADSLFSGFLL
>MmuC1QTNF9_Q4ZJN1
AFTVGLTVISKFPPIKFDKILYNELNHYNVATGKFTCHVAGVYYFTYHIT-VFSRNVQVSLVKN
GVKVLHTKDSYMSSEDQASGGIVQELKLGDEVWMQVTGGNGLFADEDDDTTFTGFLL
>MmuC1QA_P98086
AFSAIRQNPMTLGVVIFDKVLTNQESPYQNHTGRFICAVPGFYYFNFQVIWDLCLFIKSSSGGQ
PRDSLSFSNTNNKGLQVLAGGTVLQLRRGDEVWIEKDPAKGRIYQGTADSIFSGFLI
>MmuC1QB_P14106
AFSALRTINSPLRVIRFEKVITNANENYEPRNGKFTCKVPGLYYFTYHAS--SRGNLCVNLVRG
MQKVVTFCDYAQNTFQVTTGGVVLKLEQEEVVHLQATDKNSLLGIEGANSIFTGFLL
>MmuC1QC_Q02105
VFTVTRQTTQYPELVRFNSVVTNPQGHYNPSTGKFTCEVPGLYYFVYYTS--HTANLCVHLNL
NLARVASFCDHMFNSKQVSSGGVLLRLQRGDEVWLSVNDYNGMVGIEGSNSVFSGFLL
>Cgi_K1PDL0
AFTSQISLEGAMSTIVFDLVEYNMGGAYDPTTGKFKAQVSGTYVFFFNILTRPHMRLGVDLTI
NGETRSACAYSGADPNVNGSNMVVVHLNKGDEVWVRAHKSPGVML--SFANSFAGFLL
>Mga145893971
AFSAELTHPIENMIAHFDKVRVNSGHAYHADTGKFVAPEEGFFYFSVTICTKKDSILEMALHV
NDHDEMLIHADAEHLEGCASNSEIVHLQKGDHVEVVKHGPDGVPPFYVTMSTFTGFML
>McoCL6812Contig2
AFSAELTHPIENMIAHFDKVRVNSGHAYHADTGKFVAPEEGFFFFSVTICTKKDSILEMALHV
NDHDEMVIHADAEHLEGCASNSEIVHLQKGDHVEVVKHGAEGVPPFYVSMSTFTGFML
>Bgl_XP_013081758
AFSAGLTHGVNVSNVTYDRVWTNVGNGYDPTTGVFTAPVDGTYTFLYHALAEFDGMLWLD
FYQNSNYISSAYAHIENQYGATSNSVTLNAKKGDQMYITGHGTSILYGLSDVYATFSGYLL
>Bgl_XP_013091313
GFSAIYNSPRLLETLIYNKVITNVGGAYNPSTGIMTCPLSGLYVINVGGLSTPGNLMTLNLYHN
GKYLITVHAYDESAHSSGSK-------------------------------------
>Bgl_XP_013075372
AFSVALTQDLSLEYVPFDKVYTNAGNGYNGSLSIFTAPVSGLYKFDVAVKSQDALQVRLNLY

KNNEYIMSVFSKEANKK------------------IRQQ-------------------

>Bgl_XP_013091954

AFSAGLSHHETLVKVVYDTIFTNVGGAYNSNTGIFTCPTAGLYVFQFHALSHSSGNMWLELH

HNYNYVASIWGHIDNEYSAAGE----------------------------------FHT

>Bgl_XP_013074784

VFSVNVSSSYTI-KLLFTTPSLNKWGCFNQQDAVFEAPVNGIYFFSISVNADSPGELCLGIIHNN

KSTA-LCSTCGTKNAQSSTSVLLELAVGDKVWVEANRQTDINGKIGGYSNFIGFRV

>Bgl_XP_013062292

GFSSLLNLERPIARIIFDKVLTNVGNGYDPKSGNFTCPIRGLYSFSVGVLPMPNNLVIVDIYQNK

NYLISVHGNDNAVFTS--

>Cepaea-hortensis_CAD83837

AFTAVLDKNLVLETLIPNKILINYGGGYNDKTGIFTAPKSGIYHLGVHAQTSLQSNLWLALYH

NDNYVFSIYGRQTEYSDGGANAAILPLKKGDKVHVKARDKSSLLGRPDIYTTFTGFRL

>Helix-pomatia_ABF00124

AFSAAIDREQTFDVVIYDIVITNHGNAYDNSTGLFTAPVDGMYSFQLNLLTIKEKEGWLELVH

NGQLKVSVYAKQDSTYDSSSNSVIIKMKEGDRVNVRAHKKSGLFGRDDLYNTFSGHFL

>Haliotis-madaka_ALU63755

AFSAGLTKHLTLQTVIYDKVFTNIGNAYDNNTGVFTCPQTGIYVFQYHGLSMSDDTLWLELY

HNYNYVSSAYAHTNSDYASAGNSVILHLFKGDTVMVNAEPNSNLYGVSDVYCTFSGYLI

>Haliotis-discus_ABO26662

AFSAGLTTHLTLQTVIYDKVFTNIGNAYDNNTGVFTCPQTGIYVFQYHGLSMSDDTLWLELY

HNYNYVSSAYAHTNSDYASAGNSVILHLFKGDTVMVNAEPDSNLYGVSDVYCTFSGYLI

>Aca_XP_005112559

AFSAGLTTMLNVSNVTYDRVFLNTGNGYDSSSGIFTAPYDGVYMFIYHGLAETNGTLWLDLY

KNGDYKSSAYAHITAQYAAASNAMVLELKKGT---------------------------

>Aca_XP_005095519

SFSAQLGFNQPYLMIVFDQVLVNNGGAYDPYSGQFTAPISGVYFFSATILSGFNSTIETMISLNE

EEVSRLYAGSFNNRGSGSNAVVLNLREGDRVGVQVFYGNGDYVHG-KWSAFTGTLL

MSP130 (supplementary figure S10)

>SMP_Equ21150

--------EQTVRNIDFKAFNSKVSGLLPTGVRFVYRENNNTFSADLEPEYIALDPETNKAYVCLQE

NNAVAEVDLGTETVTQVYGLGYKQWG--VLDASDRDLGIQLSYWPIRAWYQPDAIQFVSWK

GRKLVVSANEGDLKKYSNFREYQRGKQFT---LGDVVKTWLQEDSQLDRLKMSKLDGKDAN

GVYQALYTYGARSFSIWDAAGFRRIYDSGSDIEKHTAFRCPHAFNTE--GDDIDEKSDSKGPET
ESLAVGQIGDRMYFFVGNENPGTILVYSVGDDTQPRFETIFCDPDNKKTLQEKFDAREIYALD
PEDLKFATGPESPTGSPVLIVAGSVSGTVSLLKIEI-
>SMP_Cne4282

-----SDSKGPETESLAVGQIGDRLYVFVGNENPGTIFVYSIGDDTKPRFETIFCDPDNK
KTLQEKYDAKEIFALDPEDIKFMNATESPTGYPVIIVAGSVSGTVSILKIDVS
>Cgi_EKC20477
QSKFVYVVGSSKLNVIDINDVENPQIIYHKMMGFDPTDVEFCGDHVFVTLDDNQDSERGR
VVVFTKFKKKTMDTVLNITVGPLPDMVQPSSDCRTVLIALEGKAFARNGELVDPEGGVGL
LKFHDLSINYSYKRLDFTRYNDRWNSLSKTGVRFIYKEQNNKFSQDLEPEYISFSKDERK
AYICLQENNAIAVVDLGTENITAIHGLGFKNWRNSKLDASDKDGGINIRPLPVYGMYQPD
AIHVINVNGEEYLVTSNEGGGKDYTAFSEEIRARDVSISEINQNNISNILDNSVLGRLVI
TTETGRLPDGSFDKLYTFGGRGFSIWKADTMALVYDSGSDVEDTHAQARPDLFNAQLKKK
TIDYRSDNKGPESESLAIGHDGDKILIFVGHERPGSISVYSVNGDSSPKFESVFWDPNSE
QTWTEAFEQRSISVIDPEDLKYISAKESPNGRPLLLVAGSVSGTLSILEVIGF
>SMP_Cgi_EKC42376
AQKILYVTGEERLNVVDLSNPSDPIMLYRKQFVMDPTDVEYCGNHVFVSVANDEKPEDGK
VLVFRKYRHNTMPIVLEVVVGPSPNMLLPTINCQKVLVALEGEAFARGGNLIDPEGAIGL
LKFPSSYITYSYKVLDFRSFNDKYPILARSGVRYVYKENGNTLAQDLEPEHITFSADEKK
AYISLQENNAIAEVDMVLETITDLRGLGFKDWAKYKLDPSDEDGGINIYPYPIYGMYQPD
AIRTVTIGGREYIVTADEGDSKDYSGFDETKRVRDITLANVLQRKIPSIQHDALLGKLSV
STQEGRLRDGTYDKLYSYGGRGFSVLRSDTMERIYDSGSIVEESHAMQYPKLFNSYAKTD
TQDSRSDSKGPECESLAIAYSGTRVIVFVGCERPGTISIYSFNSNTEGTLESIYSGKTVL
GTWGEAFDGGLLTDMDTDDIKCIPPSPKPDGSAIVLPNPRVNSRVPVKPLQSS
>Lgi_111904

-----------------ILVGNMPLMIKPTKDCKTIVVANEARAFGSTNSFQNPLGSVGI
IRFTNGVENYEYTELNFTKFNDKQTDLEKEGVRFVFR--ENTFDRDIEPESITFNCDETI
AYVALQENNAIAIVDVRKLEIDEIKGLGYKTWDKYTLDTSDKDGGIHMNNYDIRSFYLPD

RIKYHHWNDEGIIITADEGAYKSYNSFNEKRRGNWFNPGKLS-YLIAIENFSCRLGRLFF
SKLDGLDNTRKYENLYFYGGRGFTIRKASDMSILFDSGDTIERKISELAPEIFNSNYKNQ
QRDTQSDNKGPECEAVETAEIDGKLVILLGCERPGTIVVFSVGDDKETKFEYLYFNPEYD
KRLKHLYTKRKISEINPENFKYVGFDKSPNGQNILFVVGKSTGTLSAIDVDVE
>SMP_Lgi_190352
KGKILYVSGFGVFHVLDVSNVTDMIIKDRRIYYSDLTDIEFCGDYIFFALTNDASRENGT
VYVLKKYSSDSLKVIHKIQVGSLPDMIKPTKDCRTVVVAIEAEAYGSSDSFQDPKGGVGI
IRFPNGVETPEYMGLDFTKFNDRQPELEKEGVRFVYRENGNTFDRDIEPEYITFNNDETK
AYIALQENNAIAIVDLEKSVIDEIKGLGYKTWDKYTLDASDKDGGINMNNYDIRSFYLPD
SIKYHHWNDVDIIVTANEGDSKDYDGFNEERRGKKFDAAVLSDSIKSDLTNDSRLGRLKF
TNVDGRDSTGKYENLYFFGGRGFTIRKASDMSILYDSGDIIEKKTSSLAPEIFNSDTEND
QMDNQSDSKGPESETVEIAEIGGRLFIFVGSERPGTIAVFSVGDNKDTKFDHLYFNAEYD
KPLKDLYKERKISEIDPEDLKFVPARKSPNGKNILFVAGSVSGTLSILEV---
>Bfl_73459
DNYRIYTVGEAILNVIDISDPKNAALVYQLQLPGGATDVDSCGRFVAVSIHDDFKVLPGT
VLIYSMYTRKNMTLLHQIQVGALPDMVKFTKDCMTLVTCNEGEPGLESGNFVDPEGSASV
IAFQSTNLAPTVRTATFRKFDSLAEEYNSRGVRWTLPMIEFNLSQTLEPEYVAYNSDGSK
AYIALQENNAIAVLDMATATFDDIYPLGSKYWGTASIDTSNEDGG---------------
---------------------------------------VSRRVQKAMNLTSQLGCAVF
SSIDGLDPEDKYSSLHLFGGRGFSVWDADDLSLVWDSGDDVERMVAKYYPTIFNSDYDAA
RFDHRSCKKGPETESLAIGEVDGKTAFFVGNERSSTILVYSLADEITPVFQSIHFSGRTD
LTWRQAYQDRVVGDIDPEDMRFVSTRDSPTNSPLLLVAGTVSGTVSVYEVAES
>Bfl_91441
RNHHIYTVGDAILNIIDIRYPSAPRVVFRQYLPGRATDIDSCGNYIAVALQAEPITRPGT
VAIYEMYVNNDLRLIHTIQVGPLPDMLKFTKDCRKLVTCNEGEPANESGDIVDPEGSATI
IEFSSGFLEPIVRTATFHKFEQHAYRYQAKGLRWLFPEVRFSLSQSLEPEYLAFSHNETK
AYVVLQENNAIAVLDMETASFEEIYPLGSKYWGTASLDTSDEDGGINLREWPIYSMFQPD
GMKSFTHRDRHYILTANEGDNKKIERFTDIVKGRDIMKDSLGARVKRALSDETELACTHF
SAYDGKDRWEKFSALHAFGGRGFSVWDAEDLSLVWDSGDDAEKMISNYHPDIFNSYYKRK
NFDKSSCKKGPETEAVAIGSVGDQTVLFVANERSSTIMLYTLPDTISPVFQSIYWPGKLT
GTWREAYESRTVGDVDPEDLRFVSSEDSPNGRPLLLVAGTVSGTVSVYEVYDD
>Bfl_83665
TDDVIPAPGKAILNVIDISAPSTPRVLHRQKLPGGALGVDLCGAYLAVTLAGDSFVDSGK
VLVYKKYGQTSMELVHDIPVGSRPDSLKFTKDCRKLSVGNEARAGEESGNFVDPEGSVMV
IDFGSEDINPVVRTANFRKFDALGDFYQSAGVRWLMKGLNSSFYQTLEPESITLNEDGSK

AYVCFQTNNAIAVLDMATATFDNIYPLGTKYWGVSSLDASDDDGGIRFRNWPIYGLRQPD
AISYFLHGDTGYIVTANEGAPTKVVSYDEQLKGKDIYKMDLSSRLREALSEDPELGCLRF
SSADGLDPTSKLYRLHAFGGRGVSIFRADDLSLVWDSGDDLERMEAKFYPEIFNSRYDTD
EFDDRSCKRGPEPQSLTVGRIGNQTIIFISAQKTGSVFIYSLDTGITPTFQSVYWGGRAD
LTWEEAYRARQVGDMDPEDMSFVPGDRSPNGTPLLLVAGTSSGTVSVYEVMES
>Bfl_83096
VNKKVYSVGQPLLNVIDVSDPRSITLLHYQELPVTYTDVEYCGGFVALTREHEHKGLPGH
VLIYEAYGTGDMQLVYQAPVGGSPDMLLFTSDCRKLIVANEGKDGGGNGNFLNPEGSLNI
IEFSSDDLTIVKRTVNFRKFDASQDEYAARGVRWVYRGEPNRLSEELEPEYVTLSKDETK
AYVGLQENNAVIVVDLTTATAEELYPLGAKYWGDSGLDPSDKDGGIHIESWPIYGLYQPD
TVKYVSAGGRELLITSNEGNTRELVDISDEWRGMDFVANNIANLLRDALADETKLGVLRL
SNYDGKSASTKYEAFYAFGGRGFSIWDAKNLTQIWDSGDQVERAHALYYPSIFNSEYKED
TMDETSKKKGPQSESLAVGEAFGKTVIVLGNERTSTLMVYTLDTHAVPEFQTIHRH----
-------------------GRFIPHEDSPDGHPMLLVAGSLSGTVTLYRVINT
>Bfl_117163
GQKMVYAVGVELLTVIDVSVPTAARVVHTQELPGKYNDLALCGDSIAVIQSNPLDALPGT
LYIYGVYGFSDMALNNQLQVGPAPSSVKFRSDCARLVVVNEGKAGLSFGNFKNPPGTISV
VDFDINSLTLASYTINFLRFDHLQDEYTARGVRWVYRGEPGRMSDELEPEDFSFDETENK
IYVTLQENNAIAVVNLTTLIVDEIYPLGAKNWANYMLDPSDKDGGIRMQNWPIFGLYQPD
DVVSFTVGSRKLLATANEGNSRELVDITDEWKGKDFITNGVAPTLVAALQDDAQLGVLRF
SNYDGKSASGQYEQFYAFGGRGFSLWKASDLTQFWDSGADVELQHTRHLPLIFNCEFDMD
EKDEASKKKGPETESLVVAEVYGKTVIIIGNERPSSLMIYSVDTVGIPSFESIFRAGDIS
KNYDDAYNDRNIGDLDPESMKFVPAAESPDGTPLLLVVGNISGTVAVYRVVDS
>Bfl_84307
--TAQVKAERGS
VLIYDLYGKSNMELLYNISVGFHPDMLLFTDDCRRLVTCNEGEDGEANGDWRNPEGSVSI
IDFYSEEEPYSVRTASFQKFDSRADEYVRRGVRWVFRGFEFQLSQELEPEYVTFNKDETK
AYVVMQENNAVAVVDLATASVDELYSLGVKNWAESGMDASDKDGGIRINNWPIYAYYQPD
CIKYLEVGGHGLLLTSNEGAVRTTRYFSEVWRGERFVENALAPMMAVALGDNTMLGRLKF
TNCDGNSSRGMFDTFYTFGGRSFSLWNATTLEQIWDSGDDVEARHAQYYPSLFNSEYKTW
TMDEASSDM-----VTYLCELEKCSCPVSGSELSQSSSILDVTAARSPTVTKMLRD----
-------------------IAFIAFHE--DAAPFLCLAVDSGG----------
>Sko_XP_002739468
LNNIIYVIGETLIHVVDVSDPKSMKLVHTVTQNNVLSDVEVCGSYVAITMLDDFVPGNGN
LLLYSLYLKESMELMYMLQVGPNPDSLKCTSDGKTIIVANEGEAGTEFGNFEDPEGSVSI

IKLSSGDLIITQDTAWLNVFNDKADEYVANGVRWIYHGEMTTFSQDLEPESMTLSEDETT
AYVVLQENNAIAVLDMITGEFKELYALGFKKWDTTLIDASDKDGGINLHYWPIYGMYQPD
TIKYFTIAGQEYIATANEGADKEFYTFAEGLRGEDFVLGNVSEAMFLALSDETLLGRLQF
STVDGQSMDTKYEEFYSYGSRSFSVFRVSDMSLVFDSGDEVERMHAVVYPTIFNTKFEED
EMDKSSDKKGPQPESMAIGHVGSKTVFFFGCEKPSTIVMYSVIEGYDPIFESIYRAGELD
GTYEELYQSRQVGDTDPEDVRFVDVAKSPIGRAMLIVTGSLSGTLSTYEVLDD
>Sko_XP_002739469
LYNIIYVIGETLIHVVDASDPMNLKLVHTTIQTNVMTDVEVCGSYVAITMPDDFVPGNGK
LLLYSLYLRGNMKLMYMLPVGPNPDNLQCTADGKTILVANEGEAGIEYGNFHDPEGSVSI
IKLSSGDLIINQDTAWLNVFNDKADEYVANGVRWIYHGEMTTFSQDLEPESLTISEDEKT
AYVVLQ------ILNMVNS--KNFLLWGINNWNTSVMDASDEDGGINLHYWPIYGIYQPD
IIQYFAIDGEGYIATANEGANKEFYAFSEVIRGEDFVIEDVSTEMWLALSDDSLLGRLEF
STVNGRSKDTKYEEFYAYGARSFSVFRVSDMSLVFDSRDEVERLHAEFYPTIFNTQFEED
EMDASSDYKGTQPESIAIGNIGSKTVFFFGCEKPSTIVIYSVTEGRDPIFESIYRAGELD
GTYAELYQSRQVGDTDPEDIRFIDAAESPIGRPMLIVSGSLSGTLSTYAVIDD
>Spu_W4YFG2
LEGFAYVIGKEYLQIIDYDNVEKPEIVYKHTIPERANDIAHCGRFVAYLLQGSDSQEPGT
IHVYEKFQTQEFTKIHERVISSQPEMLTFTPDCRKILTADEGTAGDAEENFVNPEGMVSI
IKIDGDFASFGFKAVNFNDFDERRQKYSNLGVRFPYNGHTQTLSQSVEPEYIAINPRDNV
AYVNLQENNAIALLDLDTELIVEIVPLGNKSWANLQLDASDRDDAIKFQTHDIKSFYMPD
AIKYFEIDGVGYVATADEGSDLDYAYWGDARRGESIHDDNLASALREALNDDLRLGRLEF
SVTDGLSKTDKIEELFFFGGRGFSILRADDLTRVYDSGDEIERLIAEQYPLVFNSETYPD
IADKRSDNRGPECETIEVGVVNGKTVVFLGVDRASVIAIYTVSADGQATYESLHRRGGVN
RTFAQLLEDRDLGDLDPKDMEFVPADQSPTDKPLLMVAGAASGTLSLYHVIGD
>Spu_W4YHS7
ATKIGYSAGDNYIHITDWSDVTTPTILERFAVDTTCNDIETCGDFVGFVLEGPRKTDDGT
LHVYSLYASGDWTKLHEISLGSKPDMLHFSHDCRTIVVANEGEAAQDQTEFINREGSISI
IRLSVDGSTFKSTLLNFTQFNDRSDEYVARGVRYPYHGENDTFAQNLEPEYITYNYDDTK
AFVGLQENNAIAVVDLLADEIEDIYPLGEKSWLNLDLDASDRDDGIIFRRNDIFSIYQPD
SIKYFEVDGVGYIITTNEGEFLEYEEWAEDQRGNDFKAGDFAEELIAKLDDDDILGRLGF
SKVDGLDSSGKFEKLYFFGARGFSIFRASDIALVYDSGDEVAKIIAKFYPDVFSTDTNED
LFDKRSDNQSPEHEALSLAHCRARTPLFI------TLMVDELAHSSVPIGEKLAAGGDSV
ELYRYILE-----ETDHEDVRYISTF---------FLMGNQSSKLCLYTIVNT
>Spu_W4YZG1
TNKIAYSGGNNYIHITDWSDVTIPTILDRMTIDFTCNDIETCGDFVGFVLEGPTKVDTGT

LQIYSLYAAGNWSKLHEIPIGSKPDMLHFSHDCLTILVANKGIAKEDMTELINPEGTISI
IRLSSDGSSYNSTLRNFTEFNDRADDYVARGVRYSYRGQNHTFSQNLEPEYITYNYDDTK
AFVALLKNNAITVIDLTTDEVEDIYPLGEKSWNNLDLDASDKDDGIVFRRSNIFSFYQPD
GIKYFEVDGVGYIITTNEGGSLDYEEWGEAQRGNDFTTGDFSSELIANVSDNDILGRLMF
SKVDGLDSTGKLEKLYFFGARGFSIFRASDITQVYDSGDEVEKMIAKFYPDVFKSDTKDD
FFDKRSDNQGPECESVEIGELDERRIIFVGVDRTAVILIYSLESGVVPTFESVYRAGGRN
DTFQNLLDNRNLGDLDPEDLKFIPASMSVTGVPMLMVTGTVSGTISLYEVYDD
>Spu_W4YJ05
KNHLLYIAGDTYVQIANISDAANPAIIHSRPVSSSVNDIEVCGDYVGYLENGAHKNAQGM
LHLMRAYTSRNWTDYLEIPVGSVPDMLYFTKDCQTIVVVNEGEYDEVPGVFIDPEGTVSI
IRLESGRSGYTINHLNFTAFNERAAEYEAKGVRYSYK--NSTFSQNLEPEYVAISSDDST
AYIALQENNAIAVINIGTETIEEIYSLGLKSWKDLKLDVSDRDGGIVFRNHDIYSMYQPD
AIKYFDVGGTGYIATANEGATVEYPTWTEEKRGKSLVDDGVWGPLISALNDTSQLGRLKF
SQYEGINASGKIDQLVFYGGRSISIRKASDLSLVYDSGDVIEVKSSQELTNVFNTDTKEG
SADTRSDDYGPECESLAFAEVNGTRLLFVGIERLSAIAIFTFPSGAIPEFDSFHRAGGTD
RSYDQLLIDREVGDLDPEDIKFLPYEKSPTGKDFLIVTNNIAGTIAFYDVVNN
>Dac_WP_006002570
ASHSIMVINADTVDIIDASTLTSTALRQQLDVAGGINSVAVHGNLMAVAVENDDKQANGV
IAFYTLDAAGTATFLKTVAAGALPDNVVFSHDGAYALSANEGEPSG---YTNDPQGSVTL
VTISNGIPADSGTQITFSEDDCDAN------VRL-----GTTAAQDLEPEYITISGDSTT
AYVSLQENNAIAVITLATATVEQIYGLSAKDHSGNGLDASNKDDSINIQTYELSGLPMPD
TVANFRRDGVNYLLTANEGDSREYLSWTDEARVEDLLLDAFSD---STVQDEDQLGRLKV
ITTEGDTDGGDYDELYSFGTRSFSIWNADTGELIYDSGDDFEQITAEQGYDGFNNNNTEN
KGDNRSDDKGPEPEALAVGEVNGHYYAFIGLERTGGIMMYNIDDPTAPEFVEYLLN--RD
LEVDIETDLESAGDLAPEGMAFVDAADSPTGNALLIVGNEVSGTTTVYEVK--
>Pseudoalteromonas_WP_008130733
SKKWIYAINSSVVNIIPADTFDTAAIVNTTNLGGDANSIAIDENLLAVAMAAKSVGEAGQ
VAFYDI-SGDTPIFIKNVTAGFLPDMVTFSHDGAKVVIANEGEPNG---YSIDPQGSISI
INVNDGVIADNATNIDFTAYNDKQSELEALGLVFANPAGNTTVAMDLEPEYVSISKDNKY
AYVSIQENNGLAIVNLEDNSL-ELKGLGFKDWSSLQIDASDKDGGVNFKSYPLYGMYQPD
TISSFSWKGANFIVSANEGDAREYLAYIDESRVEDLTLAA---NFDYLNNDDNDIGRLKV
TTVKGDNNNGQYESLYAYGARSFTIWDSNGL-VVFDSGDDIGRITASVHGEAFNNNEDEN
KGDTRSDDKGAEPEALTIGTIGERTFAFIGLERMGGIMIYDITNPYDVQFEDYFYNGLIA
G-------AEITGDLAPEGMTFIPREQSATGEPLLIIGNEISGSIAVWEVSAN
>Gimesia-maris_WP_002648981

ESKRLFVVDGEAVDILDITNPSEPQLFKSVDLASKPNSITAKGGVIAAALSSDPKQEPGL
VVFLSPA------VLKTVHVGPEPDMLTISPDGHWLITANEGEATK---YTRNPEGSVSL
IDLKDGVADASVVHIDFTAYNDKSK--LPSGVRVF----NATPAQDFEPEYIAVSPDSKT
AWVSLQEANAFAIIDLETSKLVDLVPLGFKDHSENAFDASNKDKKIQLRTWPVKGMYQPD
AIYSFEIDGTAYVITADEGDHRDFDGFSEKARVGDLKLDPFPN--AKELQSPKALGRLMV
TSSNGDADGGLYEELYCFGGRSFSIRSAD--QLVYNNGNEFERIIANRFPKSFNADHESN
DLDDRSDNKGPEPEGLVVGMIEGRPVAFIGMERHSGIMVYDLTNPKQPVFCDYVIT----
RDFDKSTKKPEAGDLGPEGLTFIPADVSPTGKPLLAVSYEISGTTALFEVVSN
>Planctopirus-limnophila_WP_013112069
TTRQLFVVNVEVIDVLDLSQPALPVRVDQISVSGIPTSVSARAGRVVASVATEDRRQRGH
VVLIDPV-----KIVHQIQVGYEPDCVALTNDGKLLAVANEGSPNE---YDFDPEGTISL
IEIPDDLANAEITTIDFRRYNDNPE--MGPGVRIF----KATVAQDLEPEYITISADGQL
AWVSLQENNAIATIDLEQKSLISVRGLGYKDHSGNGFDASDRDQSINIRPWPVKGLYQPD
GLANFVSHGKRFIVTANDGKDRDYEGFSERAMVSDLQLDPFPH--ANELQKPENLGRLRV
SKATGDLNGGLVDELHAFGGRSFSIWDE-QFHQIFDSGDQLESIVASQRPHDFNSDHEKR
EFDSRSAAKGPEPESVVVTEIGSKKIAIIGLERQSGLMVYDVTNPVKPVFEQFLSTRSES
TPSDLVSPTSNEGDLGPEGLLVIPAQDSPTKTPLLVVANEISNATVIYEIRLR
>Spu_W4YDJ1
DKQLAYVGGGQFVQIVDFSDVVQPKVVKQIA--GPVADIAECGDLVAFTQPGPHFTDVGS
LKIYEKYATMMMKELCSVEVGSQPIAVRFAQGCSLIIVANEGVMGEYTKKYVNPEGTITT
VRLAGSVSSYVVSQINFHKFNAPAERLKALHVRQPYTGQPHTFSRGLEPRHITLDGQEQI
AYISLQENNAIAVVDLNNNTVIDILPMGVKNWKGLKIDASSADRGIMFQTYDLNSFPMPD
AIEYYDAMGDLYVVTANEGAKPMMGEFEEVEIGEEFIVEELPQPLGQAMAEETQLGSSLF
SMVDGINPAEFFNEVFMFGGRGISAYKVDNMTLAWDSGDVIEKEIAKFFPKIFNGAAFFM
TKDSRSSGRGPECESLAVGDVQGRKLIFVGIDGVSALAIFSVAPGSTPVYESLFKDGHID
ASYNALYKNRKTGDLSPESLSFIPPEKSQTNKPLLLVTGRVSGTVSMYEVIDV
>Spu_W4YDJ3
DTKLAYSAGKQWIHVVDFADMFFPKILDKFDSPRPVTDIAECGRYVAWAVEGQEITDSGS
VVLYDKFVDRKWNRNCEFIVGSRPKSIRFTKDCSTIVVANEGVADVGTGQWVNPETTVNL
IGITEEAMNPTVTTLDFTKFNARADWFKSQLVRQPYTGQLNTFSQGLEPEYVTFDSMETT
AYVSLQENNAIASVDLFANEITGIHPLGAKQWKRYDLDPSPGNAR-GFQKYDIESFRQPD
AIEYTAANGETYIVTANEGKQLEYGEFVEFEKGDEFPEDYLTAVSNAEMLDAFRLGNLEF
SRIDGRSTELKHDDVYFYGGRGISAYRVDNLTLAWDSGDIIEKATAKYLPKMHNGNNRAS
TFDSQSDKMGPECESIEIGDVQGTKLIFVGIDRISAIALFSVPPDNLPIFESIHRDGHID
KSFSELYRTKEFGDSDPESITFIPPEKSADKRPKLMVTGRVSGTITIYQIRDE

>Lgi_152377

NDQIIYVVGEDVLHIVDMENPRAPKILMEHRFLGKPRDVEVCNGRVAVSLSSPVEVYDGH

VKFFTTYTTQTFSYQSQLPVGVHPDNIEFTNNCAKLVVANEGVHGKPFNRFSDPEGSISI

ITIPTTGS-PTERIAGFNQFNSRTD------IRHVSSFATATLAQDIEPEYISISSDGKI

AFVTLQENNAIAQVDLNSGVVININSLGSKDWSDLQLDTSDRDGGVHLRSYPIHGLYQPD

VIKQFKVGKKTYLVTANEGAVRQYLIWSDGVLADELGRGNLNETFVNELKTDSRLGRLRV

SRIDGTTLY--LQKIYTYGGRGFSIWEAGSYSPVYDSGDEIEKKNAASLKTIFNTDCAET

LRDSTSDNMGPKLNAMDVKSSNGTLFMAVGSETMGSVYLYVVDKSITAELQTIKYDGGTG

DLWSDLYDNNMAGNAYISDIRIIPMSQSPTNQTLLYVLGGGSGSIAIYQFSYR

>Cgi_EKC32816

----------------DLSDPKNPTLATTHQFTGTPRDVAICGDEVAVAVTSIKDVYEGH

VYFFNAFGSPGLTPDGKLPAGYFPDMLTFTSDCKKLLVANEGRPGKINNVFTDPVGSITI

IERDRNGGPPSERTVSFTGQDQRFD--LRFPIRYIPQNLTPTFEEDATPEYITVTPDNKY

AYIILQKNNAVARISLSTYEITGVTPIPKKDWSSYTIDPSDRDGGVHLRQFPLKSIRQPD

NAKVITIGRNNFLVTADEGLTTSFFVWSDHSLADILARQNFDTTFVEDLKMDNKAGRLML

STIDGLNLFGKMDEATHFGGRGFSIWKTSDMSLVFDSGDEIEKELANSMKAVFNTDCIEN

LRDTQSDDYGPKIGSLDYINDNGAQYIVVGSETTGTIFLYSVNTTPKPQFETAYRTGDTD

YVWSELYDMGTAGDAGLSAVGFIGRDDNALNKTLIYVIGQYSGSVSLFQIVNM

>Cgi_EKC21174

VDKILYVASARYLHIIDMNNPASPSILMTHVFDGMITSVKACTDTIAVTLTAADPVAEGH

IELFTPYADRVFTRVGRIAVGVHPKDMAATSDCTRLVVANEGPVAIVSGTLSDPEGSVTI

IVRNDQGF-PVEINMGFQQLNDRVVDYITNGVRYVFRGDVNTFSQDLEPESVTISNDDRF

AFVSCQENNAVLKIDLFNQRIIELYALGAKNWTSYNLDASDQNDAANLRFHTVYSFYQPG

KLAYGVVDGKGYLVSADTGKSKTLYAFSDNTRARVAYNDGLDITLLSQIQDNQQLGRVHM

SRVDGYNIFNRIGDVFLFGGRGISLWDSTTMAHVFDSGDDLERRASQFYPNTFNGDCSTQ

QVDERSDDMGPEPQALATGTVGTTPVLIVG-SRNGLIYVFNMRG-VSANFESVHREGSTN

DIWNNLYANDAAGDQMISDIGFVGAGDSPSGTPFVYVIGQATGSLSVYNVVDV

>Lgi_159824

QRKVLYVVGHDMLHVINVNNVDSLTTILSHNFNGKPLDVEFCSTVLAISFSSLYEQAEGH

VILYEHVGNPVLVQKNQITVGPHPENIKFTSDCALLIVSNEGIPGEYDGKFVDPPGSVSI

ISVTVANT--PISTVSFSEVDTEPQFLLNNHVRWMLRTEINPFSNNIEPEYITISPNNAY

AYVTLQENNAIAKIDLENGYVNQIYPLGVKEWRYSTFDGSDGDSGIILKKHNISSFYQPD

KIAFFEWKNRLYLITADEGKEFSVYFYTDFDRAKNLHTNGFLVELDAELADDAQLGRLFV

SQFDGRATGSKINRVFTFGGRGFSVFDANNMIRQYESGDEIEKYSRLFHPNVFNGDCSFQ

EMDFRSTLTGPSLNAIVDGDFLGRKLLIFGSGTNGILYVYELVNPAEMEFQSVHRRGSTL

DTWGKLYSSGIIGDAGIEDLSFIPQENSPTGSPVLLVVSSKSGSVSAYTFKDQ
>Lgi_159825
TRRILYVIGEQMMHVIDLSNVDTLNTILTYNFNGKPLDVEYCSSYVAVSFESQHPQAEGH
IILYRPVGQPSLAPINSITVGPHPDNIKFTSDCSKLVVSNEGIPGTVENNFVDPPGSVDV
VVITSPTS-STVERASFSFVDDTDKELLNAGVRWMVRANINPFSNNIEPEYIAISPNDAY
AYVTLQENNAIAQISLESYTVANIYPLGSKEWKYSTLDASDMDSGKYLKKHDISSFYQPD
KIAFFEWKEKLYLITADEGKQFNIYFYTDSMLARNLHANGFLVALAAELANDTELGRYFV
SQFDGRVSG-KIRTVYGFGGRGFSVLNPSTMTREWESGDEIEKFNRLFYPDVFNGDCTFQ
EKDKLSTSTGPSLNAIIDGEFNGRKLIVVASGTNGLIYLYELSSTAELMFQSVHRRGGTI
DTWGRLYSSGNIGDAGIEDMSWIPIEHSPAGSPVLVVVSSTSGSVSAYTLTER
>Lgi_27977
NSRFLYVVGKFVLHVIDVANVASPAVLQTFTFDGYPLDIQVCRNYVAIAFGEQDPSKQGH
VILYSPYGETALKPINRITVGIDPDNIKFTSDCNKLVVSNEGVASVISGEFIDPPGTINI
VTIPLTGN-PGVKTIEFTYIDDPAERLLALGVRYMVRTNQNPFSNNIEPEYIAVSPNNAY
AYITLQENNAIAKVDLIDDNPATIFPMGWKDFQFSTLDLSDRDASVHMKRRRVRGLYQPD
KISFLEWGNKLYLLTADEGKPFNLASFADNARARDLENEYFPWQFLASVLDDTQLGRAIV
SLQDGRVG-QQITEIYTFGGRGFSIFDSNTLTREWESGDMIEKFSRWFYPDVFNSGYTID
ERDTQSPFGGPSINSLAIGTFKGRTVLFFGSGQNGQIYTFQLQASVEPEFQSVHRRGQID
GTWTNLYSRGVMGDIGISDLIVVPEAESNAGTPILIALSERSGSVSIYTLIDQ
>Spu_W4YL38

------YVSKKWNLLCDAKVGADPVSLKFFHNCETIVIANKGTPAATNTFTDPEGTISI
VRIVKAQQRMTVTTIDFRRFNTMTNRPFLNGVRMPYDGSRPTLSKALEPTYVTGDPTNDT
AYVSLRANNAVVQLILTNEAITGFNPMGMKTWFNNDLDASSTPRGISFNKYNMYSMRQPN
AIESFEIRNQSYLFTADEGATTSYKSFTDAVPFSRLQNRN-----------------LEV
SRVDGLRNPSGYDYVSFFGGRGISMFKFNNLDLVWDSGDQVARGVAETYPQVFNSKSTKS
QRDLASTERGAQCKSLAVAKVDDTTLVFVGADGPSVIGIFSVSQNSYPAYESVYRKAPMD
GEFSILMDNKNMGDIDPAKLIFVPRSATPDNYNYLMVMGKKSGTLSMYRIFRE
>Spu_W4YDJ2
DNMVAYTGGHNLLHVINFTDVVRPRIVRSLRVLDPIIDIAECGNLVAFTSRPRSRTDPGM
VHIYQKY----FGSLCNLTVGSNPKSLKFDYKCDTIIIANEGPAAEEQEMFVNPEGTVSV
VRINNTFVGSDVRHIDFRLFNDPALLMEQQFIRNPYKGQQHTFSMNVEPEYVTLDPWQPT
AWVTLQENNAIAEVWYENMTAI-IHPLGLKNWTNYGIDASTMDAKIKFNRYPIESLLQAD
AVEMMDINGEVFLITANEGAPLNYTEYTEMEEAAELAGDYISEEVRRAMTDPARLGQLRI
SLVDGLQEQEMIDRPVFFGGRGISMFKVNGLDLVWDSGDSISKGVQDLHPNAFNHPPFNR

RWDSWSPFMGPECQSPGNGPYNTHKVIIVGIKGAHVLGIFTVPLLTQPNFESVYRDGNVM
KSYNQIFRDHGIGNLDPDSIQFVPADRSRSRPPMVLVTSRVSGTVNLYRISEV
>Vca_XP_002958226
FSKLAAIVEARALLIVNYANVSSPFIHRRILVGGTPNSVAVWNGFAALTMDGVPYTASGI
LRIYH--ASGAKVAEAPLTGCSMPDSVKWSKDGHRMVIACEGEPTTVQGVEPNPSGAIAI
AYVSVSSYSISIKLLDYQGYIDSLSALLARGFRIDPRLTKATAAKDIEPEYVALHSDVNL
AYVTLQENNAVSAIDLTSERILSIWPLGLKSWKASPVDPSDVD-GIRIRNHTVYSWYQPD
TIVHATIGTGSYLFIANEGDSK-----GESRRVKELA---LDP------TQDSELGRLNV
DPLFGLKKGGPYNKLIAYGGRSWSILDAKNGRLVYESGSSMEAIFAAHASQCFNCDRDRN
DPDSRSDNAGPEPEALEVFQLGSRTYAAIGLERMGGFLLYDVSVPSAPVFGAYIYNRNFS
APRTA---TSALGDLAPEGIRFVDAKDSSSGTALILMSNE-------------
>Cre_XP_001702318
FSKLSAVVEARALLVVNHADPANPSIHLRINVSGVPNSVAVYNGYAALSLDGVPSTAPGI
LRIYN--ASGVKVGETVLPGCAMPDSVAWTRDGGRLVVACEGEPATVRGVDPNPVGAIAI
VTATRTGSSLSSKLLSFQTYIDSLSTLLDKGLKIDPRLSKSTAGRDIEPEYVAIPSDNLN
AYVTLQENNAVAVIDLTRERIKSIWPLGWKNWSRHAIDPSDRD-GTRLRTVPLYSWLQPD
TIVHEAIGARSYLFIANEGDSK-----SESLSVKDLTAAALDPPNATALVADAELGRLAI
DPLSGLKKGGPYDKLFAYGGRSWAILDAGNGRVVYESGDELEQLTANHASRCFNCDRDRN
LPDTRSDNAGPEPEALEVFKLGSRTYAAIGLERMGGFVLYDVSTPANPILGGYVYNRNFD
PALGS---AAALGDLAPEGIKFVAAGDSNSGKPLLLLSNEVSSTVSAWEISNC

Supplementary file S2. The sequences of shell matrix proteins and dart matrix proteins in *E. quesista.*

>Equ00588

XRYQWRDFWPARQGRGSTRQITQLRNTDQVLQTRIFNQSQRYESLQQQVLALTQKNVILLSR
LAELQQQIVLFQHYIKASQVTTVVRTTTRLSQVQCTRGMVHTQTRERVVVNGIPVMCDTETD
NGGWLIVESVRMMMRTXPNLGXLQEWIWXNHRQLLGWAGDHLXIHFNGIHXAQGGPGAX
REKLLRPLRCLRCGKRTGQLQTVRKWLQWGCRRQPVLPQRVWVXHERXRQXWXQKKLRP
DQWRRVVVXFLLP

>Equ02505

SYVGDEAQSKRGILTLKYPIEHGIVTNWDDMEKIWHHTFYNELRVAPEEHPVLLTEAPLNPK
ANREKMTQIMFETFNAPAMYVAIQAVLSLYASGRTTGIVLDSGDGVTHTVPIYEGYALP

>Equ02555

AILRLDLAGRDLTDYLMKILTERGYSFTTTAEREIVRDIKEKLCYVALDFEQEMATAASSSSLE
KSYELPDGQVITIGNERFRCPESLFQPSFLGMESAGIHETTYNSIMKCDVDIRKDL

>Equ09762

TLKYPIEHGIVTNWDDMEKIWHHTFYNELRVAPEEHPVLLTEAPLNPKANREKMTQIMFETF
NTPAMYVAIQAVLSLYASGRTTGIVLDSGDGVTHTVPIYEGYALPHAIMRLDLAGRDLTDYL
MKILTERGYSFTTTAEREIVRDIKEKLCYVALDFEQEMATAATSSSLEKSYELPDGQVITIGNE
RFRCPEAMFQPSFLGMESAGIHETTYNSIM

>Equ10628

HRLQSGECLKFTXKFIXFSNLKRKKEHIFFRNTSDNSRQLNXXGNNKSYFQNRLQISKTKXQR
NKLIKYTKQNKTTNIINLSMAMLRYPLAQVAAASCLLVFLSPLTAAYGVKTGADDTEVETWL
ESLIKLYQPTSDDVGQQQQTHYFTQDQIDYFKNVLNKMKAGIVKNDLKQEIVDHSFLKTQKK
KLHKKNHRQQHLQTPKNKLHKTQKNKLHKNNRQQRLQMTLRSKLYKNNRQNLLXMTLRN
KQHNKSNRQQVLQNQKNKANKNSEEDTTQQEQQTTTPADDSEENTTQQEQYITPVDNSE

>Equ10634

ELCNNCVVHHGVGYAPYPGYCDAYIQCRFYGALPTSVDIRRCPAGNFWNQDKLTCDFEANV
KCTPVNGCPNHKAIPGDWAAYLINNGQNWTRVSCPEGRLYNSVTCGCTDKHGGDDKSQEN
CYDKKAILDDKTGYMQFTGNGWVRMACPATVGYNELTCRCSDNLATQTSSTCPNTKPIPGD
RSGYMQFTGVSWISRPCPATLVYSPEHCVCTYDKTNAVDNDDGNSKRGLCKSTLILNFDNND
ATDSSTNKFWVNNTGVTFKDGKAYFNGKSRLTVPGFSNVEFGISVYIYIEYRHSSANSKQTLV
ANGDCQVRQSLAVCSGKDSVDFYAETKDQISLGKVTVPSDVGVWQNAVYALDNGNLVGTV
GTNTIVQPVKGALDRRQRGLVIGGGGDCDNFHGIIDEVQVFLCKPELXKLKWNQDXTNTYII
NYQSVQSQXKEIHNVIVVVSNXSHKFLXILLLEKKLSHVNSLSKCQLPLXQVLCGITXNNWA

>Equ10941

GDDIQTLSSASSDFSQHLYQKIALDKSNVVYSPYSIHSALSMTSLGAREETATEFSTVLGLAAL
TGNRAHLAYRDLINQLNSVTDVKLNTANALFVNPNIPIEQQFVSDARTYYLAKSSNLTWPRW
EDRRRRSMITWRTRHRESSRICFLKEAWMPVQXRFSSTPSSLTEPGRFSSRLGTHRPRTSRNW
AGPNLRXAXCMMKGTXTSRE

>Equ11259

GAGQFGGAGQFGGGGPSPLGGQGAFGGQGAFGGQGAFGGQGAFGGQGPFGAGGFPGGFPG
GFPGFPGQGTFPNFGQSNTFQTPQILTCIGSNEATDQLKITLRPEPFSSLPQQTQQRRELQRQN
WRISAMFTPAASLGRISPAGTTSLVGQFFIAVTRYGRTDGSCAGLGGILQNDDTIFGNSNDPFT
NNPYLQALRNQQNMGQPAGYIQDPILISQGGNVYSGVVRDLTEVDLRGRGIAVCLDYLCQRP
TTTCCTVAKDSSPATEQVGPVVNTGPVTGNVLAGGSAGSATAAGSSAGLISGGTGTPGGLFX
TTPQTTRVQPQTTRVQPQTTRVQPQTHGATQPLIVLPQVPTPTHLFRVQLNQFYKSXSFISVNY
PAVNYCDTMFLSYIQTLSMFKYVSVLSVSAQRVTVRLKYMNTXPKLFVLVVLVIII

>Equ11340

DKDNIHLLGCFPCHIRXCTVNETTCQDVCIXFPDIGIYKCXSISTKHAYXEGVAEPFAFSYESNL
TIVNISXFFSPFNWPEPFRNDTTATQLRHNCVCSASXLLRMNTLRLFVTLMLGGLCEAKISEVP
LPENVQKCYDKVQDADTNTYVGNLFGWMCESKIRHSELGKHVQWDAKKLSYYKRLLKSAS
PPNNTDGGATVPRRKRQATQTPCVRQEYRMLSDDRRTRYHNAVNALKQDTSVEPNKYDAIA
LLHVLYAFTAHLGDGFLGWHRLYMNMFEAAMQEVDPSVCLPYWDTSLESELGDPALSNLW
TPEFMGTPNGPVVDGPFANWNTPNGGPLIRNVDSRVDLVTATNIRNILTRTGYEQITFSRARP
RYNLEIFHGGPHVHIGGAMSQVDTAPFDPIFFLYHAFIDHIGSSSEQDSGLLAGTLNGTRIXSLP
THPTTGQLQLALIICSRRMHTGTSSLRDLYTNLLPHVLPVGHNAVQDTXPVEXVXDAVCPLQ
QAQPGHLRRVQQSLKMKRSHWRCCLTLQSLKVQWFQRPNCQEELPVTPGAXNQRIPSVRLF
RTTFAVTTRATXMTGLXSSENHQRSASHVQQIQKLPSSQGSDRQPLXHLLTTGLQPDQQIHYP
ASEXPXNIXTLQTRRKCWPSV

>Equ12236

QDISLLFEQVYEKIPSLLNVMMDAAQDPRAGKRVDFGADVMKRMIVNAFTSFLPVCAQQHR
RNEQGELLKTNIPAWYFSSLRSQNPPSRSLTKGISQKQSQDSIPLKLRRRKRGSGSEIPSDVIIIPL
DSLQS

>Equ14131

KRSHLPSVXNYPQFIIRNTIELLELPDTNRISRXTRIMLIFMAILFGAVAGQTYVDPVPRCYQGL
CQQLCLDTVNGYQCRCLAGFYVNPANTRTCIPVQGCPVPNR

>Equ14133

RRQLLSVWLXKWILPRPTEPLQLSCHVPSKSTRDHNHMWTRIYGGPSKPQRMQRHXXMPNT
KQMPTXVXQSTGKLPMHMQYRICRQCXRXHKMQGIPTVHCCSRCDSCGGFHRRDGLQQLQ
LLLSFVSEISQHFQIGPKRARFGALLFSDAVQLLFGLDDYKTSERISTEILAAPYLGGNSINFEIF

DYIRKKDLFNIESGGRPHVHDTVIVFTDGRSSTVDLTRAAAENLKNTGVTIVVVGIGNGVSAS
ELASVASTREHVLLVPRLLPHXKHSGEAGXAHLPRNRXLKPCSFKTIMRCQNAHIPKYXRTQI
IEEKSKXIKHGLALCKK
>Equ14346
FLAVIVVLALTIALVQGFVIGGRDGFGSFRGFGRRTDGFGDYGKRGGDYGIPSGFGDFTGGFG
GGWGGEYGGGFGG
>Equ14346
SXLXLLSXRSRXHLSKASXLEVEMGLGVSEDLDVGLMVSVIMEKEAAITASLVDLETSPADS
VVDGEVNTVVDLA
>Equ20976
LCIYLGKISNVNNEDYFSYLPGLYAGCSWRVPRPDGTHVPSHGHRSPDVTHYASKPTDASLPT
PTTTATPGSAAAAAAETEVHGLPDISATAPNQNQSLHALQWVLHRSQRGQPRSNSVYSSANR
IRVPGSSNAVGTSKGNPYGSSYDGATSKGNPYGSSYDGAANKATSSRGSGNPYSISRGPARAV
PSLRNPASFAPVPIPAHATRLMRTMKTLQAVREQPMKITDAQSVGCRLPSEAMASLVMYSDC
RNAAARMVCQAEMIRASM*
>Equ20977
QSALGTTQGHFRRAGRNANQHRKHGSPTDRSSAACYVSCRHISQGGPGGLGGGDGGFGGGA
GGFGGGAGGFGGGAGGFGGGMGGFGGGPGGFGGGPGGFGGGQGGFGGGQGGFGGGPGGL
GGGPGGFGGGPFGPGGPGGLGGPFGGLGGPGGFPGGFPGGFPGMFPNFGQGQFQQPQILTCIG
TNEALDQLKITLRPEPNANFPFPPMNQRREFQSQTWRISGYYVPAGSAGRANPPGTTSLVGQF
FMAITTNGRTDGSCSGLGGILQNGDTINGGNDFTQNNPYLQALRNQQFGQAAGYIQDPIVISQ
GGNVYSGVVRDLSEVDMRGRGMALCLDAMCQRPTTTCCTIAKDSVAATEHLGPVAGTTGA
FSLGTGVQSFAGVGGLITGLPGSRPAGGNPLSSSSAGSSGLFXRGGAGKDNXLTXERTNYNRX
XKDLSANSLXXLLCXRRCGKFIQYLRNSCTLVXXDIXRSANIXGSENXVGLKNTFKIXRXLAN
LVGKGRRRQIHLHTYSLAKMVXSEQERSRKRDWXSLEMANNNHPRMWAWHTLMTLKKQH
FTLVVFSVLEDDFSFSYXYCQTEXYESSXLSQQGIRDXEEVLGLCIFIVADDMFLYCLVTIIIPIV
NNISTHXLSIQXTIPHIYWAINILYGWLF
>Equ20990
GGSEQVSSSSFKKQSVIMGKEKTHINIVVIGHVDSGKSTTTGHLIYKCGGIDERTIAKFEKEAQ
EMGKGSFKYAWVLDKLKAERERGITIDIALWKFQTDKFYVTIIDAPGHRDFIKNMITGTSQAD
CAVLVVAAGTGEFEAGISKEGQTREHALLAYTLGVKQLIIAINKMDSTTPPTVSLDSRKLKRR
XAATSRRLDTTLLLYHSCPSLVGLGTTCWRSLATCPGSRAGVLKGKKATLLARLXSTHWIAF
FLQVAQQTSLCVCPCRTSTRLVVSELCPWAELRPVSSSQAWLSLRPCQHHHXGEISGDAPXGS
YXSQPRXQCRIQHQERVCQGHPSWQRGWXQQERPTQGCQVVPCSGYYSEPPRRDQERLCAR
AXLPHRPHCLQVCRDQGEMXPSFRQEDXRQPQVCQVRXRRHGXPGPQQAHVRGGFPGVPPS

GTLRRAXHETDRAVGVIKTVTKEDPGAGKVTKAATKKKXKLQEPWPLSLGRNDQHKSGVW
DGILTHNYQHNSSCEGLTPVLTPFFSLIPIKKLTQLILISWSRWLLQSKLHRKEVRRKKHLMFI
MCIXFTIHCERALSVNKVTVSQSLLAPFSGPASLMVFIISAX
>Equ20996
RVDRXEKGNICEDISECCFLLHNRQWXKCSDLERSLLLLXLQCDMSKDRRQETTCPLVHCGP
SSLSFSLHKLDAPSRTLISWVXAIPRLTSCGRLTQPLCRKXPRREHQHXSHQRRRHLCLTSTSH
PTPSAATATPTRASSSSSSRTVASRANTPKLSSSALNALSYNDILSLINQLSSGGSAAGGASSAP
TKTTTTAQDVMSAYAQLSGSGSSTSSNTAQGNTDYQALLQKYMAGAASTSNSRSSGSSSSSP
SYQSLVDQYNSLVNQGGASPGSSSSSSTSYEQLLQQYNQIMGKASETPSTASKGSSTSSMLEK
YLALISKPGTGTATTSDQLLQVYKLMSGGKTPSADLSTTTPAPSPAPPQETFEKQLATSSPSLR
VCSHSRQLPTHQHRHPAVDPPLQVSTQQRVIPLKXFSTLCSSRQINLLQARLLREHRLEQRRRS
HSGSKFXHDQKTSEXFSSQLHPEYSELSGSSEAILSNHVQNVFKTRASREMGCRFLPELLELDV
LPTAPVNCMNQCPFPYVNPQQYGMYCVCCPPGVNEQAVGMMSLMRGTSYLETPLTVVSXSL
VNTSCLLIVKK
>Equ21047
GIGSWHAAXDGTRXLSASLQVLLIINNGPSIIFXLRKSRKRCFQQRLQLGFLQVXRQNENTKQ
CRIQSSHQSNRDTGKVAGNLETKYKWSEYGLTFTEKWNTENVLNTEIKIEDQIAEGLELAFDT
SFAPQTGKKSGKIKSAYKIDYVHLNADVDFDFAGPTIHGAAVLGYEGWLAGYQLSFDTSKSK
LTSNNFSFGYNAGDFSFTTHVNDGQEFVGSIHQKVSDNLEAAVNLAWAAGTGNTTFAIGGKY
KLDSDATFSAKVNNQSHIGLGYTQTLRDGVKLTISSLIDGKNINQGATRLAWDWSLKPKYET
AQVTVFGSXYSVXDWLKCTACNRRYPDLFMVHVHCLYAACSPVFLIAECDYILTSCLXAVY
NTEYVHVKQPYPLIFIFSTSTPGVMFFYKLEDKKNVHXXLIDQLSVIYTTXMLNNRPRSLXTLT
XVDSLADDIALFXFPKFDYVYISFFVXRSAFICGXDSDHHIWQTRXNLNLRXVVFVXKTINGD
FLXKK
>Equ21122
GKMSLFSLSQCVHASADRKDVCVXFVSVCPCKSGRKDDXLNSIELPKLWCTVTCDDFSSVTP
PTVPEFSEGLTLXSAHIRXLFVRADKGLSPTETNGQTCVNCRFASWFAVCKFSNSAACSNRXS
TCVRWDRHWCSSPGIVPGQGTRNPREERTGISRPGFPLXQTGQRNXVQTHRGERFTWDHDLG
TGETVSERAFAPTANKVIVVKSRDRALEAYNITRSTSVYAFSDLSSIVAVIPWRRSGPCLVRNS
SLTYQSAKDLLTARNGSTVRTQYRISVDGNVAPLSTEETTTFXPPTHNCDVPVARDKLSSPQK
XETTQLHSPTPRKRSPPSAWRPRCPSLCLSFPTSQTPVNIPTTNDDLEVKATAGVPGLLDSTTTI
AMTFKLHVSRCHSISSSTSYIIKLHHSXTVNCCINICGXHTHTHTHTH
>Equ21466
VVSELEVFMEVVIMFVMLTFDDAVTSSVFSQIYYNLMIENTYKLFNPDNCTIRSTFFVSHEYT
NYNFVQHLYQAGHDIASHTVNHTSSSSVYEEVAAEIVGIRQQVKANTNIPPEEVTGFRAPFLRI

FGDVQYDVLRDYNFTYDSSLVNIEIQIGRKPLWPFTLDYPIPSDRCPNKPCPERSHPGVWEIPM
NGWVGDNGYSCGMIDGCSIKGANFNGTVDDFCDITGATSVFLQGQSPDAYVHTRSNVYEVQ
RSIPGSYNIHTXTVGHEQRVVRDPKPSNLLDEKCSDKCRLKSXRLELQAAGLNNKLSTNXKK
>Equ22322
RQKTXNRNCSKQRSPDAPESXLSEDIIMLLAIFSICAVLSSVSCDSEAHAAFSALINGNVYYTA
GNIVRYPVVTTNNGNNYNPATGVFTAPATGFYFFHVNAQAQLEKVLALGLYHNHKYVLSVY
ASSSKQPHWANGSNAIVLKLNYGDTVCVKARGPSFLYGLPGPGDCCIQSSFSGFQLAAXARA
YLYLPQXTDPGVAXCELAPVVRMELNPXQIHSQHLLIKK
>Equ22329
KQTRFQVKFSRNPHLSQLSVXPCYXTPLSCLSSYLQCSLRFFVQRSKASEMRHFAAIKTGYTL
GKEQVVKYDTVLTNVSDAFDKSTSIFTARKRGIYVFHFNTLANINSHCDMGLYRNNAAVVG
VSTNVLSGYPMGGNTAVLRLERGDRVYVKTYIPCNIYGKLYTSFSGYLIELGEGEEXTSMQTS
QDSTTXTIGQINKYVKIXNVNXYPKLQYIKNSFXXNYK
>Equ22594
SRQDLNSQWPSHLGRYRTVHLSYLRWXLSTPIMLAVVFIGLSLAVISQAIVFNDNMLMYYM
MMNPQFGLFGGQEGGQSGMPLFLMASGNDNMKRLGQSFLLSRMLMGGGGGAPAPAQGXTI
TNYPHDVIHIPECISFSEFRHNXLMASYFXKLLXLQIYIDLPKSQVFEMIIQSLSLKXTGFDSFL
MYRYSLLVXIKQNNILYIQYETLKK
>Equ22616
ISFTIFSSSPCHFIATTPRTSFLLHASAHLKTKNADFCEDLTGKTITLEVEPSDTIENVKAKIQDK
EGIPPDQQRLIFAGKQLEDGRTLSDYNIQKESTLHLVLRLRGGIIEPSLRILASKYNCDKMICRK
CYARLHPRATNCRKRKCGHTSNIRPKKKIKXTLPFSGIPQWPLQCTXDXKQVPSCLLYGVLQS
XSKVFTINCWFNFKK
>Equ22744
HSGIPNSLDMFSNNPAARTRTVTTVSQNTIKSYFPKYGLLVELDANGRIVGSLHDPTGQVYSAI
SEAAENQGLLYVASPSNFFTGRIVLPQGDGRVVTLDSVIQVMRSRCQIPEDKLAQARAVLNSY
IEKQTGSPAIAPQGAQTAPGSSLQPRAAAAHPSPRDADDVNWSDSYHPAHQSASPRLASRRX
YIYSLHFSIVTXTN
>Equ22829
GLSVLSGRPREVTYCSAALPLHPPVKSACXAGAFRDSFXNNMATAGFIVAVSGFICFIAGVAS
QATDASSHCAYLINSVSRYQQCCCLXDLHXVKVTADLQWXTYXSNYRAIQQLVELLQLHRL
HPVRHPVQRGQPGDRVHRHRVAARGVFQLFDKWRAGAGGLNCNPRSRAEDSVGAFEDRLL
ATFKTYYPNNPMLSRYH
>Equ22899
ADCLNRKTSHETHSKYHNGRHEESXRPTAAVYWCCVPHSXWVPEHIXSLSLQKSSRRXLRLC

PPACQYGKFITCSNGRALKKDCPLGWFKKPGDSPKNRFTKPLVYDPYSKYCLQGSQVCPRKY
DDFIERGKXIQWTPLKTLMNIXLCNLHSKISIYLSIYLSIYLSIYLSIYLSIYLSIYLSIYLSIYLSIY
LSIY
>Equ23501
XXQFWALFDVALLSDTYSFSTMTSALTFTLIILASMTVARAQVSISKLAFRYYQNEAVMLGSQ
TCAQYGRSCVNYPCCQYENRGPIDCFSGQCLQAQCGYPGQQCGSGIGECCYHKGLSCVTRK
QTWLGICSDEDDXTNLFNGPFTHGIXNTSTVSVPLNXLIIGINIKSDMQKKN
>Equ24576
LPAALLRSTNASSLTRNMKLALSIAAVCLAIVCAFAQTPEQVKRNEINAVINTLTPSCKTAVQ
AQSTGVDTLEAACKKLEIFRGLMGIVGCTTADYNKLNAVLCYLNGVYVGXTXPASNARNM
VCTXVRHDLQAIPEXLMTMTRFNXNLSPKL
>Equ25494
YRYRRSPPTSLQTLLQPSNTTSAFKHYFSLQTLLQPSNTTSAAAIAKTEKEPXPIVLVRQSPPDC
SPHTMTLHWLRTGHIVALLVVFFSATSLLQTAHGFKYHEFCATPPGEMKTRFPLYRLEVDTKI
EGIPWAYRQNN
>Equ25690
XVSCMQPGTTSFPLSLRSAISTHDENRNNLDCGSMCAGYLHGTGSSRGGFGPSQAPNPLQLL
MYRSMFDMNLFPALFLSGHLGGGGGGMGSNMLFPLMFMXKTTHMIYHTRTHIRVNHKYILV
RIYHNGIHLVQIXN
>Equ26376
GRSLLVAETGRARILSISLERTGQRPAPTIFTDNLPGVPTNIRGTSHGTFWVGLSLIRHSRIANS
MDQYSNNPAARYRTYHRDSLNQITKYYPSTVWLWNQCTRRHNXKHSRSLRYSLLVHXRGC
RRXR
>Equ26417
PPARNQVSVLWWPTKEALLYPEIKFVANIKSMGNWFKLQSTPWKVNRPVDQWANTESMLA
QYQAMQNNMRAMERRLEQPVXIGGKQAQATHAFVAHGLGFFENKQQNXHESILNNYNYSN
LNXEIRKLX
>Equ31647
LLALSGAFAAEPGLKITTTTKGIDYANQIAGNALRSKLQTITIPNQSGSQGSKSWNINNIRVTGI
TGPDTHISLNPGAGGVTLGISSFGASCMQTGT
>Equ32691
TSEVILXCNILYGCTMQIFVKTLTGKTITLEVEPSDTIENVKAKIQDKEGIPPDQQRLIFAGKQL
EDGRTLSDYNIQKESTLHLVLRLRSTG
>Equ53877
RVSPTSHPPSGPARRCQAHLRLIYEETRGVLKVFLENVIRDAVTYTEHAKRKTVTAM

>Equ16217-21060

PPNFKPPDLKPMLPPPMPMKQSPFLPPPMPMPMAPPGPPRVPTPNDGIFMDMPNAGGGAYGA

GAPYGGGAPYGGGPYMGPPVMRSYHYCPPGPTTADHCKDQKLQEALYFPDGTPRYNWVPP

RNPWDTSLLDTVKETAKNILMMKVNSRPSRTPTPKEWELMSLLGDPKEQGPAGSNPFAGRX

>Equ15522-15523

MGVLLVALLLCALITVAVGNASGSQRFQADKFLDMLGRSFDPKVAYVDLLAELTHRLNIDV

NSSSATSLASLIGAKRASAGNDKSACQEAPKVKGTTVTYTSDIHDVGSRAFYQCDTGYDGMS

KFIYCTQGGQWSTLASWEGCSPVDCGLITPHVTNADPTFTSTTYKSLIFYACHAGYKLDQPNG

NVSCLDTGFWDKPKFSCEKAAAPSLRLPSLQKAFNKNMGVQKIQQLQQLATTLLEATPSPPVI

IKPQPRQNLPAPLARKKTRYLRSLGPLTSSEPQSLPSSLTSSTANTSNPENPMMEAFMKSMQLF

HALPKMPNIEQAKNSLAVPTVSSFANPMAMFAHMFPTSP

>Equ23617-24364

GYFDVCAQLVRVVVPPAGVMGLPDLEAGPEKSPTKCLKKSFKWAIVGGLTGALGITGLLVGL

HLSGNLVTDKKPETVSAASQTADTLAPTFVNSAANSLGAAMIASSDGVKDTVSNVVDGFKD

AAGNVGDAVANVGDAVANVGDAVANVGDGVKDIVSDVAGGVKDAVGNVGDAVADVASG

VKDVVDGVGDKTKDIVGDVGDVVKDVVGDVGDVVKDVVGDAGDAIKDVVKDVVGDAGD

AIKDVVKDVVGDAEDVITDVSDAGDAVKDVAEDVGDVVKDVVDTAGDVLKDVVDDIGDA

AKDAAEGVGELGEHISEGFSEGADIFXEPRRNQALEGN

>Equ44650-23959

WSTTGRRDSSRWAVGGVTNRGRGSDYVSLKWFADPCWRHVFTHDSSGNQLRGSRESLVAAI

KDGHRVRVVVENKAMEAAFIRLKNNHVSAYFLDELSSKGGQGFDQFDFTTDTYYKFSTTHT

TGTFRQYGHFVRNTSTTVTPSLTKQKISWMIDVKPWETVLKVNDKGLAIWGQKQNVKSAAL

KAAAIRMGIQFDSSSGTLYVGADNTKVSTTPTDEDTVAQSVRVLDDRPIGSFNH*

>Equ04504

EEHPVLLTEAPLNPKANREKMTQIMFETFNTPAMYVAIQAVLSLYASGRTTGIVLDSGDGVT

HTVPIYEGYALPHAIMRLDLAGRDLTDYLMKILTERGYSFTTTAEREIVRDIKEKLCYVALDF

EQEMATAATSSSLEKSYELPDGQVITIGNERFRCPEAMFQPSFLGMESAGIHETTYNSIMKCDV

DIRKDLYAN

>Equ12085

KSRVAQEYRNIGRNSYIGDRVSFGSGMYGDSYPVKRKRKSATHIAYNEAEMRPAASDGSNLR

RKRFLFNMEPTEVLQTPSLFSIHQGTALDPNSGKRLEFGGDVMKRAFGSTLMSFLPPGFQPPP

GTEPGDFIKTVSPLLLANNGGGVNIKTRHRNGLFGDALPGKRKRKRSPDDSSKDVVRLGDVA

VDLFGRKGLDRFX

>Equ39344

RPRYLNDVLQTPDGKIYVSDSSDKFDAYRDLYIILEGRTSGRILELNPSTGGISVFADGIAYPNG

LELTSDGRSLLVA
>Equ16104
KLLKNLFKVMAEAGKMTGLLSTARLTSPGVPGTFAATPDMQMESDVHLTEAGCNDGKFKDI
AAQLIKDYKDINIIIGQGREYFMKPSSRNVSSNSSVYPWRRDLDLTTYWAMEKLLRKKTYKE
AVGTQAVKDAVRGQDNLQYFLGFLDEVQSSPASDDLQDLVSLVVSALNGKPNNNGYFLTIY
DDQLARDHENDAYNVGTDLNNILKAVNYLD
>Equ21457
RTXGKDLDKHQPRTTSVRPTTKHKVLSIHTALLLLSANLKSAHTMTLLTALCICCALVAVGK
CQDMTSFMFQALGMDPPPLPPIMPQSLREHLYPGARERQLMLQKQFQLQKQRYINYLIAQKQ
KQMKANPYNAAYSTNTRLQSQPQSRPQSQPQSQPQPRDNSVFSPANMIRQPGAVNSAAASKG
NPYSVPYQDGGARKGGAGNPYSSSRAAASSNPAVRNSALRTAFSSPSQAAKFYKTWQTMKV
MRDQPRRITDAKAAGCKLPVDSAAASVLMLNDCRNPAARMVCQAELMTCVNIGMTAMCCP
YGMNRLAMD
>Equ21104
TNAASVPPGGATILAIPLSVVSTSDDNIKSPLAAVKDVVADVAEKVADVVANVREKVADVVG
DVRDKVSDFVDDAKAGGSSHHDAGVISKTNENSNESTDTSDQTDTIDDKSEELKSDGGNSTE
GTNKDDDSNDKDKKANDDDDKSKNQSEKSSAEVRTRTYNNRGKQKDDSKEKVNDVDSGN
NVNGTDDSGEKQDAAKKETVDNNNDDDSREKGDAKSMWSWLKSLIGDIENESGYSDTKVR
QYLTQISKVRGYLDAMDSNFAASVAKQSGTCPVYPVQVGNNRVTITTENGCKIIIRLRGLPVD
KIEDGHEEKKKTHKRSVLVSYSDHX
>Equ21247
MTSIMRHTLALILLCGVWLISTTNTQQTTQRFQAEKFLDMLGKSYDSKHAYVELLSALHKRL
NLESAAQSNGSSSISDGGSTNFNAMAETVANCQEATPRQGASITYTSQNRVMGSRALYQCEQ
GYDGQSKFIQCGSGGQWTPLGSWAGCIPVDCGPAVPDVQNADRTLTSTKFPSVVVYTCQPG
YKMNVTNANITCLDTGFWDSPAFACEKDNIPSNPMMAALMQSMPGFASMPNTGLAGSPSML
PSSMNSNNPMAAMIAQMFQKSAAAQTPPPTPSPTTTTAPAVKTLPGGFQLPSHLRNLLPNGLS
STSGMRPQDPMALASMGLGSSNPAAAPRDLSQLLGTMNPMSLFGGAMGLNMGGAGAGG
>Equ25307
SIPTCGLLTSIRMGVDGQLLVLDAYRGLFQVNPITGAIRQLYSAINQVGGRLPRYLNDMVQTP
DGIVFISDSSDRFDAANDIYIIMEGRPSGRILALNPVTGAITEVLRDVLAYPNGLELTADGTALL
IAETGRARIFEX
>Equ12964
AKESHKGIVAFFANIKQARDYAVNEIVKFDNVITNDGNAYNKDTGIFTARKPGLFVFYFSTLS
AQGGLFLFDLFLNNEILASAYENVGSSYGQGGNSAIVRIKTGDQVCVKAKQASKIYNNPDRP
YATFSGYSLRREXGGRYVLEQLRNRLVCVTRGGGDFIILXKAEGX

>Equ15435

MAFVETLIGALLFACVAAQMPIQGFFNVPATAFNRGGRVDFGGSSLGQVCKQIMMMGGKEA
LSDGMGMAAKWFSNQRINGQAAGGNDPQMQCLYSQATGVTIQGAVGARYPGNMLGDCFD
TVIEYPMILDPGTGPQCDGKVNRTVLLMQTFAPMMRMGGLRGMGGKGAGRVGGMPGAKPF
AGMAQNPILSALMPRQNTAPAALGPAAN

>Equ09811

MVKTAPNLYIAQYLQATGQFTGQPRTEIIKFLEAGYQRQLSYQRVDGGFSAFGNSDESGSTW
LTSYVIRVFVDVRNFVFVDPSFLLRGTDWLVDRQNLDGSFNEFGRLLDTSIQGAGTGSALTAF
VLLALLKVQPLVRTDCVVDFDCPRVSRWGNATTNAIRNLERLVDSNSITDQLSLALASYALA
EANSSRAVPVFWQTGSTRSQRRSPTFLGRQLYNPPGQTSEFGLWRPPRVQTRAINVLITAYAI
QAYIAHNRIQDALPSVLWLTTQRNPSGGFVSSQDTSVALQALAAYATETADAYTDIDIKVSN
GAAVLAEFNITSQNALQLQIRELSRAAPRADVVATGTGFAVLEIVIQFNTNKQLSTPSFEVSTV
LIYDNLDSFNLLICTRWLWGRESGMVVQTISVPSGFSPDMESLGNIAGVRRSERRGNTVNVYF
DRIGTTSLCYSIRMTRSDKVVRTQENYIITSAYYNPSDQTVSFWQPKRLRDSTVCDVCLECCP*

>Equ21150

EQTVRNIDFKAFNSKVSGLLPTGVRFVYRENNNTFSADLEPEYIALDPETNKAYVCLQENNAV
AEVDLGTETVTQVYGLGYKQWGVLDASDRDLGIQLSYWPIRAWYQPDAIQFVSWKGRKLV
VSANEGDLKKYSNFREYQRGKQFTGLGDKIPDVVKTWLQEDSQLDRLKMSKLDGKDANGV
YQALYTYGARSFSIWDAADGFRRIYDSGSDIEKHTAFRCPHAFNTEGDDIDEKSDSKGPETES
LAVGQIGDRMYFFVGNENPGTILVYSVGDDVTQPRFETIFCDGLPDNKKTLQEKFDAREIYAL
DPEDLKFATGPESPTGSPVLIVAGSVSGTVSLLKIEI

>Equ21679

MTPLVACLTSALVFMGNLRVASSGADQYADLVKIHGLLKTADDRLVLLSTRNCQSGSGFVD
FCPIAGKLSFSEVFSCFYNTITSGPVVKEVEIYFDSTFITKPNVAVFTSGISHFVVVSYNITVTKV
TRTGLTIQAVGGPAPFSLGFNYVACDQQVLFPKAYLDIQDEIAFHSEQX

>Equ43690

RNIARPIQSRSPWQGSIWDLQQRRLQQLQSRLNGIGLTFWDRLSRNPAISQMMSPHWWFGNI
WSGLYGNRNX

>Equ47492

HVASTDPWSEVCLCPEWQKLADDDLTCLDRNACGAVITEPRGYISVTTTQHCIHXTSHVTGQ
YWDP

>Equ50224

RHDQRACAPLISGEAESDLGQRFITCSFGEVHYQHCQPGLRYDTATDSCNWPNWQLVVCLQ
M

Supplementary file S3. Matching peptides of frame shifted sequences

The peptide sequences that matched the sequences in the LC-MS/MS analysis are shown in red.

>contig16217_F3

PPNFKPPDLKPMLPPPMPMKQSPFLPPPMPMPMAPPGPPRSQPQTMVSSWTCPMQEVAHTEP
GHPYGW

>contig21060_F1

HGHAQCRRWRIRSRGTVRWGRTLWRWPLHGAPVMRSYHYCPPGPTTADHCKDQKLQEALY
FPDGTPRYNWVPPRNPWDTSLPDTVKETAKNILMMKVNSRPSRTPTPKEWELMSLLGDPKEQ
GPAGSNPFAGR*RIKISRESIGALPGCRNQWRLRRPTFTGAEET*SYSRFTPLTSTTFISRKFASF
ASRISHPKMCIILSYVSGPQTRSDREILPGKPVWKVFFITAHFNSIFDKEEFWGVQIV*S*FHAN
KDWLFSNRNVT*LVLKISCNMSMMIGCL*IS*IKT*FHSSHKFTFND*NCCNHGQGVQILMVL*
CFYRNSVTA*I*H*ISRSCEWRGELL*QDKKNVFSKSANHWTKILT*LFFCKSWVCSGKYMRIK
DIYFNIILLRLVSLLHE*RVLENRFCKIKGT*RVIYNGMQIS*SISKL*PR*LASHFAHNDV*MTG
SFIHTWWPYMYVQFELHDLLLTLTVIVMTALFISI*INQTVICNCK

>Equ16217-21060

PPNFKPPDLKPMLPPPMPMKQSPFLPPPMPMPMAPPGPPRVPTPNDGIFMDMPNAGGGAYGA
GAPYGGGAPYGGGPYMGPPVMRSYHYCPPGPTTADHCKDQKLQEALYFPDGTPRYNWVPP
RNPWDTSLLDTVKETAKNILMMKVNSRPSRTPTPKEWELMSLLGDPKEQGPAGSNPFAGR*

>contig15522_F2

GSHSV*GSTIAGRMGVLLVALLLCALITVAVGNASGSQRFQADKFLDMLGRSFDPKVAYVDL
LAELTHRLNIDVNSSSATSLASLIGAKRASAGNDKSACQEAPKVKGTTVTYTSDIHDVGSRAF
YQCDTGFDGMSKFIYCTQGGQWSTLASWEAVLQLTADS*LHM*LTLTPPSPQPPTGR*YSTLA
MQDTSWISQWECLMPGHWVLGQAKVLL*ESRGTFLTTSQPSESFQQKHGSPENTAASATSHH
PVGSNTITTRHHQATTTQNLPAR*REX

>contig15522_F3

EVTLCEAQRLLAGWECCWWPCCSAL**QWQSETRQVLRDFKQTNSWTCLADHLIQKLLMWI
CLLN*RID*I*MSIPVLLPRWRR**EPKEPAPGMTNQLVKRRLK*KGQPLRTPVTFTTWDLGRSI
SVTLDSTGCPSLFTARREASGQLWLAGKLFSS*LRTHNSTCD*R*PHLHLNHLRVADILHLPCR
IQAGSANGNVSCLDTGFWDKPKFSCEKAAAPSLRLPSLQKAFNKNMGVQKIQQLQQLATTLL
EATPSPPVIIKPQPRRICQHASAK

>contig15523_F2

PMMEAFMKSMQLFHALPKMPNIEQAKNSLAVPTVSSFANPMLCLRTCSQLRPSHAT

>Equ15522-15523

MGVLLVALLLCALITVAVGNASGSQRFQADKFLDMLGRSFDPKVAYVDLLAELTHRLNIDV
NSSSATSLASLIGAKRASAGNDKSACQEAPKVKGTTVTYTSDIHDVGSRAFYQCDTGYDGMS
KFIYCTQGGQWSTLASWEGCSPVDCGLITPHVTNADPTFTSTTYKSLIFYACHAGYKLDQPNG
NVSCLDTGFWDKPKFSCEKAAAPSLRLPSLQKAFNKNMGVQKIQQLQQLATTLLEATPSPPVI
IKPQPRQNLPAPLARKKTRYLRSLGPLTSSEPQSLPSSLTSSTANTSNPENPMMEAFMKSMQLF
HALPKMPNIEQAKNSLAVPTVSSFANPMAMFAHMFPTSP

>contig23617_F1
GKGA**LNGIFRRLCPVGPSRGPPSRSGTPAESGVGRQHGTP*SRGWAREVADEMPEEILQVAI
VGGLTGALGITGLLVGLHLSGNLVTDKKPETVSAASQTSDTLAPTFVNSAANSLGAAMIASSD
GVKDTVSNVVDGFKDAAGNVGDAVANVGDAVANVGDAVANVGDGVKD
>contig24364_F1
NVGDAVADVASGVKDVVEGVGDKTKDIVGDVGDVVKDVVGDVGDVVKDVVGDAGDAIK
DVVKDVVGDAGDAIKRCCEGCCWRC*RCYKKMLLVTLVMP*RMLQRMLVML*KMS*TPQV
MF*KTLLMILAMPRKTLQRAWRTWGTHQRRF*RGCRHLWG
>contig24364_F3
RW*CGR*RCFRC*RCC*RRW*QDERYCW*CWGCCEGCCW*CWGCCEGCCWRCWRCYKRC
CEGCCWRCWRCYKKML*RMLLAMLKML*KDVVSDAGDAVKDVAEDVGDVVKDVVDTAG
DVLKDVVDDIGDAAKDTAEGVENLGNTSAKVLARVQTSLG
>Equ23617-24364
GYFDVCAQLVRVVVPPAGVMGLPDLEAGPEKSPTKCLKKSFKWAIVGGLTGALGITGLLVGL
HLSGNLVTDKKPETVSAASQTADTLAPTFVNSAANSLGAAMIASSDGVKDTVSNVVDGFKD
AAGNVGDAVANVGDAVANVGDAVANVGDGVKDIVSDVAGGVKDAVGNVGDAVADVASG
VKDVVDGVGDKTKDIVGDVGDVVKDVVGDVGDVVKDVVGDAGDAIKDVVKDVVGDAGD
AIKDVVKDVVGDAEDVITDVSDAGDAVKDVAEDVGDVVKDVVDTAGDVLKDVVDDIGDA
AKDAAEGVGELGEHISEGFSEGADIFXEPRRNQALEGN

>contig44650_F1
WSTTGRRDSSRWAVGGVTNRGRGSDYVSLKWFADPAGVTCSLTTAAGTSFEAPESRWLLQL
KDGHRVRVVX
>contig44650_F3
VNDRPQGQLQVGRWRGHQPGSRLRLRLTQVVRRSCWRHVFTHDSSGNQLRGSRESLVAAIK
RWPPCSSCG
>contig23959_F1

WRINHGGRFY*AEE*SC*RILPGRAF*QRRTRI*SVRLHHRHLLQVQHDTHDRHFRQYGHFVR
NTSTTVTPSLTKQKISWMIDVKPWETVLKVNDKGLAIWGQKQNVKSAALKAAAIRMGIQFD
SSSGTLYVGADNTKVSTTPTDEDTVAQSVRVLGDRPIGSF

>contig23959_F2

GE*TMEAAFIRLKNNHVSAYFLDELSSKGGQGFDQFDFTTDTYYKFSTTHTTGTSDSTDILFET
PPQPLHPA*PNRRYRG*SMLNHGRRSSK*TIKVWLYGVRSRTSRALLSKLLPSEWVFSSILLAA
PFTLERTTLKCPPLLQTKTPLLKVSEF*ATDQSAVS

>Equ44650-23959

WSTTGRRDSSRWAVGGVTNRGRGSDYVSLKWFADPCWRHVFTHDSSGNQLRGSRESLVAAI
KDGHRVRVVVENKAMEAAFIRLKNNHVSAYFLDELSSKGGQGFDQFDFTTDTYYKFSTTHT
TGTFRQYGHFVRNTSTTVTPSLTKQKISWMIDVKPWETVLKVNDKGLAIWGQKQNVKSAAL
KAAAIRMGIQFDSSSGTLYVGADNTKVSTTPTDEDTVAQSVRVLDDRPIGSFNH*

>contig04504_F2

RSIQSC*LRPPQPQSQQRKDDPDHV*NLQRPSHVRRHPGRAVPVRFRSYHRYRP*LRRRCHPH
CSHL*RLRPAPRHPPSGLGRT*SHRLPDENPHRERLLIHHHG*ARDCPRHQRETLLRPLDFEQE
MATAASSSSLEKSYELPDGQVITIGNERFRCP*VLIPAILLGYGSAGIHETTYNSIMKCDVDIRK
DLYAN

>contig04504_F3

GASSPVD*GPLNPKANREKMTQIMFETFNAPAMYVAIQAVLSLYASGRTTGIVLDSGDGVTH
TVPIYEGYALPHAILRLDLAGRDLTDYLMKILTERGYSFTTTAEREIVRDIKEKLCYVPWTSSR
RWPQLHPPLPWRRATSCPTVRSSPLVTSVSGAPESLFQPSFLGMDLLVSMKPHTTPS*SAMLIS
VRISTPT

>Equ04504

EEHPVLLTEAPLNPKANREKMTQIMFETFNTPAMYVAIQAVLSLYASGRTTGIVLDSGDGVT
HTVPIYEGYALPHAIMRLDLAGRDLTDYLMKILTERGYSFTTTAEREIVRDIKEKLCYVALDF
EQEMATAATSSSLEKSYELPDGQVITIGNERFRCPEAMFQPSFLGMESAGIHETTYNSIMKCDV
DIRKDLYAN

>contig12085_F2

EEPCCPRISQHWAKQLYRGSGELWVRDVRRFLPSKEKAGKCNTHCI**S*NETCCFSWIKLET
EKVLV*YGAYRSSTDTFLVQYSPRNRSRPKLGQKVRIGGDVMKRAFGSTLMSFLPPGFQPPPG
TEPGDFIKTVSPLLLANNGGGVNIKTRHRNGLFGDALPGKRKRKRSPDDSSKDVVRLGDVAV
DLFGRKGLDRF*PPEKSPWFGSTFSDAIAMVTVLHALVG*KSCSSDVR*VPLEGRVSL*ASFGI
HTAAVPRHWRREVEMPPPNLLLKE*RNNY*LDSMGQNIQIKEFTGEQMV*IY*K*VSHSVLLC

YVGVLLNSSSIALLLLIFLSSSSSSFFLFFPSFSFSLVLYPCFAI*CQKGYYAST*LMSYTHNDIYS
LGNSPIQSTVSLNLRSRQHRYNNAGKP*NQLQQHYKNIHTY*SVLIIVVVDSDPKLLSCLLLPA
RCHSLNDIEDIWVWCCRHLPPEGHNESIFLYDCRTDGLLTSPFGSGGSHYAQRFSEPHKNLFP
ASHR

>contig12085_F3
KSRVAQEYRNIGRNSYIGDRVSFGSGMYGDSYPVKRKRESATHIAYNEAEMRPAASVGSNLR
RKRFLFNMEPTEVLQTPSLFSIHQGTALDPNSGKRLELEVMS*KEHLGAP*CRFCLQDFSRHQ
GQNLETSLKLFPHFCSLTTAEASTLRHVIETGYLETLFQVRGKERGLLTTAARMLSDLVTLL*I
CLGEKGWIVFNHQKNLHGLGQRFPMRLPW*LCCMLLLDRNRVQVMSDKFPLKVESHSKQAS
VFTLQRYLDTGEERWKCHRQISYLKSEGTTTNWTAWAKTYK*KNSLENRWSEYTENKYHIV
YCFVM*AFF*TLHL*LSSS*FSSPPPPPLSFFSFLLSLFRSFCIHVLPYSVRRDITRVLSLCLTHITIF
TAWVIHQSKAPFL*IYGLDSTDITMLGNPRINCSNITKTFTRIDRYSLLLLLILIPSC*VVYSCQP
DVIH*MTLKIFGFGAAGIYLPKGTTRVSFFTTAGQMVYSLLRLALEEVTMLRGSVSLTKIYSPL
VTG

>Equ12085
KSRVAQEYRNIGRNSYIGDRVSFGSGMYGDSYPVKRKRKSATHIAYNEAEMRPAASDGSNLR
RKRFLFNMEPTEVLQTPSLFSIHQGTALDPNSGKRLEFGGDVMKRAFGSTLMSFLPPGFQPPP
GTEPGDFIKTVSPLLLANNGGGVNIKTRHRNGLFGDALPGKRKRKRSPDDSSKDVVRLGDVA
VDLFGRKGLDRF*

>contig39344_F1
KTQVPERCGPNSRW*DIRQ*LQRQIRAYRDLYIILEGRASGRILELNPSTGEISVFADGIAYPNG
LELTSDGRSLLVA

>contig39344_F2
RRPRYLNDVVQTPDGKIYVSDSSDKFELTAIFTSSWKVGQAAES*S*IRQLVRSVYSQMG*HT
QTGWS*HLMAEVCWWX

>Equ39344
RPRYLNDVLQTPDGKIYVSDSSDKFDAYRDLYIILEGRTSGRILELNPSTGGISVFADGIAYPNG
LELTSDGRSLLVA

>contig16104_F2
EAAEEPLQGHG*SWQDDGPAEHGAPDLTWSRRNIRSHSRHADGVGRSPDRGGLQRWQVQG
HCCTAHQGLQGHKHHHRSRTGILHEAQQQERLLQQFSLSLEARPGSDNILGHGETVEEEDV*
RSCGHTGGQGRSERSGQSAIFSRLPG*GPELSGQRRPAGPGQSRGQRPQRQAQQQRILLTIYDD
QLARDHENDAYNVGTDLNNILKAVNYLD

>contig16104_F3

KLLKNLFKVMAEAGKMTGLLSTARLTSPGVAGTFAATPDMQMESDVHLTEAGCNDGKFKDI

AAQLIKDYKDINIIIGQGREYFMKPSSRNVSSNSSVYPWRRDLDLTTYWAMEKLLRKKTYKE

AVGTQAVKDALRGQDNLQYFLGFLDEVQSSPASDDLQDLVSLVVSALNGKPNNNGYF*PST

MTSWPVTTRTTPTTSALTSTTFSRPSTTWT

>Equ16104

KLLKNLFKVMAEAGKMTGLLSTARLTSPGVAGTFAATPDMQMESDVHLTEAGCNDGKFKDI

AAQLIKDYKDINIIIGQGREYFMKPSSRNVSSNSSVYPWRRDLDLTTYWAMEKLLRKKTYKE

AVGTQAVKDAVRGQDNLQYFLGFLDEVQSSPASDDLQDLVSLVVSALNGKPNNNGYFLTIY

DDQLARDHENDAYNVGTDLNNILKAVNYLD

>contig21457_F1

NEREGKT*TSISRGQHLSDQPLSTRSSRYIQLSCCCQPISSLPTP*HFLLPSVSVVRWSLLANART

*RHSCSKPWAWIRLRCPQ*CPSL*ENILPGDTGTTTHAPETVPAAEATIHKLSHRPEAEADESK

PL*RRL*YKHTASVTASVTTSVTASVTASAPRQLCLLPSKHDPTAGGCQLCRCLQR*PIQRAVS

RCGARKGGAGNPYSSSRAAASSNPAVRNSALRTAFSSPSQAAKFYKTWQTMKVMRDQPRRI

TDAKAAGCKLPVDSAAASVLMFNDCRNPAARMVCQTELMTCVNVGMTAMCCPYGMNRLA

MD

>contig21457_F2

TNVRERLRQASAEDNICQTNH*AQGPLDISSSLVAVSQSQVCPHHDTSYCPLYLLCAGRCWQ

MPGHDVIHVPSPGHGSASDAPNNAPVSERTFYPGTRERQLMLQKQFQLQKQRYINYLIAQKQ

KQMKANPYNAAYSTNTRPQSQPQSRPQSQPQSQPQPRDNSVFSPANMIRQPGAVNSAAASKG

NPYSVPYQDAVPGKVVREIPIAAPGPQHPVTLRCETVLSGLRFPRPHKQPSSTRPGRR*R**GTS

PAGSRTPKRPAVNFRWTVPRPACLCSTTAGIPRPAWCVRPN**RVSTLA*RPCAVLTA*TVWP

WT

>Equ21457

RT*GKDLDKHQPRTTSVRPTTKHKVLSIHTALLLLSANLKSAHTMTLLTALCICCALVAVGKC

QDMTSFMFQALGMDPPPLPPIMPQSLREHLYPGARERQLMLQKQFQLQKQRYINYLIAQKQK

QMKANPYNAAYSTNTRLQSQPQSRPQSQPQSQPQPRDNSVFSPANMIRQPGAVNSAAASKGN

PYSVPYQDGGARKGGAGNPYSSSRAAASSNPAVRNSALRTAFSSPSQAAKFYKTWQTMKVM

RDQPRRITDAKAAGCKLPVDSAAASVLMLNDCRNPAARMVCQAELMTCVNIGMTAMCCPY

GMNRLAMD

>contig21104 _F1

PANRPPTNAASVPPGGATILPFHSAL*PLATTTSNLRWQQ*RML*LTLLKKWPTWWLMSERR
LLTLLMT*ETRYLILSTMQRLVVVHITMLALSLKLTRTVTRLRTPATKLIQLMIKVRNLSPTAV
TARREPIRTMILTIRIRRLTTMTTNLKNQSGKSSAEVRTRTYNNRGKQKDDSKEKVNDVDSGN
NVNGTDDSGEKQDAGKKDTVDNNNDDDSREKGDAKSMWSWLKGLIGDIENESGDSDTKVR
QYLTQISKVRGYLDAMDSNFAASVAKQSGTCPVYPVQVGNNRVTITTENGCKIIIRLKGLTVD
KIEDGHEEKKKTHKRSVLVSYSDH*RRHVSKDEIEE*MTSKNCNCPAFDDETLHSLDKDIMHR
HRHTCMQLSITKTRWL*CRCMMTTYKQYACD*S*QL*DTEKGTENSHYSAFQN*CPQISFNIS*
IIAX
>contig21104_F3
C*QTADQRRKRPTRRSDHPAIPLSVVATSDDDIKSPLAAVKDVVADVAEKVADVVANVREK
VADVVDDVRDKVSDFVDDAKAGGSSHHDAGVISKTNENSNETTDTSDETDTIDDKSEELKSD
GGNSTEGTNKDDDSNDKDKKTNDDDDESKKPVWEIIS*STYTYL***RKAER*QQGKS*RCRQ
W**CKWNR*QWRETRCWQERHC**Q*R*RLEREGRRQKHVVMVERSHWRHRERVW*QRHE
SPPISDANLKGQRLPGCHGFELCCLRGKTIRHLPCISGPGWQ*PSHHNNREWMQNNYQTERTY
GGQN*GRA*REEENTQKICPCVLLGPLTTSCQQRRDRRMNDFKELQLSSI*R*NTTQSGQRHN
A*T*TYMHAT*HNKDSLALMSLYDDNI*TVCV*LKLTTVGHRKRN*EFTL*CVSKLMPSDFI*Y
LLNHCM
>Equ21104
TNAASVPPGGATILAIPLSVVSTSDDNIKSPLAAVKDVVADVAEKVADVVANVREKVADVVG
DVRDKVSDFVDDAKAGGSSHHDAGVISKTNENSNESTDTSDQTDTIDDKSEELKSDGGNSTE
GTNKDDDSNDKDKKANDDDDKSKNQSEKSSAEVRTRTYNNRGKQKDDSKEKVNDVDSGN
NVNGTDDSGEKQDAAKKETVDNNNDDDSREKGDAKSMWSWLKSLIGDIENESGYSDTKVR
QYLTQISKVRGYLDAMDSNFAASVAKQSGTCPVYPVQVGNNRVTITTENGCKIIIRLRGLPVD
KIEDGHEEKKKTHKRSVLVSYSDH*

>contig21247_F1
KVLQKHDFYNETYTGFDSALWSLVNINYKHTTNNAKISSREVSRHVRKII*FQTRIRRVTFSFT
*AVKS*VSSAV*WKLFYIRWWVYQFNAMAETVANCQEATPRQGASITYTSQNRVMGSRALY
QCEQGYNGQSKFIQCGSGGQWTPLGNWEGCIPVDCGPAVPDVQNADRTLTSTKFPSVVVYT
CQPGYKMNVTNANITCLETGFWDSPAFACEKDNIPSNPMMAALMQSMPGFASMPNTGLAGS
PSMLPSSMNSNNPMAAMIAQMFQKSVAAQTPPPTPSPTTTTAPAVKTLPGGFQLPSHLRNLLP
NGLSSTSGMRPQDLMALASMGLGSSNPAAAPRDLSQLLGTMNPCPYLEVRWVSTWVEPGQE
X
>contig21247_F2

RCYRNMTSIMKHTLALILLCGVWLISTTNTQQTTQRFQAEKFLDMLGKSYDSKHAYVELLSA
LHKRLNLESAAQSNGSSSISDGGSTNLTQWPKQLPTARRRHRDKVPA*LTQVRTVLWAPELSI
SVSKVTTASPSSYSVAVEVSGHRWATGRDAFQLTVGQQCPMSRTLTALSLQQSSRQ*LSTPA
NPATR*T*PMQI*RVWRQDSGTLQPSLVRRTTSHPIP*WLH*CKVCQVSPACQTQVWPALHPC
CPAP*TVTTQWQP*LLRCFKSQSQHKHHRLRHHQRQQLHQQLKRYLEASSSQVTYGICFLMA
CPAPLACVLRISWL*RQWAWDLAIQQLPQGT*VSC*GP*THVLIWRCDGSQHGWSRGRR
>Equ21247
MTSIMRHTLALILLCGVWLISTTNTQQTTQRFQAEKFLDMLGKSYDSKHAYVELLSALHKRL
NLESAAQSNGSSSISDGGSTNFNAMAETVANCQEATPRQGASITYTSQNRVMGSRALYQCEQ
GYDGQSKFIQCGSGGQWTPLGSWAGCIPVDCGPAVPDVQNADRTLTSTKFPSVVVYTCQPG
YKMNVTNANITCLDTGFWDSPAFACEKDNIPSNPMMAALMQSMPGFASMPNTGLAGSPSML
PSSMNSNNPMAAMIAQMFQKSAAAQTPPPTPSPTTTTAPAVKTLPGGFQLPSHLRNLLPNGLS
STSGMRPQDPMALASMGLGSSNPAAAPRDLSQLLGTMNPMSLFGGAMGLNMGGAGAGG

>contig25307_F2
SIPTCGLLTSIRMGVDGQLLVLDAYRGLFQVNPITGAIRQLYSAINQVGGRLPRYLNDMVQTP
DASFSSATPATGSMLPMTFTSSWRPAQRQDLGPEPSDRGNH*GPQRCPGIP*RPGTDG*RNSPA
HSRDRQSQDLRMSSX
>contig25307_F3
ASRLVVF*PASGWGWMVSCWCWTLTGACSR*TRSQVPSDSCIQLSTRSVGGCPGT*TTWFKH
RTHRFHQRLQRQVRCCQ*HLHHHGGRPSGRILALNPATGAITEVLRDVLAYPNGLELTADGT
ALLIAETGRARIFE*AP
>Equ25307
SIPTCGLLTSIRMGVDGQLLVLDAYRGLFQVNPITGAIRQLYSAINQVGGRLPRYLNDMVQTP
DGIVFISDSSDRFDAANDIYIIMEGRPSGRILALNPVTGAITEVLRDVLAYPNGLELTADGTALL
IAETGRARIFE*

>contig12964_F2
AKESHKGIVAFFANIKQARDYAANEIVKFDNVITNDATPTTRTLASSLLASRVCTCFTSAHYLP
RRCLSSRPVPQQ*DSGLCLRERSIVLWPGRKLRDCENQDR*PSLCESQASIQTLQ*PRPPVRHIQ
RVFS*A*IRGNTCA*TAEE*ISLCHGGGGGIYHFVKGRGT
>contig12964_F3
QKNHTKE*WLSLLISNRLETMQRMRSSSTMLSQTMQRLQQGHWHLHCSQAGSVRVSLQHT
ICQGGVFLLDLFHNNEILASAYENVASSYGQGGNSVIVKIKTGDQVFVKAKQASKLYNNPDR
PYATFSGYSLRRE*GGIHVLEQLRNRLVCVTVGGGGFIIL*KAEGX

>Equ12964

AKESHKGIVAFFANIKQARDYAVNEIVKFDNVITNDGNAYNKDTGIFTARKPGLFVFYFSTLS
AQGGLFLFDLFLNNEILASAYENVGSSYGQGGNSAIVRIKTGDQVCVKAKQASKIYNNPDRP
YATFSGYSLRRE*

>contig15435_F1

CCSLQLISSSKTINTHFCVVH*KISCQRIFINSMALVETLIGALLFACVAAQMPIQGFFNVPATAF
NRGGRVDFGGSSLGQVCKQIMMMGGKEALSDGMGMAAKWFSNQRINGQAAGGNDPQMQ
CLYSQATGAMMPGAVGARDPGNMFGDYFDTVIEYPMILDLHGTSV*RQSQPYSFTHADICSH
DEDGRYERYGRHGCRDGWRYARSQSIRGNGTESTLVCSDAPTEHRPRCPGPSSQX

>contig15435_F2

VVLYS**AQAKR*IHIFVWSIKKLVVSAYL*TAWR*SKH*SVHFCLPVSQLKCPSKDSSMSQQQ
PSTEADV*TLVDQV*DRCASKS**WVAKKHYLMVWGWLPNGFLIKESTVKQLVETTHRCSVS
TRRLRAL*CLVLSEQEIQAICLATILTQ*SSIR*FWTCTGPQCDGKVNPTVLLMQTFAPMMRMG
GMRGMGGMGAGMGGGMPGANPFAGMAQNPLLSALMPRQNTAPAALGPAAN

>Equ15435

MAFVETLIGALLFACVAAQMPIQGFFNVPATAFNRGGRVDFGGSSLGQVCKQIMMMGGKEA
LSDGMGMAAKWFSNQRINGQAAGGNDPQMQCLYSQATGVTIQGAVGARYPGNMLGDCFD
TVIEYPMILDPGTGPQCDGKVNRTVLLMQTFAPMMRMGGLRGMGGKGAGRVGGMPGAKPF
AGMAQNPILSALMPRQNTAPAALGPAAN

>contig09811_F1

SMVKTAPNLYIAQYLQATGQFTGQPRTEIIKFLEAGYQRQLSYQRVDGGFSAFGNSDESGST
WLTSYVIRVFVDVRNFVFVDPAFLLRGTDWLVDRQNLDGSFNEFGRLLDTSIQAPGLEVL*Q
HSCCWLCSKCSHWSELIASWTLIVPECPAGEMQQLTP*ETWRDWWTPTPSLTSSLWLWPATP
WQRPTAAEPCRSLADWKHSLATKVAYVSGQTTLQSPWPDF*VRPLASTESPDSGNQRPDHSI
RNPLTSPIIESKMPSHLFCG*PPKEIRLVGSYLHRTLRLLYKL*LPTPRRQLMLTRTLTSRSPMGP
RSWQSSTSPARTPCSCRSES*AEQRRGPMWSPRGQALLSWRLSYSSTPTNS*VRPRLRSAQC*F
MTTWTRSTCSSAPGGCGAESPGWWCRPSVCRPASVRTWRVWGTLLVSGDPSGEATPSTFTST
GSAPHPSATASG*PAQTRWSGPRRTTSSPRPTTTPVTRPCLSGSPRDSGILQSVMSA*SAAPRPA
HQPQPSLSHSSEVTFR*LLHSSDLSHSSDYHIQVI

VTF

>contig09811_F3

HGKDGPQPLHCSIPAGHRSVHWSAED*DNQVPGGGLPEAAELPASGRRLQCFWEFR*EWQHL
VDELCHQSVRGCQELCVCGSCISTARHRLAGGQTEPRWLLQRVRQTSGHQHPGAGTGSALT

AFVLLALLKVQPLVRTDCVVDFDCPRVSRWGNATTNAMRNLERLVDSNSITDQLSLALASY
ALAEANSSRAVPVFGRLEALARNEGRLRFWADNSTIPLARLLSSASGVHRESRLGQSTS*SQH
TQSTYIAHNRIQDALPSVLWLTTQRNPSGGFVSSQDTSVALQALAAYATETADADTDIDIKVS
NGAAVLAEFNITSQNALQLQIRELSRAAPRADVVATGTGFAVLEIVIQFNTNKQLSTPSFEVST
VLIYDNLDSFNLLICTRWLWGRESGMVVQTISVPSGFSPDMESLGNIAGVRRSERRGNTVNVY
FDRIGTTSLCYSIRMTRSDKVVRTQENYIITSAYYNPSDQTVSFWQPKRLRDSTVCDVCLECCP
*ASPPATAIIVTFK*SHI*VIVAFK*FVTFK*LSHSSDCYIX

>Equ09811

YIAQYLQATGQFTGQPRTEIIKFLEAGYQRQLSYQRVDGGFSAFGNSDESGSTWLTSYVIRVF
VDVRNFVFVDPSFLLRGTDWLVDRQNLDGSFNEFGRLLDTSIQGAGTGSALTAFVLLALLKV
QPLVRTDCVVDFDCPRVSRWGNATTNAIRNLERLVDSNSITDQLSLALASYALAEANSSRAV
PVFGRLEALARNEGRLRFWADNSTIPPGQTSEFGLWRPPRVQTRAINVLITAYAIQAYIAHNRI
QDALPSVLWLTTQRNPSGGFVSSQDTSVALQALAAYATETADAYTDIDIKVSNGAAVLAEFN
ITSQNALQLQIRELSRAAPRADVVATGTGFAVLEIVIQFNTNKQLSTPSFEVSTVLIYDNLDSFN
LLICTRWLWGRESGMVVQTISVPSGFSPDMESLGNIAGVRRSERRGNTVNVYFDRIGTTSLCY
SIRMTRSDKVVRTQENYIITSAYYNPSDQTVSFWQPKRLRDSTVCDVCLECCP*

>contig21150_F1

NGPEGTEQTVRNIDFKAFNSKVSELLPTGVRFVYRENNNTFSADLEPEYIALDPETNKAYVCL
QENNAVAEVDLGTETVTQVYGLGYKQWGVLDASDRDLGIQLSYWPIRAWYQPDAIQFVSW
KGRKLVVSANEGDLKKYSNFREYQRGKQFTGLGDKIPDVVKTWLQEDSQLAD*KCPSWTVR
TRTASTRRSTPTGLGASPSGRR*RLPADLRQRK*HREAHGLPVSSCVQHRGR*YRREV*QQGP
RNRIPGRGTDRGQNVFLRGKRESWNYFSLQRWR*RHAASL*DYFLRRASRQQENPPGKFDAR
EIYALDPEDLKFATGPESPTGSPVLIVAGSVSGTVSLLKIEIS*KMADSLGLSEYFGSGRAKVTV
TRRMASGIVSLTSLSFSSQLIYQDLFACGFHFLVH

>contig21150_F2

MAQKALNRQ*ETSTSRLSTAKCRSCCPLGSGSCTARTTTHSAQTWSPSTSPLTPRPTRPTSACR
RTMPWPRWIWARRQSRKSMGWATSSGGSWMPATEIWVFS*ATGQSEPGTNPTPFSSSAGRE
GSWLSLPTKATSRSTATSGSISGENSSRDLVTRFQMS*RPGFRRTHSWQTENVQAGR*GRERR
LPGALHLRGSELLHLDAADGFRRIYDSGSDIEKHTAFRCPHAFNTEGDDIDEKSDSKGPETESL
AVGQIGDRMYFFVGNENPGTILVYSVGDDVTQPRFETIFCDGLPDNKKTLQENSTPGKSML*I
LKTSSSLLARKVRLALRSSSWPGQFPEQSPC*RSKSAKRWPTALDFQNTLAVAERK*L*RDEW
RQGLYH*PRCRFHHNSYIRTYLLVVFIFLYX

>Equ21150

EQTVRNIDFKAFNSKVSGLLPTGVRFVYRENNNTFSADLEPEYIALDPETNKAYVCLQENNAV
AEVDLGTETVTQVYGLGYKQWGVLDASDRDLGIQLSYWPIRAWYQPDAIQFVSWKGRKLV
VSANEGDLKKYSNFREYQRGKQFTGLGDKIPDVVKTWLQEDSQLDRLKMSKLDGKDANGV
YQALYTYGARSFSIWDAADGFRRIYDSGSDIEKHTAFRCPHAFNTEGDDIDEKSDSKGPETES
LAVGQIGDRMYFFVGNENPGTILVYSVGDDVTQPRFETIFCDGLPDNKKTLQEKFDAREIYAL
DPEDLKFATGPESPTGSPVLIVAGSVSGTVSLLKIEI

>contig21679_F1
PEITNTERLSIQEDL*NCTD*SQQQVDKMTPLVACLTSALVFMGSLRIVSSGADQYADLVKIHG
LLKTADDRLVLLSTRNCQSGSGFVDFCPIAGKLSFSEVFSCFYNTITSGPVVKEVEIYFDSTSSP
NQTWLFSHPGYPIL*WSHTTSPLPR*PELDSLFRQ*EGLHLSLCDLITWPVTSRFSFPKRTWISK
TRLPSTASSRTVVNPATQLIICTDMHGAYTEP*NYRQQARTNEC*FSCCFFLHSSFFFSFFLLLL
LLLLLLLLLLLLLLLLLLLLL
>contig21679_F3
*DYKY*ATLYTGRFIELHRLIPATSRQNDTPGCLSD*CPGLHGQLTNRLQRRRPVR*PGEDTRT
VKDCR*PFSAAIDKELSEWIRIC*FLPYCRKTQFLGGLQLFLQHNHKWTSSEGS*NLLRLDFITK
PNVAVFTSGISHFVMVSYNITVTKVTRTGLTIQAVGGPAPFSLRFNYVACDQQVLFPKAYLDI
QDEIAFYSEQ*DGCKSCNTTNYLYRYARSLH*AMKLPSASKDKRMLI*LLFLPSFFLLLFFLPPS
SSSSSSSSSSSSSSSSSSSSSS
>Equ21679
MTPLVACLTSALVFMGNLRVASSGADQYADLVKIHGLLKTADDRLVLLSTRNCQSGSGFVD
FCPIAGKLSFSEVFSCFYNTITSGPVVKEVEIYFDSTFITKPNVAVFTSGISHFVVVSYNITVTKV
TRTGLTIQAVGGPAPFSLGFNYVACDQQVLFPKAYLDIQDEIAFHSEQ*

>contig43690_F1
PGTSRGQSSQGHPGKAASGICSRGGFSNYRAV*TELD*RWDRLSRNPAISQMMSPHWWFGNI
WSGLYGNRNX
>contig43690_F3
RNIARPIQSRSPWQSSIWDLQQRRLQQLQSRLNGIGLTLGPVESQSGYFPNDVAPLVVRQ
YLEWAVWEPQP
>Equ43690
RNIARPIQSRSPWQGSIWDLQQRRLQQLQSRLNGIGLTFWDRLSRNPAISQMMSPHWWFGNI
WSGLYGNRNX
